# Electronic and Steric Effects of the ProPhos Ligand Family for Nickel-Catalyzed Suzuki–Miyaura Coupling of Heteroaromatics

**DOI:** 10.1021/acscatal.5c07745

**Published:** 2025-12-29

**Authors:** Jin Yang, Hengyuan Zhao, Johnathan E. Schultz, Steven R. Wisniewski, Eric M. Simmons, Tianning Diao

**Affiliations:** † Department of Chemistry, 5894 New York University, 100 Washington Square East, New York, New York 10003, United States; ‡ Chemical Process Development, Bristol Myers Squibb Company, New Brunswick, New Jersey 08903, United States; § Department of Chemistry, 2007 Yeshiva University, 2495 Amsterdam Avenue, New York, New York 10033, United States

**Keywords:** Suzuki–Miyaura, nickel, ProPhos, heterocycles, active pharmaceutical ingredients

## Abstract

Nickel-catalyzed Suzuki–Miyaura coupling (Ni-SMC) reactions offer a cost-effective approach for active pharmaceutical ingredient (API) synthesis but remain constrained by their limited compatibility with heteroaromatic substrates and the need for high catalyst loadings. Our laboratory recently developed the ProPhos ligand, which significantly addresses these challenges. In this study, we systematically investigate the electronic and steric effects of the ProPhos ligand framework. Evaluation of over 20 ProPhos derivatives, alongside representative monodentate and bidentate phosphine ligands, reveals that the beneficial influence of the tethered hydroxyl group is general. Other nucleophilic substituents were less effective, and variations in the three-carbon linker length compromised the catalytic efficiency. Increased steric bulk on phosphorus decreased reactivity, whereas electron-donating substituents such as *para*-tolyl enhanced performance; in contrast, replacing aryl groups with cyclohexyl groups proved detrimental. Finally, the application of the optimized ligand, ProPhos*, to a range of previously challenging substrates demonstrated the robustness and efficiency of Ni-SMC at low catalyst loading.

## Graphical abstract

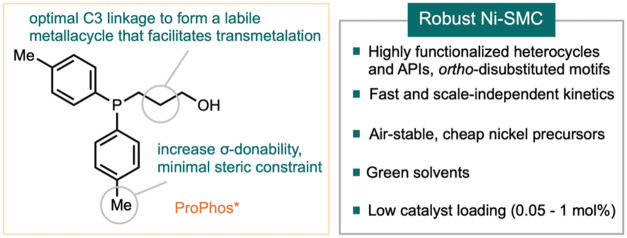

## Introduction

The construction of active pharmaceutical ingredients (APIs) bearing heteroaromatic frameworks is most commonly achieved through palladium-catalyzed Suzuki–Miyaura coupling (Pd-SMC),^1–3^ which accounts for roughly one-third of all catalytic transformations used in process-scale synthesis.^4,5^ Although the nickel-catalyzed variant (Ni-SMC) presents an attractive, cost-effective, and sustainable alternative,^6,7^ its industrial adoption has remained limited.^8,9^ Broader implementation of Ni-SMC has been constrained by several persistent challenges,^10,11^ including the requirement for relatively high catalyst loadings (5–10 mol %),^12^ sluggish reaction kinetics,^13^ reliance on air-sensitive and costly precatalysts or ligands,^14^ a narrow substrate scope for heteroarene coupling partners, and insufficient catalyst robustness under process conditions.^15^

In response to this longstanding challenge, our lab recently developed the ProPhos ((diphenylphosphino)­propanol)^16^ and *tri-*ProPhos ligands,^17^ which incorporate hydroxyl groups tethered to the phosphine moiety. Both ligands exhibit excellent complementary reactivity across a broad range of heteroaromatic nucleophiles and electrophiles (Scheme 1A). In a THF:H_2_O solvent mixture, ProPhos facilitates colocation of the catalyst and boronic acids and esters^18^ through coordination via its hydroxyl group (**1** → **2**).^16^ In *i-*PrOH or H_2_O, *tri-*ProPhos undergoes intramolecular ligand substitution through its tethered hydroxyl group (**1** → **4**).^17^ Both pathways promote transmetalation through the formation of intermediate **3**, which resembles a “nickel-hydroxo” species^19^ from which transmetalation proceeds.

**Scheme 1:**
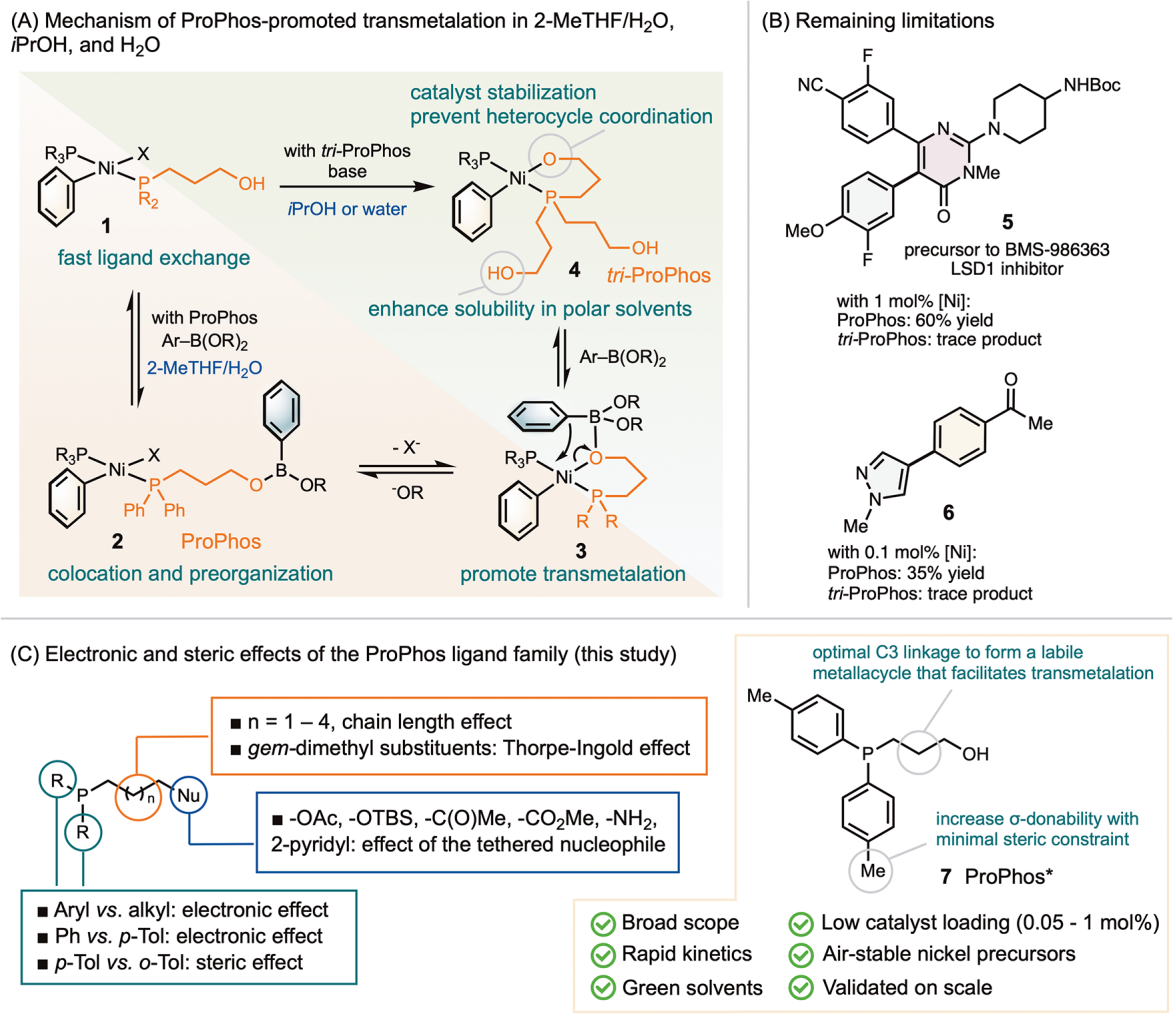
Development of the ProPhos Ligand Family and Motivation of Investigating Ligand Derivatives. (A) Proposed Mechanism of ProPhos-Promoted Transmetalation; (B) Remaining Synthetic Limitations; and (C) Proposed Modifications of ProPhos

In our initial report, we did not examine the compatibility of ProPhos with a broad range of heterocyclic substrates and employed Ni­(cod)_2_ as the precatalyst, which lacks air stability and is unsuitable for large-scale or process applications.^16^ In testing the generality of the ProPhos ligand family, we identified limitations when targeting highly challenging products such as **5**, the final intermediate en route to clinical candidate LSD1 inhibitor BMS-986363 (CC-90011),^20^ and **6**, the core structure of PCSK9 inhibitor PF-06815345 ^21^ (Scheme 1B).

These limitations motivated a comprehensive and systematic evaluation of structural modifications to ProPhos, including variation of the aryl and alkyl substituents on phosphorus to tune electronic and steric effects, adjustment of linker length and substitution pattern, and exploration of alternative tethered nucleophiles beyond hydroxyl groups (Scheme 1C). In this study, we employed the inexpensive and air-stable precatalyst NiCl_2_·6H_2_O ($21/mol)^8,12^ to advance the method toward process-relevant implementation. Through this effort, we discovered that ProPhos* **7**, featuring electron-rich aryl substituents on phosphorus, markedly enhances catalyst reactivity and stability. The new system operates efficiently at low loadings (≤1 mol %), a threshold that makes it suitable for adoption in pharmaceutical process synthesis, and exhibits a substantially expanded substrate scope. Notably, Ni-SMC with ProPhos* enables coupling of previously inaccessible and highly challenging substrates while retaining all of the advantageous features of the parent ProPhos system.

## Methods

We first synthesized a series of ProPhos derivatives (Figure 1). Mixed alkyl–aryl ProPhos ligands, such as ProPhos and ProPhos* **7**, exhibit sufficient stability to be purified by flash chromatography in air. However, they undergo gradual oxidation on the bench over the course of weeks. ProPhos is a white crystalline solid and oxidizes more slowly than oily ProPhos* **7**. In contrast, ^Cy^ProPhos **8** is highly air-sensitive and must be synthesized and handled under an inert atmosphere. *Tri*-ProPhos is air-stable at room temperature but undergoes oxidation over time and should be stored under an inert atmosphere.^17^

**Figure 1:**
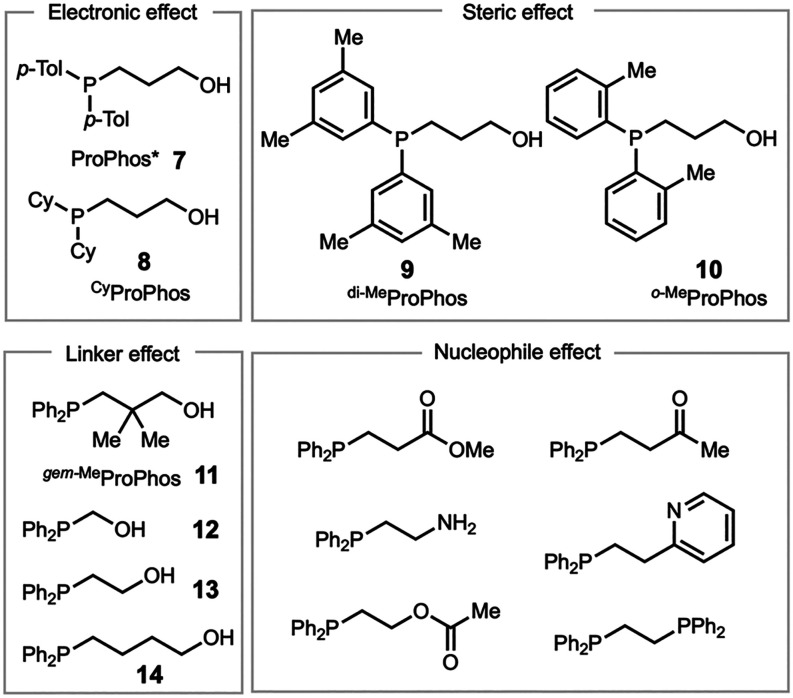
ProPhos derivatives were synthesized and evaluated for establishing structure–activity relationships.

We evaluated the reactivity of the ProPhos ligand family in comparison to common monodentate (blue bars)^22–26^ and bidentate (orange bars)^27–29^ phosphine ligands. All reactions apply 2.5 equiv of K_3_PO_4_ as the base with 1 mol % NiCl_2_·6H_2_O as the precatalyst and *i*-PrOH as the solvent. We compared each of the ligands using three model Ni-SMC reactions of heterocyclic nucleophiles and electrophiles: 4-bromophenylethanone **15** with 3-pyridinylboronic acid **16** (reaction I), 3-chloropyridine **18** with *para-*fluorophenyl boronic acid **19** (reaction II), and 3-chloropyridine **18** with pyrimidinyl boronic acid **21** (reaction III). Among the model reactions, the reaction between **15** and **16** appeared to be the most challenging, likely due to catalyst poisoning by the sterically accessible, nucleophilic pyridine.^25^

ProPhos derivatives with free hydroxyl groups (Scheme 2, green bars) exhibited excellent reactivity across all three model reactions, regardless of the length of the carbon linkage between the phosphine and hydroxyl groups, except for Ph_2_PCH_2_OH **12**, which showed low reactivity. In contrast, ProPhos derivatives with protected hydroxyl groups displayed varying degrees of success (yellow bars). Phosphine ligands tethered with other nucleophiles, such as amine, pyridine, ketone, and ester groups, did not exhibit the same level of reactivity as ProPhos, suggesting that the hydroxyl group plays a crucial role in reactivity. Bidentate phosphine ligands demonstrated the lowest reactivity overall (orange bars), while monodentate ligands, including P*i*Pr_3_, PPh_3_, PBn_3_, and PCy_3_, were successful in model reaction III (blue bars). In model reaction I, both bidentate and monodentate ligands gave low yields, except 1,2-bis­(dimethylphosphino)­ethane (dmpe), which showed modest reactivity.

**Scheme 2:**
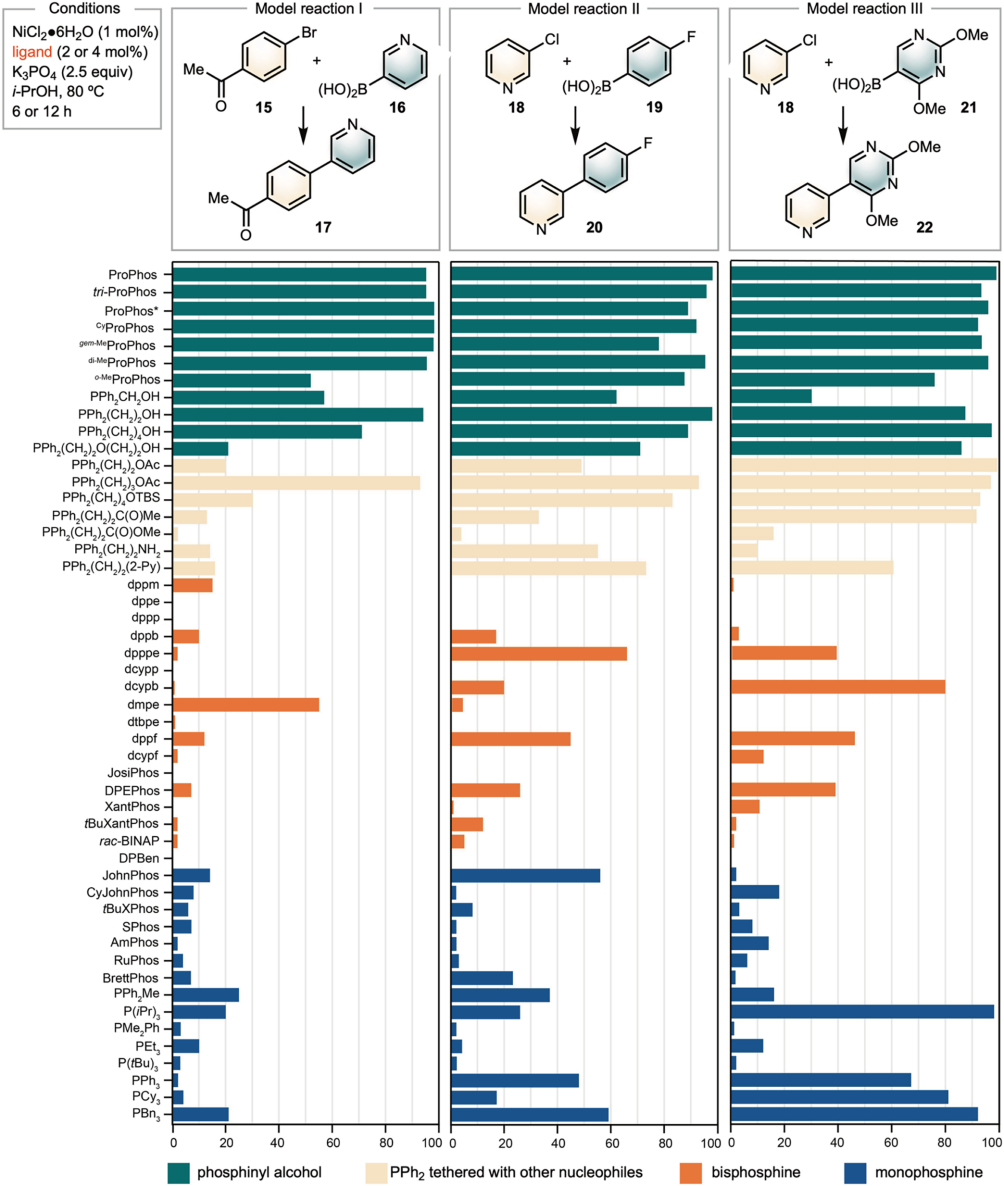
Comparison of ProPhos Ligand Family with Precedented Catalysts

ProPhos derivatives bearing free, ester-protected, or silyl-protected hydroxyl groups (green and part of the yellow bars) fully solubilized NiCl_2_·6H_2_O in *i*-PrOH (cf. Table S2). In contrast, phosphine ligands with tethered ketones, amines, carboxylates, and pyridines, as well as most monodentate and bidentate phosphine ligands, resulted in heterogeneous catalyst mixtures (cf. Table S2), leaving NiCl_2_·6H_2_O barely dissolved.

Kinetic analysis of model reaction I with selected ligands at 0.1 mol % nickel loading revealed that the reactivity of the ProPhos ligand family is highly sensitive to the steric environment at the phosphorus center (Figure 2A). Among the ligands tested, ProPhos exhibited the highest reactivity, reaching full conversion within 3 h, while ^di‑Me^ProPhos showed slightly reduced reactivity. Other ligands, including ^Cy^ProPhos, ProPhos-OAc, ^*gem‑*Me^ProPhos, and ^*o‑*Me^ProPhos, displayed significantly lower reactivity. Tabulation of the initial rates and corresponding %*V*_bur_ (min) values for each ligand (Figure 2B)^30^ revealed a correlation (Figure 2C). Ligands with %*V*_bur_ (min) values greater than 27 showed markedly diminished reactivity, consistent with prior studies on ligand steric effects.^24^ Notably, although ProPhos-OAc shares the identical %*V*_bur_ (min) as ProPhos, its substantially lower activity highlights the beneficial role of the tethered hydroxyl group. In the case of ^*gem‑*Me^ProPhos, the reduced reactivity may arise from the formation of an overly stable metallacycle, analogous to intermediate **4**, which fails to undergo efficient ring opening during transmetalation.

**Figure 2:**
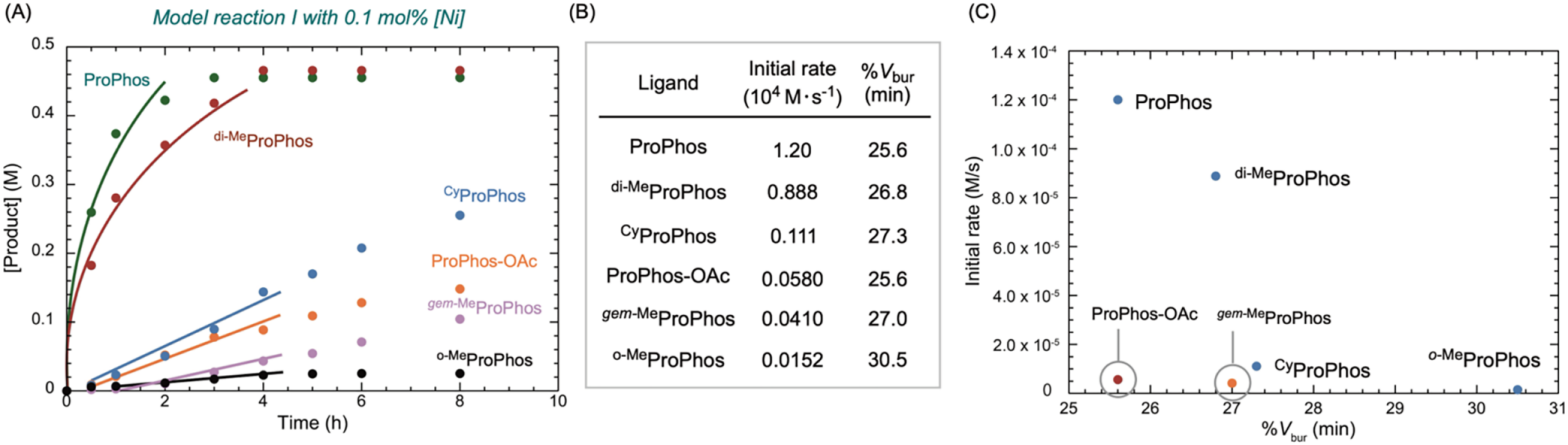
Steric effect of the ProPhos ligand family. (A) Comparison of initial rates for model reaction I using various ligands at 0.1 mol % catalyst loading. Reaction conditions: [**15**] = 0.67 M, [**16**] = 1.02 M, K_3_PO_4_ (2.5 equiv); [Ni] = 0.67 mM; [ligand] = 2.7 mM, solvent = *i-*PrOH, 80 °C. (B) Summary of initial rates. (C) Correlation of initial rates with the %*V*_bur_ (min) of ProPhos ligands.

Evaluation of linker length within the ProPhos ligand family at 0.1 mol % catalyst loading for model reaction I revealed that a C3 linker delivers optimal reactivity (Figure 3). Both the C1 and C4 linkers (**12** and **14**) resulted in low yields and slow conversion, whereas the C2 linker (**13**) showed slower kinetics but ultimately furnished a high yield of product **17**(Figure 3). This result contrasts with our previous findings using **13** under 2-MeTHF/H_2_O conditions.^16^ Nonetheless, all ProPhos derivatives tested outperformed the PPh_2_Me ligand previously identified via high-throughput experimentation (HTE).^25^

**Figure 3:**
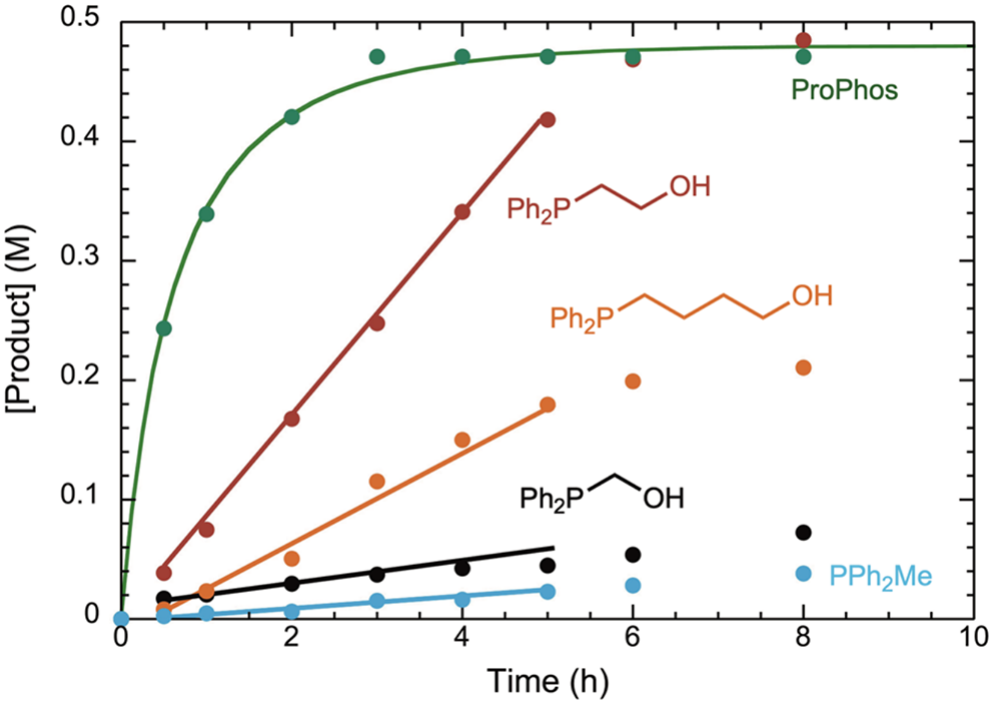
Chain length effect of the ProPhos ligand family. Comparison of initial rates for model reaction I using various ligands at 0.1 mol % catalyst loading. Reaction conditions: [**15**] = 0.67 M, [**16**] = 1.02 M, K_3_PO_4_ (2.5 equiv); [Ni] = 0.67 mM; [ligand] = 2.7 mM, solvent = *i-*PrOH, 80 °C.

We next compared the performance of four ligands, ProPhos, ProPhos* **7**, ^Cy^ProPhos **8**, and ^*gem‑*Me^ProPhos **11**, in model reaction I at reduced catalyst loadings (Figure 4A). At 0.1 mol % catalyst loadings, both ProPhos and ProPhos* **7** delivered yields above 90%, while **8** and **11** gave only modest yields. Upon further lowering the catalyst loading to 0.04 mol %, the yield with ProPhos decreased to 68%, whereas ProPhos* **7** maintained a high yield of 90%. Time course of the reactions with ProPhos and ProPhos* at 0.04 mol % revealed comparable initial rates; however, the reaction with ProPhos plateaued earlier (Figure 4B). These results indicate that ProPhos* **7** is more robust at low catalyst loadings, establishing it as a promising platform for extending Ni-SMC to the synthesis of APIs.

**Figure 4:**
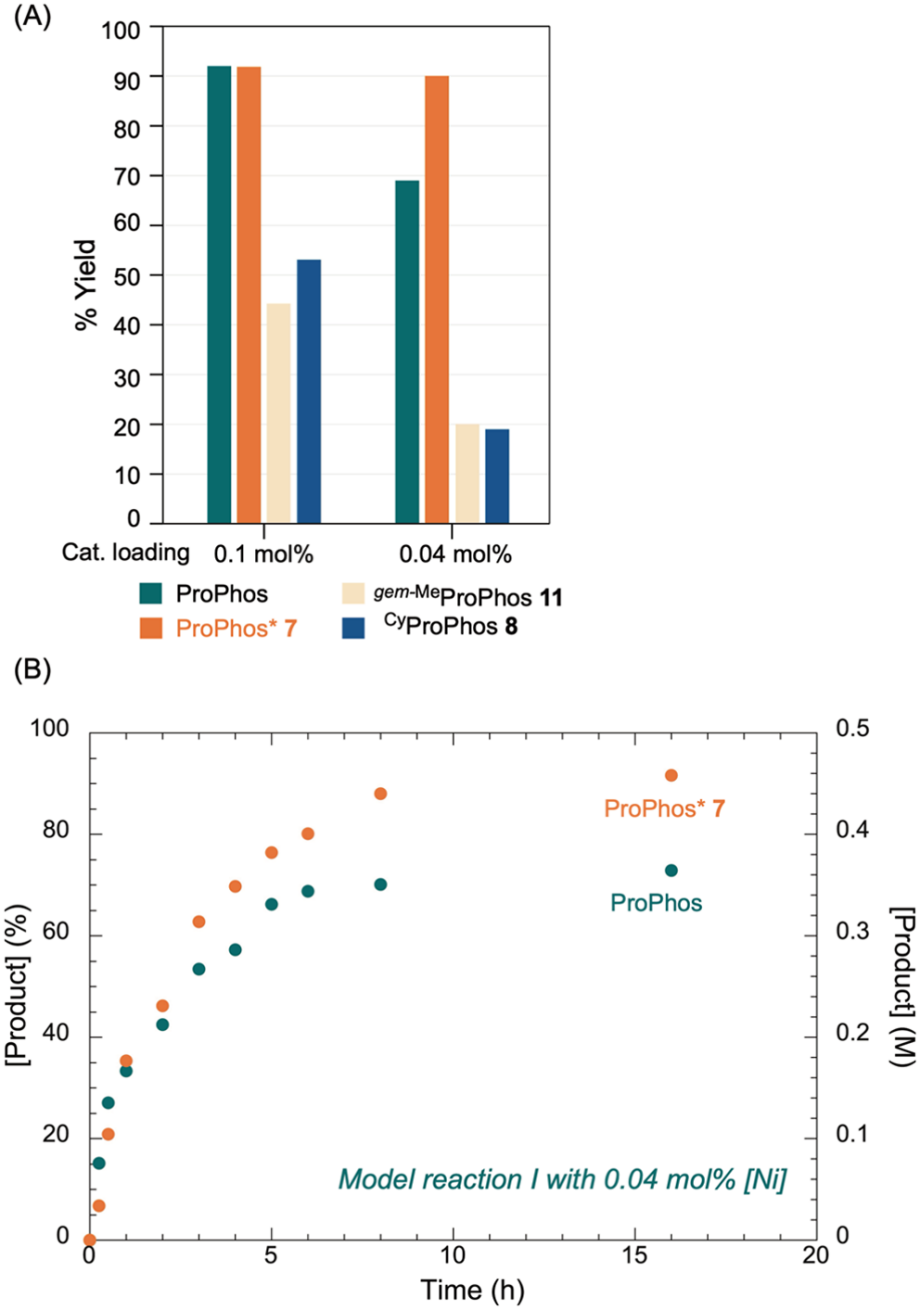
(A) Comparison of catalytic activity for model reaction I using selected ProPhos ligands under extremely low catalyst loadings for Model reaction I with a reaction time of 16 h. [**15**] = 0.67 M, [**16**] = 1.02 M, and K_3_PO_4_ (2.5 equiv); solvent = *i*-PrOH, 80 °C. (B) Time course of model reaction I at 0.04 mol % catalyst loading with ProPhos and ProPhos* **7**. Reaction conditions: [**15**] = 0.67 M, [**16**] = 1.02 M, K_3_PO_4_ (2.5 equiv); [Ni] = 0.27 mM; [ligand] = 1.1 mM, solvent = *i-*PrOH, 80 °C.

To understand why ProPhos* is more robust than ProPhos, we compared their coordination affinities to nickel­(II) to assess their resistance to catalyst deactivation via heterocycle coordination or ligand displacement (Figure 5A). When the isolated Ni­(ProPhos)_2_Cl­(*o*-Tol) **23** was treated with 2.2 equiv of ProPhos*, the ^31^P­{H} NMR spectrum displayed new resonances, including an AB quartet at 14.9 and 12.9 ppm and a singlet at 13.3 ppm. The singlet at 13.3 ppm is consistent with the independently synthesized complex Ni­(ProPhos*)_2_Cl­(*o*-Tol) **25**. The AB quartet was assigned to the mixed-ligand species Ni­(ProPhos)­(ProPhos*)­Cl­(*o*-Tol) **24**, with the large coupling constant (^2^ *J*_PP_ = 301 Hz) consistent with a *trans* coupling.^31^ Integration of the ^31^P­{H} signal yielded equilibrium constants *K*_eq_ of 3.5 and 0.89 for the conversions of **23** to **24** and **24** to **25**, respectively. Upon addition of 10 equiv of pyridine to the isolated complex **25**, less than 5% coordination to yield the pyridine adduct **26** was observed (Figure 5B). This contrasts with PPh_3_, which has previously been shown to be displaced by pyridine.^15,25,32^ Collectively, these results demonstrate that ProPhos* binds more strongly to nickel than does ProPhos, thereby stabilizing the catalyst and preventing deactivation via heterocycle coordination.

**Figure 5:**
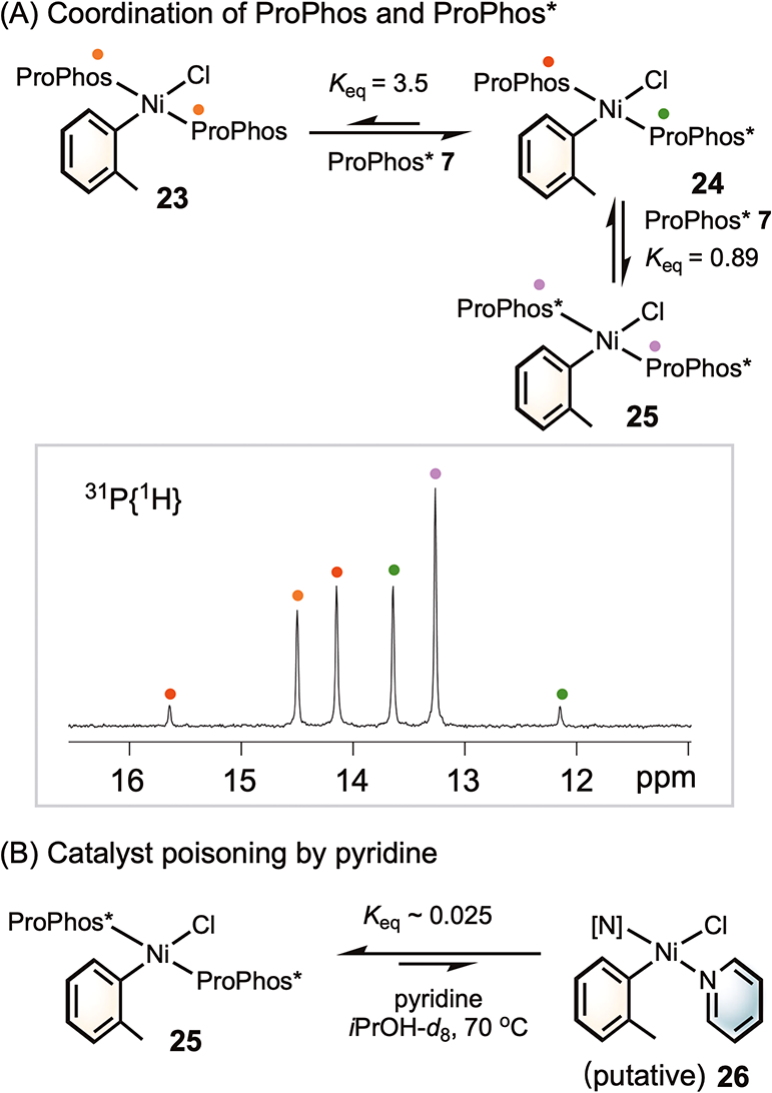
Study of the coordination affinities of ProPhos*. Reaction conditions: (A) [**23**] = 13 mM, ProPhos* (2.2 equiv) in *i*-PrOH-*d*_8_ (0.6 mL) at room temperature; (B) [**25**] = 13 mM, pyridine (10 equiv) in *i*-PrOH-*d*_8_ (0.6 mL) at 70 °C.

We then investigated the application of ProPhos* **7** in promoting Ni-SMC of heterocyclic coupling partners at low (≤1 mol %) catalyst loadings (Scheme 3). In general, (ProPhos*)Ni exhibits high activity in *i*-PrOH (conditions A), particularly with heterocyclic boronic acids. Many substrates undergo efficient coupling at catalyst loadings as low as 0.05 mol %. However, certain sterically hindered substrates, such as those in the synthesis of **5**, suffer from competitive protodehalogenation of the electrophile in alcohol solvents,^5^ potentially due to slow transmetalation. To mitigate this issue, we employed a 2-MeTHF/H_2_O solvent system along with air-stable Ni­(II) precatalyst Ni­(PPh_3_)_2_(Naph)Cl (conditions B), which enables rapid *in situ* generation of the active (ProPhos*)Ni species at room temperature. This homogeneous biphasic system effectively suppresses dehalogenation, though it typically requires higher catalyst loadings (1–3 mol %). Overall, both conditions A and B provide versatile and complementary platforms for scalable process synthesis.

**Scheme 3:**
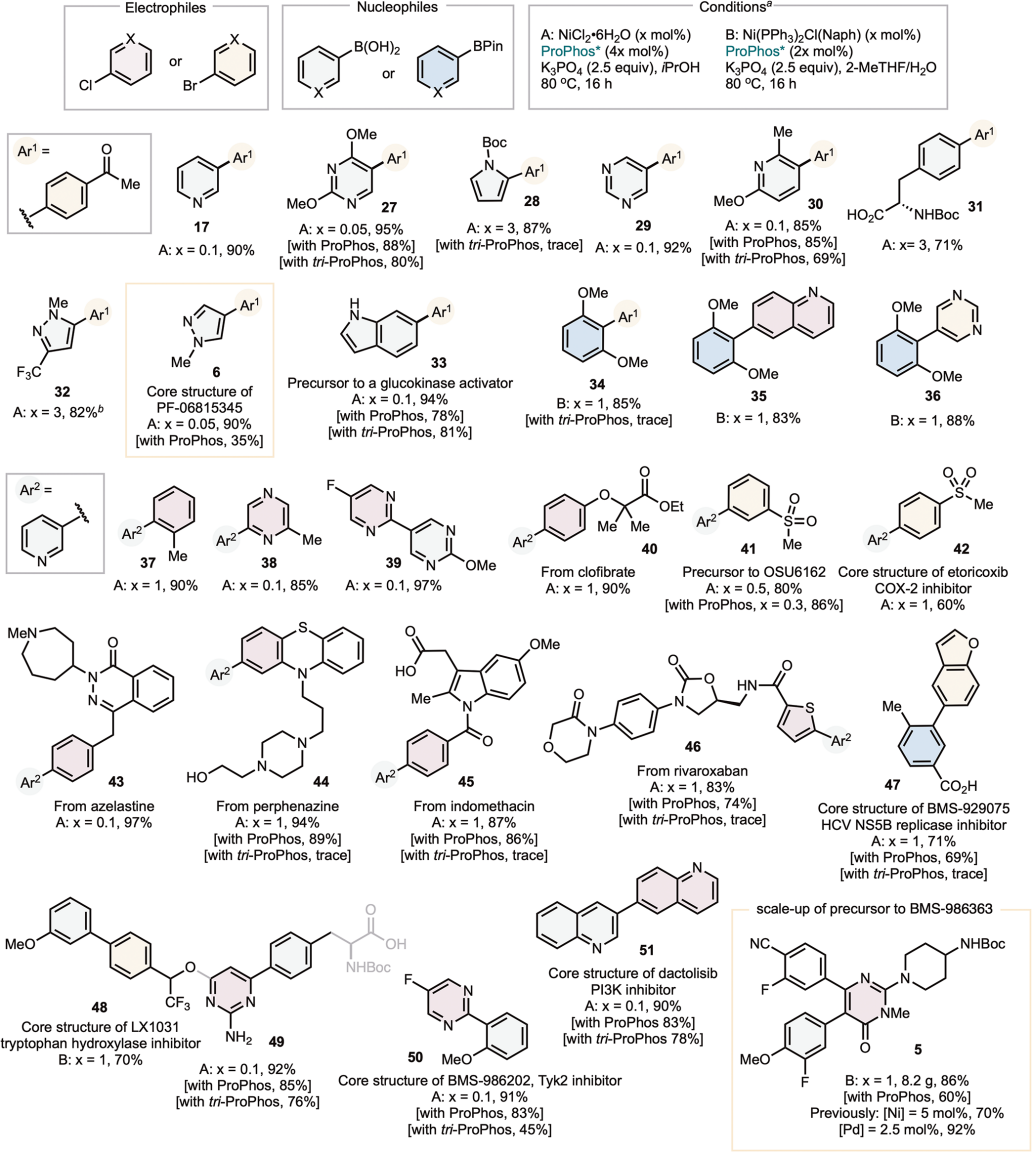
Synthesis of Heteroaromatic Molecules via (ProPhos*)­Ni-SMC

a Isolated yields unless otherwise noted.
b NMR yields with internal standard due to product instability during flash column purification.

(ProPhos*)­Ni-SMC demonstrated compatibility with aryl chlorides as electrophiles, which are typically more cost-effective and readily available compared to aryl bromides.^33,34^ The (ProPhos*)­Ni-SMC reactions exhibited broad applicability across diverse heterocycles and functional groups, including pyridines **17**, **37**–**38**, **40**–**46**, pyrimidines **27**, **29**, **39**, **50**, pyrrole **28**, pyrazoles **6**, **32**, indole **33**, quinoline **51**, free acids **31**, **45**, **47**, and free alcohol **48** as well as esters **40** and amides **46**. A common challenge in SMC is the coupling of substrates bearing *ortho-*substituents. (ProPhos*)Ni catalysts proved effective even in these cases, as notably evidenced by the synthesis of **34**–**36**. In general, ProPhos* exhibits complementary reactivity compared to *tri-*ProPhos,^17^ providing good yields for reactions that performed poorly with *tri-*ProPhos (e.g., **28**, **34**, **44**, **45**, **46**, **47**). Compared to ProPhos,^16^ ProPhos* offers a similar scope but improves yields in several cases at low catalyst loadings (e.g., **5**, **6**, **27**, **30**, **33**, **44**, **49**, **50**).

Many of the heteroaromatic Ni-SMC products shown in Scheme 3 contain the core structures of APIs, represent derivatizations of pharmaceutical products, or serve as precursors to commercial drugs. These examples include core structures of PF-06815345 **6**,^21^ etoricoxib **42**,^35^ HCV NS5B replicase inhibitor (BMS-929075) **47**,^36^ tryptophan hydroxylase inhibitor (LX1031) (**48** and **49**),^37^ Tyk2 inhibitor (BMS-986202) **50**,^38^ and PISK inhibitor dactotisib **51**.^39^ We further compared (ProPhos)­Ni-SMC to established palladium-catalyzed processes for synthesizing known intermediates and precursors to APIs. The previously optimized synthesis of **41**, en route to a dopamine stabilizer OSU6162, employed Pd­(PPh_3_)_4_, an undesired catalyst due to its instability. Additionally, this protocol required diethylborylpyridine as the nucleophile, increasing the cost and air-sensitivity of the process.^40^ In contrast, both ProPhos and ProPhos* Ni-SMC achieved efficient conversion of 3-pyridinylboronic acid **16** to **41** in high yields with 0.3 and 0.5 mol % catalyst loadings, respectively. Furthermore, in the process lab at BMS, we successfully scaled up the synthesis of **5** to 8 g with 1 mol % nickel and 2 mol % ProPhos*. In comparison, the previous synthesis of **5** required 5 mol % nickel^28^ with a lower yield or 2.5 mol % palladium.^20^

We propose that (ProPhos/ProPhos*)­Ni-SMC follows a similar mechanism in *i-*PrOH as that reported with *tri-*ProPhos.^17^ Fast oxidative addition of aryl halides to **52** forms intermediate **53**, likely as the catalyst resting state (Scheme 4). In the presence of a base, the tethered hydroxyl group of ProPhos/ProPhos* displaces the halide with concomitant deprotonation, forming the chelating intermediate **54**. The subsequent transmetalation and formation of **56** from **54** is rapid, facilitated by the coordination of ProPhos to **55**. The following steps, reductive elimination and hydrolysis of the boronic acid on ProPhos, are all expected to be fast.

**Scheme 4:**
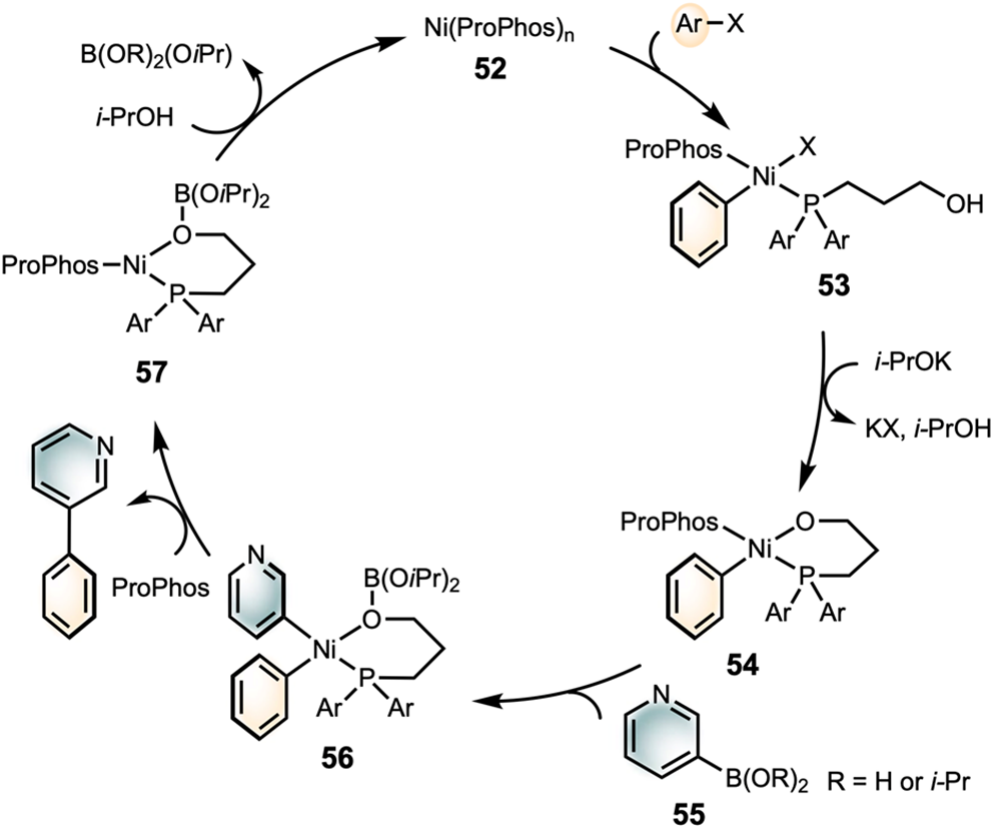
Proposed Catalytic Cycle

## Conclusion

We conducted a comprehensive study on the ligand effects of various ProPhos derivatives, following the design principle of incorporating a tethered hydroxyl group to facilitate transmetalation—either by colocalizing the boronic acid or ester nucleophile or by displacing the halide on nickel to generate a Ni–alkoxy species. Our data show that the ProPhos ligand family, featuring a tethered hydroxyl group, generally outperforms prior monodentate and bidentate phosphine ligands across a broad range of heterocyclic substrates in terms of scope, yield, and reaction kinetics. Among various nucleophilic tethers, the hydroxyl group proved the most effective, with a C3 linkage between the phosphine and hydroxyl delivering the highest reactivity. Substituents on the phosphine also have a significant impact: while sterically bulky aryl groups (e.g., *o*-Tol) or alkyl groups (e.g., Cy) inhibit the reaction, electron-rich substituents enhance catalyst stability. In particular, ProPhos* demonstrates robust activity at catalyst loadings below 0.05 mol % and improves yields by 10–50% compared to ProPhos.

The optimized conditions utilize the cost-effective and air-stable Ni­(II) precatalyst NiCl_2_·6H_2_O, enabling efficient synthesis of core structures and intermediates for APIs bearing diverse heterocycles and *ortho-*disubstituted motifs at catalyst loadings ranging from 0.05 to 1 mol %. Notably, the synthesis of the final intermediate en route to LSD1 inhibitor BMS-986363 under our conditions outperforms previously reported methods, achieving higher yields at lower catalyst loadings, and has been validated on a decagram scale. When comparing ProPhos and ProPhos*, both exhibit similarly broad substrate scopes; however, ProPhos* provides superior catalyst stability at low loadings (Scheme 5). Meanwhile, *tri*-ProPhos offers complementary reactivity, particularly in polar solvents and with hydrophilic substrates.

**Scheme 5:**
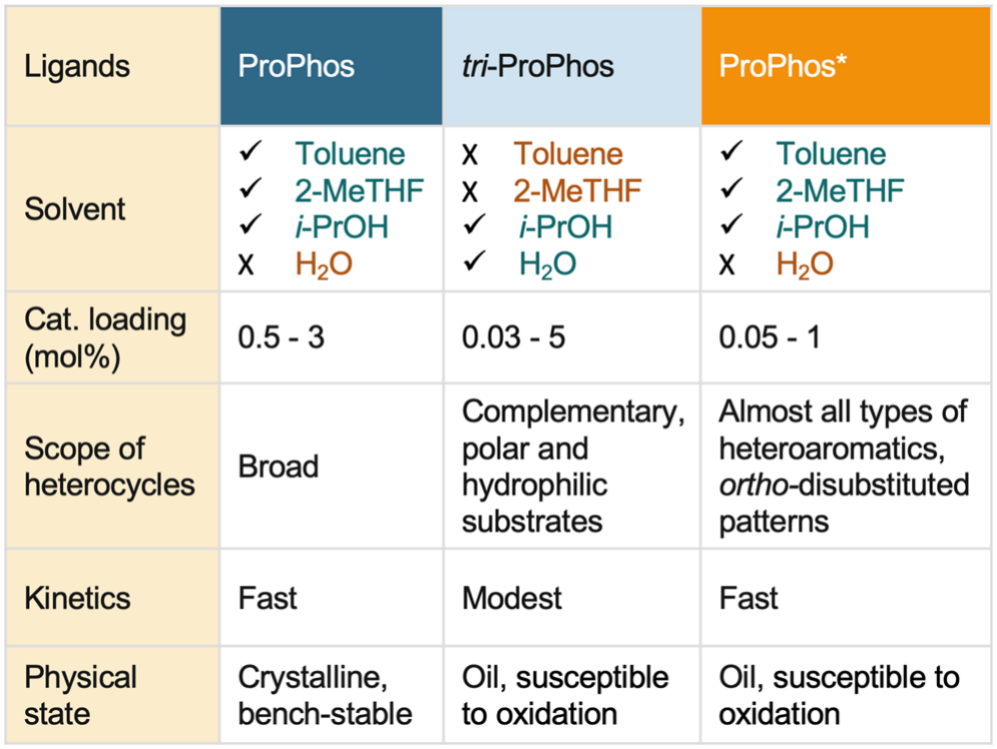
Comparison of ProPhos, *tri-*ProPhos, and ProPhos*

Overall, the ProPhos ligand family addresses key limitations in base-metal catalysis such as high catalyst loadings and restricted functional group tolerance. This work introduces a cost-effective and environmentally friendly alternative for API synthesis and offers a platform to enable the routine implementation of base-metal catalysis in large-scale pharmaceutical manufacturing.

## Supplementary Material



## Notes

Detailed experimental procedures and characterization data for all newly synthesized compounds are provided (PDF)
