# Supplementary material for: Electronic and Steric Effects of the ProPhos Ligand Family for Nickel-Catalyzed Suzuki–Miyaura Coupling of Heteroaromatics
Source: ACS Catal. 2025 Dec 29;16(2):1565–73. doi: 10.1021/acscatal.5c07745 (PMC12813972; doi:10.1021/acscatal.5c07745)
Supplement: Supplementary file 1 [file cs5c07745_si_001.pdf]

Supporting information

Electronic and Steric Effects of the ProPhos Ligand Family for Nickel-Catalyzed Suzuki-Miyaura Coupling of Heteroaromatics

Jin Yang,[1,3] Hengyuan Zhao,[1] Johnathan E. Schultz,[2] Steven R. Wisniewski,[2] Eric M. Simmons[2] and Tianning Diao[1]*

[1]Department of Chemistry, New York University, 100 Washington Square East, New York, NY 10003, United States

[2]Chemical Process Development, Bristol Myers Squibb Company, New Brunswick, NJ 08903, United States

[3]Department of Chemistry, Yeshiva University, 2495 Amsterdam Avenue, New York, NY 10033, United States

*E-Mail: diao@nyu.edu

Table of Contents

1. Materials and Methods 3

2. Synthesis of Phosphine Ligands and Ni Catalysts 4

3. Phosphine Ligand Screenings on Ni-SMC 13

4. Reactivity Studies of (ProPhos)Ni catalyst in *i*PrOH 26

5. Substrate Scope using (ProPhos)Ni Catalysts 32

6. NMR Spectra 53

7. References 132

1. Materials and Methods

All air- and moisture-sensitive reactions and manipulations were performed under nitrogen in a glovebox or using conventional Schlenk techniques. Common solvents (e.g., THF) were dried and deoxygenated by passing through alumina in a solvent purification system. Deuterated solvents were purchased from commercial sources (Cambridge Isotope Laboratories, Inc. and Millipore Sigma). Deuterated solvents used in the glovebox (e.g., C_6D_6 and *i*PrOD-d_8) were degassed using three freeze-pump-thaw cycles and stored over activated molecular sieve for 3 days before use. Unless otherwise specified, reagents and phosphine ligands were purchased from commercial sources (*vide infra*). The nickel precatalysts, $NiCl_2$•$6H_2O$ and $Ni(PPh_3)_2Cl$(Naph), were purchased from commercial sources. Phosphine ligands and other organometallic compounds were obtained from commercial suppliers or prepared according to the methods described in Sections 2.

NMR spectra were recorded on a Bruker Avance 400 spectrometer (400.30 MHz for ^1H, 376.46 MHz for ^{19}F, 162.04 MHz for ^{31}P and 100.67 MHz for ^{13}C), and a Bruker Avance 500 spectrometer (500.20 MHz for ^1H, 470.61 MHz for ^{19}F, 202.49 MHz for ^{31}P, and 125.79 MHz for ^{13}C). Unless otherwise noted, chemical shifts were reported in ppm at room temperature. ^1H chemical shifts were referenced to residual proteo-solvent peak at 7.26 ppm ($CHCl_3$), 7.16 ppm (C_6D_5H), 4.79 ppm (DHO), 3.88 ppm (CF_3CHDOD), 3.31 ppm (CD_2HOD), 2.50 ppm ($(CD_2H)S(O)(CD_3)$), 2.08 ppm (C_7D_7H) and 1.00 ppm (C_3D_7HOD); ^{13}C chemical shifts were referenced to 128.1 ppm (C_6D_6), 77.1 ppm ($CDCl_3$), 62.9 ppm (C_3D_7OD), 61.5 ppm (CF_3CD_2OD), 49.0 ppm (CD_3OD), 29.8 ppm ($(CD_3)_2SO$) and 20.4 ppm (C_7D_8). ^1H and ^{13}C chemical shifts were reported relative to tetramethylsilane (TMS), and ^{31}P chemical shifts were reported relative to 85% H_3PO_4 (aq).

High resolution mass spectra (HRMS) were recorded on an Agilent 6224 TOF LC/MS (APCI source). GCMS data were obtained using a Shimadzu GC-2010 with a Shimadzu SH-Rxi-5Sil MS column. GC data for kinetic experiments and %yield determination were obtained using a Shimadzu GC-2010 Plus with a Restek Rxi-5MS column (L 15 m, ID 0.25, DF 0.25). UltiMate 3000 UHPLC system equipped with Luna 10u C18(2) 100A, AXIA column (250 x 21.2 mm) was used for some sample purifications.

2. Synthesis of Phosphine Ligands and Ni Catalysts

All ProPhos ligands, phosphinyl alcohols, and ProPhos derivatives not listed below were prepared following previously reported procedures.[1] Ni(ProPhos)$_2$Cl(*o*-Tol) **23** were synthesized by using previously reported procedures.[1] Complex Ni(TMEDA)Cl(*o*-Tol)[2] was prepared using a slight modified method and used for ligand substitution without full purification.

2.1 Synthesis of P(*p*-Tol)$_2$CH$_2$CH$_2$CH$_2$OH (ProPhos*, 7)

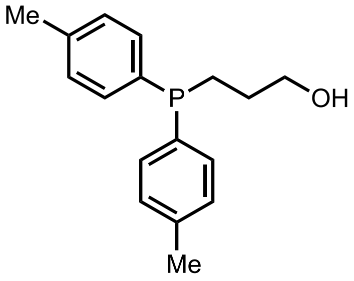

7

3-(Di-p-Tolylphosphino)-1-propanol. To a solution of di-*p*-tolylphosphine (0.700 g, 3.27 mmol, 1.0 equiv) and 3-chloropropanol (0.273 mL, 3.27 mmol, 1.0 equiv) in THF (20 mL), *n*-butyl lithium (3.3 mL, 2.20 M in hexanes, 7.26 mmol, 2.2 equiv) was added dropwise at 0 °C. The mixture was stirred for 1 h at room temperature. Afterward, deoxygenated water (10 mL) was added, and the mixture was evaporated. The residue was then extracted with CH_2Cl_2 (2 x 10 mL). The resulting suspension was filtered, and the solution was evaporated. The remaining residue was purified further by column chromatography using a solvent system of hexane/ CH_2Cl_2 (1:1) initially, followed by CH_2Cl_2/EtOAc (7:3), yielding the product (0.342 g, 1.26 mmol, 38%) as a colorless oil.

^{1}H NMR (500.20 MHz, CDCl$_3$) δ: 7.32 (t, *J* = 7.7 Hz, 4H, H$_o$), 7.14 (d, *J* = 7.6 Hz, 4H, H$_m$), 3.70 (td, *J* = 6.4, 1.6 Hz, 2H, OCH$_2$), 2.34 (s, 6H, CH$_3$), 2.08 (ddd, *J* = 7.9, 6.4, 1.7 Hz, 2H, PCH$_2$), 1.73 – 1.64 (m, 2H, CH$_2$), 1.42 (s, 1H, OH).

^{31}P{^{1}H} NMR (202.47 MHz, CDCl$_3$) δ: -18.3 (s).

^{13}C{^{1}H} NMR (125.79 MHz, CDCl$_3$) δ: 138.7 (s, C$_p$), 135.3 (d, *J* = 11 Hz, C$_{ipso}$), 132.8 (d, *J* = 19 Hz, C$_o$), 129.4 (d, *J* = 7 Hz, C$_m$), 63.8 (d, *J* = 14 Hz, OCH$_2$), 29.3 (d, *J* = 15 Hz, PCH$_2$), 24.6 (d, *J* = 11 Hz, CH$_2$), 21.4 (s, CH$_3$).

HRMS (ESI-TOF, CH_3OH) m/z: $[M + Na]^+$ calculated for $C_{17}H_{21}PONa$ 295.1222, found 295.1223.

2.2 NMR characterization of $PCy_2CH_2CH_2CH_2OH$ (^{Cy}ProPhos, 8)

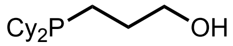

8

3-(Dicyclohexylphosphino)-1-propanol (CAS: 2360-11-4). The Cy-ProPhos was purchased from the commercial source (Sinocompound). To verify the actual purity and ensure the correct stoichiometry for catalytic reactions, NMR characterization was performed. The purity of this batch of compound was estimated to be >95% based on the $^{31}P\{^1H\}$ NMR analysis.

^1H NMR (500.20 MHz, C_6D_6) δ: 3.50 (t, *J* = 6.2 Hz, 2H, CH_2O), 1.85 – 1.80 (m, 2H), 1.74 – 1.60 (m, 10H), 1.51 – 1.45 (m, 2H), 1.39 – 1.34 (m, 2H), 1.24 – 1.13 (m, 10H).

$^{31}P\{^1H\}$ NMR (202.47 MHz, C_6D_6) δ: -5.8 (s).

$^{13}C\{^1H\}$ NMR (125.79 MHz, C_6D_6) δ: 63.8 (d, *J* = 12 Hz, CH_2O), 33.9 (d, *J* = 14 Hz, CH), 32.0 (d, *J* = 18 Hz, CH_2), 30.8 (d, *J* = 15 Hz, CH_2), 29.4 (d, *J* = 8 Hz, CH_2), 27.7 (d, *J* = 12 Hz, CH_2), 27.6 (d, *J* = 8 Hz, CH_2), 26.9 (s, CH_2), 18.2 (d, *J* = 18 Hz, CH_2).

HRMS (ESI-TOF, CH_3OH) m/z: $[M + H]^+$ calculated for $C_{15}H_{30}PO$ 257.2029, found 257.2028.

2.3 Synthesis of $P(3,5\text{-di-Me-Ph})_2CH_2CH_2CH_2OH$ ($^{di\text{-}Me}$ProPhos, 9)

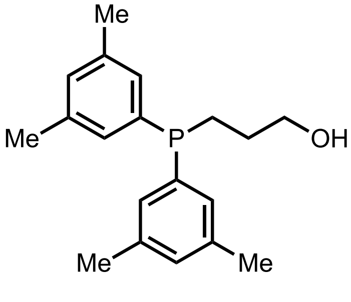

9

3-(bis(3,5-Dimethylphenyl)phosphaneyl)propan-1-ol. Followed by the procedure of synthesizing ProPhos* **7**, bis(3,5-dimethylphenyl)phosphane (1.00 g, 4.13 mmol, 1.1 equiv), 3-chloropropanol (0.314 mL, 3.75 mmol, 1.0 equiv) and *n*-butyl lithium (3.75 mL, 2.00 M in hexanes, 7.50 mmol, 2.0 equiv) were used. The product (0.635 g, 2.11 mmol, 56%) was obtained as a colorless oil.

^{1}H NMR (500.20 MHz, CD_3Cl) δ: 7.05 (dd, *J* = 7.8, 1.6 Hz, 4H, H_o), 6.95 (s, 2H, H_p), 3.71 (t, *J* = 6.4 Hz, 2H, CH_2OH), 2.29 (s, 12H, Me), 2.12 – 2.05 (m, 2H, PCH_2), 1.82 (br s, 1H, OH), 1.74 – 1.63 (m, 2H, CH_2).

$^{31}P\{^{1}H\}$ NMR (202.47 MHz, CD_3Cl) δ: -16.5 (s).

$^{13}C\{^{1}H\}$ NMR (125.79 MHz, CD_3Cl) δ: 138.4 (d, *J* = 12 Hz, C_{ipso}), 137.9 (d, *J* = 7 Hz, C_m), 130.6 (d, *J* = 6 Hz, C_o), 130.4 (s, C_p), 63.8 (d, *J* = 14 Hz, CH_2OH), 29.3 (d, *J* = 16 Hz, PCH_2), 24.3 (d, *J* = 11 Hz, CH_2), 21.4 (s, Me).

HRMS (ESI-TOF, CH_3CN) m/z: $[M + Na]^+$ calculated for $C_{19}H_{25}PONa$ 323.1535, found 323.1534.

2.4 Synthesis of P(*o*-Tol)$_2$CH$_2$CH$_2$CH$_2$OH ($^{o\text{-}Me}$ProPhos)

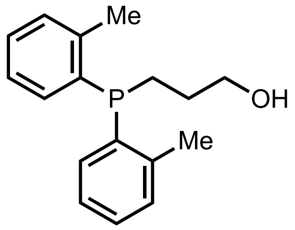

10

3-(di-o-Tolylphosphaneyl)propan-1-ol. Followed by the procedure of synthesizing ProPhos* **7**, di-o-tolylphosphane (1.00 g, 4.67 mmol, 1.1 equiv), 3-chloropropanol (0.355 mL, 4.24 mmol, 1.0 equiv) and *n*-butyl lithium (3.73 mL, 2.50 M in hexanes, 9.33 mmol, 2.2 equiv) were used. The product (0.754 g, 2.77 mmol, 65%) was obtained as a white solid.

^{1}H NMR (500.20 MHz, CD_3Cl) δ: 7.25 – 7.19 (m, 4H, Ar), 7.16 (q, *J* = 7.3, 5.6 Hz, 4H, Ar), 3.72 (t, *J* = 6.4 Hz, 2H, CH_2OH), 2.43 (s, 6H, *o*-Me), 2.09 – 2.00 (m, 2H, PCH_2), 1.78 – 1.66 (m, 2H, CH_2), 1.62 – 1.52 (m, 1H, OH).

$^{31}P\{^{1}H\}$ NMR (202.47 MHz, CD_3Cl) δ: -38.1 (s).

$^{13}C\{^1H\}$ NMR (125.79 MHz, CD_3Cl) δ: 142.5 (d, J = 25 Hz), 136.9 (d, J = 13 Hz), 131.1 (s), 130.2 (d, J = 5 Hz), 128.6 (s), 126.2 (s), 63.9 (d, J = 14 Hz, CH_2OH), 29.2 (d, J = 16 Hz, PCH_2), 23.4 (d, J = 12 Hz, CH_2), 21.4 (d, J = 21 Hz, Me).

HRMS (ESI-TOF, CH_3CN) m/z: $[M + Na]^+$ calculated for $C_{17}H_{21}PONa$ 295.1222, found 295.1240.

2.5 Synthesis of $PPh_2CH_2C(Me)_2CH_2OH$ ($^{gem\text{-}Me}$ProPhos, 11)

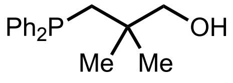

11

3-(Diphenylphosphino)-2,2-dimethyl-1-propanol (CAS: 195450-69-2). To a solution of diphenylphosphine (1.74 mL, 10.0 mmol, 1.0 equiv) and 3-chloro-2,2-dimethylpropanol (1.23 g, 10.0 mmol, 1.0 equiv) in THF (20 mL), *n*-butyl lithium (13.6 mL, 2.20 M in hexanes, 30.0 mmol, 3.0 equiv) was added dropwise at 0 °C. The yellow reaction mixture was heated to reflux for 18 h, during which the color gradually changed from orange to dark red. The reaction was quenched with deoxygenated water (20 mL), and the solvents were removed under vacuum. The combined organic layers were concentrated and the residue was further purified via column chromatography (hexane:CH_2Cl_2 1:1→CH_2Cl_2:EtOAc 7:3) to yield a viscous oil. This oil was dissolved in a small amount of THF, layered with Et_2O, resulting in an off-white crystal (1.54 g, 5.65 mmol, 56%). The synthesis was slightly modified according to the establish literature procedure, and the NMR data matched with the reported values. [3]

^1H NMR (400.30 MHz, C_6D_6) δ: 7.49 (ddt, J = 8.8, 5.6, 1.6 Hz, 4H, H_o), 7.11 – 6.99 (m, 6H, H_m&H_p), 3.22 (s, 2H, OCH_2), 2.16 (d, J = 3.4 Hz, 2H, PCH_2), 0.95 (s, 6H, CH_3), 0.85 (s, 1H, OH).

$^{31}P\{^1H\}$ NMR (162.04 MHz, C_6D_6) δ: -24.7 (s).

$^{13}C\{^1H\}$ NMR (100.67 MHz, C_6D_6) δ: 140.8 (d, J = 13 Hz, C_{ipso}), 133.4 (d, J = 20 Hz, C_o), 128.7 (d, J = 7 Hz, C_m), 128.6 (s), 72.1 (d, J = 9 Hz, CH_2O), 39.5 (d, J = 17 Hz, PCH_2), 36.4 (d, J = 13 Hz, $C(Me)_2$), 25.96 (d, J = 9 Hz, CH_3).

HRMS (ESI-TOF, CH_3CN) m/z: $[M + H]^+$ calculated for $C_{17}H_{22}PO$ 273.1403, found 273.1366.

2.6 Synthesis of PPh_2CH_2OH (12)

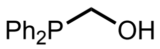

12

1-(Diphenylphosphino)-1-methanol (CAS: 5958-44-1). Diphenylphosphine (1.87 mL, 10.7 mmol, 1.0 equiv) and paraformaldehyde (0.323 g, 10.7 mmol, 1.0 equiv) were heated to 110°C with stirring for 30 min until the paraformaldehyde completely dissolved, forming a clear liquid. The mixture was then allowed to cool to room temperature and stored at -35 °C for 2 days. Crystals is formed in the colorless oil (2.30 g, 10.6 mmol, 99%). The synthesis was carried out according to the established literature procedure, and the NMR data matched with the reported values.[4]

Note: The thermal hydrophosphination reaction produces P-capped oligomers, such as $PPh_2(CH_2O)_n$-H, as byproducts. Due to the thermal instability of PPh_2CH_2OH,[4, 5] distillation cannot be used for further purification. Its purity, determined to be 91% by $^{31}P\{^1H\}$ NMR analysis, is considered adequate for use in catalytic reactions.

^{1}H NMR (400.30 MHz, $CDCl_3$) δ: 7.53 – 7.45 (m, 4H, H_o), 7.37 (dtd, *J* = 6.1, 3.6, 1.5 Hz, 6H, H_m&H_p), 4.46 – 4.38 (m, 2H, CH_2), 1.51 (h, *J* = 3.1 Hz, 1H, OH).

$^{31}P\{^1H\}$ NMR (162.04 MHz, $CDCl_3$) δ: -9.8 (s).

$^{13}C\{^1H\}$ NMR (100.67 MHz, $CDCl_3$) δ: 135.4 (d, *J* = 12 Hz, C_{ipso}), 133.3 (d, *J* = 18 Hz, C_o), 129.1 (s, C_p), 128.8 (d, *J* = 7 Hz, C_m), 62.7 (d, *J* = 16 Hz, CH_2).

LCMS (ESI-TOF, CH_3OH) m/z: $[M + H]^+$ calculated for $C_{13}H_{14}OP$ 217.08, found 217.08.

2.7 Synthesis of $PPh_2CH_2CH_2OCH_2CH_2OH$ (Peg-ProPhos)

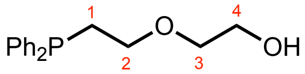

(Peg-ProPhos)

2-[2-(Diphenylphosphino)ethoxy]ethanol (CAS: 144934-94-1). To a solution of diphenylphosphine (1.03 mL, 5.91 mmol, 1.0 equiv) and 2-(2-chloroethoxy)ethan-1-ol (0.630 mL, 5.91 mmol, 1.0 equiv) in THF (20 mL), *n*-butyl lithium (5.90 mL, 2.20 M in hexanes, 13.0 mmol, 2.2 equiv) was added dropwise at 0 °C. The mixture was stirred at room temperature for 3 h. Deoxygenate Water (10 mL) was then added, and the mixture was evaporated. The residue was extracted with CH_2Cl_2 (2 x 10 mL). The organic layers were combined and evaporated, and the remaining residue was

purified by column chromatography using a solvent gradient of hexane/CH_2Cl_2 (1:1) to CH_2Cl_2/EtOAc (7:3), yielding the product as a yellow oil (0.753 g, 2.75 mmol, 47%). Note: This compound was previously isolated as a byproduct from another mixture.[1] The chemical shifts observed in this synthesis are slightly different, likely due to the excess H_2O present in the previously isolated compound, which causes hydrogen bonding.

^{1}H NMR (400.30 MHz, $CDCl_3$) δ: 7.44 (ddt, *J* = 7.5, 5.2, 2.6 Hz, 4H, H_o), 7.37 – 7.30 (m, 6H, H_m&H_p), 3.69 – 3.60 (m, 4H, H2&H3), 3.53 – 3.47 (m, 2H, H4), 2.41 (t, *J* = 7.3 Hz, 2H, H1), 1.93 (s, 1H, OH).

^{31}P{^{1}H} NMR (162.04 MHz, $CDCl_3$) δ: -23.6 (s).

^{13}C{^{1}H} NMR (100.67 MHz, $CDCl_3$) δ: 138.4 (d, *J* = 12 Hz, C_{ipso}), 132.8 (d, *J* = 19 Hz, C_o), 128.9 (s, C_p), 128.6 (d, *J* = 7 Hz, C_m), 71.9 (s, C3), 68.6 (d, *J* = 22 Hz, C2), 61.9 (s, C4), 29.1 (d, *J* = 13 Hz, C1).

LCMS (ESI-TOF, CH_3OH) m/z: $[M + H]^+$ calcd for $C_{16}H_{20}O_2P$ 275.12, found 275.11.

2.8 Attempted synthesis of P(4-MeOPh)$_2$CH$_2$CH$_2$CH$_2$OH

Method A: Following the general procedure in Section 2.1, analysis of a reaction aliquot by ^{31}P{^{1}H} NMR indicated that the predominant species was the diphosphine (4-MeOPh)$_4$P$_2$, with only trace amounts of the oxidized phosphine (Figure S1a).

Method B: Using the procedure described in Section 2.5, the reaction mixture was refluxed under N_2 for 16 h. ^{31}P{^{1}H} NMR analysis again showed that the diphosphine remained the major product, accompanied by a slightly increased amount of the oxidized phosphine (Figure S1b). The reaction mixture was purified by column chromatography to afford (4-MeOPh)$_4$P$_2$. The ^{1}H and ^{31}P{^{1}H} NMR spectra (Figure S2) of the isolated compound match those reported in the literature.

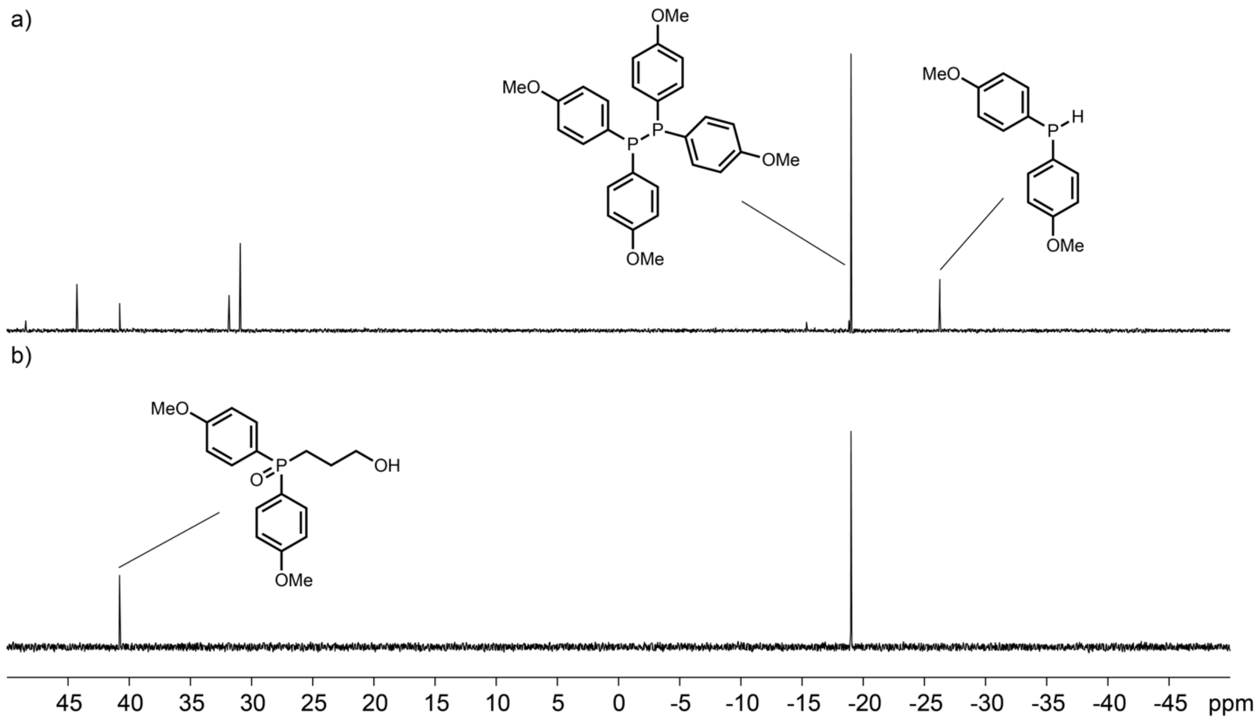

Figure S1. $^{31}P\{^{1}H\}$ NMR spectra of the crude synthesized by Method A at room temperature (a) and Method B after reflux for 16 h (b). Some unidentified species was also observed in (a).

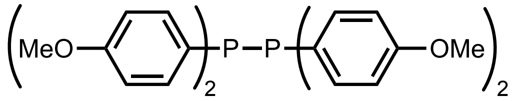

^{1}H NMR (400.30 MHz, $CDCl_3$) δ: 7.36 – 7.30 (m, 8H), 6.90 – 6.85 (m, 8H), 3.80 (s, 12H, MeO).

$^{31}P\{^{1}H\}$ NMR (162.04 MHz, $CDCl_3$) δ: -19.0 (s).

Metal phosphido species are known to produce this diphosphine.[6] We proposed that the MeO substituent could facilitate P–P bond formation rather than promoting nucleophilic attack on the alkyl chloride.

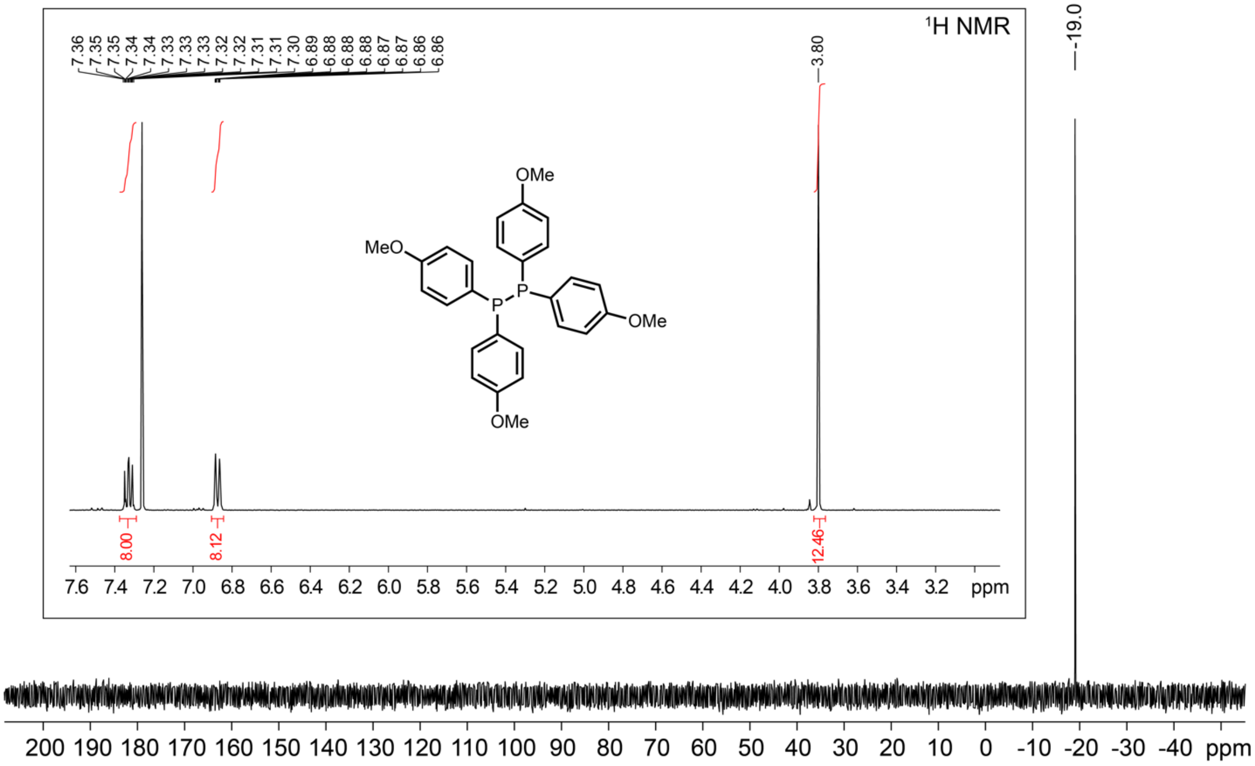

Figure S2. $^{31}P\{^1H\}$ NMR and ^{1}H NMR (inset) spectra of isolated diphosphine$(4\text{-}MeOPh)_4P_2$.

2.9 Synthesis of Ni(ProPhos*)$_2$Cl(*o*-Tol) (25)

A vial was charged with Ni(TMEDA)Cl(*o*-Tol) (100 mg, 0.332 mmol, 1.0 equiv), ProPhos* (190 mg, 0.697 mmol, 2.1 equiv) and THF (4 mL). The mixture was stirred at room temperature overnight, and the solvent was subsequently removed under vacuum to yield an orange oil. THF (2 mL) was added to the oil, and the suspension was filtered through Celite. The Celite was washed with THF (3 x 2 mL). The resulting orange filtrate was concentrated under vacuum, layered with pentane (4 mL), and stored at -35 °C overnight, yielding an oil. The oil was triturated with pentane and further dried under vacuum for 4 h to afford a yellow solid (188 mg, 0.257 mmol, 77% yield).

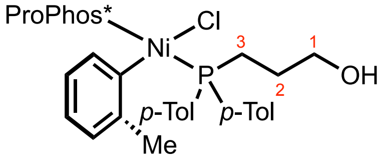

25

^{1}H NMR (500.20 MHz, *i*PrOD-*d*$_8$) δ: 7.45 (dt, *J* = 7.9, 4.9 Hz, 4H, H$_o$ at *p*-Tol), 7.08 (dt, *J* = 8.6, 4.5 Hz, 4H, H$_o$ at *p*-Tol), 6.95 (dd, *J* = 10.2, 7.8 Hz, 8H, H$_m$ at *p*-Tol), 6.49 – 6.44 (m, 1H, *o*-Tol),

6.38 (td, J = 7.3, 1.4 Hz, 1H, *o*-Tol), 6.30 (td, J = 7.4, 1.6 Hz, 1H, *o*-Tol), 6.19 (dd, J = 7.5, 1.6 Hz, 1H, *o*-Tol), 3.09 (qt, J = 10.6, 6.5 Hz, 4H, H1), 2.46 (s, CH_3 at *o*-Tol) 2.24 (s, 6H, CH_3 at *p*-Tol), 2.22 (s, 6H, CH_3 at *p*-Tol), 1.65 – 1.49 (m, 4H, H3), 1.31 – 1.22 (m, 2H, H2, overlapped with some residual pentane), 1.19 – 1.10 (m, 2H, H2, overlapped with some residual pentane). The OH signal is not observed in *i*PrOD-d_8, presumably due to the fast H/D exchange.

$^{31}P\{^1H\}$ NMR (202.47 MHz, *i*PrOD-d_8) δ: 13.6 (s).

$^{13}C\{^1H\}$ NMR (125.79 MHz, *i*PrOD-d_8) δ: 149.1 (s, *o*-Tol), 143.5 (s, *o*-Tol), 139.9 (s, C_p at *p*-Tol), 139.3 (s, C_p at *p*-Tol), 136.8 (s, *o*-Tol), 134.7 (t, J = 6 Hz, C_o at *p*-Tol), 133.5 (t, J = 5 Hz, C_o at *p*-Tol), 129.1 (t, J = 5.0 Hz, C_m at *p*-Tol), 128.9 (t, J = 5 Hz, C_m at *p*-Tol), 128.5 (t, J = 21 Hz, C_{ipso} at *p*-Tol), the other Cipso signal is overlapped with the signals due to C_m at *p*-Tol, 128.1 (s, *o*-Tol), 123.9 (s, *o*-Tol), 122.7 (s, *o*-Tol), 62.5 (t, J = 8 Hz, C1), 27.8 (s, C3), 26.4 (s, CH_3 at *o*-Tol), 23.0 (t, J = 14 Hz, C2), 21.5 (CH_3 at *p*-Tol).

HRMS (ESI-TOF, CH_3CN) m/z: $[M - Cl]^+$ calculated for $C_{41}H_{49}NiO_2P_2$ 693.2556, found 693.2551.

3. Phosphine Ligand Screenings on Ni-SMC

3.1 General procedure for the ligand screening

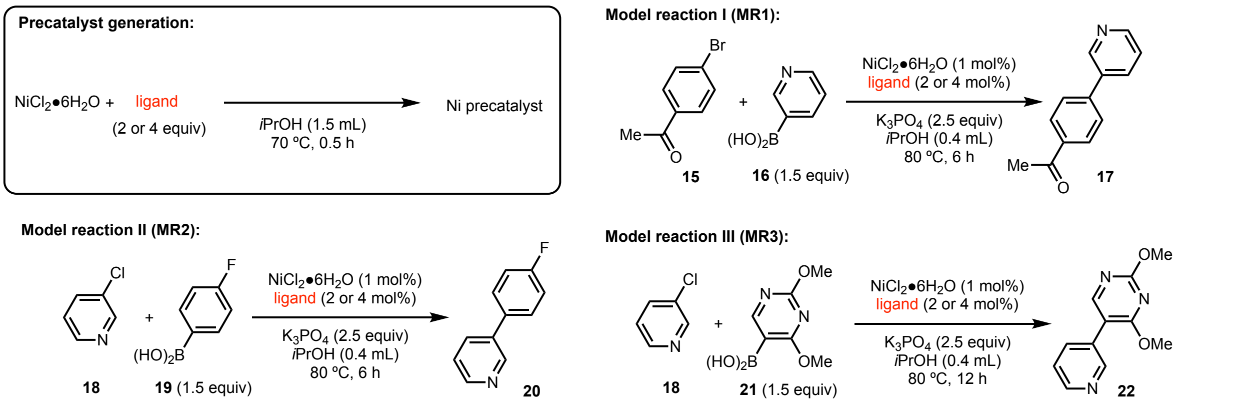

In a nitrogen-filled glove box, $NiCl_2{\bullet}6H_2O$ (7.5 μmol, 1.7 mg, 1.0 equiv) was combined with either monodentate/hemi-labile phosphines (30 μmol, 4.0 equiv) or bidentate phosphines (15 μmol, 2.0 equiv) in a 2 mL glass vial containing isopropanol (*i*PrOH) (1.5 mL). The vial was then removed from the glove box, placed in a shaker, and heated to 70 °C with agitation at 800 rpm for 30 min. The resulting stock solution or suspension was brought back into the glove box for further use. Separately, an aryl halide (**15** or **18**, 0.20 mmol, 1.0 equiv) and a boronic acid (**16**, **19** or **21**, 0.30 mmol, 1.5 equiv) were weighed or measured (for liquid) into a 2 mL glass vial, along with solid K_3PO_4 (2.5 equiv). The precatalyst solution/suspension (0.4 mL) was added to the vial. For suspensions, an aliquot was taken while vigorously shaking the vial to ensure even distribution of the catalyst. The vial was then sealed, removed from the glove box, and placed in a shaker, heated to 80 °C with agitation at 800 rpm for 6 to 12 h. After cooling to room temperature, *n*-decane was added as an internal standard. The samples were quenched with water and extracted with ethyl acetate. The organic layer was combined, and an aliquot was analyzed by GC-FID (Calibration curves for three isolated biaryl products,[7] **17**, **20** and **22**, were created to determine their R_f values for quantitative analysis). The % yields from duplicate runs had an error margin of ±2%. Kinetic plots were prepared using the same procedure.

Scheme S1. Structures of Monodentate and Bidentate Phosphine Ligands.

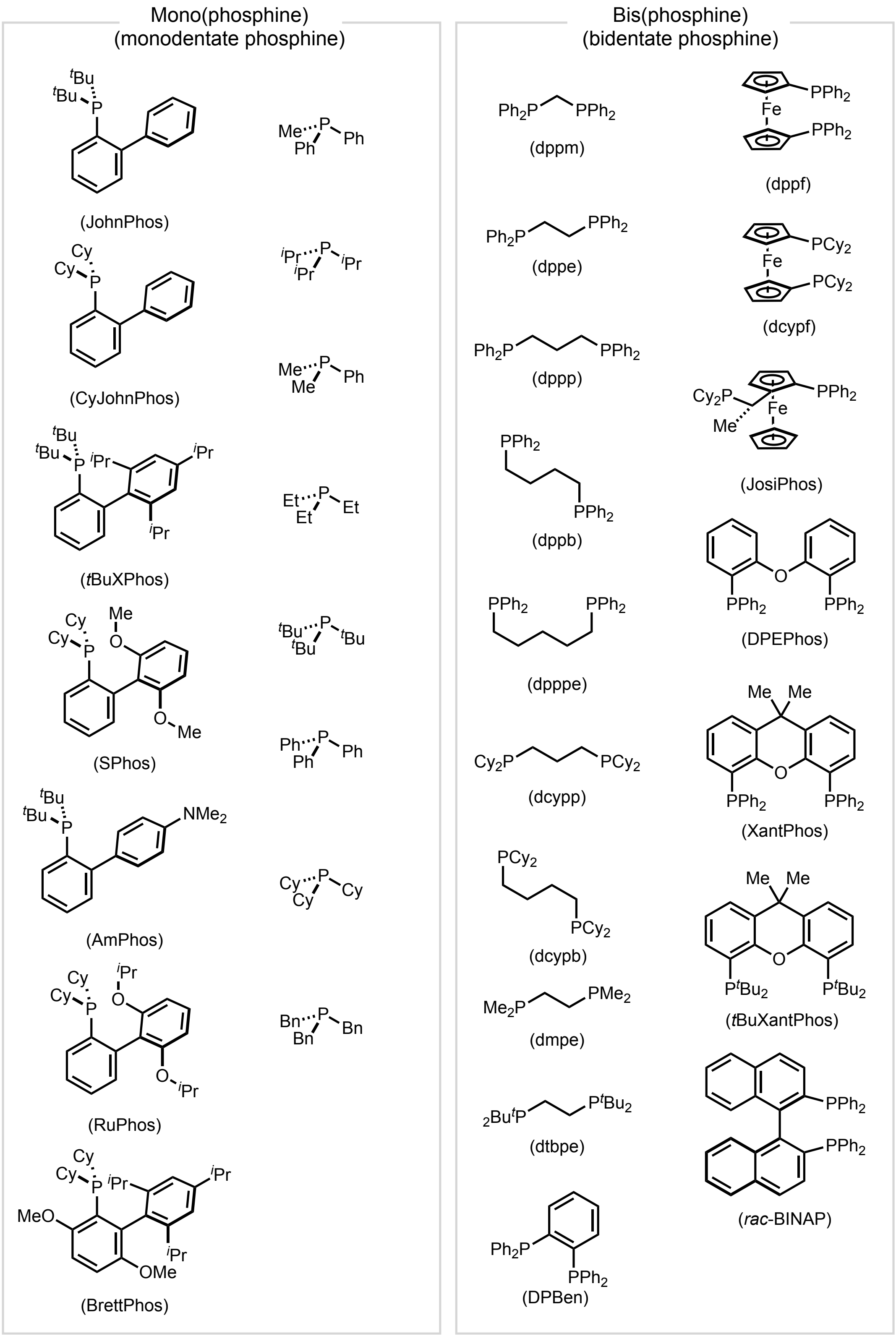

Scheme S2. Structures of Hemi-Labile Phosphine Ligands.

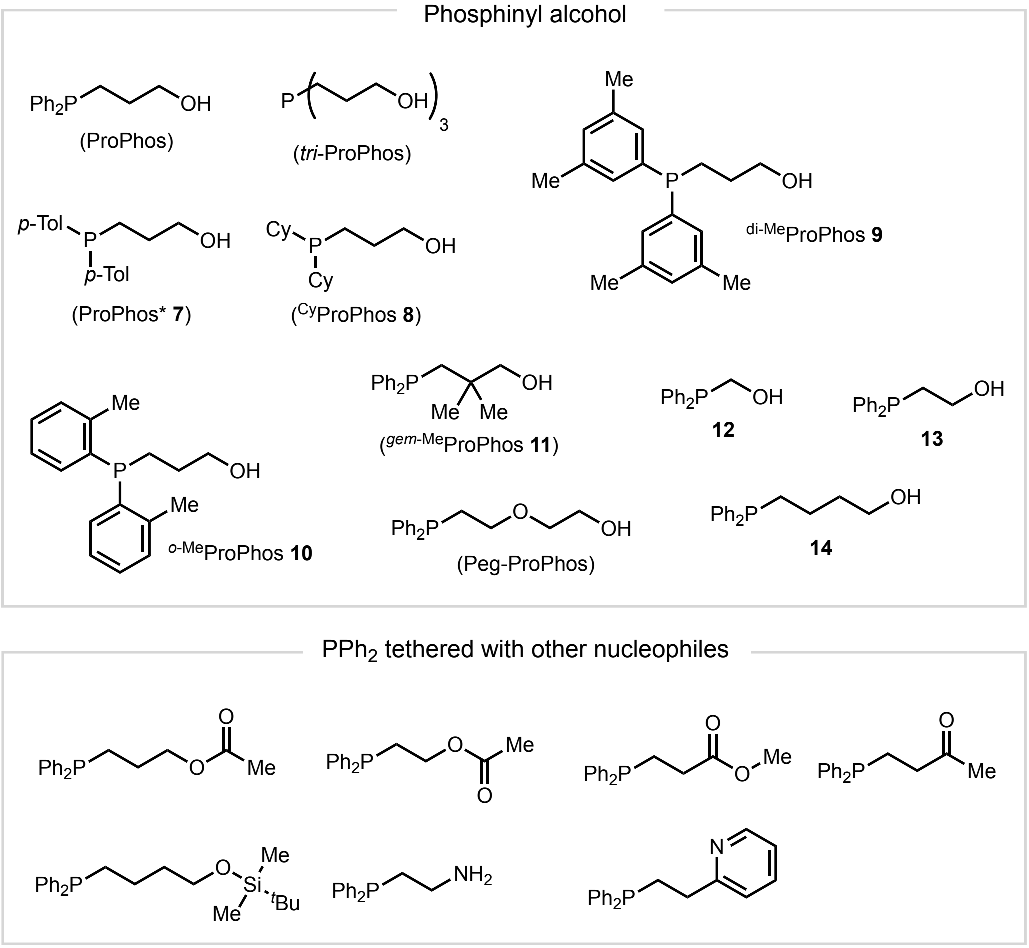

Table S1. Evaluation of Ligands in the Three Model Catalytic Reactions.[a]

Entry	Ligands	Type	%Yield MR1	%Yield MR2	%Yield MR3	Source
**1**	JohnPhos	A	14	56	2	Strem Chemicals
**2**	CyJohnPhos	A	8	2	18	Strem Chemicals
**3**	*t*BuXPhos	A	6	8	3	Sigma
**4**	SPhos	A	7	2	8	Ambeed
**5**	AmPhos/APhos	A	2	2	14	Ambeed
**6**	RuPhos	A	4	3	6	Ambeed
**7**	BrettPhos	A	7	23	2	Ambeed
**8**	PPh_2Me	A	25	37	16	Ambeed
**9**	$P\textit{i}Pr_3$	A	20	26	98	Thermo Fisher
**10**	$PPhMe_2$	A	3	2	1	Sigma
**11**	PEt_3	A	10	4	12	Sigma
**12**	$P\textit{t}Bu_3$	A	3	2	2	Sigma
**13**	PPh_3	A	2	48	67	Sigma
**14**	PCy_3	A	4	17	81	Sigma
**15**	PBn_3	A	22	59	92	Alfa
**16**	Dppm	B	15	0	1	Sigma
**17**	Dppe	B	0	0	0	Sigma
**18**	Dppp	B	0	0	0	Sigma
**19**	Dppb	B	10	17	3	Sigma
**20**	Dpppe	B	2	66	39	Sigma
**21**	Dcypp	B	0	0	0	Ambeed
**22**	Dcypb	B	2	19	80	Ambeed
**23**	dmpe	B	55	4	0	Strem Chemicals
**24**	Dtbpe	B	1	0	0	Ambeed
**25**	Dppf	B	12	45	46	Sigma

**26**	Dcypf	B	2	0	0	Ambeed
**27**	JosiPhos	B	0	0	0	Sigma
**28**	DPEPhos	B	7	26	39	Sigma
**29**	XantPhos	B	0	1	11	Sigma
**30**	*t*BuXantPhos	B	2	12	2	Strem Chemicals
**31**	*rac*-BINAP	B	2	5	1	Sigma
**32**	DPBen	B	0	0	0	Sigma
**33**	PPh_2CH_2OH (**12**)	C	57	62	30	Synthesized
**34**	$PPh_2(CH_2)_2OH$ (**13**)	C	94	98	87	Synthesized
**35**	ProPhos	C	95	98	99	Synthesized
**36**	$PPh_2(CH_2)_4OH$ (**14**)	C	71	89	97	Synthesized
**37**	Peg-ProPhos	C	21	71	86	Synthesized
**38**	$^{gem\text{-}Me}$ProPhos (**11**)	C	98	78	92	Synthesized
**39**	ProPhos* (**7**)	C	98	96	93	Synthesized
	$^{di\text{-}Me}$ProPhos (**9**)	C	95	96	95	Synthesized
	$^{o\text{-}Me}$ProPhos (**10**)	C	52	87	76	Synthesized
**40**	*tri*-ProPhos	C	95	89	96	Sigma
**41**	^{Cy}ProPhos (**8**)	C	97	92	93	Sinocompound
**42**	$PPh_2(CH_2)_2OAc$	D	20	49	99	Synthesized
**43**	$PPh_2(CH_2)_3OAc$	D	93	93	97	Synthesized
**44**	$PPh_2(CH_2)_2CO_2Me$	D	2	4	19	Synthesized
**45**	$PPh_2(CH_2)_2Ac$	D	13	33	92	Synthesized
**46**	$PPh_2(CH_2)_4OTBS$	D	30	83	93	Synthesized
**47**	$PPh_2(CH_2)_2NH_2$	D	14	55	10	Ambeed
**48**	$PPh_2(CH_2)_2$(2-Py)	D	16	73	61	Ambeed
**49**	N/A	control	0	trace	0	

[a] For Type, A = mono(phosphine); B = bis(phosphine); C = Phosphinyl alcohol; D = PPh_2 tethered with other nucleophiles.

3.2 Effect of precatalyst solubility on catalytic activity

Table S2. Solubility and Appearance of $NiCl_2$•$6H_2O$ and Ligands in *i*PrOH.

Entry	Ligands	Appearance	Entry	Ligands	Appearance
1	JohnPhos	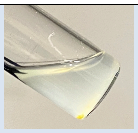 heterogeneous	25	Dppf	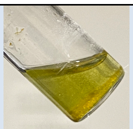 heterogeneous
2	CyJohnPhos	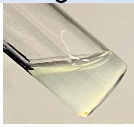 heterogeneous	26	Dcypf	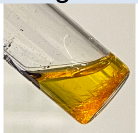 heterogeneous
3	*t*BuXPhos	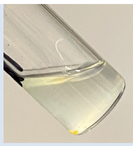 heterogeneous	27	JosiPhos	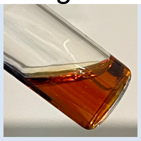 homogeneous
4	SPhos	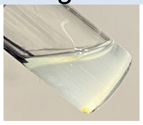 heterogeneous	28	DPEPhos	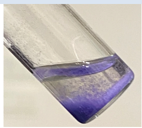 heterogeneous
5	AmPhos /APhos	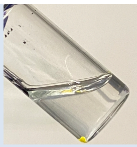 heterogeneous	29	XantPhos	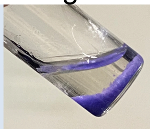 heterogeneous
6	RuPhos	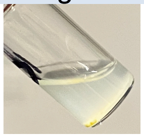 heterogeneous	30	*t*BuXantPhos	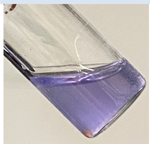 heterogeneous
7	BrettPhos	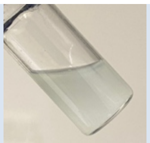 heterogeneous	31	*rac*-BINAP	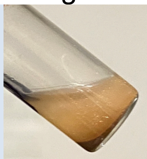 heterogeneous
8	PPh_2Me	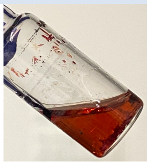 homogeneous*	32	DPBen	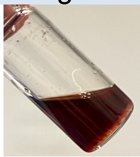 heterogeneous
9	$P\textit{i}Pr_3$	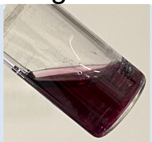 homogeneous	33	PPh_2CH_2OH (**12**)	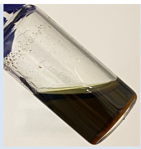 homogeneous

10	$PPhMe_2$	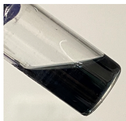 homogeneous
11	PEt_3	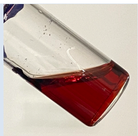 homogeneous
12	P*t*Bu_3	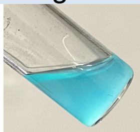 homogeneous
13	PPh_3	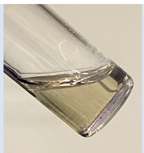 heterogeneous
14	PCy_3	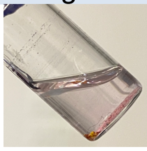 heterogeneous
15	PBn_3	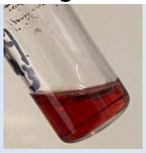 homogeneous
16	Dppm	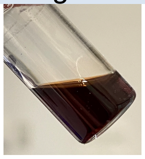 homogeneous
17	Dppe	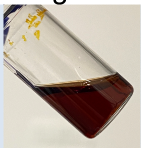 homogeneous
18	Dppp	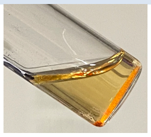 heterogeneous
34	$PPh_2(CH_2)_2OH$ (**13**)	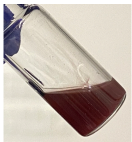 homogeneous
35	ProPhos	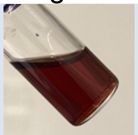 homogeneous
36	$PPh_2(CH_2)_4OH$ (**14**)	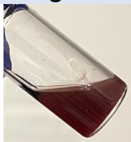 homogeneous
37	Peg-ProPhos	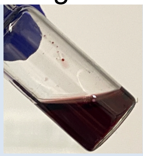 homogeneous
38	$^{gem\text{-}Me}$ProPhos (**11**)	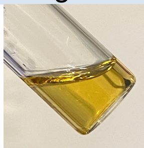 homogeneous
39	e.g. ProPhos* (**7**)	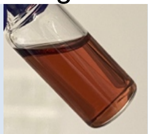 homogeneous
40	*tri*-ProPhos	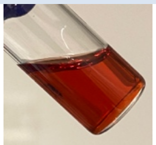 homogeneous
41	^{Cy}ProPhos (**8**)	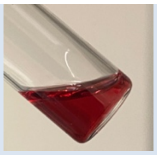 homogeneous
42	$PPh_2(CH_2)_2OAc$	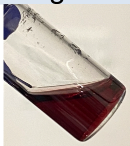 homogeneous

19	Dppb	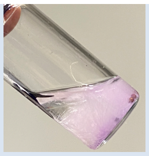 heterogeneous	43	$PPh_2(CH_2)_3OAc$	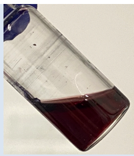 homogeneous
20	Dpppe	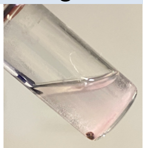 heterogeneous	44	$PPh_2(CH_2)_2Ac$	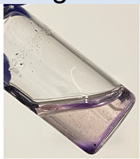 heterogeneous
21	Dcypp	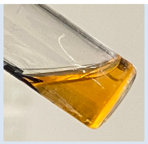 homogeneous	45	$PPh_2(CH_2)_2Ac$	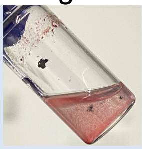 heterogeneous
22	Dcypb	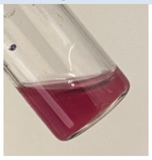 Homogeneous*	46	$PPh_2(CH_2)_4OTBS$	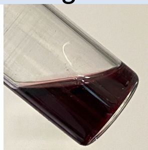 homogeneous
23	Dmpe	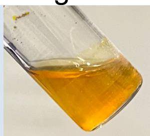 Homogeneous*	47	$PPh_2(CH_2)_2NH_2$	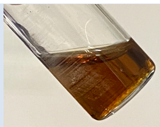 heterogeneous
24	Dtbpe	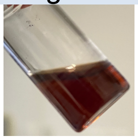 Homogeneous*	48	$PPh_2(CH_2)_2$(2-Py)	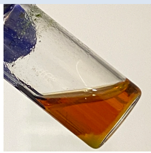 heterogeneous

* homogeneous at 70 °C.

As shown in Figure S3, we found that the solubility of the precatalyst strongly influences its catalytic performance. Typically, precatalysts that remain homogeneous in the reaction medium produce much higher yields compared to those that are partially or fully heterogeneous.

Model reaction I (MR1):

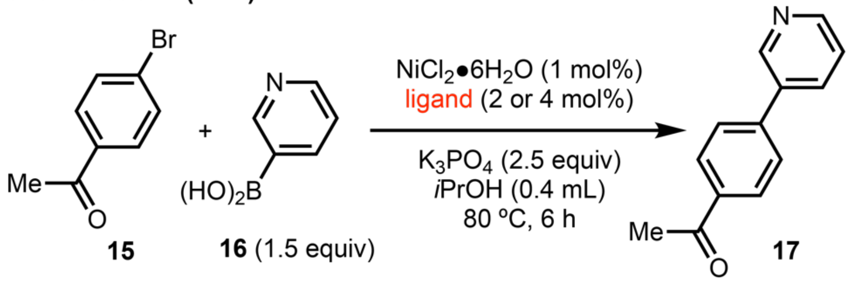

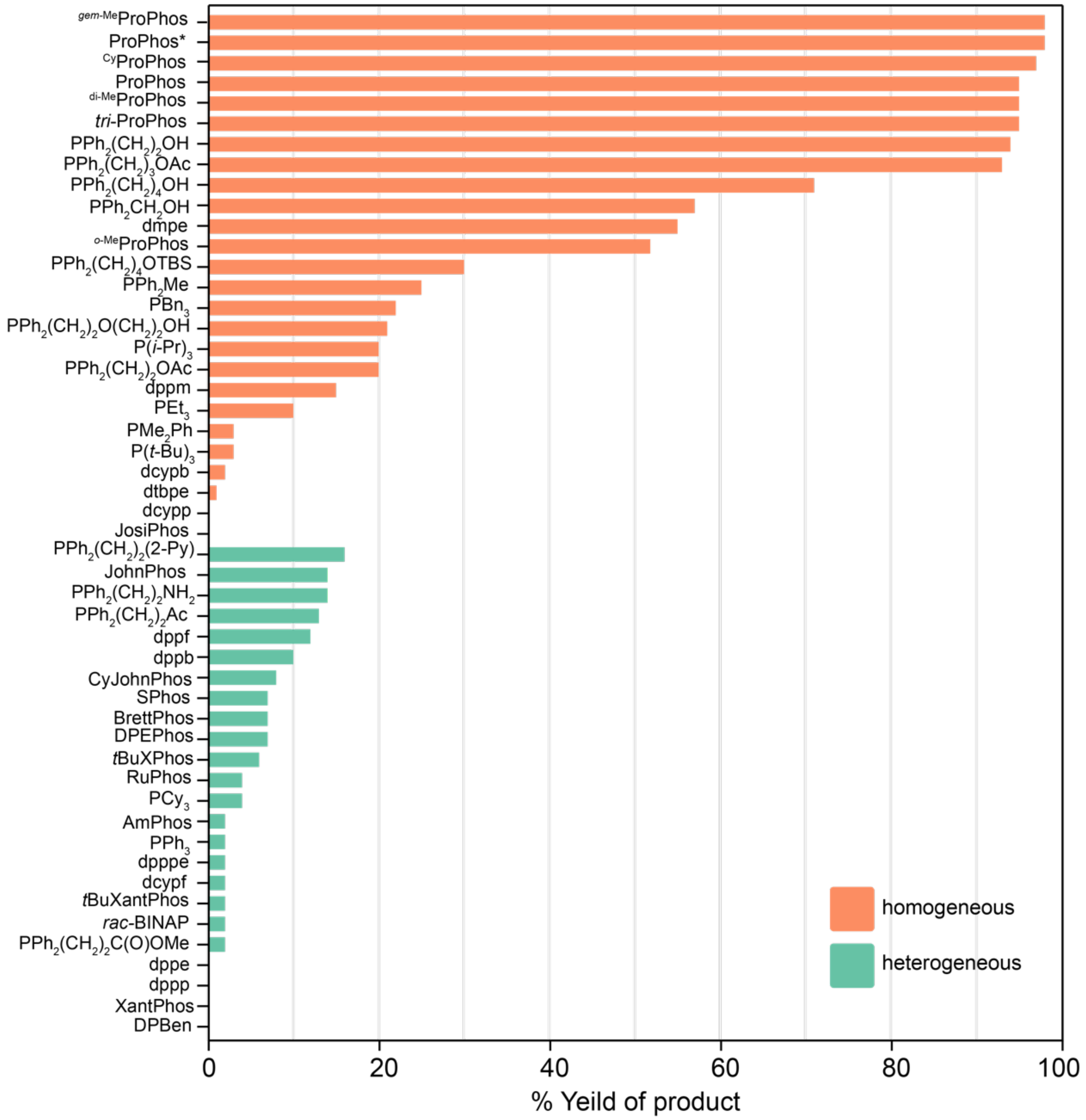

Figure S3. %Product conversion in model reaction I, as a relationship of the solubility of catalyst precursors, $NiCl_2•6H_2O$ and ligands, in *i*PrOH.

3.3 Effect of tethered Lewis basic group on catalytic activity

Based on the data for L1–L7 shown in Figure S4, we examined the influence of Lewis basic substituents on the $Ph_2PCH_2CH_2$ moiety. Unexpectedly, $Ph_2PCH_2CH_2OH$ (**13** or L1), which had shown poor performance under THF/H_2O conditions,[1] displayed excellent activity across all three model reactions in *i*PrOH. In contrast, ligands L2–L7 exhibited very low activity in model reaction 1 and variable outcomes in reactions 2 and 3. Notably, the bidentate phosphine dppe (L7) was completely inactive in all cases, likely due to its strong chelation to the Ni center.

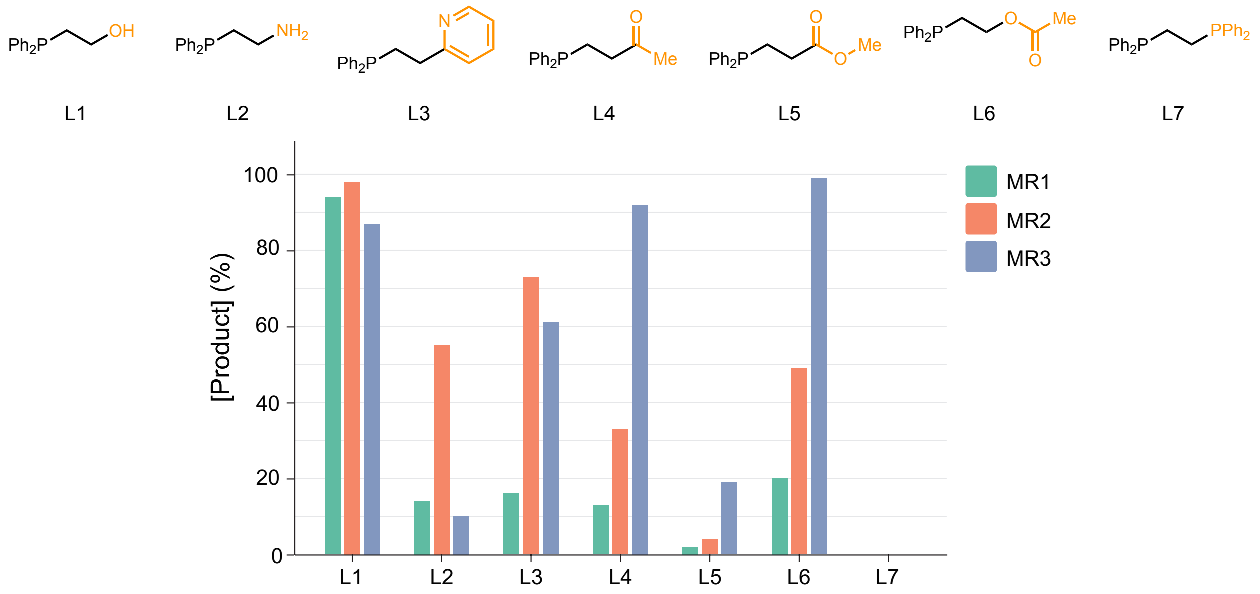

Figure S4. Comparison of the catalytic model reactions (MR1, MR2 and MR3) using the fragment of $PPh_2CH_2CH_2$- with different tethered functional groups. Detailed data in Table S1. Condition: [Ni] = 1 mol% and [ligand] = 2 or 4 mol%. Product = **17**.

3.4 Effect of size of (mono)phosphines on catalytic activity of Ni-SMC of heteroaromatics

The steric properties of monodentate phosphine ligands play a critical role in Ni-catalyzed Suzuki–Miyaura couplings. A distinctive steric descriptor, the minimum percent buried volume $\%V_{bur}$ (min), has been demonstrated to effectively distinguish active from inactive catalysts at a comparable threshold value.[8-10] Previous comprehensive analyses have primarily focused on Ni-SMC reactions involving non-heteroaromatic substrates. Using our dataset derived from heteroaromatic model reactions, we sought to examine whether the same trend applies. By defining 10% yield as the cut-off between active and inactive catalysts, we observed that monodentate phosphines with $\%V_{bur}$ (min) < 32 are generally active, consistent with earlier findings for non-heteroaromatics (Figure S5A). More specifically, the most active monodentate phosphines tend to have $\%V_{bur}$ (min) value in the range of 25–30. The ProPhos ligands, which can also be regarded as monodentate phosphines, were included in this analysis (Figure S6). Their $\%V_{bur}$ (min) values (25.6–30.5) fall within the "active" region. Even when increasing the cut-off to 50% (a level more relevant to process optimization), ProPhos ligands still outperform other monodentate phosphines (Figure S5B).

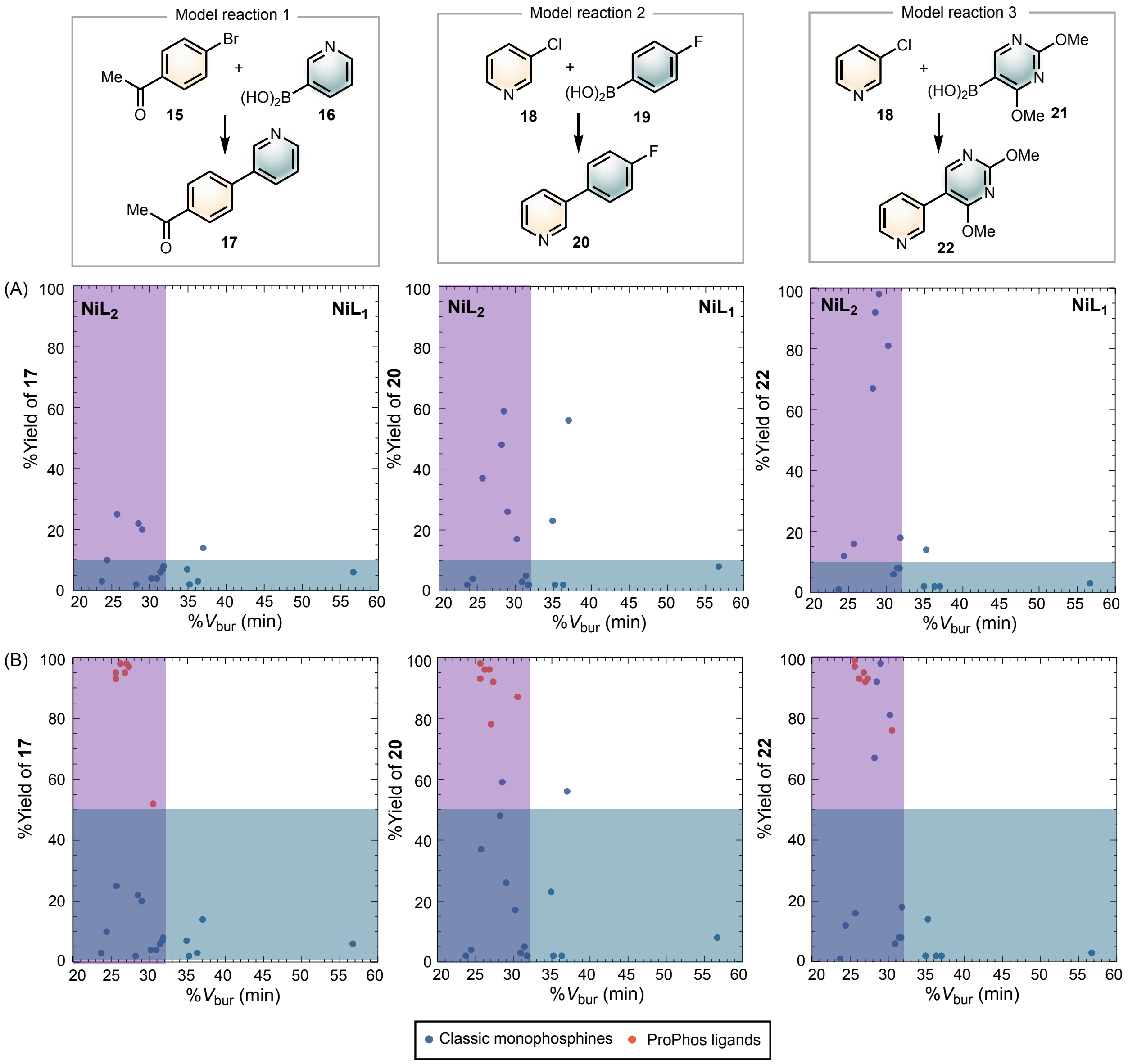

Figure S5. Investigation of monodentate phosphine steric parameters in Ni-SMC of heteroaromatic substrates. (A) Classic monodentate phosphines; (B) Classic monodentate phosphines and ProPhos ligands.

We next examined the steric effects among the ProPhos ligands and found that even a slight increase in steric bulk led to a noticeable decrease in catalytic activity, an effect that became more pronounced under very low catalyst loadings (Figure S6). This observation suggests that heterocycle inhibition is a key factor governing Ni-SMC of heteroaromatic substrates. The Ac-protected-ProPhos (pale blue) exhibited comparable activity to ProPhos (dark blue) at 1 mol% Ni loading. However, when the Ni loading was reduced to 0.1 mol%, a significant drop in activity was observed for the Ac-protected analogue, again underscoring the importance of the hydroxyl

group. The residual activity at higher catalyst loading is likely due to partial hydrolysis of the acetate protecting group under basic conditions, generating a small amount of free ProPhos in situ.

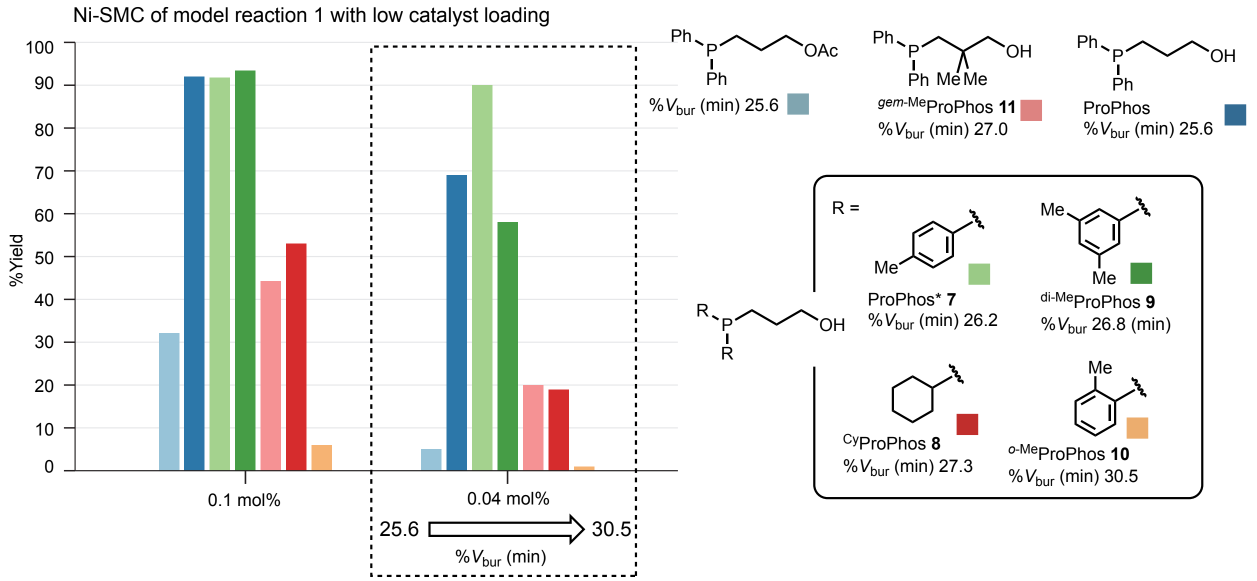

The $\%V_{bur}$ (min) values were either obtained from literature databases or calculated according to reported methods.[11]

Figure S6. Investigation of steric parameters of ProPhos ligands in Ni-SMC of heteroaromatic substrates at low catalyst loading.

Overall, while Ni-SMC of heteroaromatics still follows the general $\%V_{bur}$ (min)-based steric trend, optimizing the size of conventional monodentate phosphines has only a negligible impact on improving reactivity. For ProPhos ligands, a less bulky phosphorus center with stronger σ-donor capability is crucial for achieving efficient catalysis under extremely low catalyst loadings.

4. Reactivity Studies of (ProPhos)Ni catalyst in *i*PrOH

4.1 Kinetic studies of Ni-SMC

General experimental procedure for the product analysis using GC-FID

Followed by general procedure described in Section 3.1, $NiCl_2•6H_2O$ (1.0 equiv) and a phosphine (4.0 equiv, ProPhos in most cases) were used for preparing stock solutions. In most cases, 4-bromoacetophenone **15** and a boronic substrate (3-Py)$B(OH)_2$ **16** were weighed into a 2 mL crimp-top vial (or 4 mL vial). Solid K_3PO_4 was added to the vial. Subsequently, appropriate volumes of the Ni phosphine stock solution and deoxygenated *i*PrOH were added to reach a total volume of 0.4 mL (or 2 mL for the reactions run in 4 mL vial). The vial was sealed and removed from the glove box, then placed in a shaker. The reaction mixture was heated to 80°C with agitation at 800 rpm (or stirring at 800 rpm in an oil bath for 4 mL vial reactions). Reaction progress was monitored by preparing a series of ten identical samples. At specific time points, a sample was removed from the shaker. Each sample was extracted with ethyl acetate, and the resulting organic layer was collected and washed with water. An aliquot of the organic layer was analyzed by gas chromatography (GC-FID). Reactions were conducted in duplicate. Concentrations determined from duplicate runs had an error margin of ±2%. The k_{obs} values were simulated by Copasi (https://copasi.org/) and initial rates were obtained.

4.2 Substitution of Ni-ProPhos complexes with heterocycles

Why do we perform this substitution studies

Oxidative addition complexes, such as NiP_2XAr, have been established as the catalyst resting state in Ni-SMC in previous studies,[1] suggesting that catalyst inhibition (i.e. heterocycle coordination) most likely occurs at this stage. Prior studies have shown that $Ni(PPh_3)_2Cl$(o-Tol) is susceptible to pyridine/heterocycle coordination. Thus, we performed analogous substitutions to investigate how ProPhos ligands interact with excess heterocycles at high temperature.

General experimental procedure for the Ni speciation analysis by NMR

Ni complexes (6.8–7.4 µmol) with pyridine (10 equiv) were prepared in a 4 mL vial and transferred to an NMR tube with *i*PrOD-d_8 (0.6 mL). The NMR tube was sealed and placed in an oil bath for heating to 70 °C. Monitoring was performed using $^{31}P\{^1H\}$ NMR spectroscopy with an internal standard PPh_3 in a sealed capillary. Replicated runs indicate that the percentage consumption of the Ni complexes measured in these experiments has an error margin of ±3%.

Product analysis using GC in parallel catalyses with pyridine

Following general experimental procedure for the product analysis using GC (Section 4.1), Ni complexes (6.8 μmol, 10 mol%) were weighed into a 1.5 mL vial. B(3-Py)$(OH)_2$ **16** (1.5 equiv) was then added to the vial, followed by the addition of dry K_3PO_4 (2.5 equiv). Finally, 2-chlorotoluene (8.0 μL, 68 μmol, 1 equiv), pyridine (5.5 μL, 68 μmol, 1 equiv) and *i*PrOH (0.6 mL) were added to the mixture. %Conversions to product determined from these experiments had an error margin of ±2%.

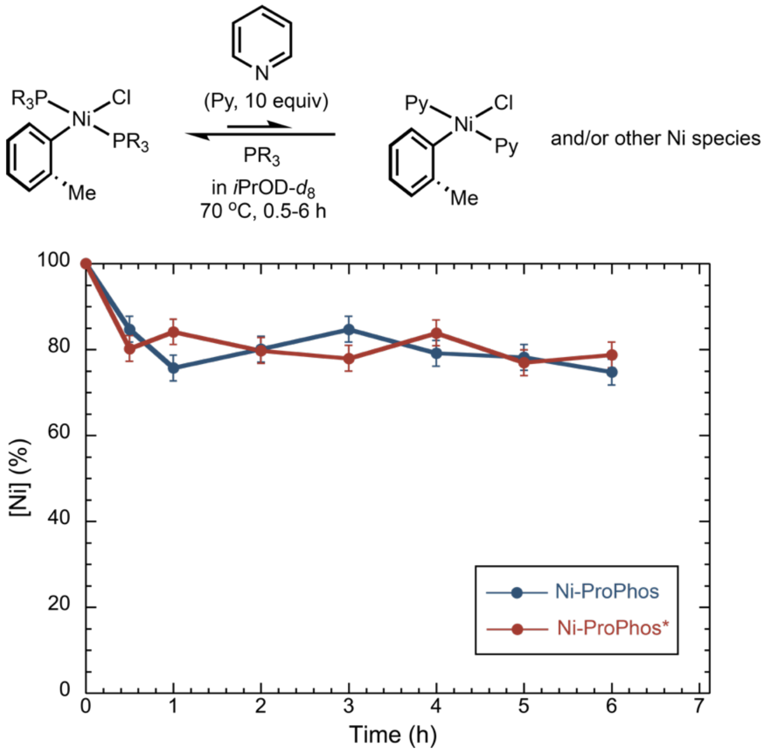

Figure S7 The consumption of the resting state complexes in the presence of excess pyridines, determined by $^{31}P\{^1H\}$ NMR. The error bars indicate no significant changes in the Ni-ProPhos and Ni-ProPhos* complexes, **23** and **25**, after treatment with pyridine, suggesting that equilibria may have been established.

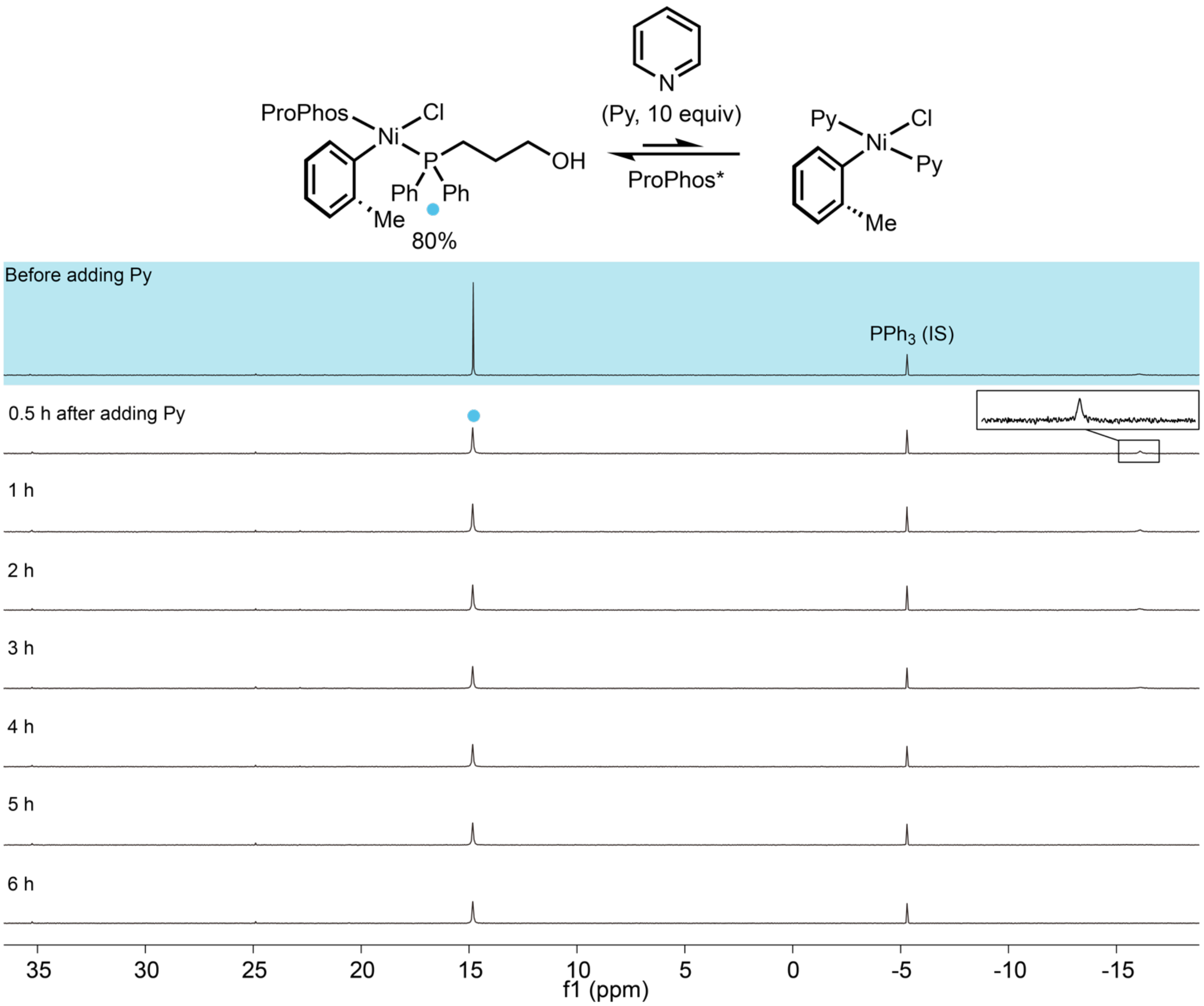

Figure S8. $^{31}P\{^{1}H\}$ NMR (202.47 MHz, *i*PrOD-*d*$_8$) spectra of the reaction of Ni(ProPhos)$_2$Cl(*o*-Tol) **23** with pyridine (10 equiv) at 70 °C overtime. The inset shows the broadening of free ProPhos, suggesting that it is likely involved in equilibria. Internal standard (IS): PPh_3 in a small capillary.

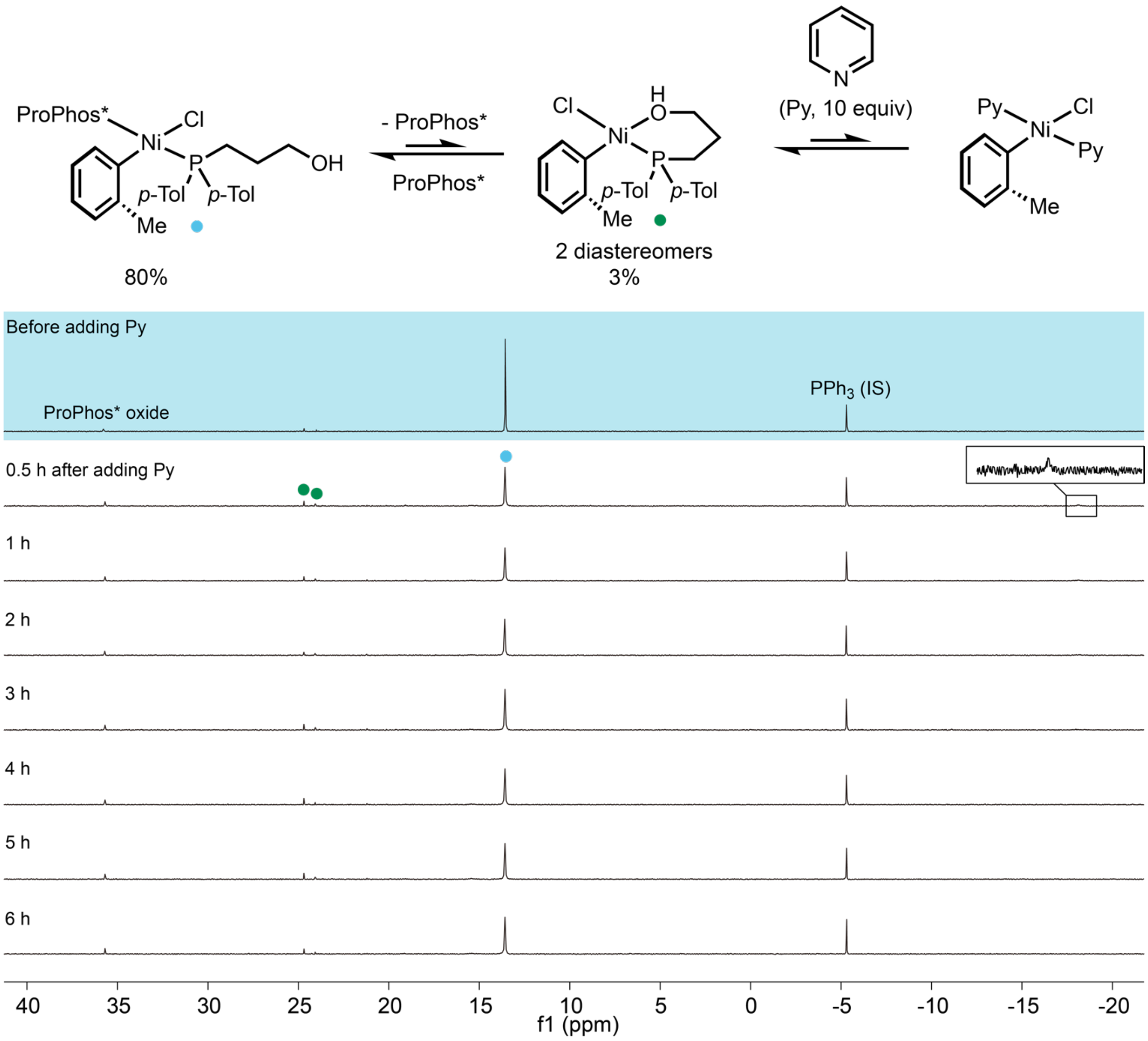

Figure S9 $^{31}P\{^{1}H\}$ NMR (202.47 MHz, *i*PrOD-d_8) spectra of the reaction of Ni(ProPhos*)$_2$Cl(*o*-Tol) **25** with pyridine (10 equiv) at 70 °C overtime. The inset shows the broadening of free ProPhos*, suggesting that it is likely involved in equilibria. Internal standard (IS): PPh_3 in a small capillary.

4.3 Relevance between heterocycle resistance and catalytic activity

To explore the influence of heterocycle coordination on the Ni-ProPhos-SMC system, Ni-ProPhos and Ni-ProPhos* resting-state complexes, **23** and **25**, were reacted with an excess of pyridine (Figures S7-9). Upon adding pyridine to the solution of these Ni complexes in *i*PrOD-d_8, an equilibrium with the heterocycle was observed, strongly favoring the resting state (~80% of total Ni). This equilibrium is hypothesized to play a critical role in maintaining a high concentration of the resting complex within the catalytic system. Furthermore, the ProPhos and ProPhos* ligands

were found to dissociate readily, facilitating efficient opening of coordination sites necessary for promoting transmetalation and oxidative addition.

To further evaluate the impact of heterocycle coordination or inhibition on catalytic activity, we conducted parallel catalytic reactions with **23** and **25** in the presence of pyridine (Scheme S3). The reactions achieved high product conversions within 3 h, indicating that pyridine coordination has minimal influence on the performance of the Ni-ProPhos-SMC system.

Scheme S3. Catalytic Reactions using Ni(PR_3)$_2$Cl(*o*-Tol) (**23** or **25**) with Excess Pyridine

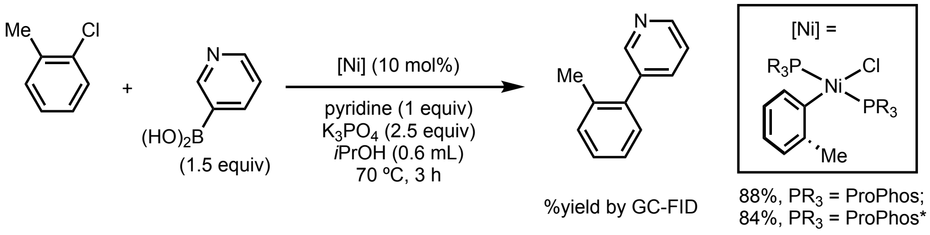

4.4 Competitive coordination of ProPhos and ProPhos*

We attribute the ability of ProPhos* to outperform ProPhos at extremely low catalyst loadings to its slightly stronger σ-donation. We propose that even this modest electronic difference becomes significant once the effect is amplified under such low catalyst concentrations, thereby helping the system resist catalyst inhibition.

Experimental details

Ni(ProPhos)$_2$Cl(*o*-Tol) (**23**, 5.4 mg, 8.0 μmol, 1.0 equiv) was weighed into a 4 mL vial containing a stir bar. ProPhos* (4.8 mg, 18 μmol, 2.2 equiv) was then added to the vial. *i*PrOD-d_8 (0.6 mL) was added to the vial and transferred to an NMR tube. Monitoring was performed using $^{31}P\{^1H\}$ NMR spectroscopy.

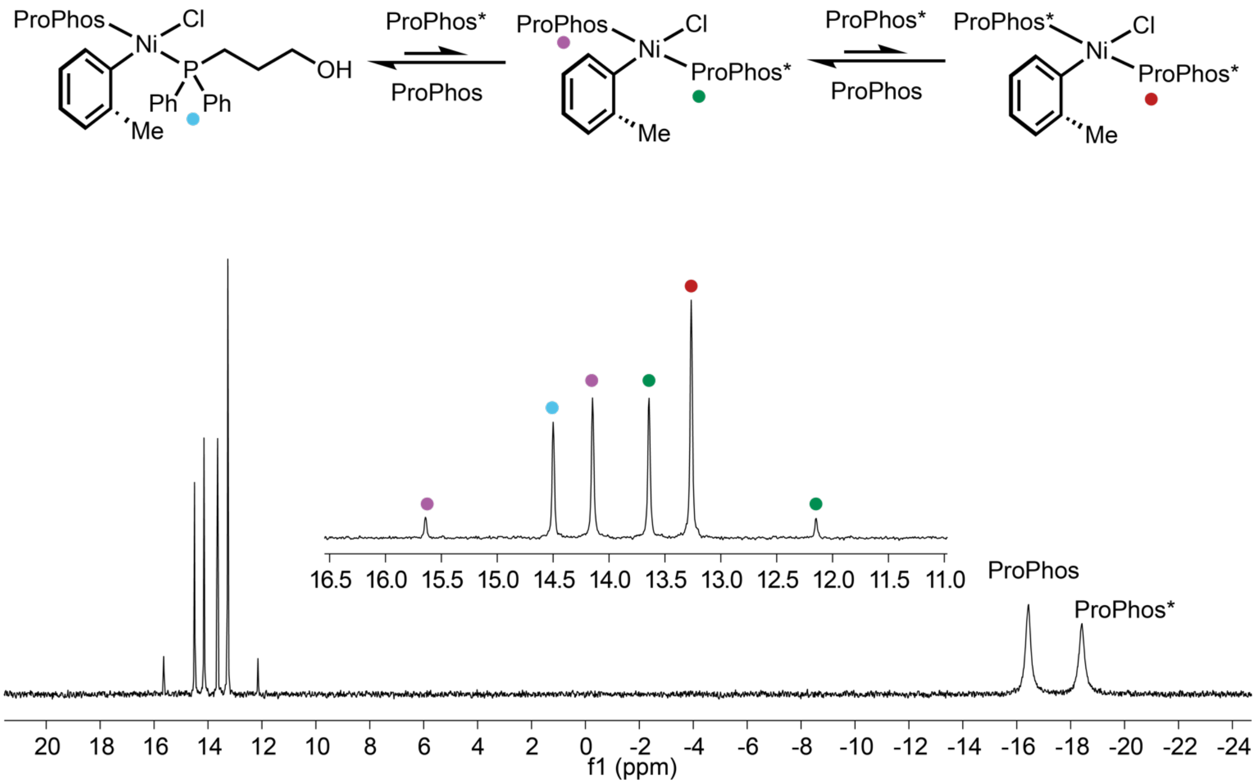

Figure S10. $^{31}P\{^{1}H\}$ NMR (202.47 MHz, *i*PrOD-d_8) spectrum of reaction of Ni(ProPhos)$_2$Cl(*o*-Tol) **23** with ProPhos* **7** at rt.

5. Substrate Scope using (ProPhos)Ni Catalysts

5.1 General procedure for synthesizing biaryl compounds

Method A – $NiCl_2•6H_2O$ in iPrOH

In most case, a vial was charged with $NiCl_2•6H_2O$ (1.4 mg, 6.0 μmol), *i*PrOH (2 mL) and a phosphine ligand (24 μmol). The vial was sealed, removed from the glove box, and placed in a shaker. It was then heated to 70 °C with agitation at 800 rpm for 30 min, resulting in a red stock solution. This solution was returned to the glove box for subsequent use. Appearance of the Ni precatalyst solution is shown in Table S2.

A separate vial was prepared with the aryl boronic acid or ester (0.30 mmol, 1.5 equiv), aryl halide (0.20 mmol if solid, 1.0 equiv), and K_3PO_4 (0.50 mmol, 2.5 equiv). If the aryl halide was liquid, it was added afterward (0.20 mmol, 1.0 equiv). The Ni phosphine stock solution and deoxygenated *i*PrOH (0.3-0.4 mL) were then added. The vial was sealed and removed from the glove box, then placed in a shaker. The reaction mixture was heated to 80 °C with agitation at 800 rpm for 16 h and then cooled to room temperature. The mixture was diluted with 10 mL of ethyl acetate (EtOAc) and washed with water (2 x 5 mL) or saturated NH_4Cl solution (2 x 5 mL). The combined aqueous layers were further extracted with 5 mL of EtOAc. An aliquot of the combined organic layers was analyzed by gas chromatography (GC) or ultra-performance liquid chromatography-mass spectrometry (UPLC-MS) to monitor the reaction progress. The combined organic layers were then concentrated in vacuo, and the crude product was purified by flash chromatography. Multiple Ni catalyst loadings (0.05–1 mol%) with three phosphine ligands, (ProPhos, ProPhos* **7**, or *tri*-ProPhos), respectively, were used for synthesizing these compounds. Unless otherwise specified, the isolated yields obtained using **7** are reported below.

Method B – $Ni(PPh_3)_2Cl(Naph)$ in 2-MeTHF/H_2O

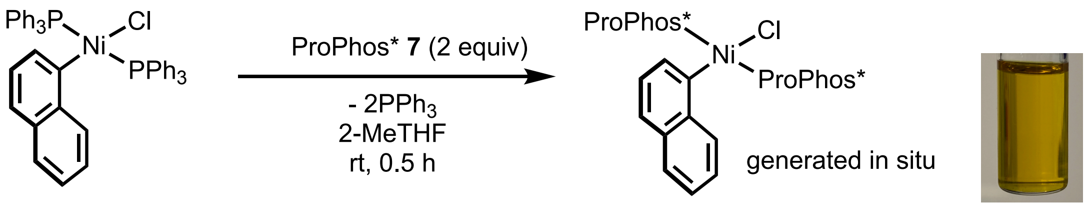

A vial was charged with $Ni(PPh_3)_2Cl(Naph)$ (1.5 mg, 2.0 μmol, 1 mol%), 2-MeTHF (0.3 mL) and ProPhos* **7** (4 μmol, 2 mol%). The vial was sealed, removed from the glove box, and placed in a shaker with agitation at 800 rpm for 30 min, resulting in an orange stock solution. This solution

was returned to the glove box for subsequent use. A separate vial was prepared with the aryl boronic ester (0.24 mmol, 1.2 equiv), aryl halide (0.20 mmol if solid, 1.0 equiv), and K_3PO_4 (0.50 mmol, 2.5 equiv). The Ni phosphine stock solution and extra 0.1 mL 2-MeTHF (rinsing the vial containing the Ni solution) were then added. After that, H_2O (80 µL) were added to the vial outside the glovebox, and then placed in a shaker. The reaction mixture was heated to 80 °C with agitation at 800 rpm for 16 h and then cooled to room temperature. The progress of the reaction and the isolation procedure were carried out as outlined in Method A.

5.2 Synthesis and characterization of Ni-SMC products

1-[4-(3-Pyridinyl)phenyl]ethenone (***17***)

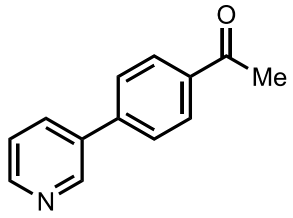

17

Method A: Using Ni loading (0.1 mol%) with ProPhos* **7**, 4-bromophenylethanone (39.8 mg, 0.200 mmol, 1.0 equiv) and pyridin-3-ylboronic acid (36.9 mg, 0.300 mmol, 1.5 equiv) were used, purified by flash chromatography (EtOAc;hexane), to give a white solid (35.5 mg, 0.180 mmol, 90%). The NMR data are consistent with our previous report[1] and the literature reports.[12]

^{1}H NMR (400.20 MHz, CDCl$_3$) δ: 8.88 (s, 1H), 8.70 – 8.58 (m, 1H), 8.07 (d, *J* = 8.3 Hz, 2H), 7.91 (dt, *J* = 8.0, 2.0 Hz, 1H), 7.68 (d, *J* = 8.3 Hz, 2H), 7.40 (dd, *J* = 7.9, 4.8 Hz, 1H), 2.64 (s, 3H).

1-(4-(2,4-Dimethoxypyrimidin-5-yl)phenyl)ethan-1-one (***27***)

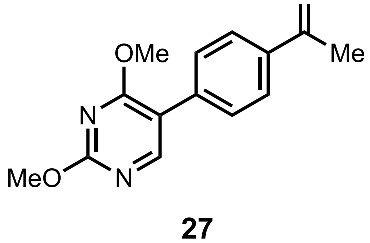

27

Method A: Using Ni loading (0.05 mol%) with ProPhos* **7**, 4-bromophenylethanone (39.8 mg, 0.200 mmol, 1.0 equiv) and 2,4-dimethoxypyrimidin-5-yl)boronic acid (55.2 mg, 0.300 mmol, 1.5 equiv) were used, purified by flash chromatography (EtOAc:hexane), to give a white solid (49.0 mg, 0.190 mmol, 95%).

^{1}H NMR (400.30 MHz, $CDCl_3$) δ: 8.31 (s, 1H), 8.05 – 7.98 (m, 2H), 7.64 – 7.58 (m, 2H), 4.05 (s, 3H, OMe), 4.04 (s, 3H, OMe), 2.63 (s, 3H, Me).

^{13}C{^{1}H} NMR (100.67 MHz, $CDCl_3$) δ: 197.7 (s), 168.3 (s), 165.1 (s), 158.0 (s), 138.4 (s), 136.3 (s), 129.0 (s), 128.6 (s), 115.3 (s), 55.2 (s, OMe), 54.4 (s, OMe), 26.8 (s, Me).

HRMS (ESI-TOF, CH_3CN) m/z: $[M + Na]^+$ calculated for $C_{14}H_{15}N_2O_3$ 259.1077, found 259.1067.

*<u>tert-Butyl</u> <u>2-(4-acetylphenyl)-1H-pyrrole-1-carboxylate</u> (**28**)*

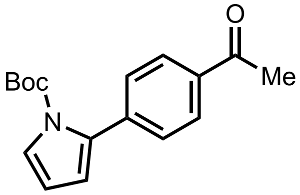

28

Method A: Using Ni loading (3 mol%) with ProPhos* **7**, 4-bromophenylethanone (39.8 mg, 0.200 mmol, 1.0 equiv) and (1-(tert-butoxycarbonyl)-1H-pyrrol-2-yl)boronic acid (63.3 mg, 0.300 mmol, 1.5 equiv) were used, purified by flash chromatography (EtOAc:hexane), to give a white solid (49.0 mg, 0.190 mmol, 95%). Reducing the catalyst loading to 1 mol% resulted in a marked drop in yield, likely due to rapid protodeboronation of the boronic acid. The NMR data of the isolated compound are consistent with the literature report.[13]

^{1}H NMR (400.30 MHz, $CDCl_3$) δ: 7.98 – 7.91 (m, 2H), 7.48 – 7.42 (m, 2H), 7.38 (dd, J = 3.3, 1.8 Hz, 1H), 6.28 – 6.23 (m, 2H), 2.62 (s, 3H, Me), 1.40 (s, 9H, Me at *t*Bu).

^{13}C{^{1}H} NMR (100.67 MHz, $CDCl_3$) δ: 197.9 (s, C=O), 149.2 (s, C=O), 139.1 (s), 135.7 (s), 134.1 (s), 129.2 (s), 127.9 (s), 123.7 (s), 115.7 (s), 111.0 (s), 84.2 (s, C at *t*Bu), 27.8 (s, Me at *t*Bu), 26.8 (s, Me).

1-(4-(Pyrimidin-5-yl)phenyl)ethan-1-one (***29***)

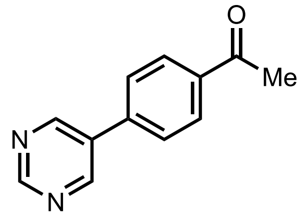

29

Method A: Using Ni loading (0.1 mol%) with ProPhos* **7**, 4-bromophenylethanone (39.8 mg, 0.200 mmol, 1.0 equiv) and pyrimidin-5-ylboronic acid (37.2 mg, 0.300 mmol, 1.5 equiv) were used, purified by flash chromatography (EtOAc;hexane), to give a white solid (36.6 mg, 0.185 mmol, 92%). The NMR data are consistent with the literature report.[14]

^{1}H NMR (400.30 MHz, CDCl$_3$) δ: 9.26 (s, 1H), 9.00 (s, 2H), 8.14 – 8.07 (m, 2H), 7.73 – 7.66 (m, 2H), 2.66 (s, 3H, Me).

^{13}C{^{1}H} NMR (100.67 MHz, CDCl$_3$) δ: 197.4 (s), 158.3 (s), 155.2 (s), 138.9 (s), 137.4 (s), 133.4 (s), 129.5 (s), 127.4 (s), 26.9 (s).

1-(4-(6-Methoxy-2-methylpyridin-3-yl)phenyl)ethan-1-one (***30***)

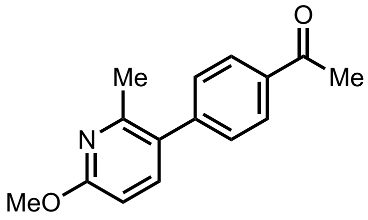

30

Method A: Using Ni loading (0.1 mol%) with ProPhos* **7**, 4-bromophenylethanone (39.8 mg, 0.200 mmol, 1.0 equiv) and (6-methoxy-2-methylpyridin-3-yl)boronic acid (50.1 mg, 0.300 mmol, 1.5 equiv) were used, purified by flash chromatography (EtOAc:hexane), to give a white solid (41.1 mg, 0.170 mmol, 85%).

^{1}H NMR (400.30 MHz, CDCl$_3$) δ: 8.01 (d, J = 8.4 Hz, 2H), 7.47 – 7.37 (m, 3H), 6.64 (d, J = 8.4 Hz, 1H), 3.97 (d, J = 1.6 Hz, 3H, OMe), 2.64 (d, J = 1.5 Hz, 3H, Me), 2.42 (s, 3H, Me).

^{13}C{^{1}H} NMR (100.67 MHz, CDCl$_3$) δ: 197.9 (s, C=O), 163.1 (s), 153.3 (s), 145.3 (s), 140.1 (s), 135.8 (s), 129.7 (s), 128.7 (s), 128.5 (s), 107.8 (s), 53.6 (s, OMe), 26.8 (s, Me), 23.2 (s, Me).

HRMS (ESI-TOF, CH_3CN) m/z: $[M + H]^+$ calculated for $C_{15}H_{16}NO_2$ 242.1176, found 242.1163.

(S)-3-(4'-Acetyl-[1,1'-biphenyl]-4-yl)-2-((tert-butoxycarbonyl)amino)propanoic acid ***(31)***

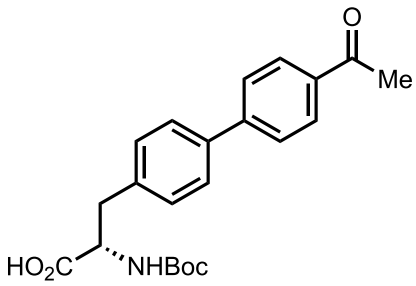

31

Method A: Using Ni loading (3 mol%) with ProPhos* **7**, 4-bromophenylethanone (39.8 mg, 0.200 mmol, 1.0 equiv) and (*S*)-3-(4-boronophenyl)-2-((*tert*-butoxycarbonyl)amino)propanoic acid (92.7 mg, 0.300 mmol, 1.5 equiv) were used, purified by flash chromatography (DCM:MeOH), to give a white solid (54.5 mg, 0.142 mmol, 71%).

^{1}H NMR (400.30 MHz, DMSO-*d*$_6$) δ: 7.99 (d, *J* = 8.1 Hz, 2H), 7.75 (d, *J* = 8.1 Hz, 2H), 7.60 (d, *J* = 7.7 Hz, 2H), 7.33 (d, *J* = 7.8 Hz, 2H), 6.42 (d, *J* = 7.3 Hz, 1H), 4.08 (br s, 1H, CH), 3.17 (br s, 1H, CH), 2.96 (dd, *J* = 13.4, 7.8 Hz, 1H, CH), 2.58 (s, 3H, Me), 1.31 (s, 9H, Me at *t*Bu). OH at at CO_2H was not observed presumably due to rapid exchange with H_2O.

^{13}C{^{1}H} NMR (100.67 MHz, DMSO-*d*$_6$) δ: 197.5 (s, C=O), 155.0 (s, C=O), 144.5 (s), 139.4 (s), 136.5 (s), 135.4 (s), 130.2 (s), 128.9 (s), 126.6 (s), 126.5 (s), 77.7 (s, C at *t*Bu), 55.9 (s, CH), 37.0 (s, CH), 28.2 (s, Me at *t*Bu), 26.8 (s, Me). The signal due to C=O at CO_2H was not observed presumably due to rapid exchange with H_2O.

HRMS (ESI-TOF, CH_3OH) m/z: $[M + Na]^+$ calculated for $C_{22}H_{25}NO_5Na$ 406.1625, found 406.1625.

<u>1-(4-(1-Methyl-3-(trifluoromethyl)-1H-pyrazol-5-yl)phenyl)ethan-1-one</u> (***32***)

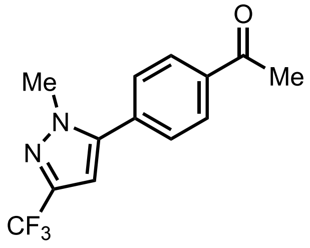

32

Method A: Using Ni loading (3 mol%) with ProPhos* **7**, 4-bromophenylethanone (39.8 mg, 0.200 mmol, 1.0 equiv) and (1-methyl-3-(trifluoromethyl)-1H-pyrazol-5-yl)boronic acid (58.2 mg, 0.300 mmol, 1.5 equiv) were used. Since the compound was unstable under flash chromatography conditions, its yield was quantified by q^{1}H NMR (82%) using 1,3,5-trimethoxybenzene as the internal reference. Reducing the catalyst loading to 1 mol% resulted in a marked drop in yield, likely due to rapid protodeboronation of the boronic acid. The NMR data of the crude are consistent with the literature report.[15] The identical SMC still requires 2 mol% Pd, giving 85% yield.[15]

^{1}H NMR (400.30 MHz, $CDCl_3$) δ: 8.13 – 8.00 (m, 2H), 7.58 – 7.51 (m, 2H), 6.63 (s, 1H), 3.95 (s, 3H, Me), 2.66 (s, 3H, Me).

^{19}F{^{1}H} NMR (470.61 MHz, $CDCl_3$) δ: -62.13 (s).

^{13}C{^{1}H} NMR (100.67 MHz, $CDCl_3$) δ: 197.2 (s, C=O), 144.0 (s), 141.8 (q, *J* = 38 Hz), 137.4 (s), 133.6 (s), 129.0 (s), 128.9 (s), 121.2 (q, *J* = 269 Hz), 104.9 (q, *J* = 2 Hz), 38.3 (s, Me), 26.7 (s, Me).

1-(4-(1-Methyl-1H-pyrazol-4-yl)phenyl)ethan-1-one (***6***)

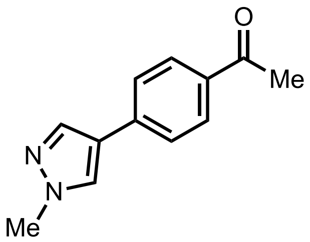

6

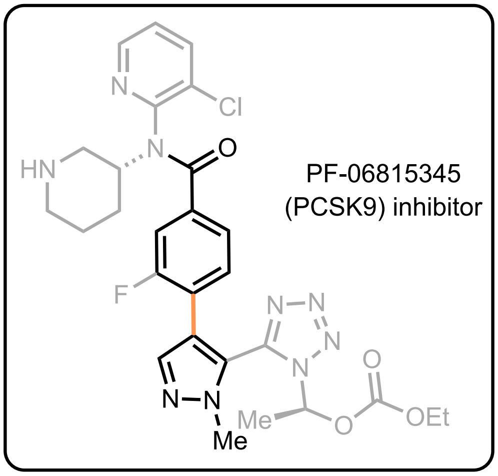

Method A: Using Ni loading (0.05 mol%) with ProPhos* **7**, 4-bromophenylethanone (39.8 mg, 0.200 mmol, 1.0 equiv) and (1-methyl-1*H*-pyrazol-4-yl)boronic acid (37.8 mg, 0.300 mmol, 1.5 equiv) were used, purified by flash chromatography (EtOAc:hexane), to give a white solid (36.0 mg, 0.180 mmol, 90%). The NMR data of the isolated product are consistent with our previous report[7] and the literature report.[16]

^{1}H NMR (400.30 MHz, $CDCl_3$) δ: 7.95 (d, *J* = 8.6 Hz, 2H), 7.83 (s, 1H), 7.70 (s, 1H), 7.54 (d, *J* = 8.5 Hz, 2H), 3.96 (s, 3H, Me), 2.60 (s, 3H, Me).

*1-[4-(1*H*-Indol-6-yl)phenyl]ethenone* (***33***)

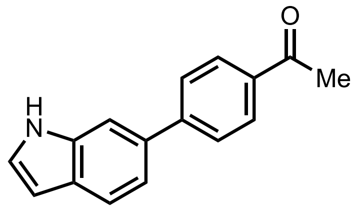

33

Method A: Using Ni loading (0.1 mol%) with ProPhos* **7**, 4-bromophenylethanone (39.8 mg, 0.200 mmol, 1.0 equiv) and (1*H*-indol-6-yl)boronic acid (48.3 mg, 0.300 mmol, 1.5 equiv) were used, purified by flash chromatography (EtOAc:hexane), to give a brown solid (44.0 mg, 0.187 mmol, 94%). The NMR data of the isolated product are consistent with our previous report in $CDCl_3$.[7]

^{1}H NMR (400.30 MHz, DMSO-d_6) δ: 11.25 (s, 1H, NH), 8.03 (dd, *J* = 8.3, 2.4 Hz, 2H), 7.83 (dd, *J* = 8.7, 2.7 Hz, 2H), 7.74 (s, 1H), 7.65 (d, *J* = 8.2 Hz, 1H), 7.48 – 7.33 (m, 2H), 6.47 (q, *J* = 4.9, 3.7 Hz, 1H), 2.61 (s, 3H, Me).

1-(2',6'-Dimethoxy[1,1'-biphenyl]-4-yl)ethenone (***34***)

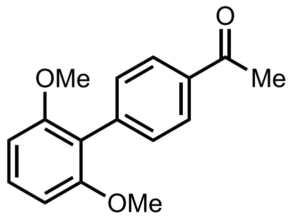

34

Method B: Using Ni loading (1 mol%) with ProPhos* **7**, 4-bromophenylethanone (39.8 mg, 0.200 mmol, 1.0 equiv) and 2-(2,6-Dimethoxyphenyl)-4,4,5,5-tetramethyl-1,3,2-dioxaborolane (63.4 mg, 0.240 mmol, 1.2 equiv) were used, purified by flash chromatography (hexane:EtOAc), to give a white solid (43.5 mg, 0.170 mmol, 85%). The NMR data are consistent with the literature reports.[17]

^{1}H NMR (400.30 MHz, CDCl$_3$) δ: 8.05 – 7.96 (m, 2H), 7.49 – 7.43 (m, 2H), 7.31 (t, *J* = 8.4 Hz, 1H), 6.67 (d, *J* = 8.4 Hz, 2H), 3.74 (s, 6H, OMe), 2.63 (s, 3H, Me).

^{13}C{^{1}H} NMR (100.67 MHz, CDCl$_3$) δ: 198.1 (s), 157.6 (s), 139.9 (s), 135.6 (s), 131.4 (s), 129.5 (s), 127.9 (s), 118.5 (s), 104.3 (s), 56.0 (s, OMe), 26.8 (s, Me).

6-(2,6-Dimethoxyphenyl)quinoline (***35***)

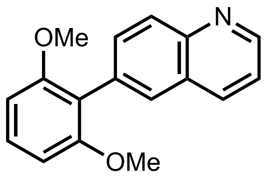

35

Method B: Using Ni loading (1 mol%) with ProPhos* **7**, 6-chloroisoquinoline (32.7 mg, 0.200 mmol, 1.0 equiv) and 2-(2,6-dimethoxyphenyl)-4,4,5,5-tetramethyl-1,3,2-dioxaborolane (63.4 mg, 0.240 mmol, 1.2 equiv) were used, purified by flash chromatography (hexane:EtOAc), to give a white solid (44.2 mg, 0.167 mmol, 83%). The NMR data are consistent with the literature report.[18]
^{1}H NMR (400.30 MHz, CDCl$_3$) δ: 8.90 (dd, *J* = 4.3, 1.7 Hz, 1H), 8.19 – 8.10 (m, 2H), 7.82 (d, *J* = 1.8 Hz, 1H), 7.73 (dd, *J* = 8.7, 1.9 Hz, 1H), 7.40 – 7.31 (m, 2H), 6.70 (d, *J* = 8.4 Hz, 2H), 3.75 (s, 6H, OMe).

$^{13}C\{^1H\}$ NMR (100.67 MHz, $CDCl_3$) δ: 157.8 (s), 150.2 (s), 147.5 (s), 136.3 (s), 133.3 (s), 132.8 (s), 129.7 (s), 129.3 (s), 128.5 (s), 128.2 (s), 120.9 (s), 118.7 (s), 104.3 (s), 56.0 (s, OMe).

5-(2,6-Dimethoxyphenyl)pyrimidine (***36***)

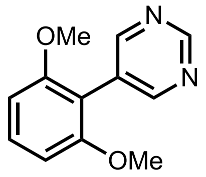

36

Method B: Using Ni loading (1 mol%) with ProPhos* **7**, 5-bromopyrimidine (31.8 mg, 0.200 mmol, 1.0 equiv) and 2-(2,6-dimethoxyphenyl)-4,4,5,5-tetramethyl-1,3,2-dioxaborolane (63.4 mg, 0.240 mmol, 1.2 equiv) were used, purified by flash chromatography (hexane:EtOAc), to give a white solid (38.1 mg, 0.176 mmol, 88%).

^1H NMR (400.30 MHz, $CDCl_3$) δ: 9.11 (s, 1H), 8.74 (s, 2H), 7.35 (t, J = 8.4 Hz, 1H), 6.67 (d, J = 8.4 Hz, 2H), 3.77 (s, 6H, OMe).

$^{13}C\{^1H\}$ NMR (100.67 MHz, $CDCl_3$) δ: 158.7 (s), 157.7 (s), 156.7 (s), 130.6 (s), 128.4 (s), 112.1 (s), 104.2 (s), 56.0 (s, OMe).

HRMS (ESI-TOF, CH_3CN) m/z: $[M + H]^+$ calculated for $C_{12}H_{13}N_2O_2$ 217.0972, found 217.0977.

3-(2-Methylphenyl)pyridine (***37***)

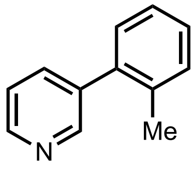

37

Method A: Using Ni loading (1 mol%) with ProPhos* **7**, 1-chloro-2-methylbenzene (23.4 μL, 0.200 mmol, 1.0 equiv) and pyridin-3-ylboronic acid (36.9 mg, 0.300 mmol, 1.5 equiv) were used, purified by flash chromatography (EtOAc:hexane), to give a colorless oil (30.4 mg, 0.180 mmol, 90%). The NMR data are consistent with our previous report[7] and the literature reports.[19]

^{1}H NMR (400.30 MHz, $CDCl_3$) δ: 8.65 – 8.53 (m, 2H), 7.64 (dq, *J* = 7.9, 1.6, 1.1 Hz, 1H), 7.37 – 7.19 (m, 5H), 2.27 (s, 3H, Me).

<u>*2-Methyl-6-(pyridin-3-yl)pyrazine*</u> *(**38**)*

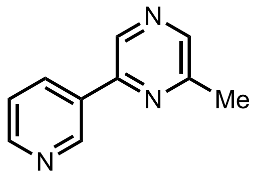

38

Method A: Using Ni loading (0.1 mol%) with ProPhos* **7**, 2-chloro-6-methylpyrazine (25.7, 0.200 mmol, 1.0 equiv) and pyridin-3-ylboronic acid (36.9 mg, 0.300 mmol, 1.5 equiv) were used, purified by flash chromatography (EtOAc:hexane), to give a colours oil (29.1 mg, 0.170 mmol, 85%).

^{1}H NMR (400.30 MHz, $CDCl_3$) δ: 9.15 (dd, *J* = 2.3, 0.9 Hz, 1H), 8.77 (s, 1H), 8.61 (dd, *J* = 4.8, 1.7 Hz, 1H), 8.37 (s, 1H), 8.24 (dt, *J* = 7.9, 2.0 Hz, 1H), 7.34 (ddd, *J* = 8.0, 4.9, 0.9 Hz, 1H), 2.56 (s, 3H, Me).

^{13}C{^{1}H} NMR (100.67 MHz, $CDCl_3$) δ: 153.6 (s), 150.4 (s), 149.0 (s), 148.0 (s), 143.4 (s), 138.6 (s), 134.2 (s), 132.1 (s), 123.6 (s), 21.6 (s, Me).

HRMS (ESI-TOF, CH_3CN) m/z: $[M + H]^+$ calculated for $C_{10}H_{10}N_3$ 172.0869, found 172.0875.

<u>*5-Fluoro-2'-methoxy-2,5'-bipyrimidine*</u> *(**39**)*

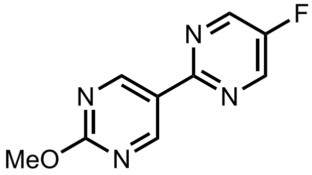

39

Method A: Using Ni loading (0.1 mol%) with ProPhos* **7**, 2-chloro-5-fluoropyrimidine (18.4 µL, 0.200 mmol, 1.0 equiv) and (2-methoxypyrimidin-5-yl)boronic acid (46.2 mg, 0.300 mmol, 1.5 equiv) were used, purified by flash chromatography (EtOAc:hexane), to give a white solid (39.8 mg, 0.193 mmol, 97%).

^{1}H NMR (500.20 MHz, CDCl$_3$) δ: 9.44 (s, 2H), 8.66 (s, 2H), 4.11 (s, 3H, OMe).

^{19}F{^{1}H} NMR (470.61 MHz, CDCl$_3$) δ: -138.91 (s).

^{13}C{^{1}H} NMR (125.79 MHz, CDCl$_3$) δ: 166.7 (s), 159.6 (s), 157.7 (d, *J* = 6 Hz), 157.0 (d, *J* = 266 Hz), 145.4 (d, *J* = 20 Hz), 124.5 (s), 55.5 (s, OMe).

HRMS (ESI-TOF, CH$_3$OH) m/z: [M + H]$^+$ calculated for $C_9H_8FN_4O$ 207.0677, found 207.0685.

*Ethyl 2-methyl-2-(4-(pyridin-3-yl)phenoxy)propanoate (**40**)*

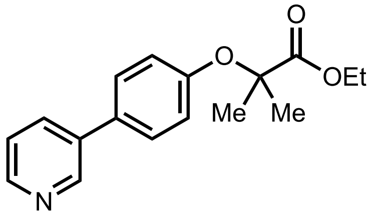

40 from clofibrate

Method A: Using Ni loading (1 mol%) with ProPhos* **7**, clofibrate (48.5 mg, 0.200 mmol, 1.0 equiv) and pyridin-3-ylboronic acid (36.9 mg, 0.300 mmol, 1.5 equiv) were used, purified by flash chromatography (DCM:MeOH), to give a white solid (51.1 mg, 0.179 mmol, 90%).

^{1}H NMR (400.30 MHz, CDCl$_3$) δ: 8.80 (d, *J* = 2.3 Hz, 1H), 8.54 (dd, *J* = 4.9, 1.6 Hz, 1H), 7.80 (ddd, *J* = 7.9, 2.3, 1.5 Hz, 1H), 7.49 – 7.41 (m, 2H), 7.37 – 7.28 (m, 1H), 6.98 – 6.89 (m, 2H), 4.24 (q, *J* = 7.1 Hz, 2H, CH$_2$), 1.63 (s, 6H, Me), 1.25 (t, *J* = 7.1 Hz, 3H, Me).

^{13}C{^{1}H} NMR (100.67 MHz, CDCl$_3$) δ: 174.2 (s, C=O), 155.8 (s), 148.1 (s), 136.2 (s), 134.0 (s), 131.5 (s), 128.0 (s), 123.7 (s), 119.5 (s), 79.3 (s), 61.6 (s), 25.5 (s, Me), 14.2 (s, Me). One quaternary carbon was not assigned.

HRMS (ESI-TOF, CH$_3$CN) m/z: [M + H]$^+$ calculated for $C_{17}H_{20}NO_3$ 286.1438, found 286.1440.

3-(3-(Methylsulfonyl)phenyl)pyridine (***41***)

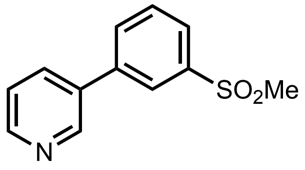

41

Method A: For the lowest Ni loading (0.3 mol%) with ProPhos, 1-bromo-3-(methylsulfonyl)benzene (47.0 mg, 0.200 mmol, 1.0 equiv) and pyridin-3-ylboronic acid (36.9 mg, 0.300 mmol, 1.5 equiv) were used, purified by flash chromatography (EtOAc:hexane), to give a light yellow oily solid (39.9 mg, 0.171 mmol, 86%). The NMR data are consistent with the literature reports.[20]

^{1}H NMR (400.30 MHz, $CDCl_3$) δ: 8.88 (dd, *J* = 2.4, 0.9 Hz, 1H), 8.67 (dd, *J* = 4.8, 1.6 Hz, 1H), 8.16 (t, *J* = 1.8 Hz, 1H), 7.99 (ddd, *J* = 7.8, 1.9, 1.1 Hz, 1H), 7.93 (ddd, *J* = 7.9, 2.4, 1.6 Hz, 1H), 7.88 (ddd, *J* = 7.8, 1.9, 1.1 Hz, 1H), 7.71 (td, *J* = 7.8, 0.5 Hz, 1H), 7.43 (ddd, *J* = 7.9, 4.9, 0.9 Hz, 1H), 3.11 (s, 3H, Me).

^{13}C{^{1}H} NMR (100.67 MHz, $CDCl_3$) δ: 149.7 (s), 148.3 (s), 141.8 (s), 139.6 (s), 134.9 (s), 134.7 (s), 132.4 (s), 130.4 (s), 127.0 (s), 126.1 (s), 124.0 (s), 44.7 (s, Me).

3-(4-(Methylsulfonyl)phenyl)pyridine (***42***)

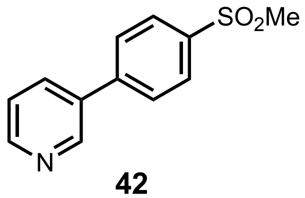

42

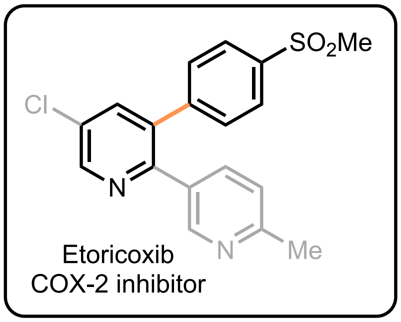

Method A: Using Ni loading (1 mol%) with ProPhos* **7**, 1-bromo-4-(methylsulfonyl)benzene (47.0 mg, 0.200 mmol, 1.0 equiv) and pyridin-3-ylboronic acid (36.9 mg, 0.300 mmol, 1.5 equiv) were used, purified by flash chromatography (EtOAc:hexane), to give a white solid (28.2 mg, 0.121 mmol, 60%). The NMR data are consistent with our previous report[7] and the literature reports.[21]

^{1}H NMR (400.30 MHz, $CDCl_3$) δ: 8.88 (d, *J* = 2.4 Hz, 1H), 8.68 (dd, *J* = 4.9, 1.6 Hz, 1H), 8.10 – 8.03 (m, 2H), 7.92 (ddd, *J* = 7.9, 2.4, 1.6 Hz, 1H), 7.81 – 7.75 (m, 2H), 7.44 (ddd, *J* = 7.9, 4.8, 0.9 Hz, 1H), 3.10 (s, 3H, Me).

2-(1-Methylazepan-4-yl)-4-(4-(pyridin-3-yl)benzyl)phthalazin-1(2H)-one ***(43)***

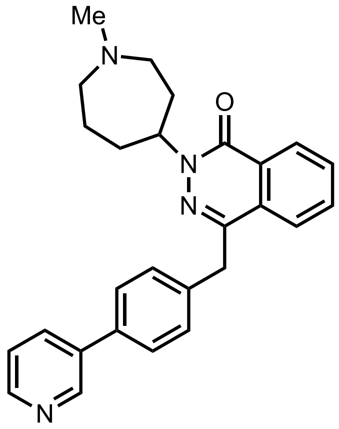

43 from azelastine

Method A: Using Ni loading (0.1 mol%) with ProPhos* **7**, azelastine (76.4 mg, 0.200 mmol, 1.0 equiv) and pyridin-3-ylboronic acid (36.9 mg, 0.300 mmol, 1.5 equiv) were used, the crude is sufficient clean, which was isolated as an IPA adduct without column purification, thus its yield was quantified by q^{1}H NMR (97%) using 1,3,5-trimethoxybenzene as the internal reference.

^{1}H NMR (400.30 MHz, $CDCl_3$) δ: 8.78 (dd, *J* = 2.4, 0.9 Hz, 1H), 8.53 (dd, *J* = 4.8, 1.6 Hz, 1H), 8.43 (ddd, *J* = 6.3, 3.0, 1.8 Hz, 1H), 7.80 (ddd, *J* = 7.9, 2.4, 1.6 Hz, 1H), 7.74 – 7.63 (m, 3H), 7.52 – 7.47 (m, 2H), 7.36 (d, *J* = 7.7 Hz, 2H), 7.30 (ddd, *J* = 7.9, 4.8, 0.9 Hz, 1H), 5.35 (tt, *J* = 9.1, 5.4 Hz, 1H), 4.33 (s, 2H), 2.78 (ddd, *J* = 13.5, 7.4, 2.5 Hz, 1H), 2.71 – 2.48 (m, 4H), 2.34 (s, 3H, Me), 2.30 – 2.19 (m, 1H), 2.19 – 1.99 (m, 3H), 1.99 – 1.86 (m, 1H), 1.74 (dtt, *J* = 15.7, 9.4, 3.2 Hz, 1H).

$^{13}C\{^{1}H\}$ NMR (100.67 MHz, $CDCl_3$) δ: 158.5 (s), 148.4 (s), 148.2 (s), 144.7 (s), 138.1 (s), 136.2 (s), 136.2 (s), 134.2 (s), 132.8 (s), 131.1 (s), 129.4 (s), 128.8 (s), 128.2 (s), 127.6 (s), 127.4 (s), 124.7 (s), 123.6 (s), 59.1 (s), 56.3 (s), 54.5 (s), 47.2 (s), 38.7 (s), 33.4 (s), 32.4 (s), 24.9 (s).

HRMS (ESI-TOF, CH_3CN) m/z: $[M + H]^+$ calculated for $C_{27}H_{29}N_4O$ 425.2336, found 425.2325.

2-(4-(3-(2-(Pyridin-3-yl)-10H-phenothiazin-10-yl)propyl)piperazin-1-yl)ethan-1-ol (***44***)

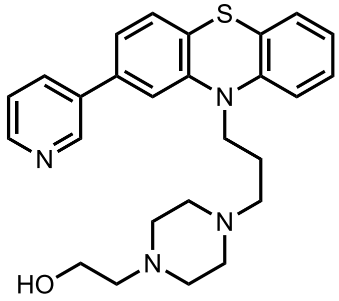

44 from perphenazine

Method A: Using Ni loading (1 mol%) with ProPhos* **7**, perphenazine (40.4 mg, 0.100 mmol, 1.0 equiv) and pyridin-3-ylboronic acid (18.4 mg, 0.150 mmol, 1.5 equiv) were used, purified by flash chromatography (DCM:MeOH), to give a white solid (42.0 mg, 0.0940 mmol, 94%).

^{1}H NMR (400.30 MHz, DMSO-*d*$_6$) δ: 8.87 (d, *J* = 2.4 Hz, 1H), 8.55 (dd, *J* = 4.7, 1.6 Hz, 1H), 8.04 (dt, *J* = 8.0, 2.0 Hz, 1H), 7.46 (dd, *J* = 8.0, 4.8 Hz, 1H), 7.27 – 7.16 (m, 4H), 7.13 (dd, *J* = 7.6, 1.5 Hz, 1H), 7.04 (dd, *J* = 8.3, 1.1 Hz, 1H), 6.93 (td, *J* = 7.5, 1.1 Hz, 1H), 4.01 (t, *J* = 6.7 Hz, 2H), 3.42 (t, *J* = 6.3 Hz, 2H), 2.47 – 2.11 (m, 12H), 1.79 (dt, *J* = 13.8, 6.7 Hz, 2H).

^{13}C{^{1}H} NMR (100.67 MHz, CDCl$_3$) δ: 148.6 (s), 147.6 (s), 145.5 (s), 144.5 (s), 136.7 (s), 135.4 (s), 134.2 (s), 127.7 (s), 127.6 (s), 127.2 (s), 123.9 (s), 123.3 (s), 122.6 (s), 121.0 (s), 116.1 (s), 114.3 (s), 60.3 (s), 58.4 (s), 54.9 (s), 53.2 (s), 52.9 (s), 44.6 (s), 23.9 (s).

HRMS (ESI-TOF, CH$_3$CN) m/z: [M + Na]$^+$ calculated for $C_{26}H_{30}N_4OSNa$ 469.2033, found 469.2048.

2-(5-Methoxy-2-methyl-1-(4-(pyridin-3-yl)benzoyl)-1H-indol-3-yl)acetic acid (***45***)

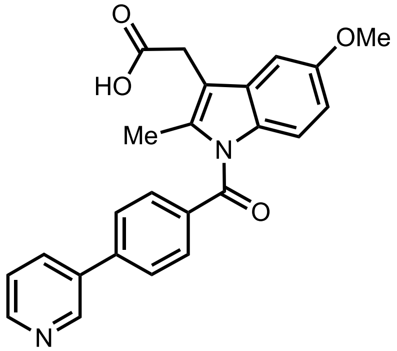

45 from indomethacin

Method A: Using Ni loading (1 mol%) with ProPhos* **7**, indomethacin (35.8 mg, 0.100 mmol, 1.0 equiv) and pyridin-3-ylboronic acid (18.4 mg, 0.150 mmol, 1.5 equiv) were used, purified by flash chromatography (DCM:MeOH), to give a white solid (35.0 mg, 0.0874 mmol, 87%).

^{1}H NMR (400.30 MHz, $CDCl_3$) δ: 8.89 (s, 1H), 8.62 (s, 1H), 7.94 (d, *J* = 7.9 Hz, 1H), 7.75 (d, *J* = 7.8 Hz, 2H), 7.63 (d, *J* = 7.8 Hz, 2H), 7.41 (t, *J* = 6.2 Hz, 1H), 6.93 (s, 1H), 6.88 (d, *J* = 9.0 Hz, 1H), 6.58 (d, *J* = 9.3 Hz, 1H), 4.86 (br s, 1H, COOH with H_2O), 3.72 (s, 3H, OMe), 3.61 (s, 2H, CH_2), 2.31 (s, 3H, Me).

$^{13}C\{^1H\}$ NMR (100.67 MHz, $CDCl_3$) δ: 168.8 (s), 155.9 (s), 148.5 (s), 147.6 (s), 141.6 (s), 135.9 (s), 135.5 (s), 135.3 (s), 135.2 (s), 130.9 (s), 130.9 (s), 130.6 (s), 127.3 (s), 124.0 (s), 114.9 (s), 113.2 (s), 111.3 (s), 101.6 (s), 55.7 (s), 13.4 (s). Some quaternary carbons were not observed.

HRMS (ESI-TOF, CH_3CN) m/z: $[M + 2NH_4]^{2+}$ calculated for $C_{24}H_{28}N_4O_4$ 218.1050, found 218.1068.

*<u>(S)-N-((2-Oxo-3-(4-(3-oxomorpholino)phenyl)oxazolidin-5-yl)methyl)-5-(pyridin-3-yl)thiophene-2-carboxamide</u> (**46**)*

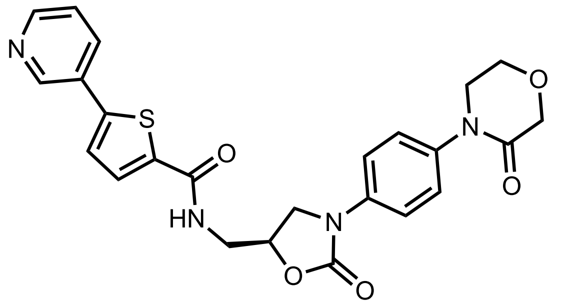

46 from rivaroxaban

Method A: Using Ni loading (1 mol%) with ProPhos* **7**, rivaroxaban (43.6 mg, 0.100 mmol, 1.0 equiv) and pyridin-3-ylboronic acid (18.4 mg, 0.150 mmol, 1.5 equiv) were used, purified by flash chromatography (DCM:MeOH), to give a white solid (39.5 mg, 0.0825 mmol, 83%).

^{1}H NMR (400.30 MHz, DMSO-d_6) δ: 8.98 (s, 1H), 8.95 (d, *J* = 2.4 Hz, 1H), 8.56 (dd, *J* = 4.8, 1.5 Hz, 1H), 8.10 (dt, *J* = 8.1, 1.9 Hz, 1H), 7.85 (d, *J* = 3.9 Hz, 1H), 7.67 (d, *J* = 3.9 Hz, 1H), 7.60 – 7.54 (m, 2H), 7.48 (dd, *J* = 8.0, 4.8 Hz, 1H), 7.44 – 7.37 (m, 2H), 4.87 (dq, *J* = 8.8, 5.6 Hz, 1H), 4.20 (d, *J* = 8.4 Hz, 3H), 3.96 (dd, *J* = 6.1, 4.1 Hz, 2H), 3.88 (dd, *J* = 9.1, 6.0 Hz, 1H), 3.70 (dd, *J* = 6.0, 4.1 Hz, 2H), 3.67 – 3.61 (m, 2H).

$^{13}C\{^1H\}$ NMR (100.67 MHz, DMSO-d_6) δ: 166.0 (s), 161.5 (s), 154.1 (s), 149.3 (s), 146.4 (s), 144.0 (s), 139.5 (s), 137.1 (s), 136.5 (s), 133.1 (s), 129.6 (s), 129.1 (s), 126.0 (s), 125.7 (s), 124.1 (s), 118.4 (s), 71.4 (s), 67.7 (s), 63.5 (s), 49.0 (s), 47.5 (s), 42.3 (s).

HRMS (ESI-TOF, CH_3CN) m/z: $[M + H]^+$ calculated for $C_{24}H_{23}N_4O_5S$ 479.1384, found 479.1397.

*3-(Benzofuran-5-yl)-4-methylbenzoic acid (**47**)*

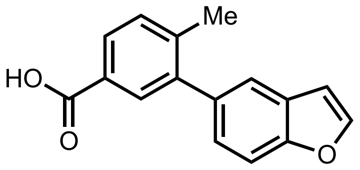

47

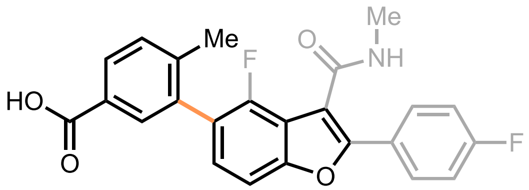

BMS-929075
HCV NS5B replicase inhibitor

Method A: Using Ni loading (0.1 mol%) with ProPhos* **7**, 5-bromobenzofuran (25.1 μL, 0.200 mmol, 1.0 equiv) and 4-methyl-3-(4,4,5,5-tetramethyl-1,3,2-dioxaborolan-2-yl)benzoic acid (78.6 mg, 0.30 mmol, 1.5 equiv) were used, purified by flash chromatography (MeOH:DCM), to give a white solid (mixed with unreacted boronic ester). The mixture was further purified by HPLC (35.8 mg, 0.142 mmol, 71%).

^1H NMR (400.30 MHz, $CDCl_3$) δ: 8.06 – 7.97 (m, 2H), 7.68 (d, J = 2.2 Hz, 1H), 7.59 – 7.53 (m, 2H), 7.38 (dt, J = 7.8, 0.7 Hz, 1H), 7.26 – 7.23 (m, 1H), 6.81 (dd, J = 2.2, 1.0 Hz, 1H), 2.35 (s, 3H, Me). Although the proton of -CO_2H was not detected, we noticed that a broad signal around 4.66 ppm, which could be due to the H_2O affected by the CO_2H.

$^{13}C\{^1H\}$ NMR (100.67 MHz, $CDCl_3$) δ: 171.3 (s), 154.4 (s), 145.8 (s), 142.6 (s), 142.5 (s), 135.7 (s), 132.0 (s), 130.7 (s), 129.0 (s), 127.6 (s), 126.9 (s), 125.7 (s), 121.8 (s), 111.2 (s), 106.8 (s), 21.1 (s, Me).

HRMS (ESI-TOF, CH_3CN) m/z: $[M + H]^+$ calculated for $C_{16}H_{13}O_3$ 253.0859, found 253.0856.

(1R)-2,2,2-Trifluoro-1-(3'-methoxybiphenyl-4-yl)ethanol (***48***)

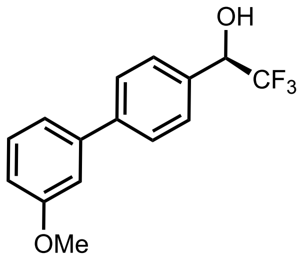

48

Method A: Using Ni loading (1 mol%) with ProPhos* **7**, (*R*)-1-(4-bromophenyl)-2,2,2-trifluoroethanol (51.0 mg, 0.200 mmol, 1.0 equiv) and (3-methoxyphenyl)boronic acid (45.6 mg, 0.300 mmol, 1.5 equiv) were used, purified by flash chromatography (EtOAc:hexane), to give a white solid (39.4 mg, 0.140 mmol, 70%). The NMR data are consistent with the literature report.[22]

^{1}H NMR (500.20 MHz, $CDCl_3$) δ: 7.63 (d, *J* = 8.2 Hz, 2H), 7.55 (d, *J* = 8.0 Hz, 2H), 7.37 (t, *J* = 7.9 Hz, 1H), 7.18 (dt, *J* = 7.7, 1.2 Hz, 1H), 7.12 (t, *J* = 2.1 Hz, 1H), 6.93 (dd, *J* = 8.2, 2.5 Hz, 1H), 5.08 (q, *J* = 6.8 Hz, 1H, CH), 3.87 (s, 3H, OMe), 2.63 (s, 1H, OH).

^{19}F{^{1}H} NMR (470.61 MHz, $CDCl_3$) δ: -78.25 (s).

^{13}C{^{1}H} NMR (125.79 MHz, $CDCl_3$) δ: 160.1 (s), 142.5 (s), 142.0 (s), 133.1 (s), 130.0 (s), 128.0 (s), 127.6 (s), 125.5 (s), 123.3 (s), 119.8 (s), 113.2 (s), 113.1 (s), 72.8 (q, *J* = 32 Hz), 55.5 (s, OMe).

4-(4-Methylphenyl)-2-pyrimidinamine (***49***)

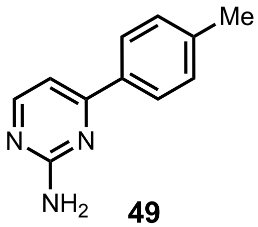

49

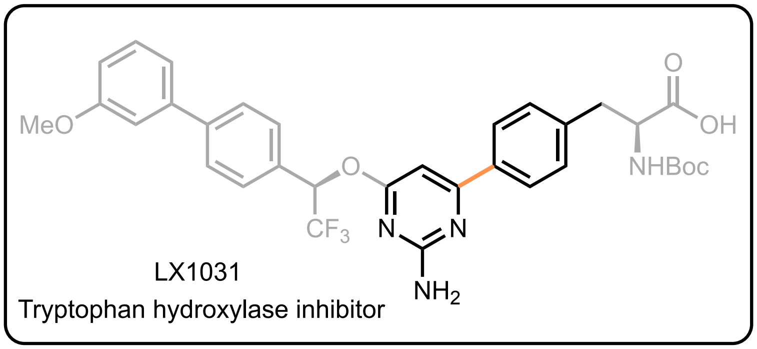

Method A: Ni loading (0.1 mol%) with ProPhos* **7**, 4-chloropyrimidin-2-amine (25.9 mg, 0.200 mmol, 1.0 equiv) and *p*-tolylboronic acid (40.8 mg, 0.300 mmol, 1.5 equiv) were used, purified by flash chromatography (EtOAc:hexane), to give a white solid (34.2 mg, 0.185 mmol, 92%). This is a known compound and NMR data are consistent with the literature report.[23]

^{1}H NMR (400.30 MHz, CDCl$_3$) δ: 8.32 (d, *J* = 5.3 Hz, 1H), 7.90 (d, *J* = 8.3 Hz, 2H), 7.27 (d, *J* = 8.1 Hz, 2H), 7.02 (d, *J* = 5.3 Hz, 1H), 5.14 (s, 2H, NH$_2$), 2.41 (s, 3H, Me).

^{13}C{^{1}H} NMR (100.67 MHz, CDCl$_3$) δ: 165.6 (s), 163.3 (s), 158.7 (s), 141.1 (s), 134.5 (s), 129.7 (s), 127.1 (s), 107.6 (s), 21.6 (s, Me).

5-Fluoro-2-(2-methoxyphenyl)pyrimidine ***(50)***

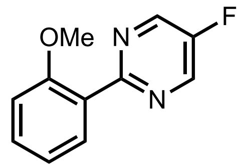

50

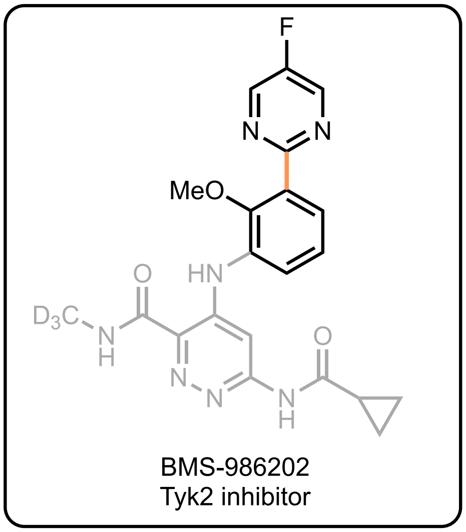

BMS-986202
Tyk2 inhibitor

Method A: Ni loading (0.1 mol%) with ProPhos* **7**, 2-chloro-5-fluoropyrimidine (18.4 µL, 0.200 mmol, 1.0 equiv) and (2-methoxyphenyl)boronic acid (45.6 mg, 0.300 mmol, 1.5 equiv) were used, purified by flash chromatography (EtOAc:hexane), to give a white solid (37.1 mg, 0.182 mmol, 91%).

^{1}H NMR (400.30 MHz, CDCl$_3$) δ: 8.71 (s, 2H), 7.68 (dd, *J* = 7.6, 1.8 Hz, 1H), 7.51 – 7.38 (m, 1H), 7.14 – 6.96 (m, 2H), 3.87 (s, 3H, OMe).

^{19}F{^{1}H} NMR (470.61 MHz, CDCl$_3$) δ: -140.68 (s).

$^{13}C\{^1H\}$ NMR (100.67 MHz, $CDCl_3$) δ: 162.2 (d, *J* = 6 Hz), 157.6 (s), 155.0 (s), 144.9 (d, *J* = 20 Hz), 131.8 (s), 131.3 (s), 127.5 (d, *J* = 2 Hz), 120.8 (s), 112.1 (s), 56.2 (s, OMe).

HRMS (ESI-TOF, CH_3CN) m/z: $[M + H]^+$ calculated for $C_{11}H_{10}FN_2O$ 205.0772, found 205.0772.

<u>*3,6'-Biquinoline*</u> *(**51**)*

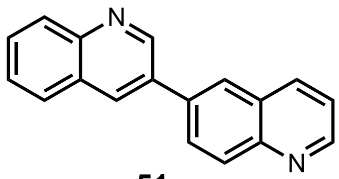

51

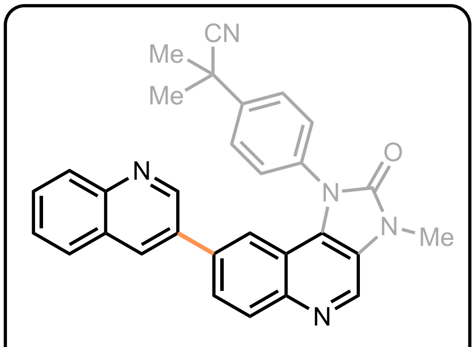

Dactolisib
PI3K inhibitor – cancer treatment

Method A: Ni loading (0.1 mol%) with ProPhos* **7**, 6-chloroisoquinoline (32.7 mg, 0.200 mmol, 1.0 equiv) and isoquinolin-4-ylboronic acid (51.9 mg, 0.300 mmol, 1.5 equiv) were used, purified by flash chromatography (EtOAc:hexane), to give a white solid (45.9 mg, 0.179 mmol, 90%). The NMR data were consistent with our previous report.[7]

^1H NMR (400.30 MHz, DMSO-d_6) δ: 9.43 (q, *J* = 2.2 Hz, 1H), 8.95 (dq, *J* = 4.0, 1.8 Hz, 1H), 8.83 (dt, *J* = 5.8, 2.9 Hz, 1H), 8.55 (dd, *J* = 5.1, 2.7 Hz, 1H), 8.51 – 8.45 (m, 1H), 8.31 (ddt, *J* = 9.7, 5.3, 2.4 Hz, 1H), 8.19 (dd, *J* = 9.0, 2.9 Hz, 1H), 8.10 (dd, *J* = 7.9, 2.8 Hz, 2H), 7.81 (ddt, *J* = 7.3, 4.6, 2.8 Hz, 1H), 7.72 – 7.65 (m, 1H), 7.61 (dt, *J* = 7.8, 3.9 Hz, 1H).

Scale-up synthesis of BMS-986363 (CC-90011) (***5***)

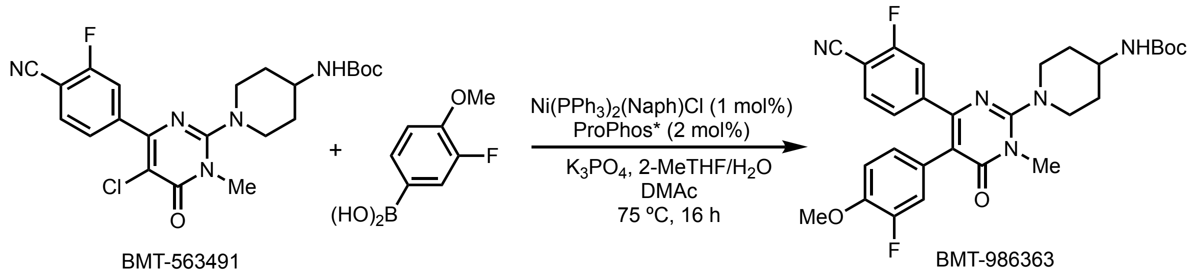

Under nitrogen flow, degassed 2-MeTHF (160 mL, 20 vol) was added to a 250 mL reactor, followed by BMT-563491 (8.00 g, 17.3 mmol, 1.00 equiv), *trans*-chloro(1-naphthyl)bis(triphenylphoshine) nickel(II) (130 mg, 0.174 mmol, 0.01 equiv), ProPhos* (100 mg, 0.367 mmol, 0.02 equiv), and (3-fluoro-4-methoxy-phenyl)boronic acid (4.16 g, 27.0 mmol, 1.41 equiv). Degassed DMAc (16 mL, 2 vol) was then added, and the solution aged for 15 min with nitrogen sparging. In a separate 100 mL Erlenmeyer flask, K_3PO_4 (9.30 g, 43.4 mmol, 2.51 equiv) was dissolved in water (28 mL, 3.5 mL/g) and degassed by nitrogen sparging for 15 min. The K_3PO_4 solution (aq.) was then added to the reactor. With stirring at 450 rpm to mix the biphasic homogeneous solution, the reactor was heated to 75 °C for 16 h, resulting in crystallization of the product as a white solid. The reaction was then cooled to room temperature, sampled and analyzed by LC, which showed high conversion (<2% starting material BMT-563491). The reaction was diluted with 2-MeTHF (20 mL, 2.5 vol), then filtered. The cake was washed with THF (20 mL, 2.5 vol) and water (20 mL, 2.5 vol), followed by drying at 50 °C overnight under vacuum with nitrogen sweep to yield the product **5** as an off-white solid (8.18 g, 14.8 mmol, 86%). The characterization data are consistent with that reported in the literature.[24]

^{1}H NMR (500.20 MHz, TFE-*d*$_3$) δ: 7.44 (dd, *J* = 8.1, 6.4 Hz, 1H, Ar), 7.31 (d, *J* = 10.0 Hz, 1H, Ar), 7.19 (d, *J* = 8.1 Hz, 1H, Ar), 7.00 – 6.91 (m, 2H, Ar), 6.83 (dd, *J* = 8.4, 2.0 Hz, 1H, Ar), 3.86, (s, 3H, Me), 3.75 – 3.60 (m, 3H, CH_2&CH), 3.56 (s, 3H, Me), 3.09 (t, *J* = 12.4 Hz, 2H, CH_2), 2.11 – 2.00 (m, 2H, CH_2), 1.72 – 1.58 (m, 2H, CH_2), 1.46 (s, 9H, *t*Bu).

^{19}F{^{1}H} NMR (470.61 MHz, TFE-*d*$_3$) δ: -109.14 (s), -137.69 (s).

^{13}C{^{1}H} NMR (125.79 MHz, TFE-*d*$_3$) δ: 168.3 (s), 164.9 (d, *J* = 258 Hz), 160 (s), 159.0 (br s), 158.7 (s), 154.2 (d, *J* = 245 Hz), 149.2 (d, *J* = 11 Hz), 148.2 (d, *J* = 8 Hz), 134.5 (s), 129.2 (d, *J* =

4 Hz), 128.4 (d, *J* = 7 Hz), 127.9 (d, *J* = 3 Hz), 120.1 (d, *J* = 19 Hz), 119.2 (s), 119.1 (s), 115.6 (s), 115.4 (s), 102.1 (d, *J* = 16 Hz), 57.3 (s), 49.8 (s), 49.4 (br s), 35.3 (s), 33.0 (s), 28.9 (s).

6. NMR Spectra

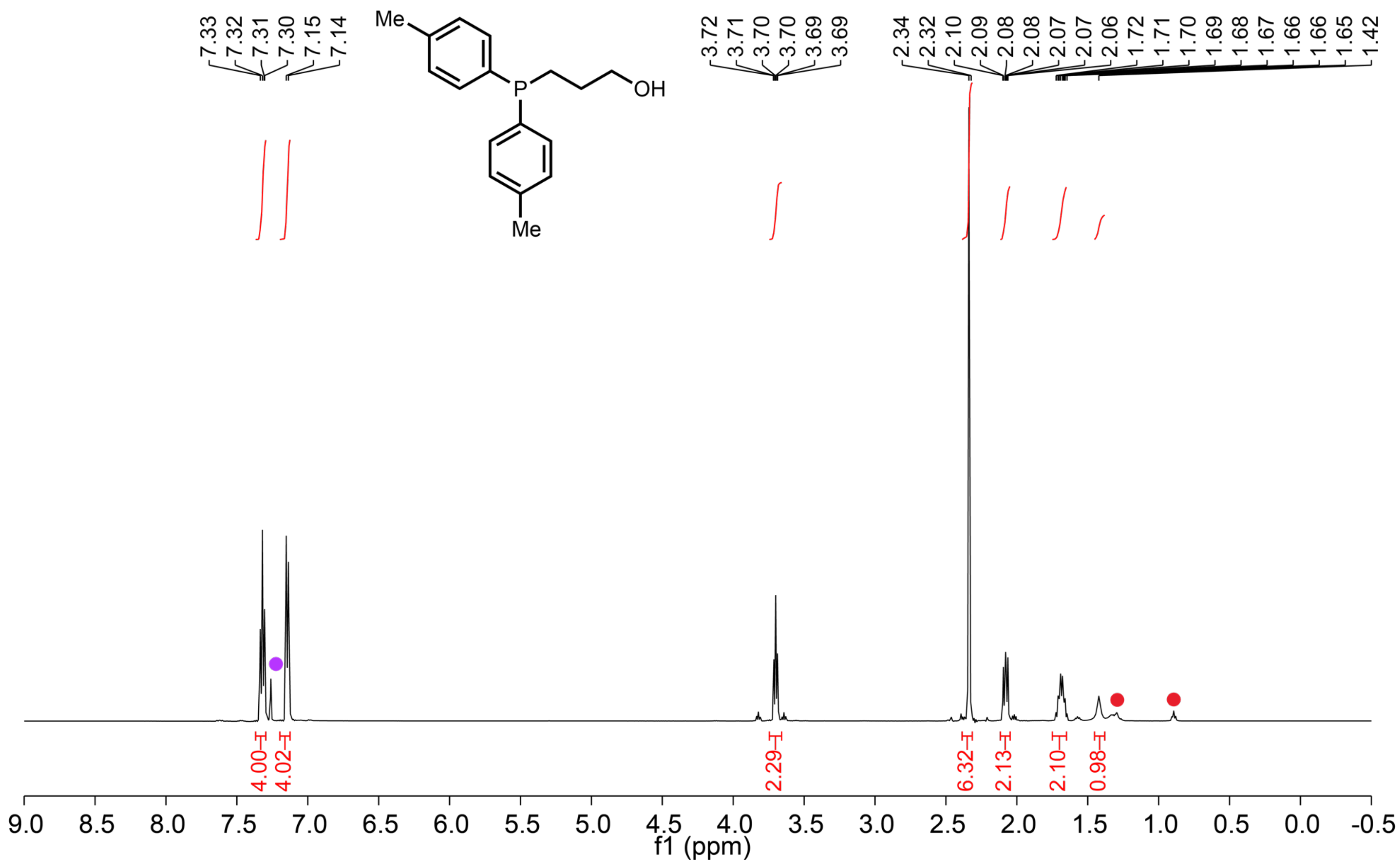

Figure S11. $^{31}P\{^{1}H\}$ NMR (202.47 MHz, $CDCl_3$) spectrum of ProPhos* **7**.

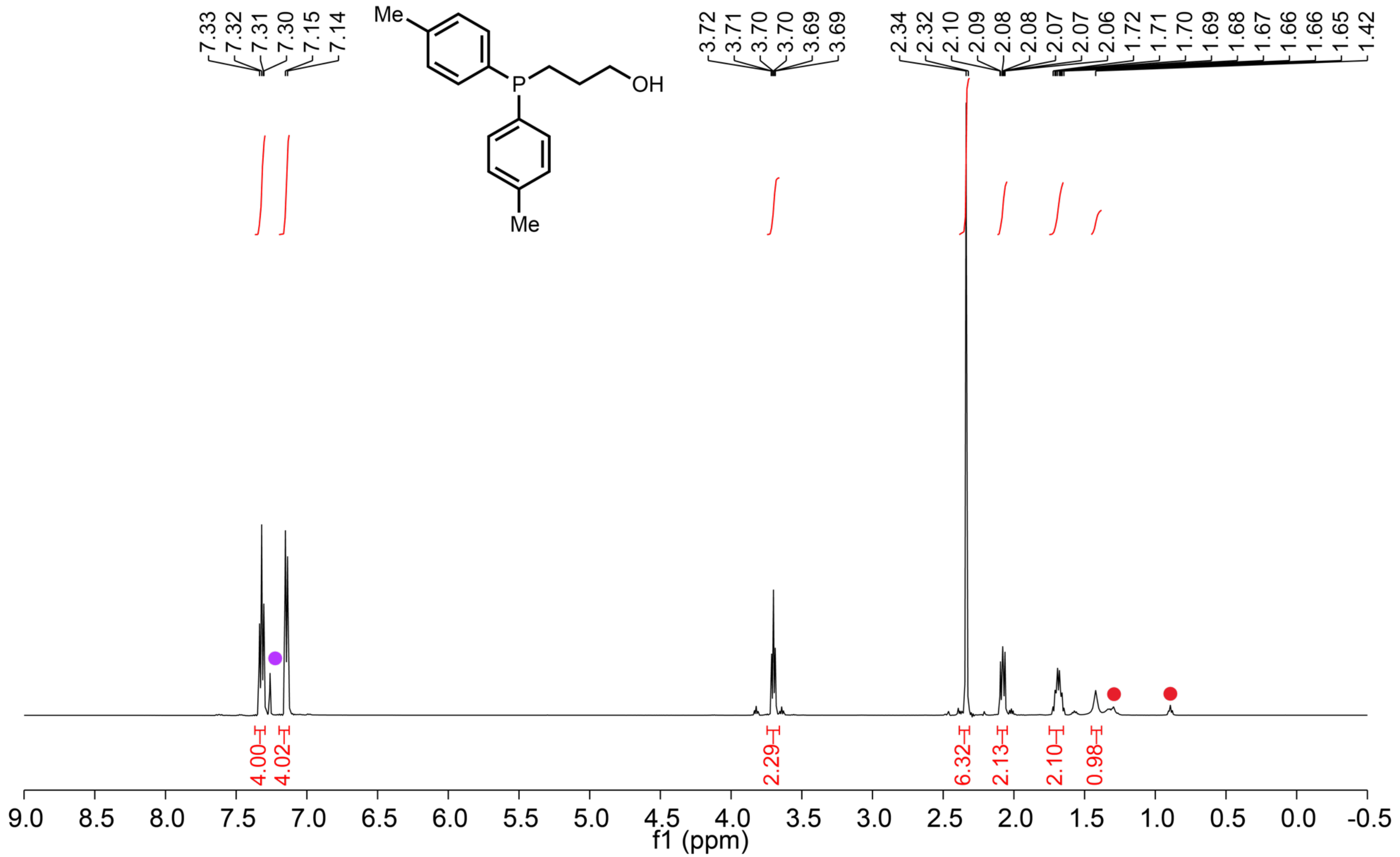

Figure S12. ^{1}H NMR (500.20 MHz, $CDCl_3$) spectrum of ProPhos* **7**. Residual proteo-solvent (•) and hexane (•), other trace impurities are due to the phosphine oxide and unreacted $ClCH_2CH_2CH_2OH$. The purity of this product phosphine is >95%.

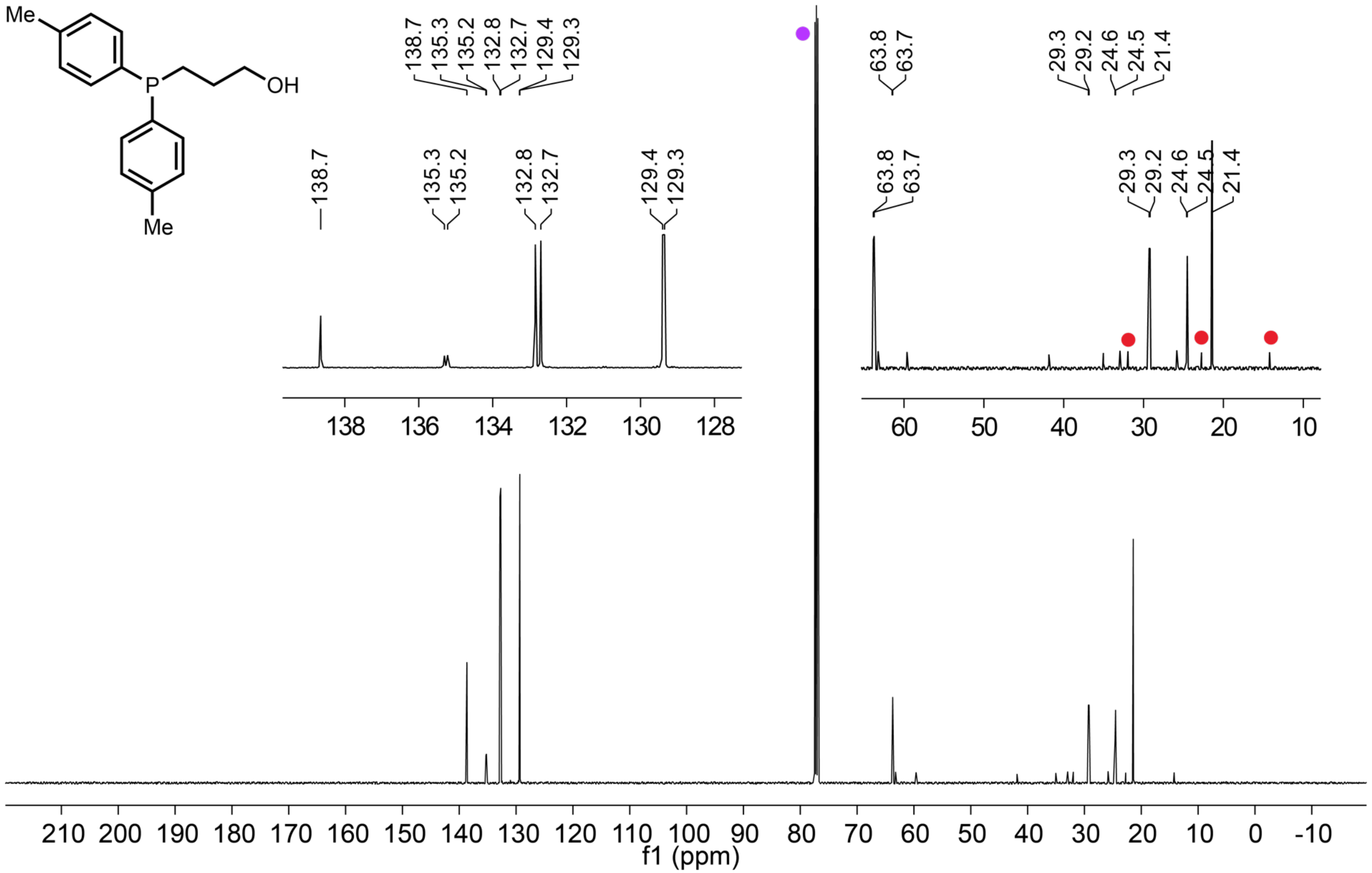

Figure S13. $^{13}C\{^1H\}$ NMR (125.79 MHz, $CDCl_3$) spectrum of ProPhos*, **7**. Hexane (●), other trace impurities are due to the phosphine oxide and unreacted $ClCH_2CH_2CH_2OH$. Deuterated solvent (●)

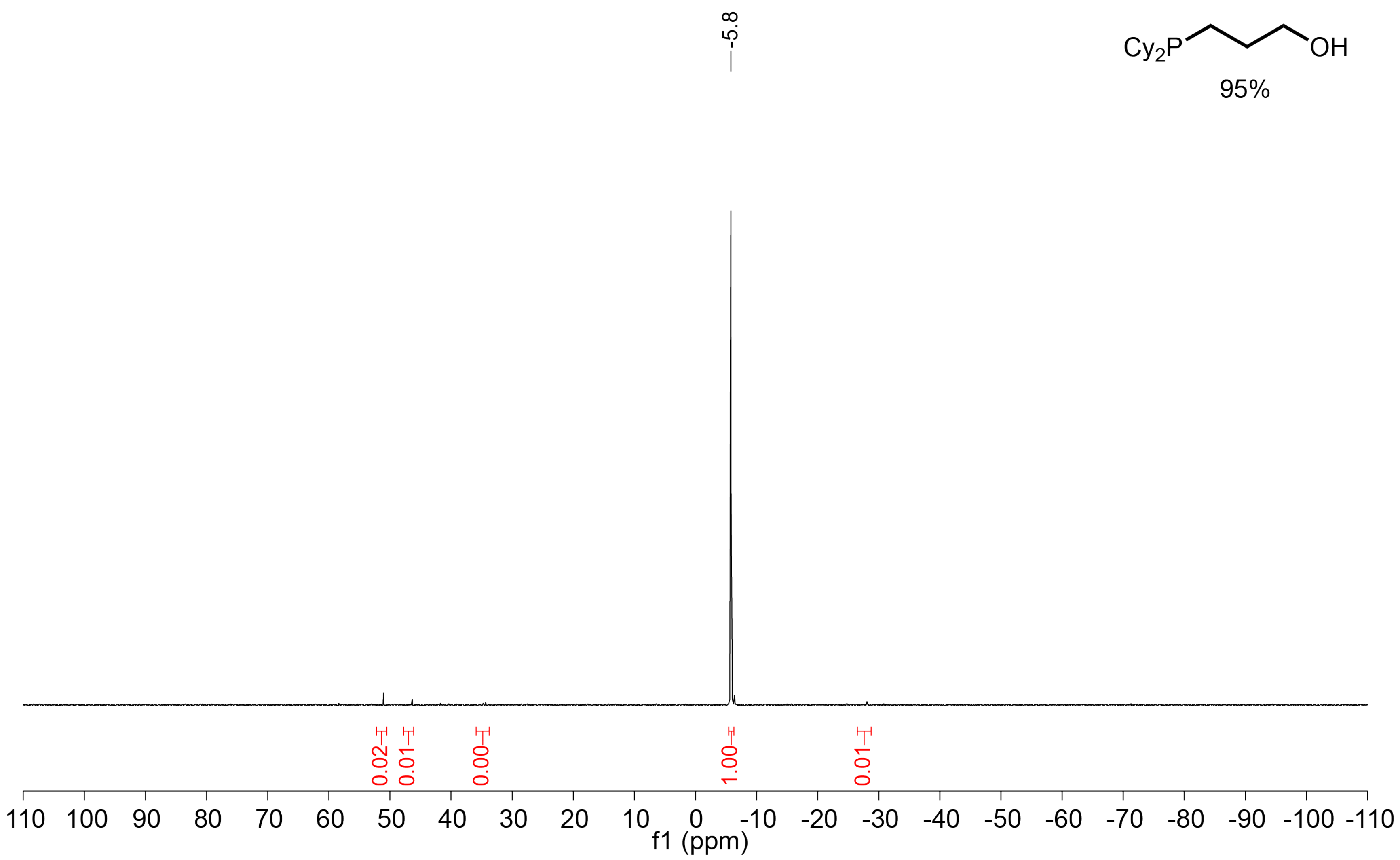

Figure S14. $^{31}P\{^1H\}$ NMR (202.47 MHz, C_6D_6) spectrum of ^{Cy}ProPhos **8**.

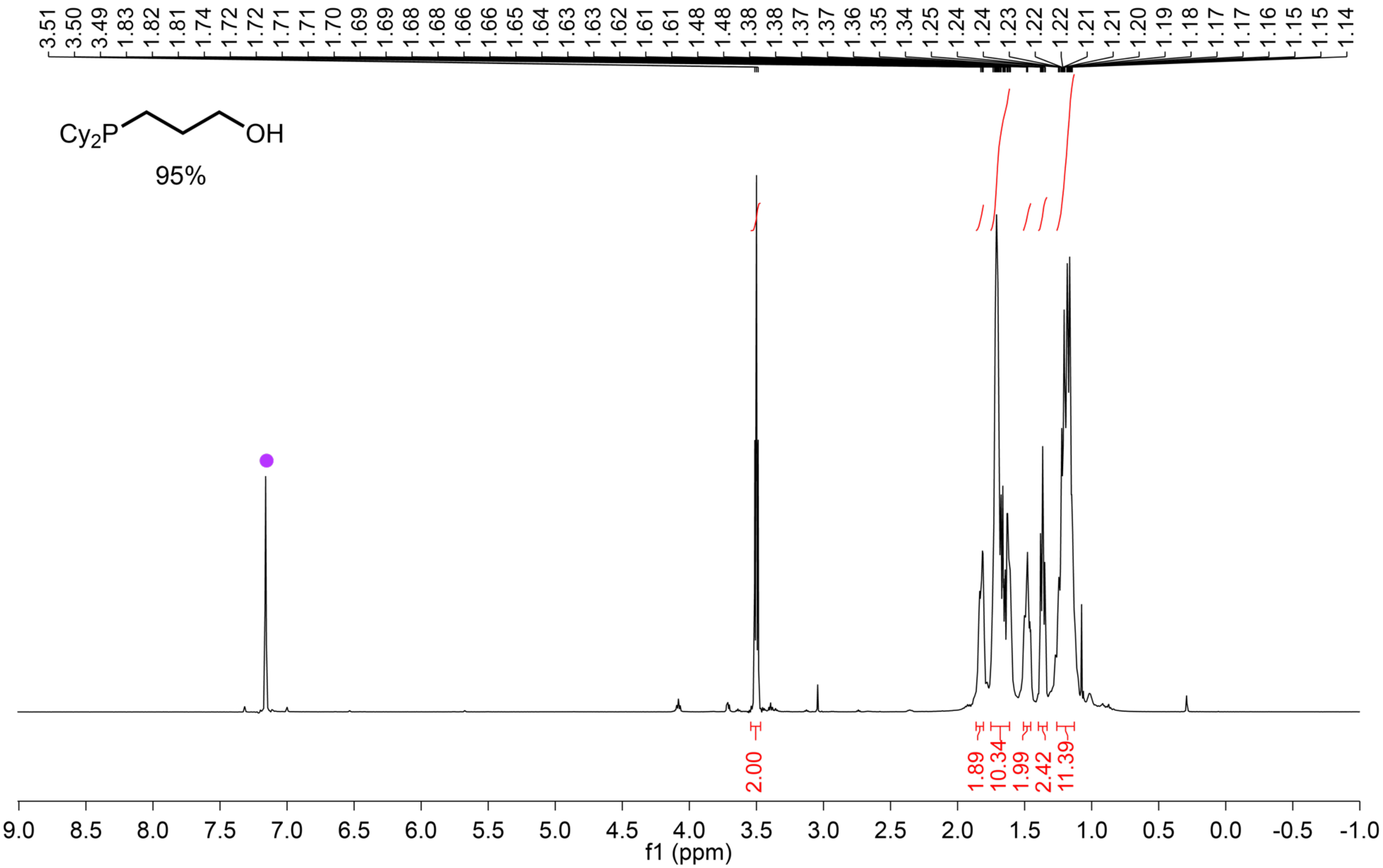

Figure S15. ^{1}H NMR (500.20 MHz, C_6D_6) spectrum of [Cy]ProPhos **8**. Residual proteo-solvent (●).

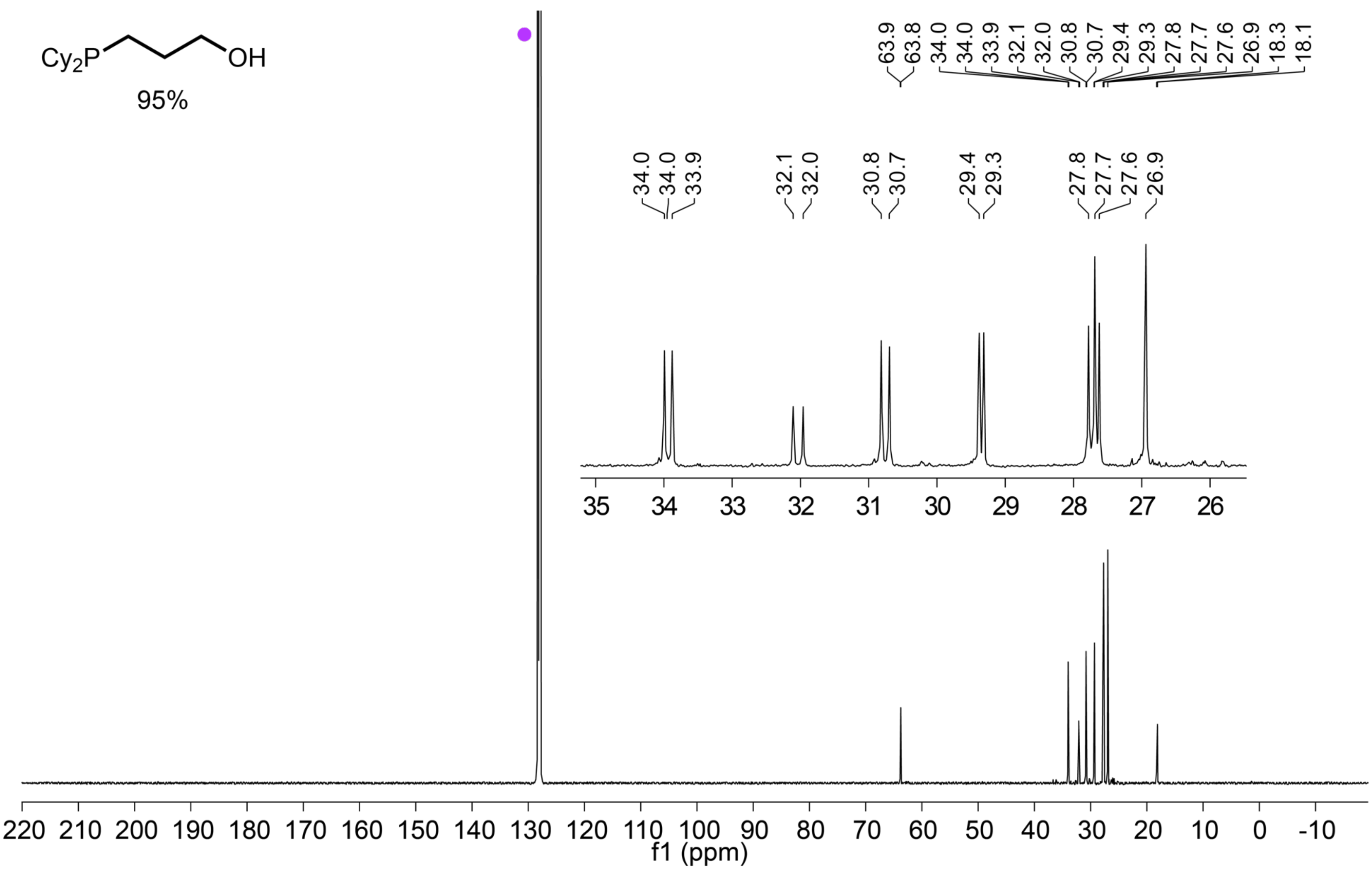

Figure S16. $^{13}C\{^{1}H\}$ NMR (125.79 MHz, C_6D_6) spectrum of ^{Cy}ProPhos **8**. Deuterated solvent (•)

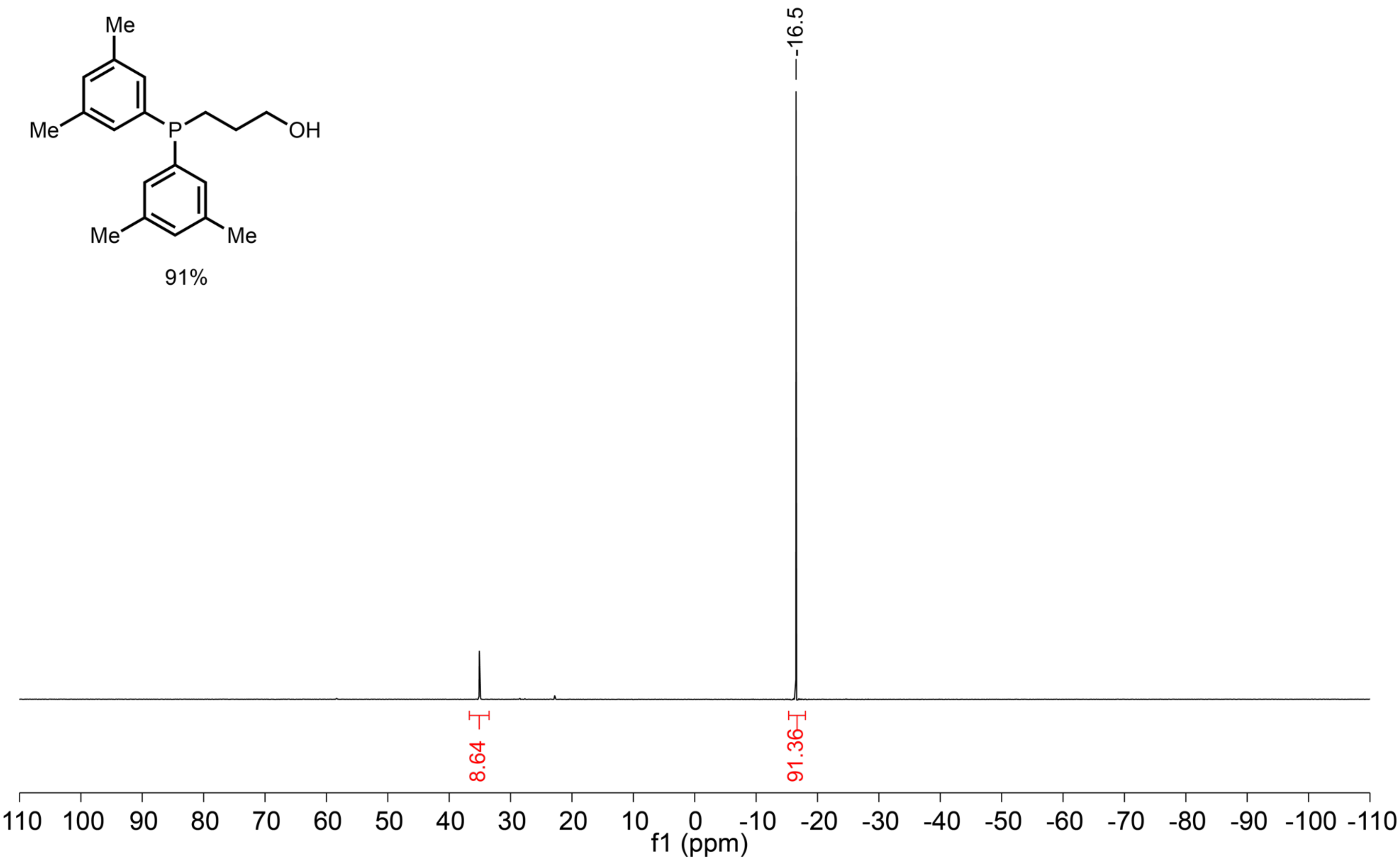

Figure S17. $^{31}P\{^1H\}$ NMR (202.47 MHz, $CDCl_3$) spectrum of $^{di\text{-}Me}$ProPhos* **9**. Signal at ~35 ppm is due to the phosphine oxide (~9%).

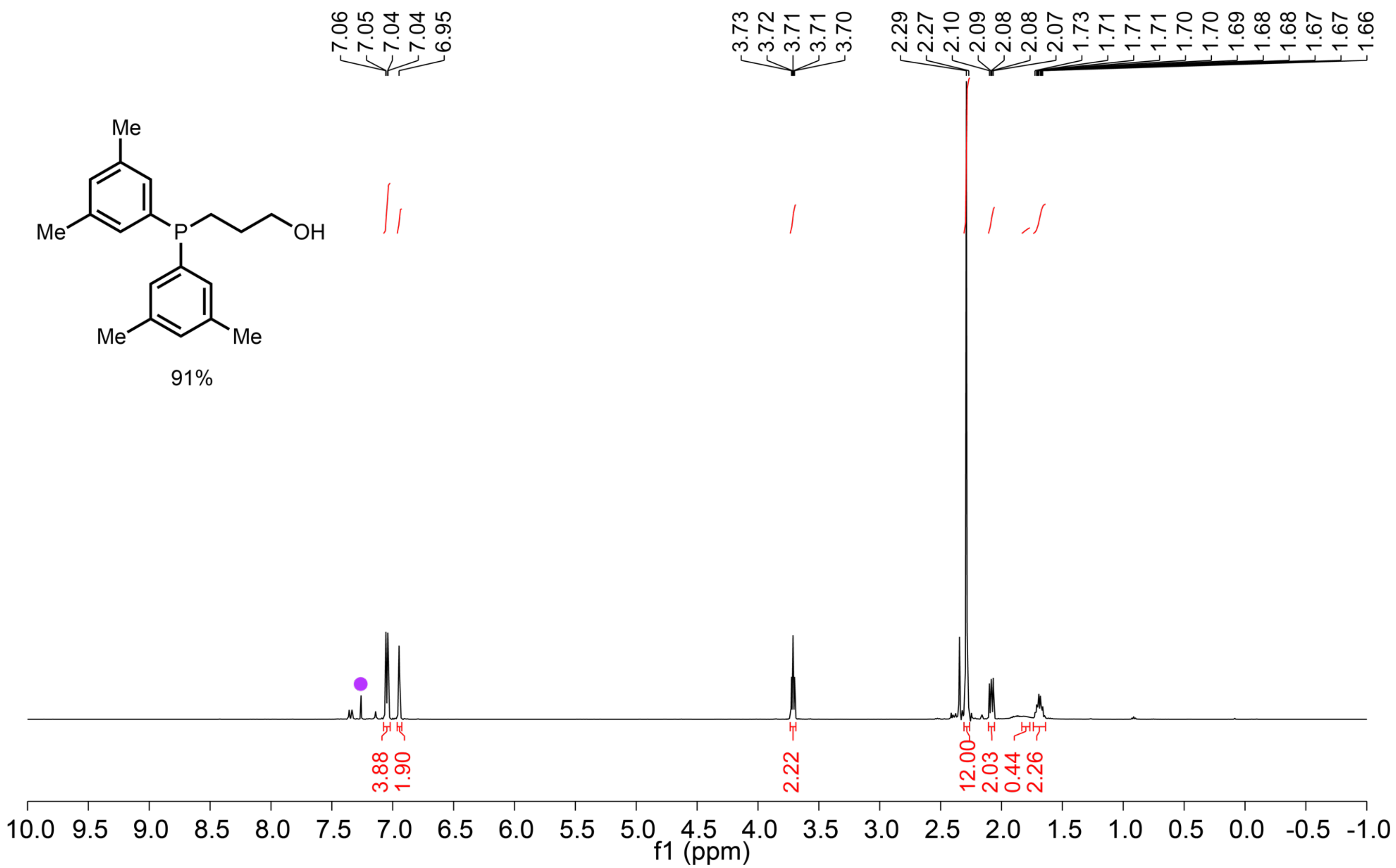

Figure S18. ^{1}H NMR (500.20 MHz, $CDCl_3$) spectrum of $^{\text{di-Me}}$ProPhos* **9**. Residual proteo-solvent (•).

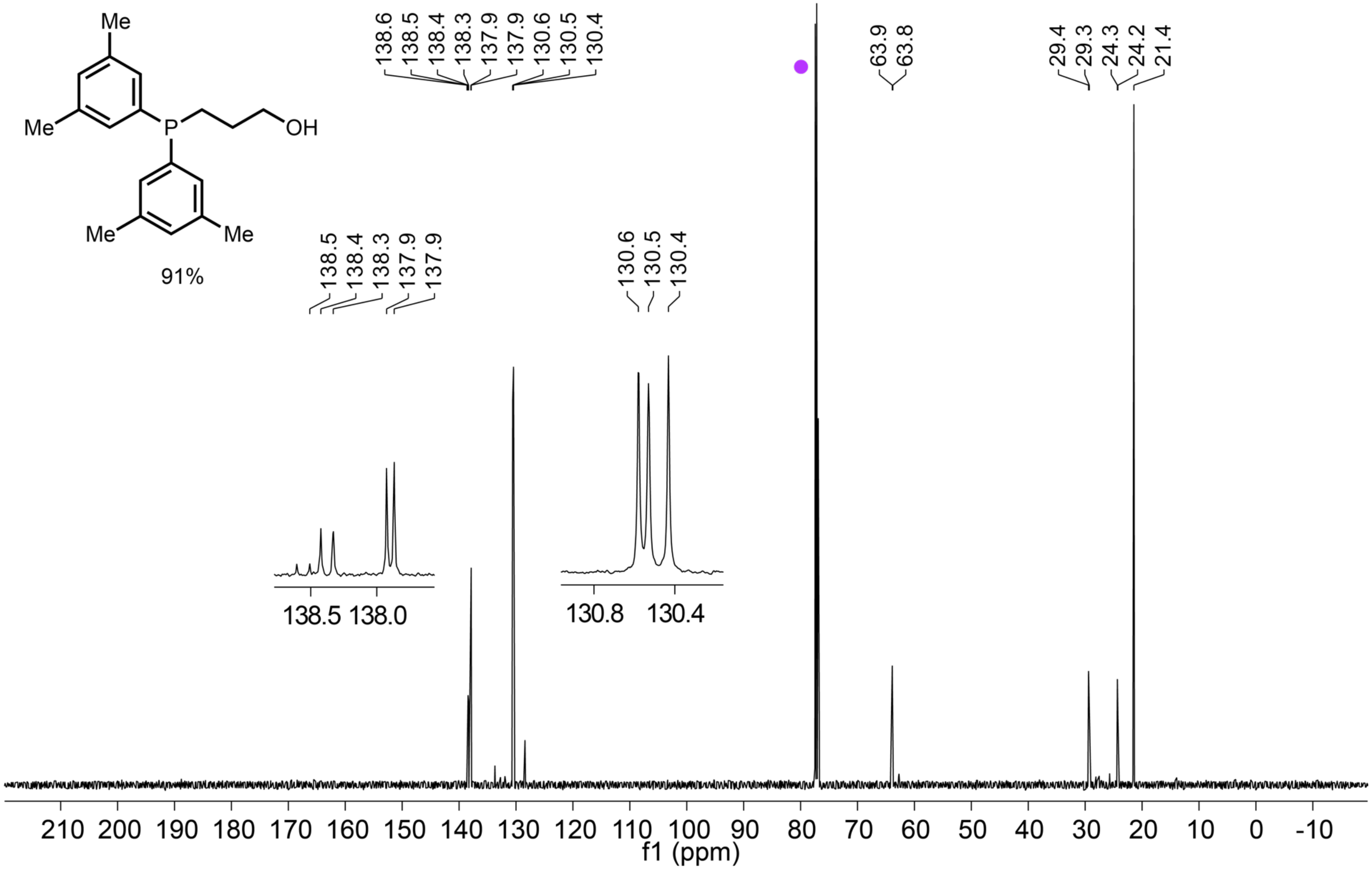

Figure S19. $^{13}C\{^{1}H\}$ NMR (125.79 MHz, $CDCl_3$) spectrum of $^{di\text{-}Me}$ProPhos* **9**. Deuterated solvent (●)

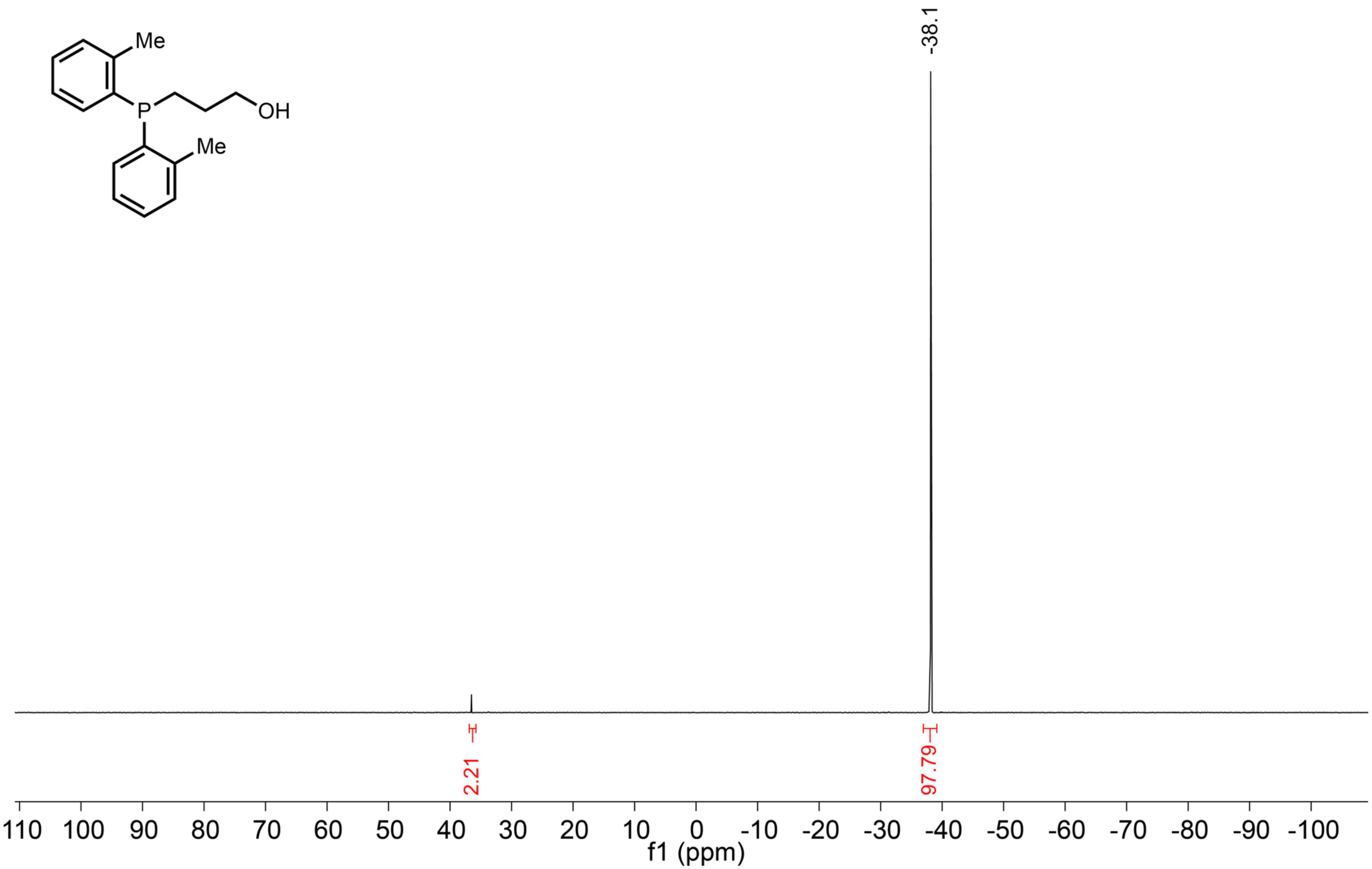

Figure S20. $^{31}P\{^{1}H\}$ NMR (202.47 MHz, $CDCl_3$) spectrum of $^{o\text{-Me}}$ProPhos* **10**. Signal at ~36 ppm is due to the phosphine oxide (~2%).

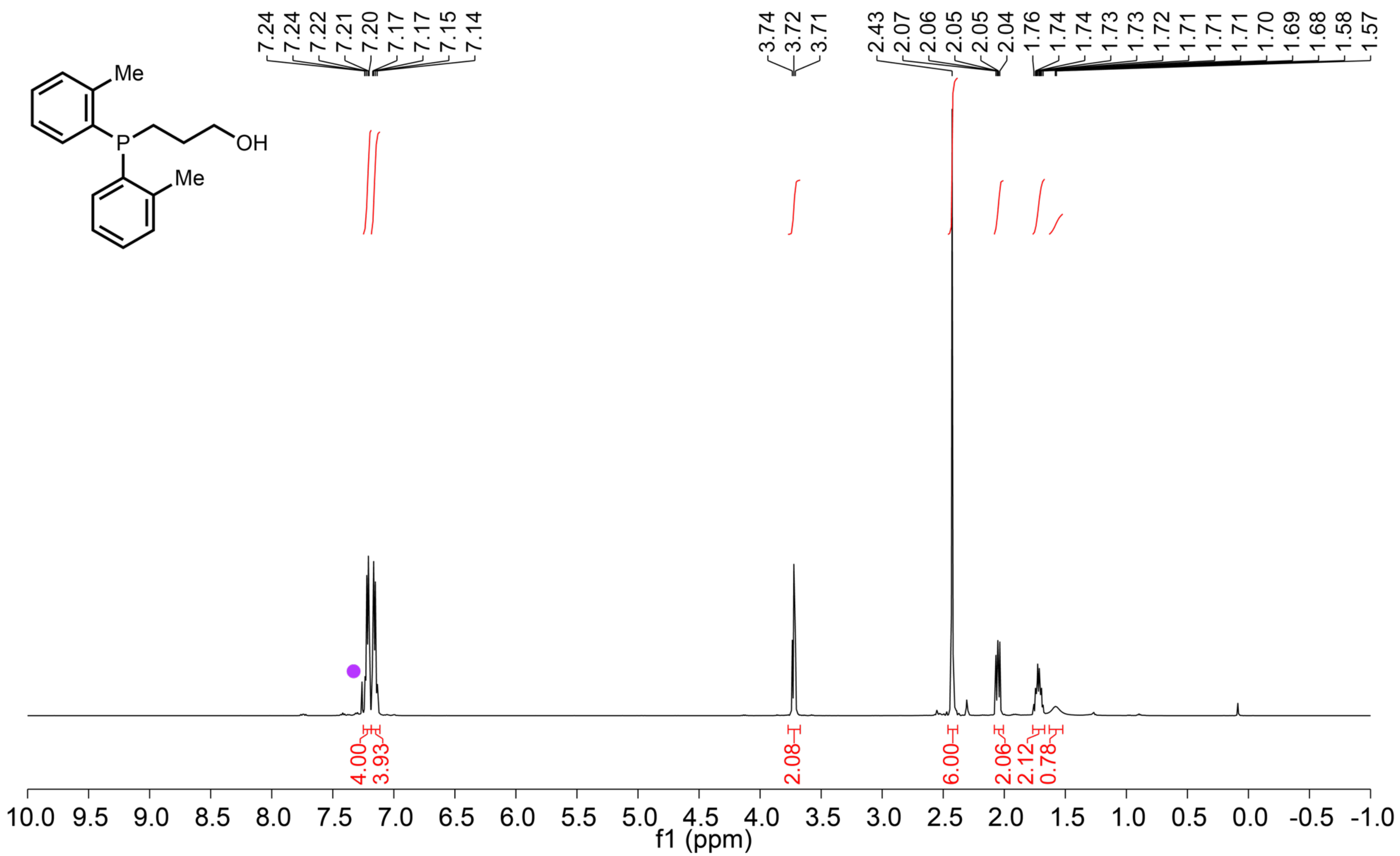

Figure S21. ^{1}H NMR (500.20 MHz, $CDCl_3$) spectrum of $^{o\text{-Me}}$ProPhos* **10**. Residual proteo-solvent (●).

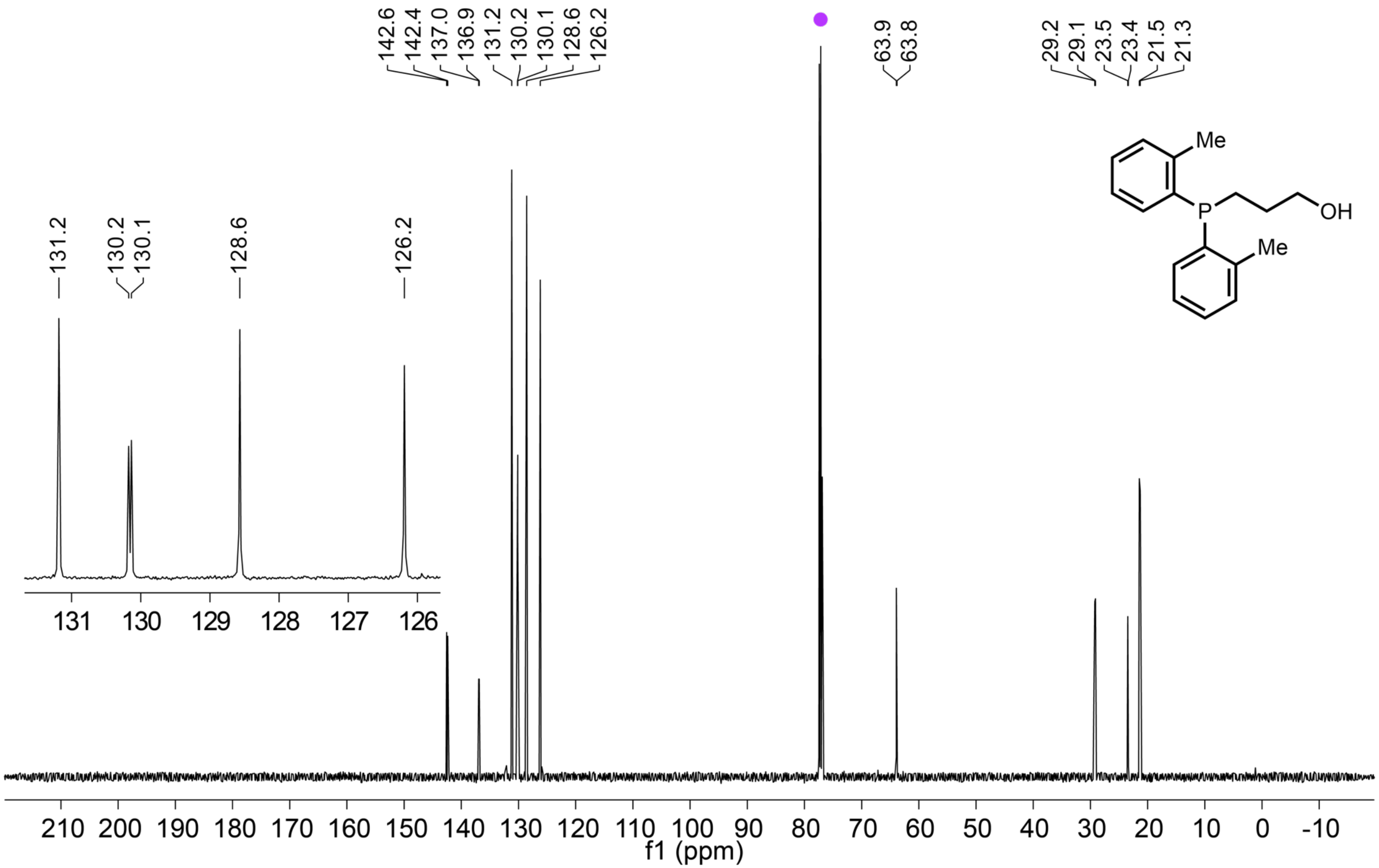

Figure S22. $^{13}C\{^1H\}$ NMR (125.79 MHz, $CDCl_3$) spectrum of $^{o\text{-}Me}$ProPhos* **10**. Deuterated solvent (•)

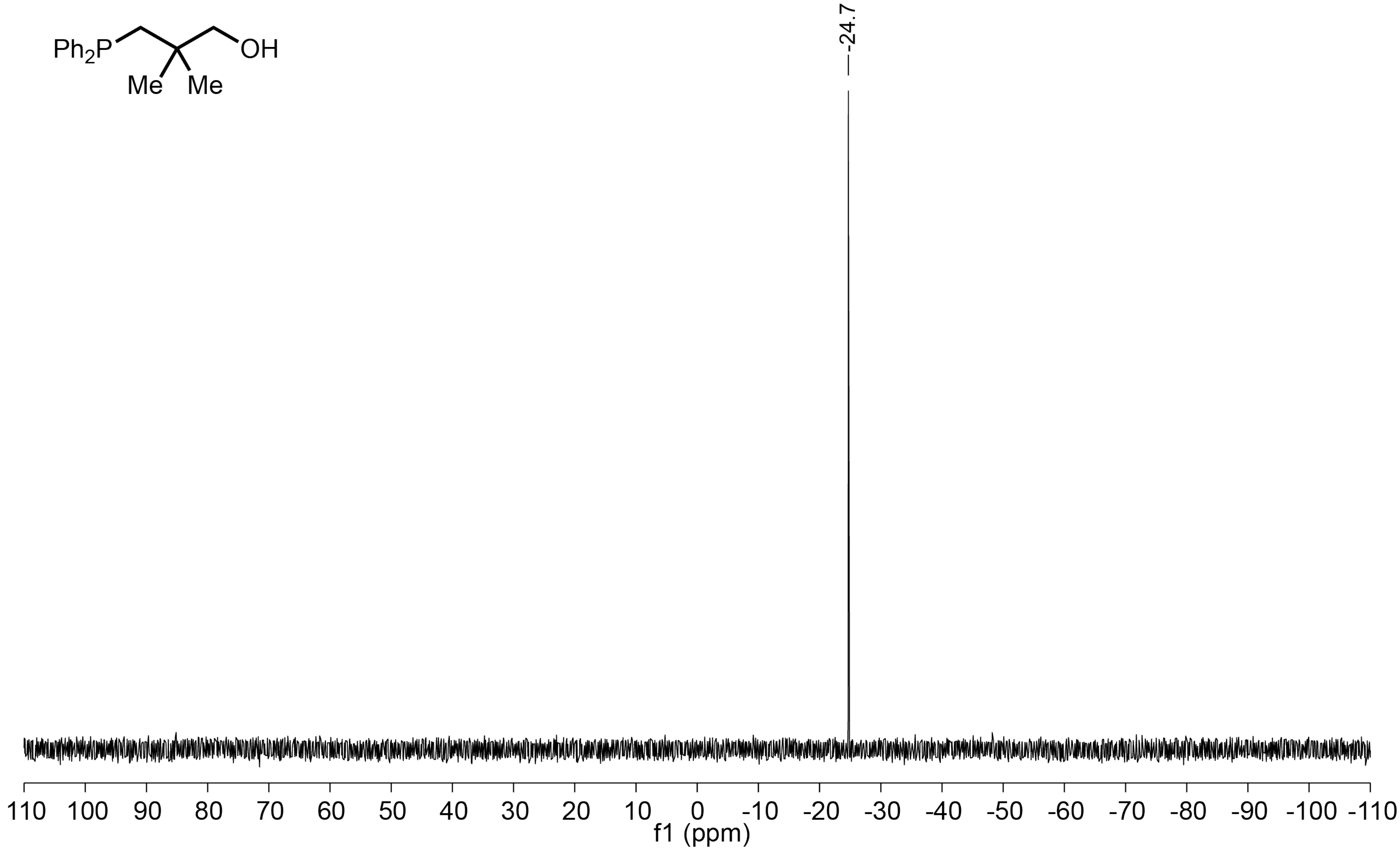

Figure S23. $^{31}P\{^1H\}$ NMR (162.04 MHz, C_6D_6) spectrum of $^{gem\text{-}Me}$ProPhos **11**.

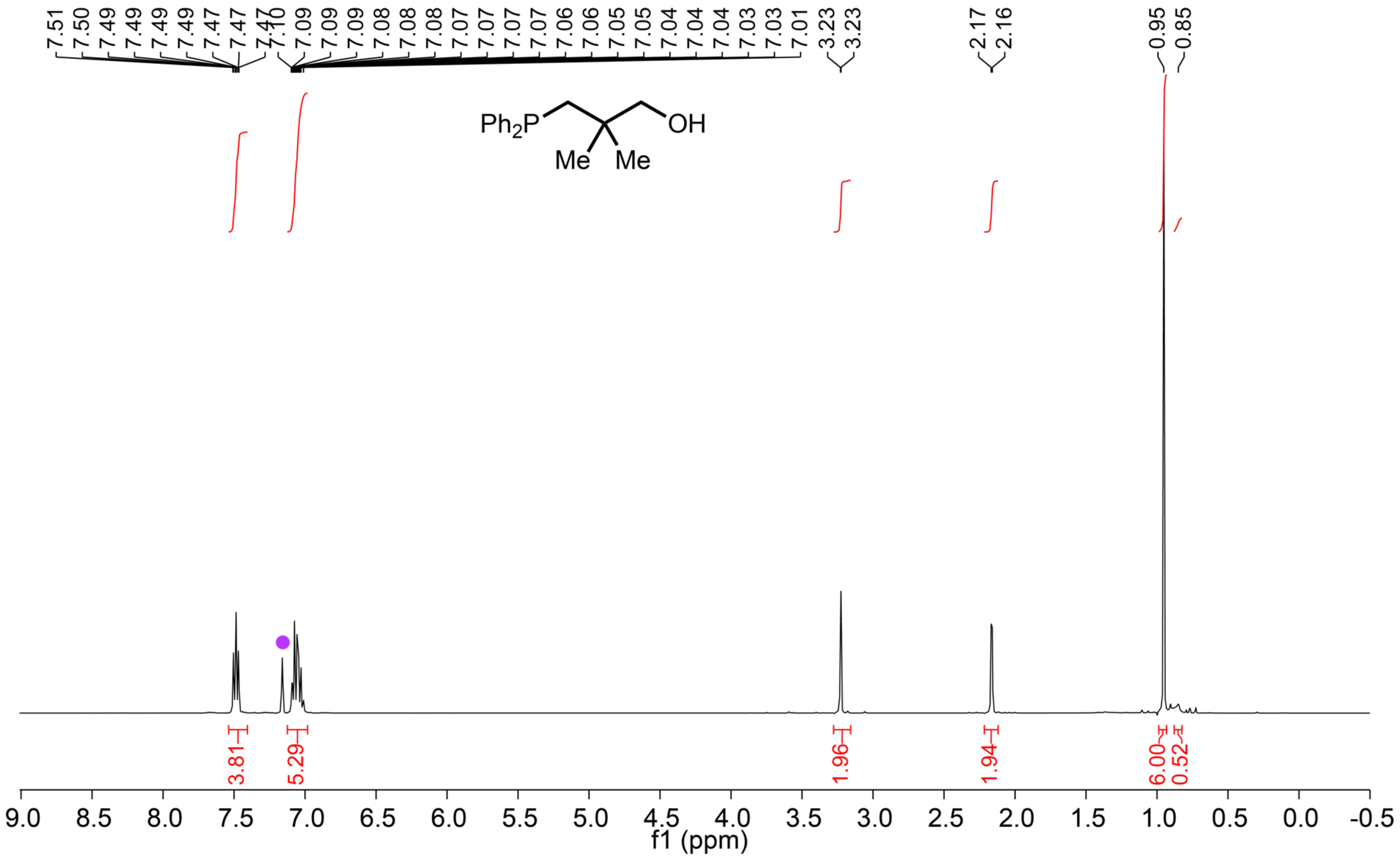

Figure S24. ^{1}H NMR (400.30 MHz, C_6D_6) spectrum of $^{gem\text{-}Me}$ProPhos **11**. Residual proteo-solvent (•).

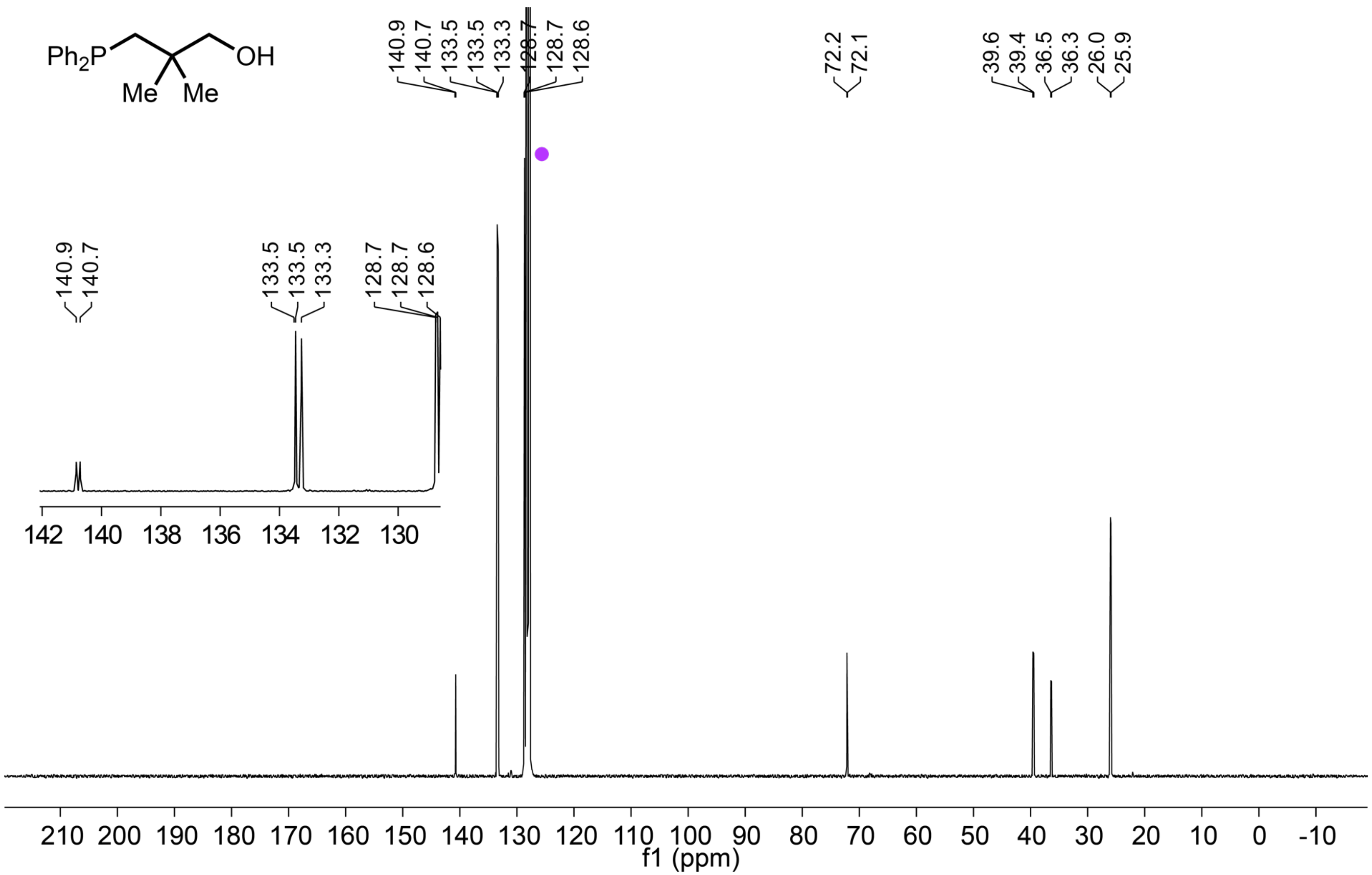

Figure S25. $^{13}C\{^1H\}$ NMR (100.67 MHz, C_6D_6) spectrum of $^{gem\text{-}Me}$ProPhos **11**. Deuterated solvent (•)

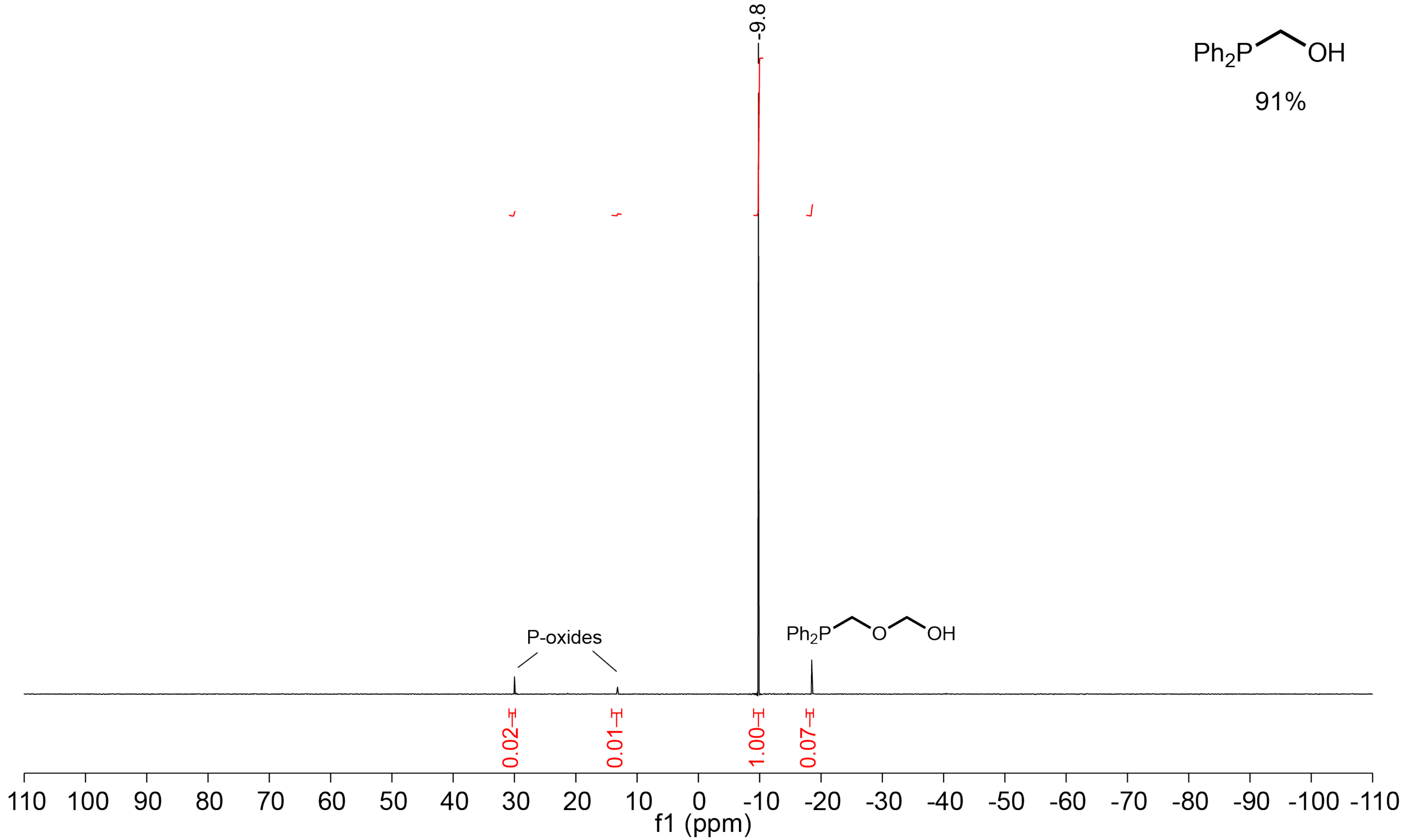

Figure S26. $^{31}P\{^{1}H\}$ NMR (162.04 MHz, $CDCl_3$) spectrum of PPh_2CH_2OH **12**. The purity of the phosphine is determined to be around 91%.

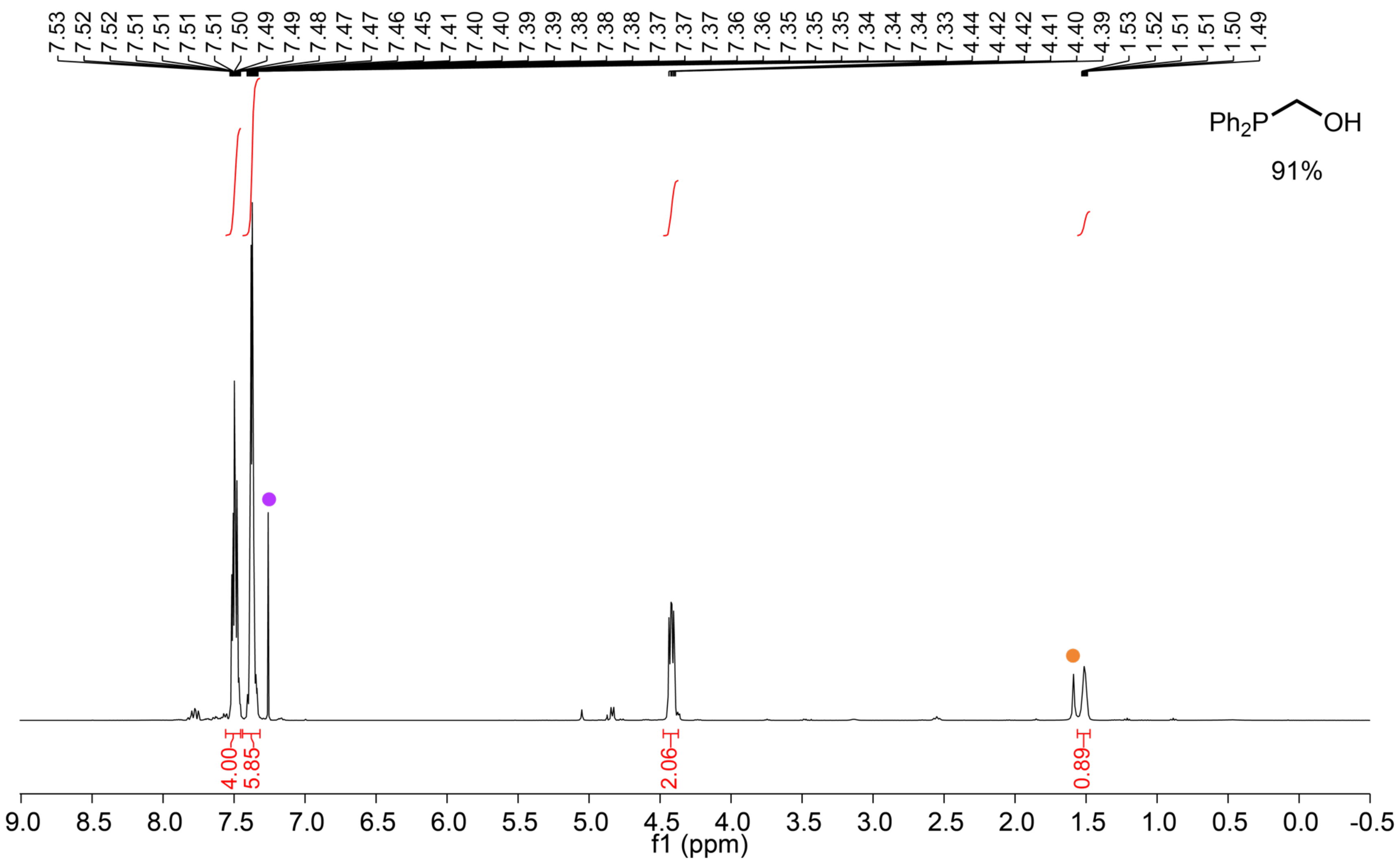

Figure S27. ^{1}H NMR (400.30 MHz, $CDCl_3$) spectrum of PPh_2CH_2OH (**12**). Residual proteo-solvent (●) and H_2O (●).

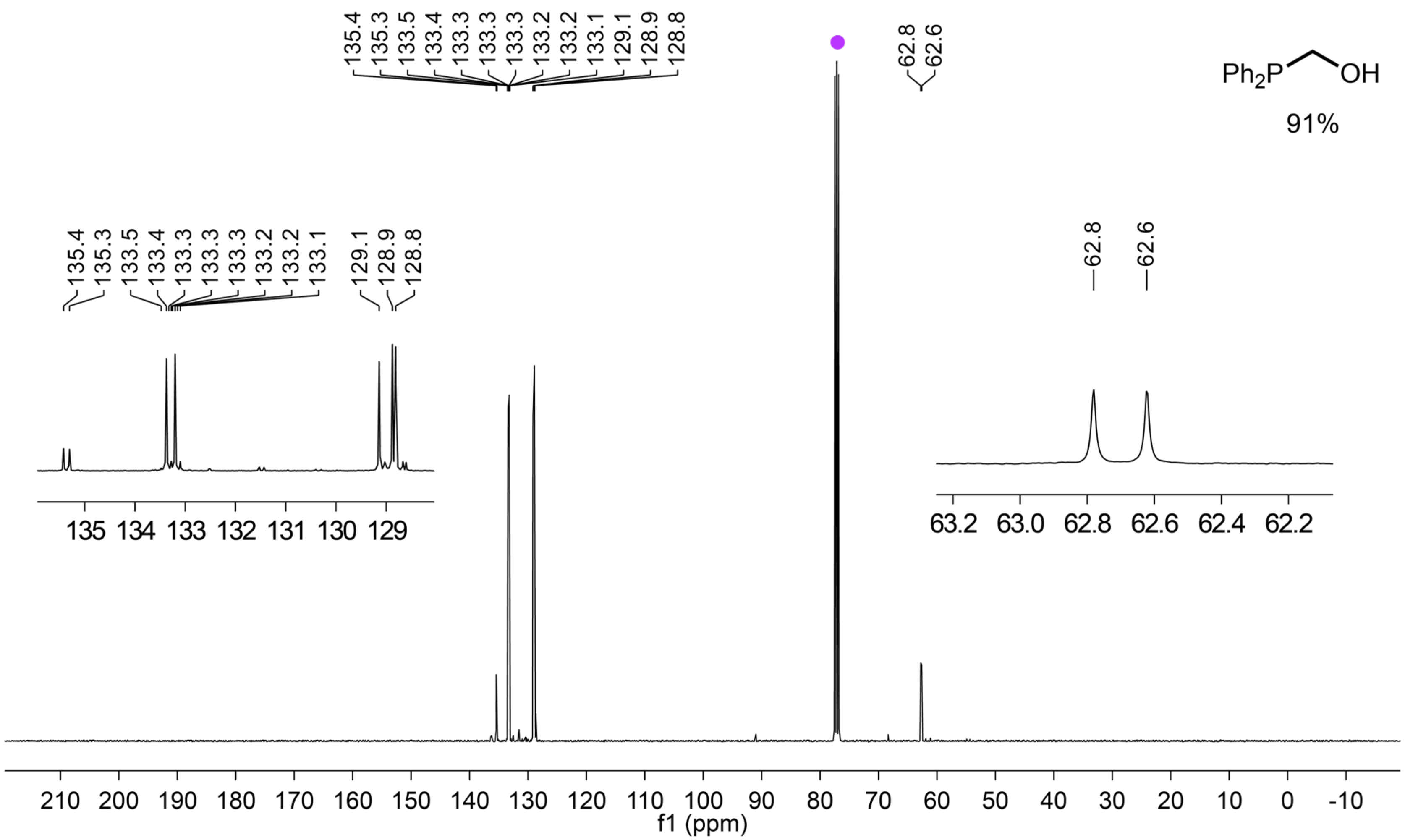

Figure S28. $^{13}C\{^{1}H\}$ NMR (100.67 MHz, $CDCl_3$) spectrum of PPh_2CH_2OH (**12**). Deuterated solvent (•)

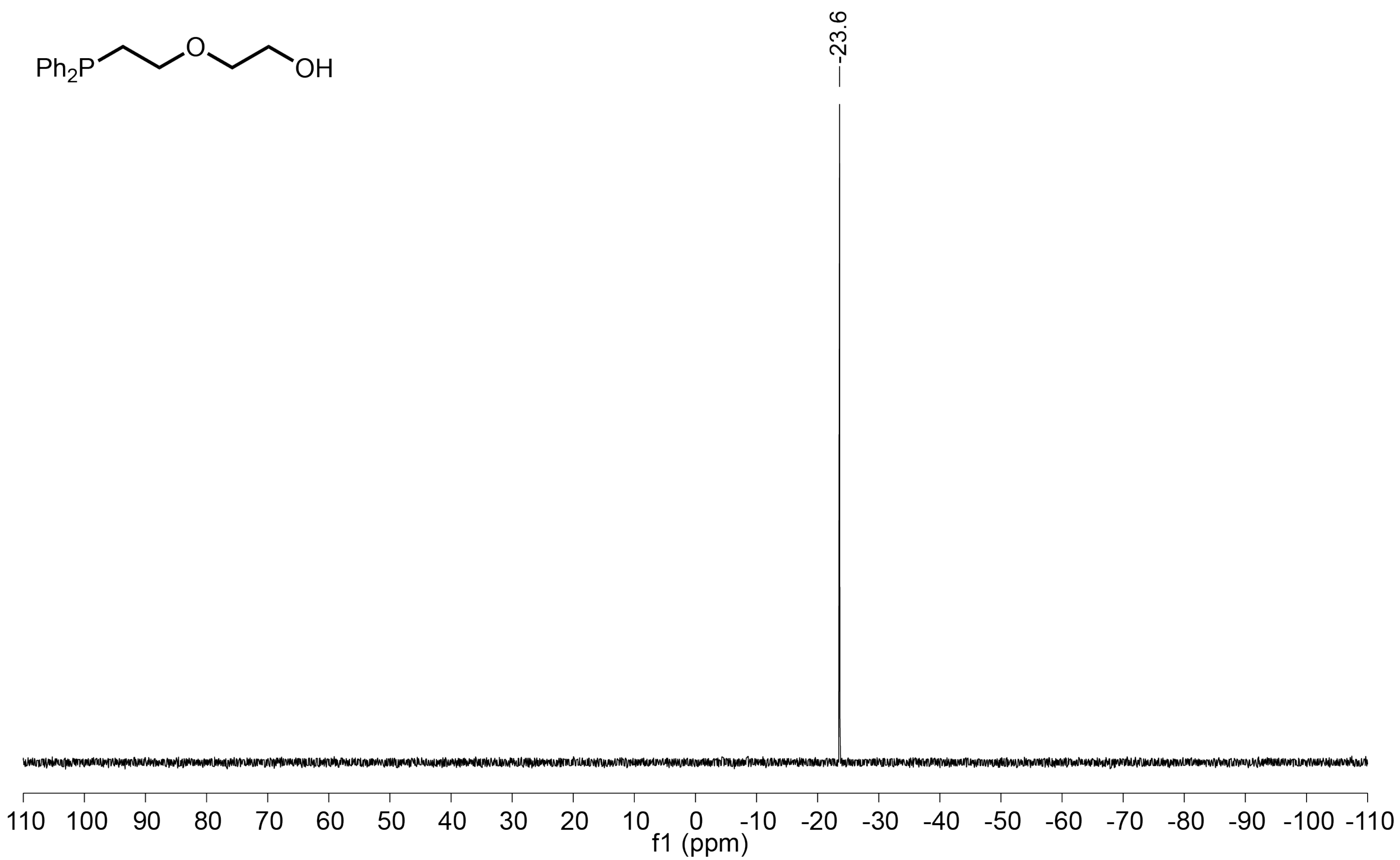

Figure S29. $^{31}P\{^{1}H\}$ NMR (162.04 MHz, $CDCl_3$) spectrum of Peg-ProPhos.

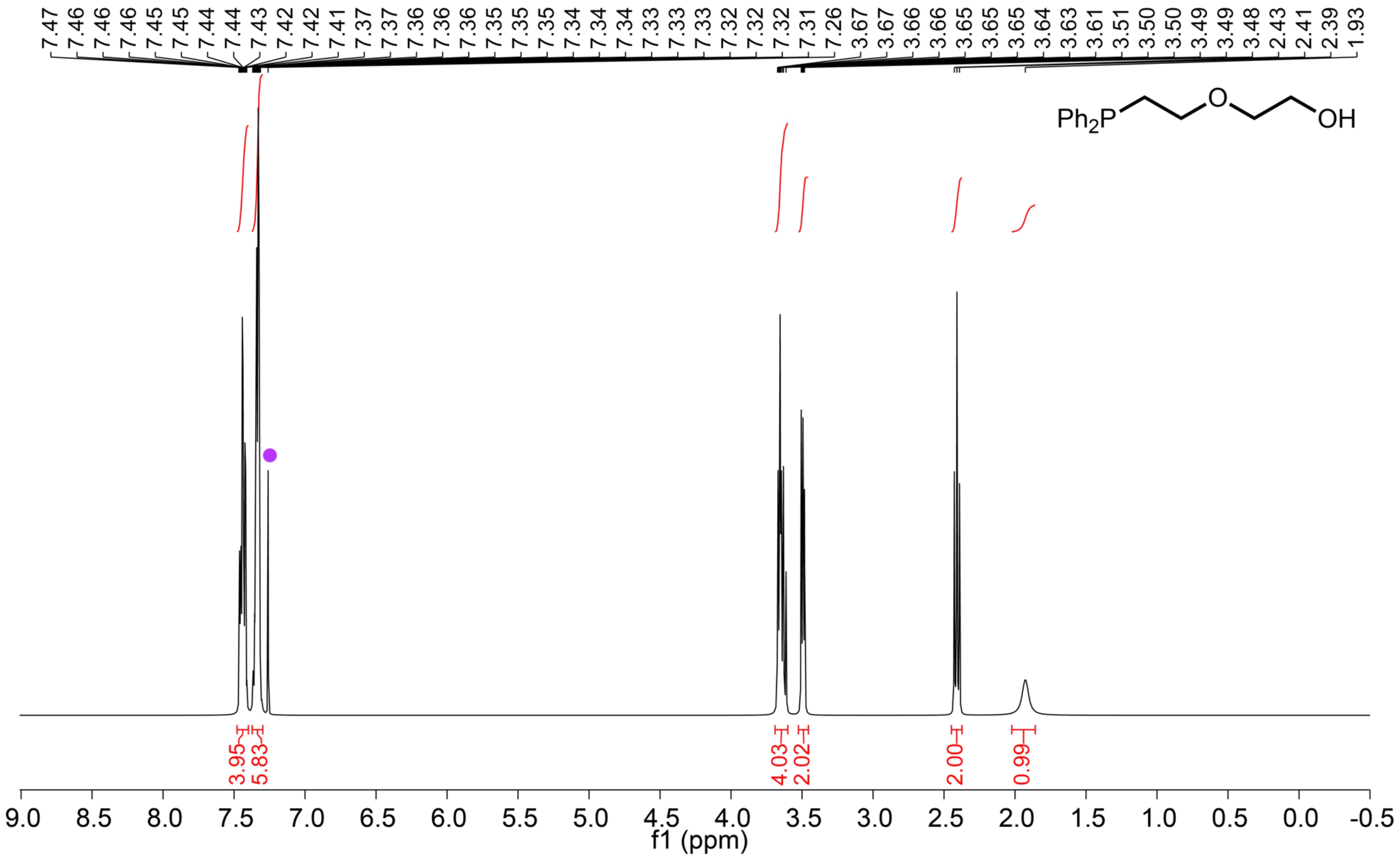

Figure S30. ^{1}H NMR (400.30 MHz, $CDCl_3$) spectrum of Peg-ProPhos. Residual proteo-solvent (•)

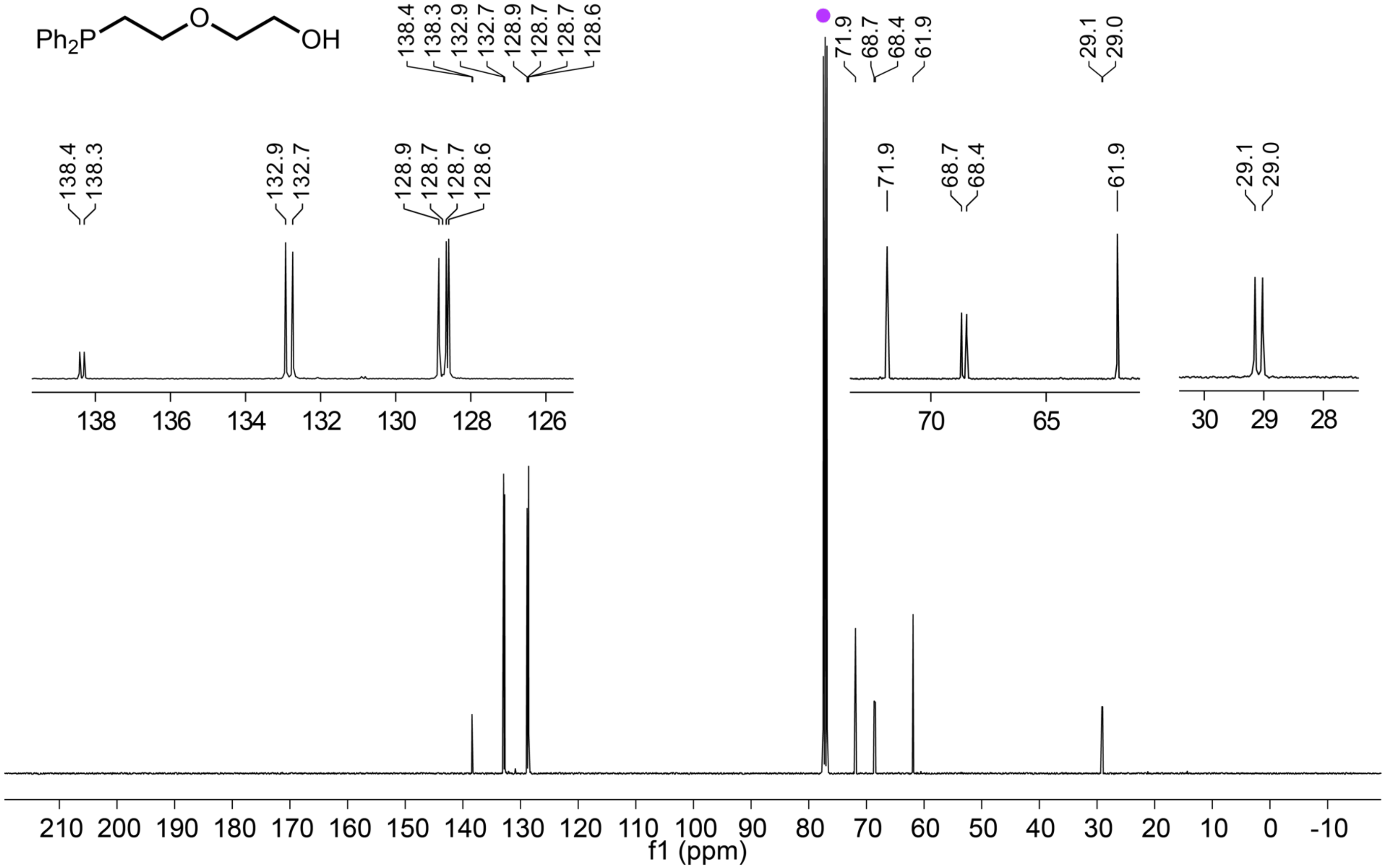

Figure S31. $^{13}C\{^1H\}$ NMR (100.67 MHz, $CDCl_3$) spectrum of Peg-ProPhos. Deuterated solvent (•)

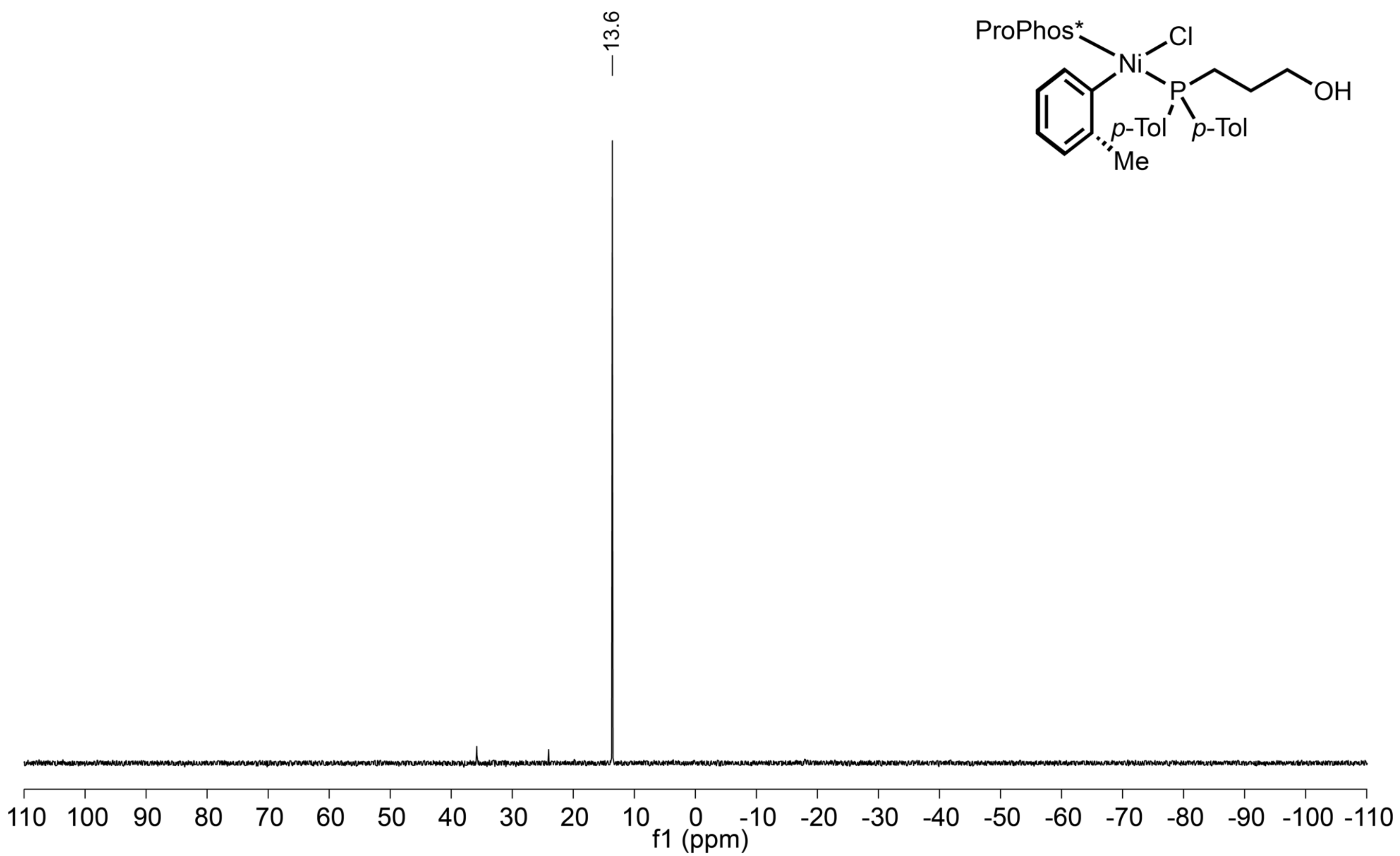

Figure S32. $^{31}P\{^{1}H\}$ NMR (202.47 MHz, *i*PrOD-*d*$_8$) spectrum of Ni(ProPhos*)$_2$Cl(*o*-Tol) **25**. The isolated complex contains a small amount of ProPhos* oxide (~36 ppm) and one unidentified non-Ni species (~24 ppm).

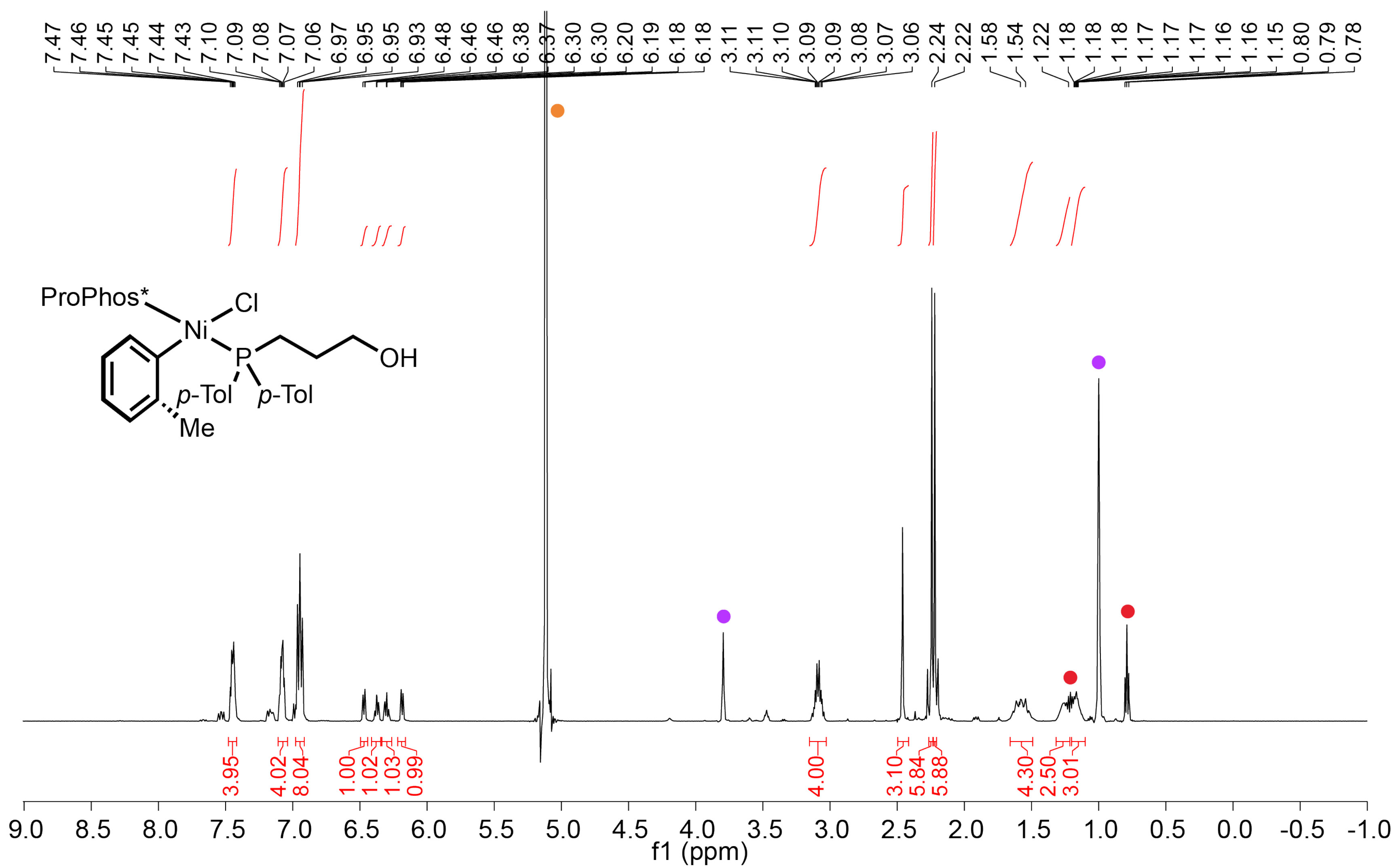

Figure S33. ^{1}H NMR (500.20 MHz, *i*PrOD-*d*$_8$) spectrum of Ni(ProPhos*)$_2$Cl(*o*-Tol) **25**. H_2O (●), residual proteo-solvent (●) and pentane (●).

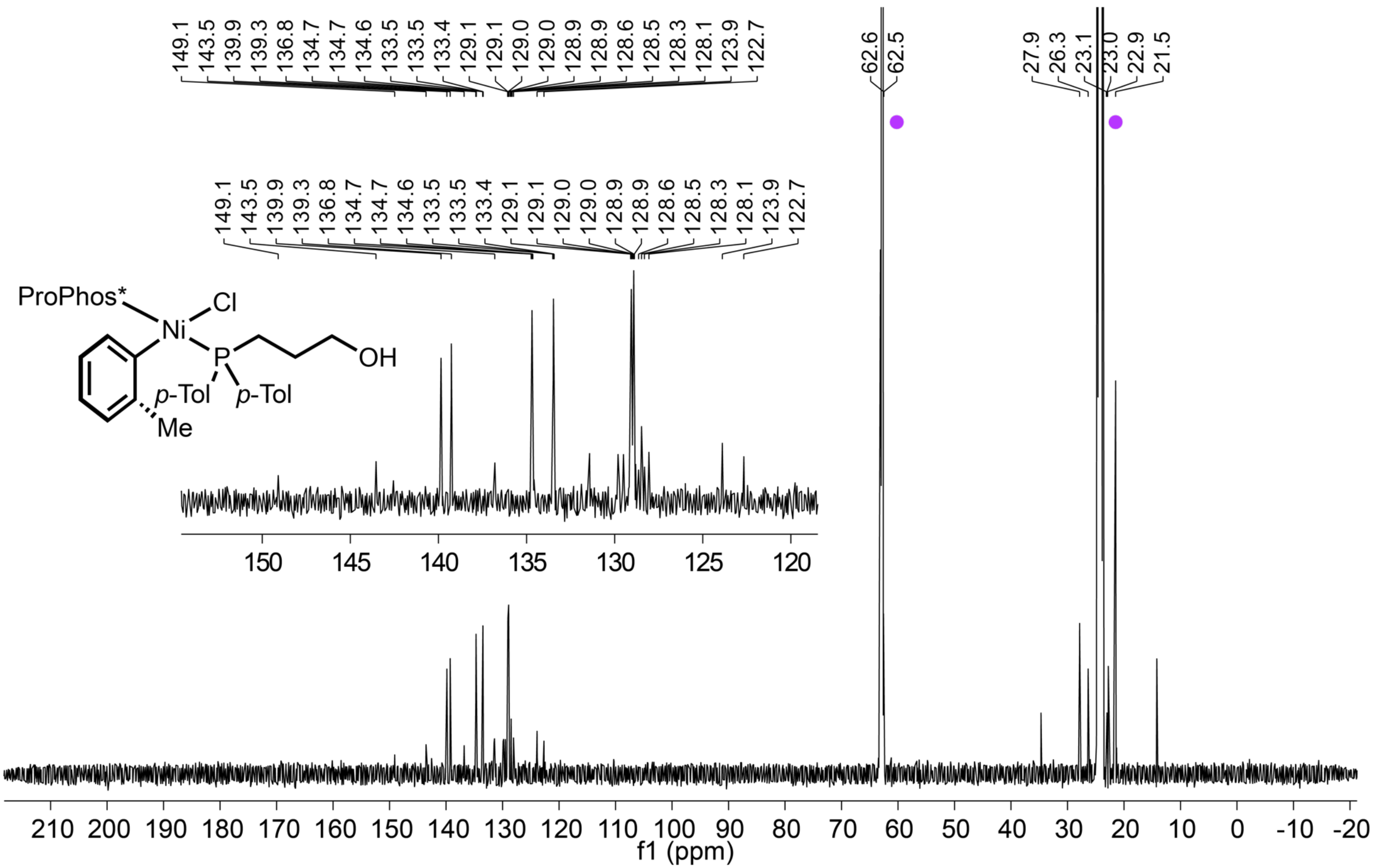

Figure S34. $^{13}C\{^1H\}$ (125.79 MHz, *i*PrOD-d_8) NMR spectrum of Ni(ProPhos*)$_2$Cl(*o*-Tol) **25**. Deuterated solvent (●).

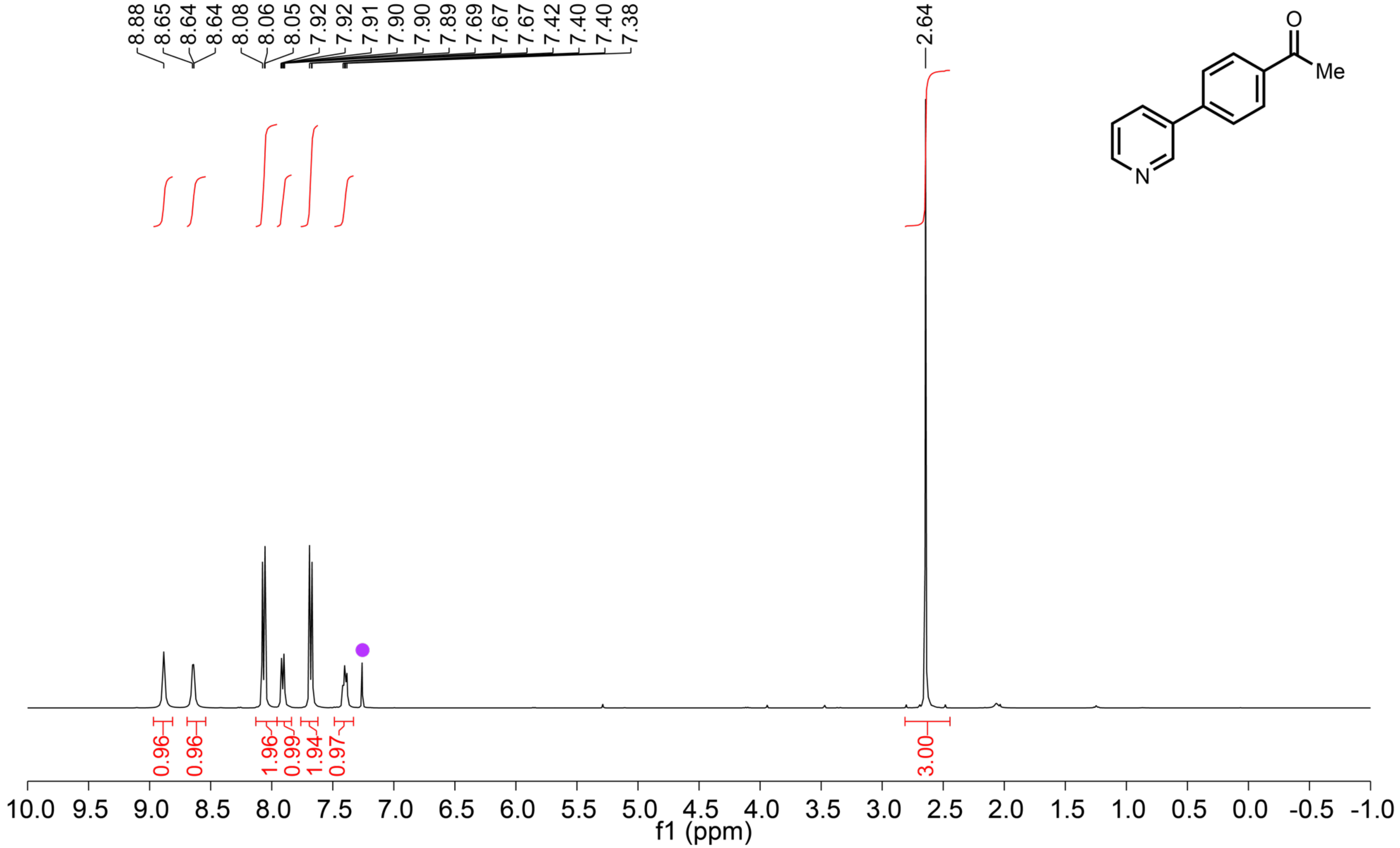

Figure S35. ^{1}H NMR (400.30 MHz, $CDCl_3$) spectrum of compound **17**. Residual proteo-solvent (•)

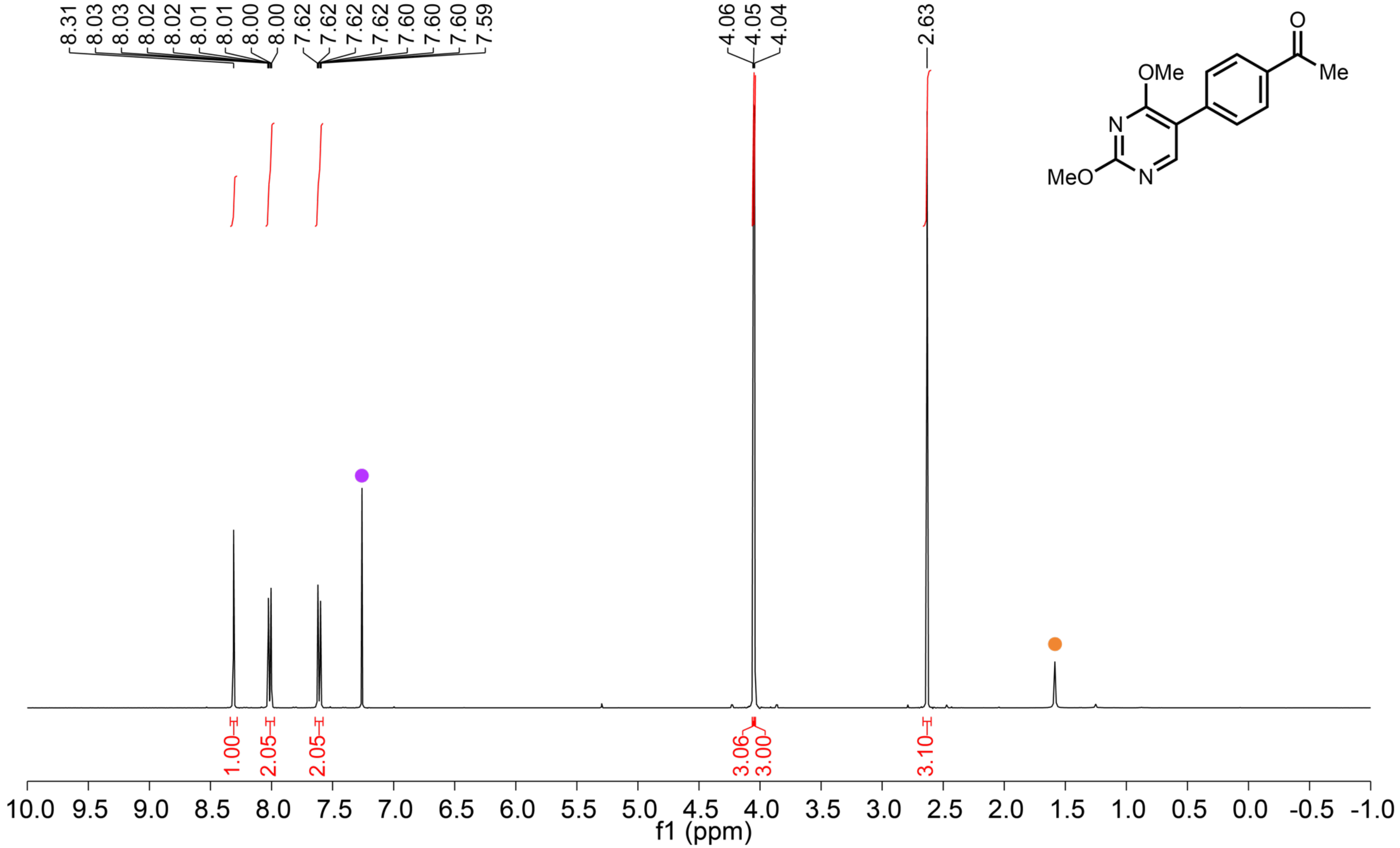

Figure S36. ^{1}H NMR (400.30 MHz, $CDCl_3$) spectrum of compound **27**. Residual proteo-solvent (•) and H_2O (•).

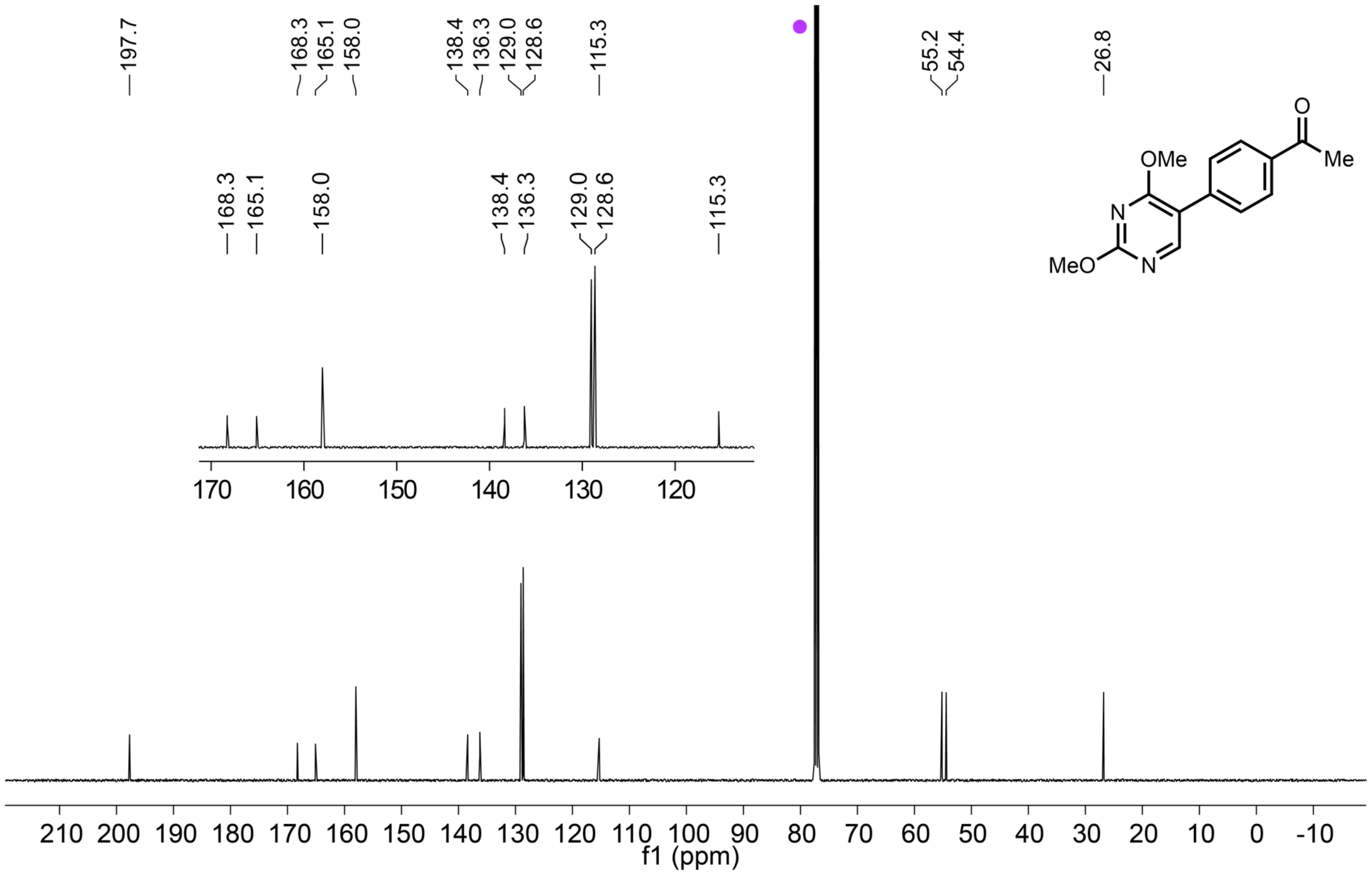

Figure S37. $^{13}C\{^1H\}$ NMR (100.67 MHz, $CDCl_3$) spectrum of compound **27**. Deuterated solvent (•).

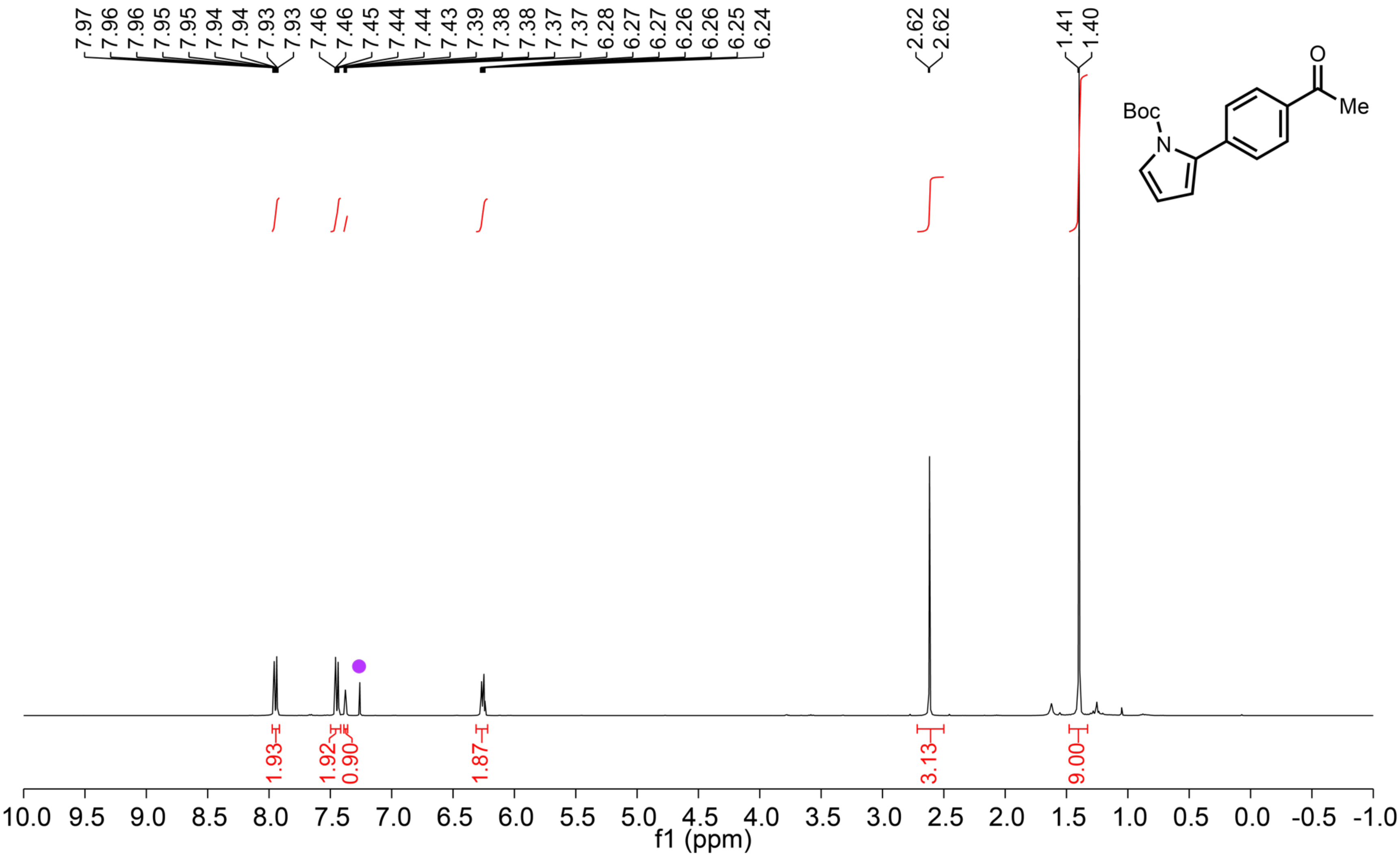

Figure S38. ^{1}H NMR (400.30 MHz, $CDCl_3$) spectrum of compound **28**. Residual proteo-solvent (•).

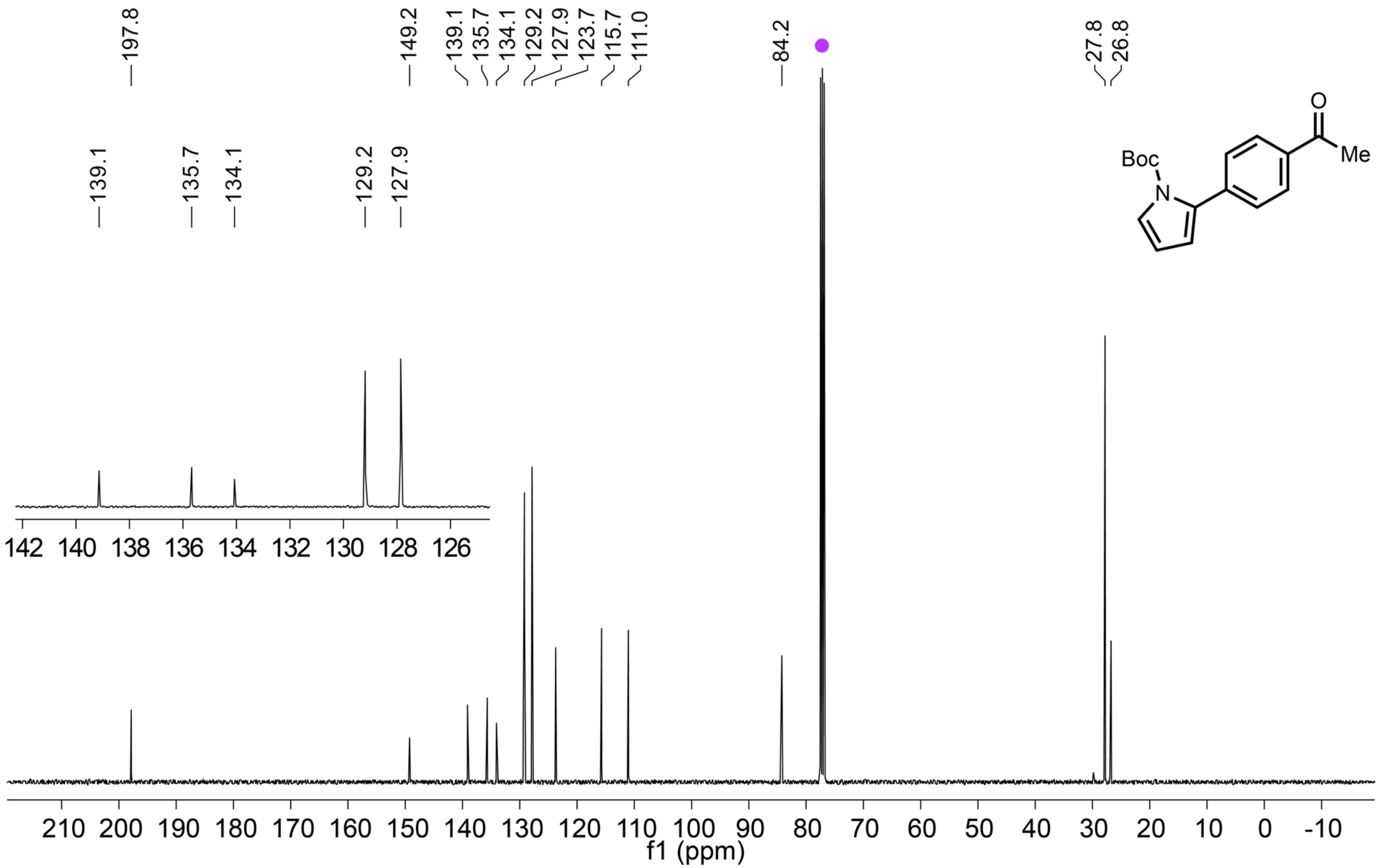

Figure S39. $^{13}C\{^1H\}$ NMR (100.67 MHz, $CDCl_3$) spectrum of compound **28**. Deuterated solvent (•).

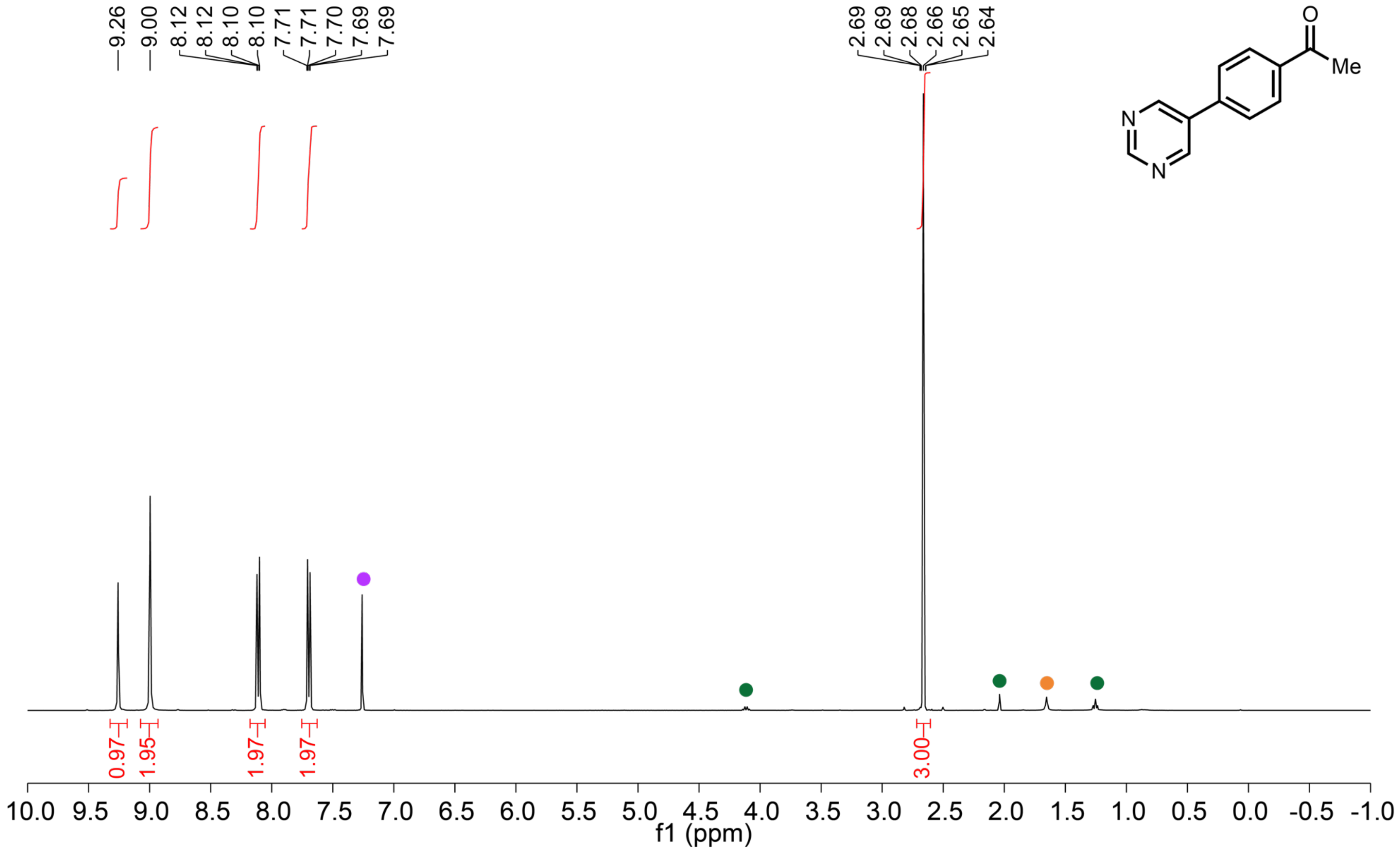

Figure S40. ^{1}H NMR (400.30 MHz, $CDCl_3$) spectrum of compound **29**. Residual proteo-solvent (•), EtOAc (•) and H_2O (•).

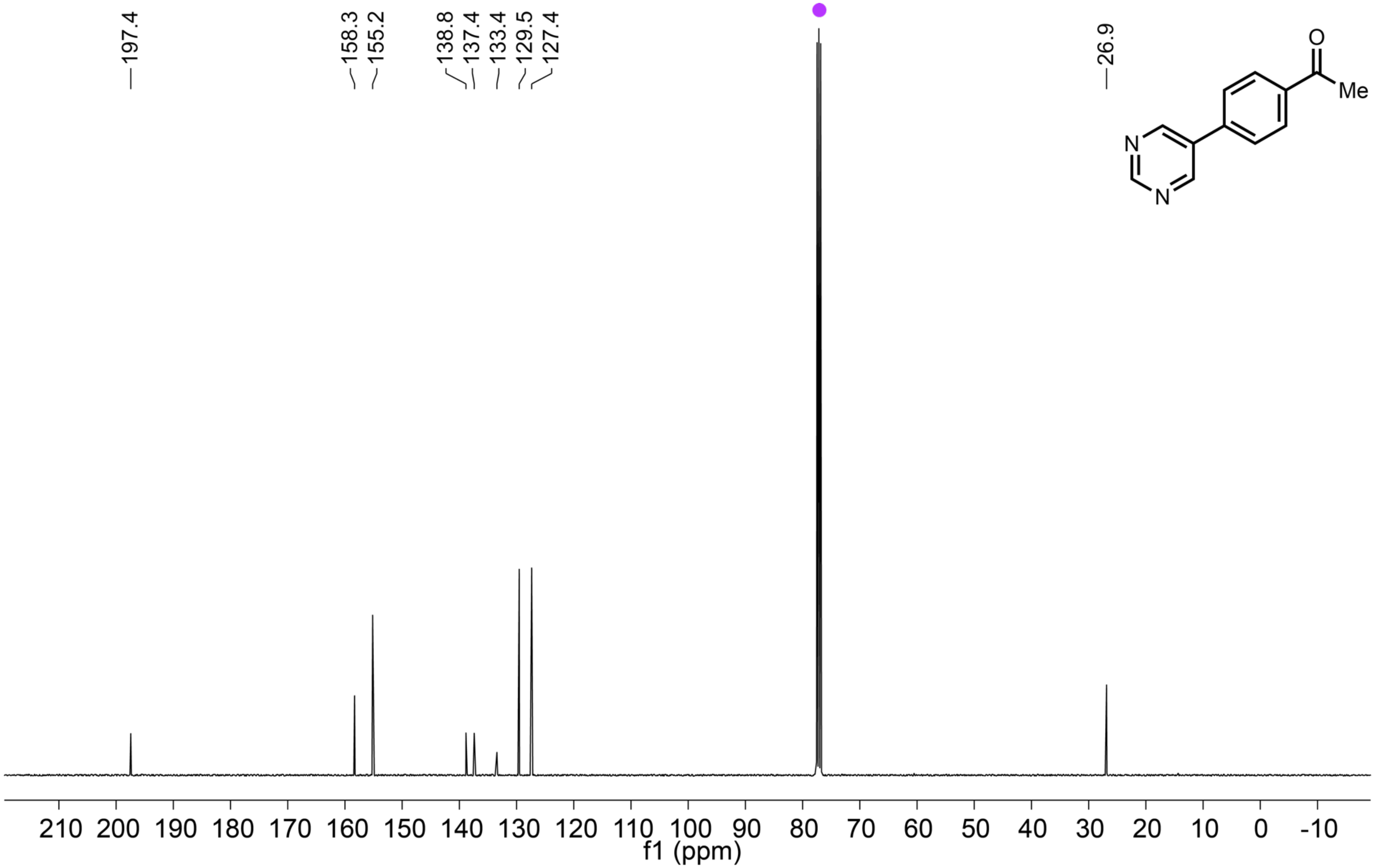

Figure S41. $^{13}C\{^{1}H\}$ NMR (100.67 MHz, $CDCl_3$) spectrum of compound **29**. Deuterated solvent (•).

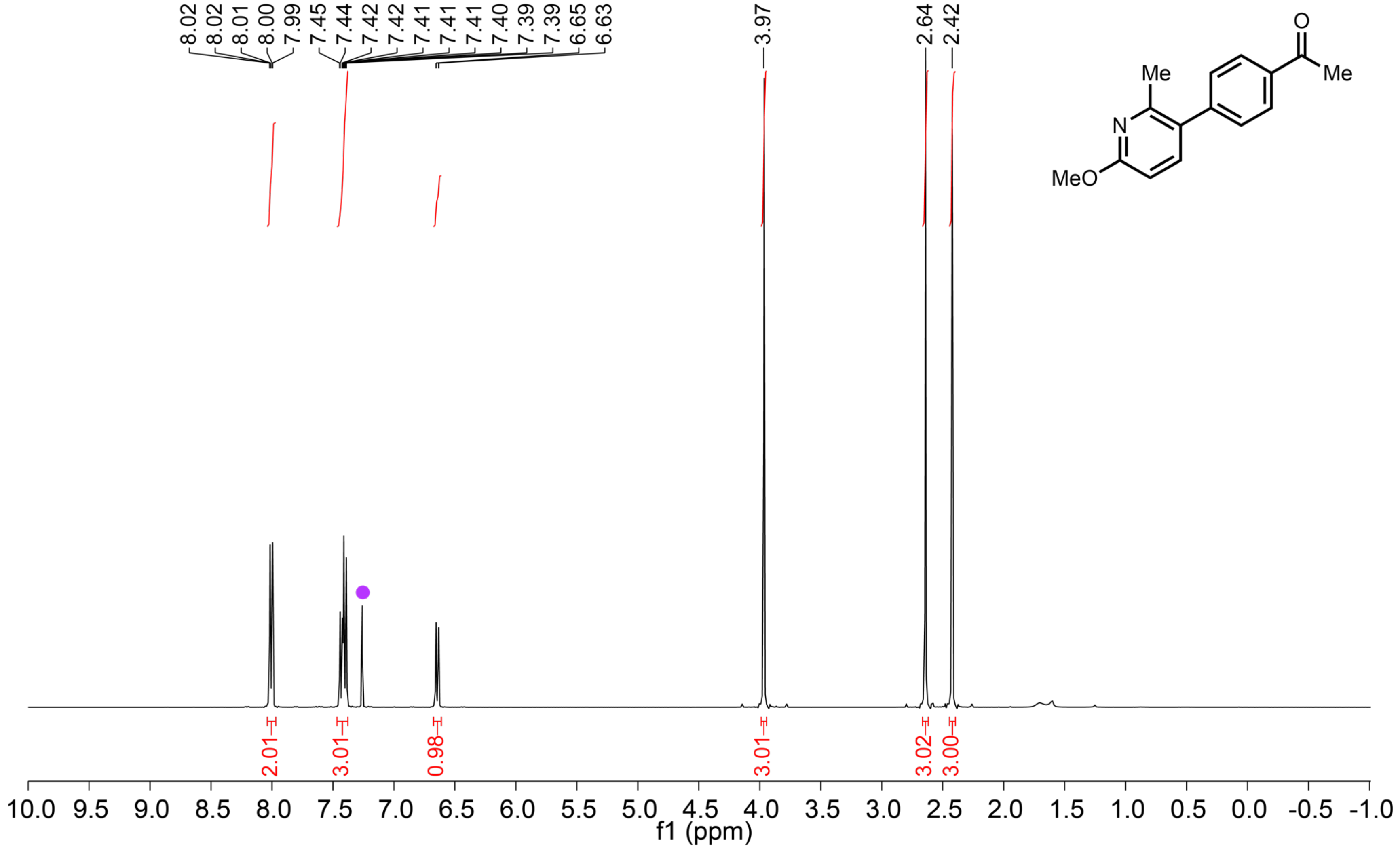

Figure S42. ^{1}H NMR (400.30 MHz, $CDCl_3$) spectrum of compound **30**. Residual proteo-solvent (•).

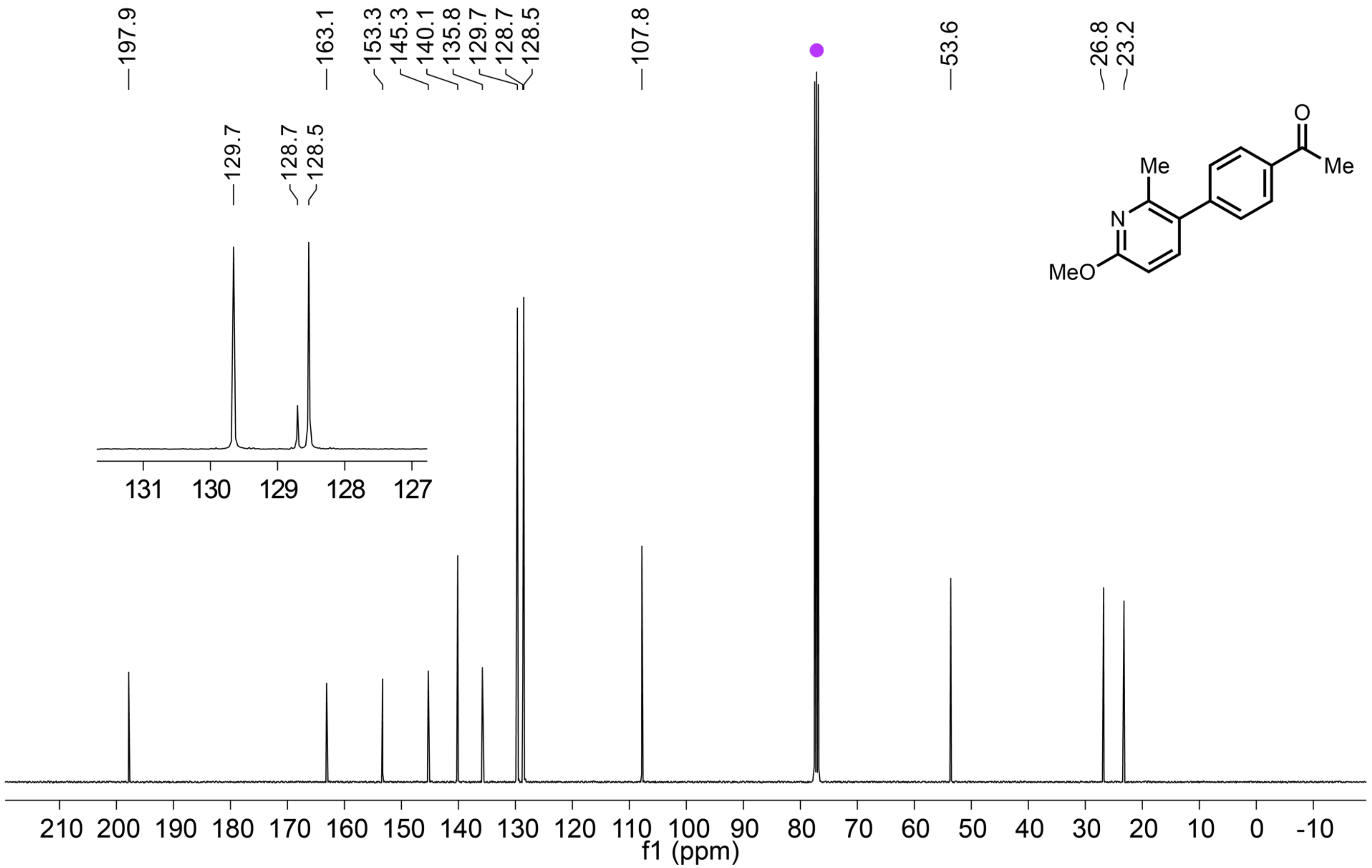

Figure S43. $^{13}C\{^{1}H\}$ NMR (100.67 MHz, $CDCl_3$) spectrum of compound **30**. Deuterated solvent (•).

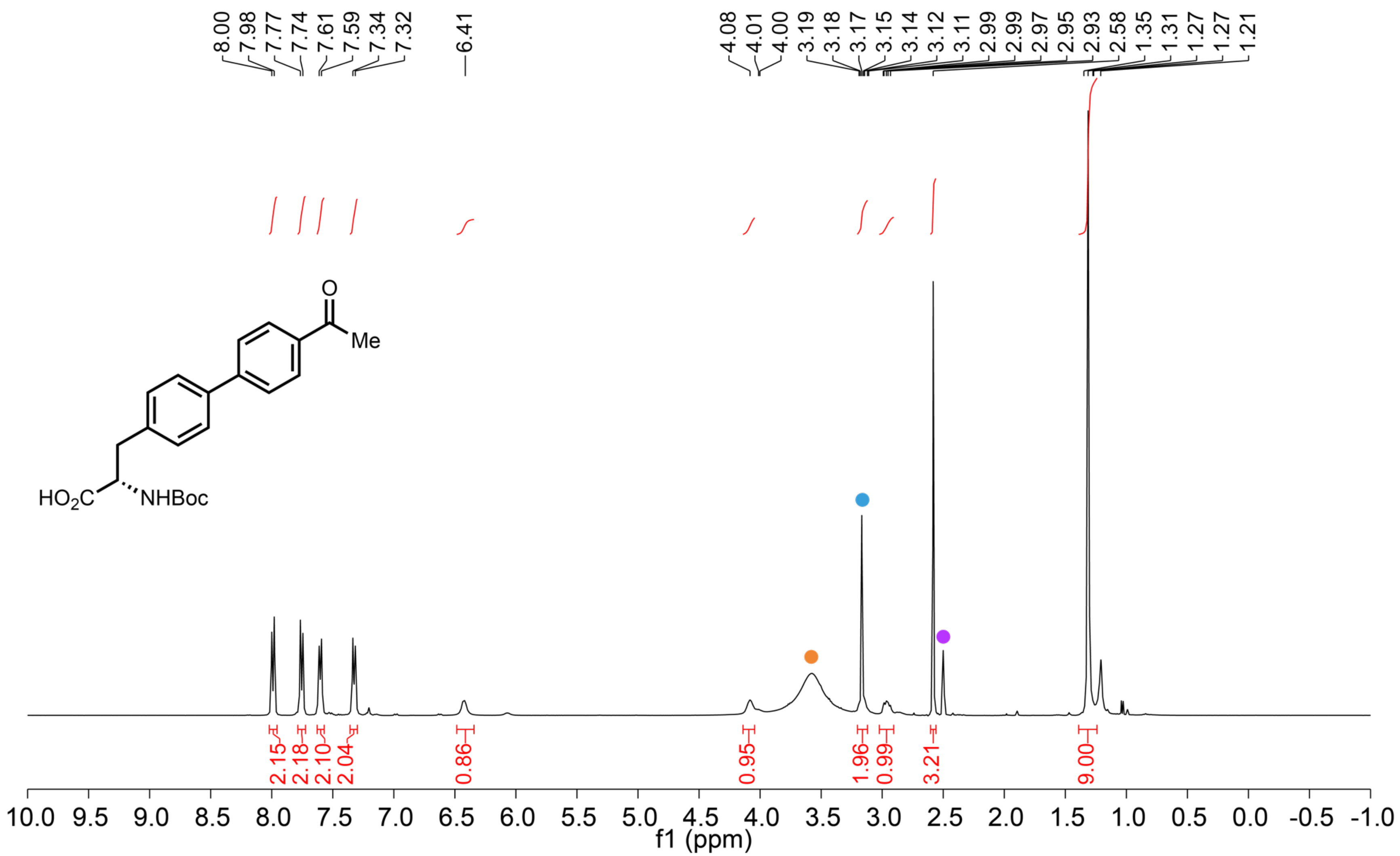

Figure S44. ^{1}H NMR (400.30 MHz, $CDCl_3$) spectrum of compound **31**. H_2O (●), MeOH (●) and residual proteo-solvent (●).

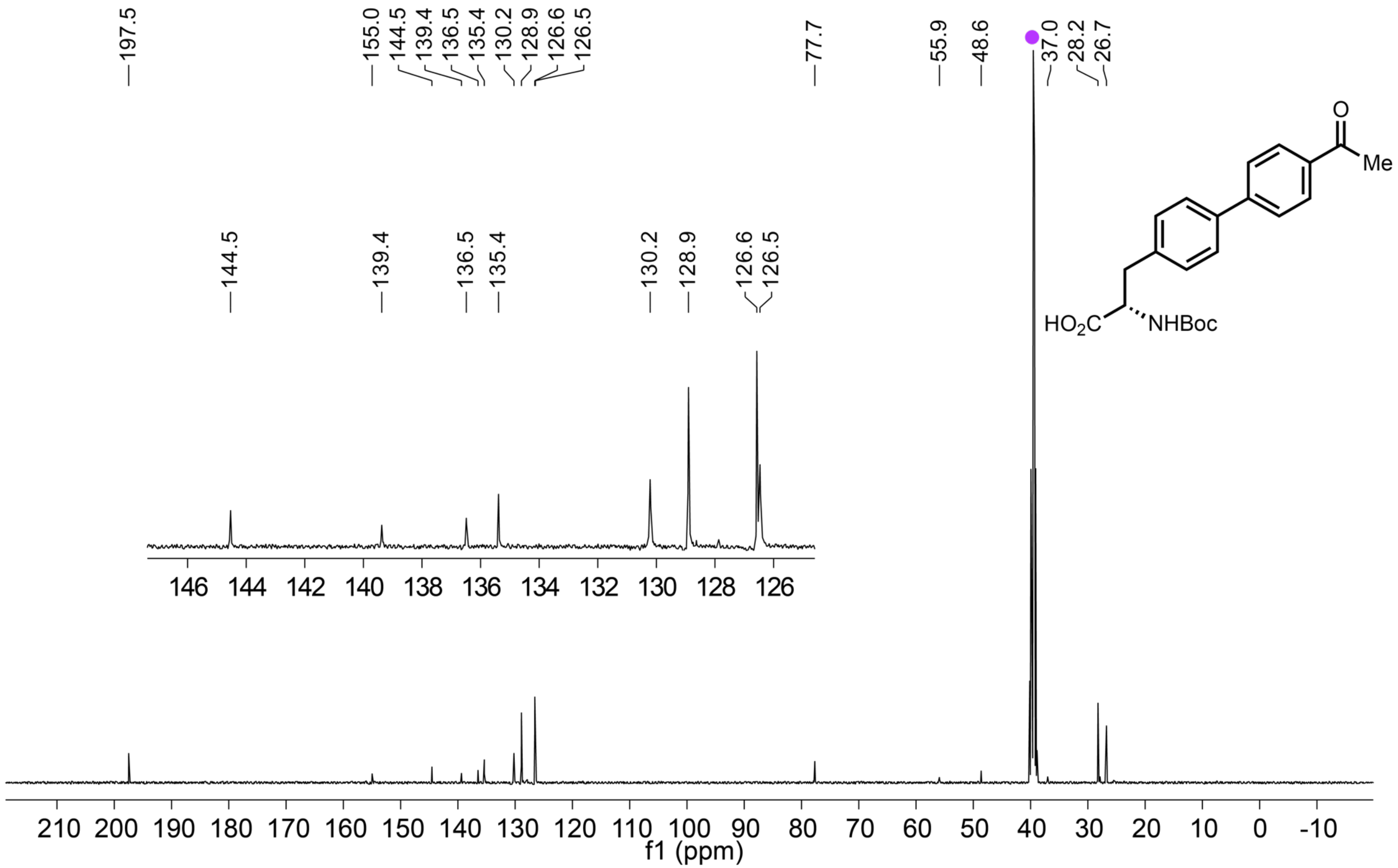

Figure S45. $^{13}C\{^1H\}$ NMR (100.67 MHz, $CDCl_3$) spectrum of compound **31**. Deuterated solvent (•).

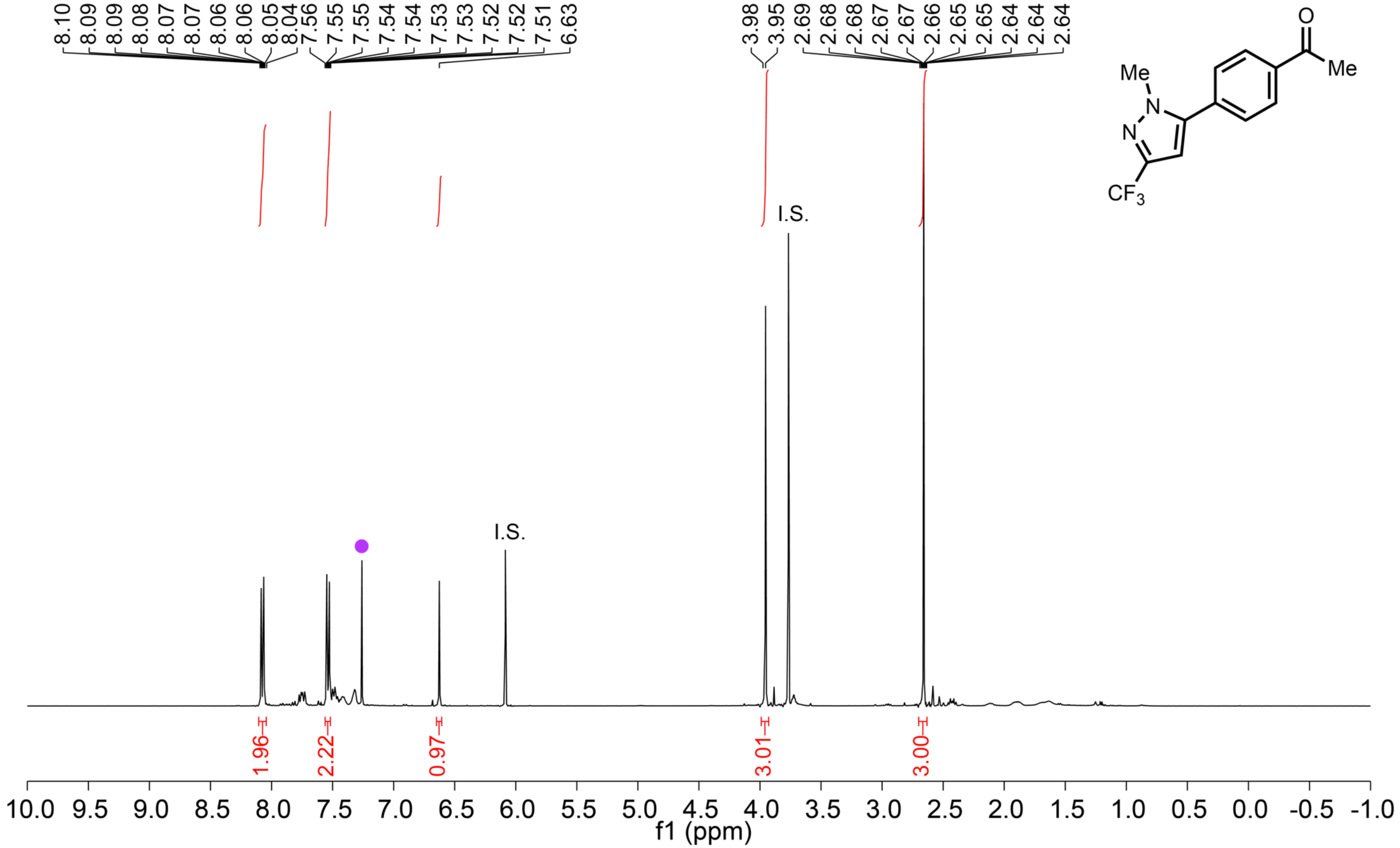

Figure S46. ^{1}H NMR (400.30 MHz, $CDCl_3$) spectrum of the crude of compound **32** with internal standard, 1,3,5-trimethoxylbenezene (I.S.). Residual proteo-solvent (•).

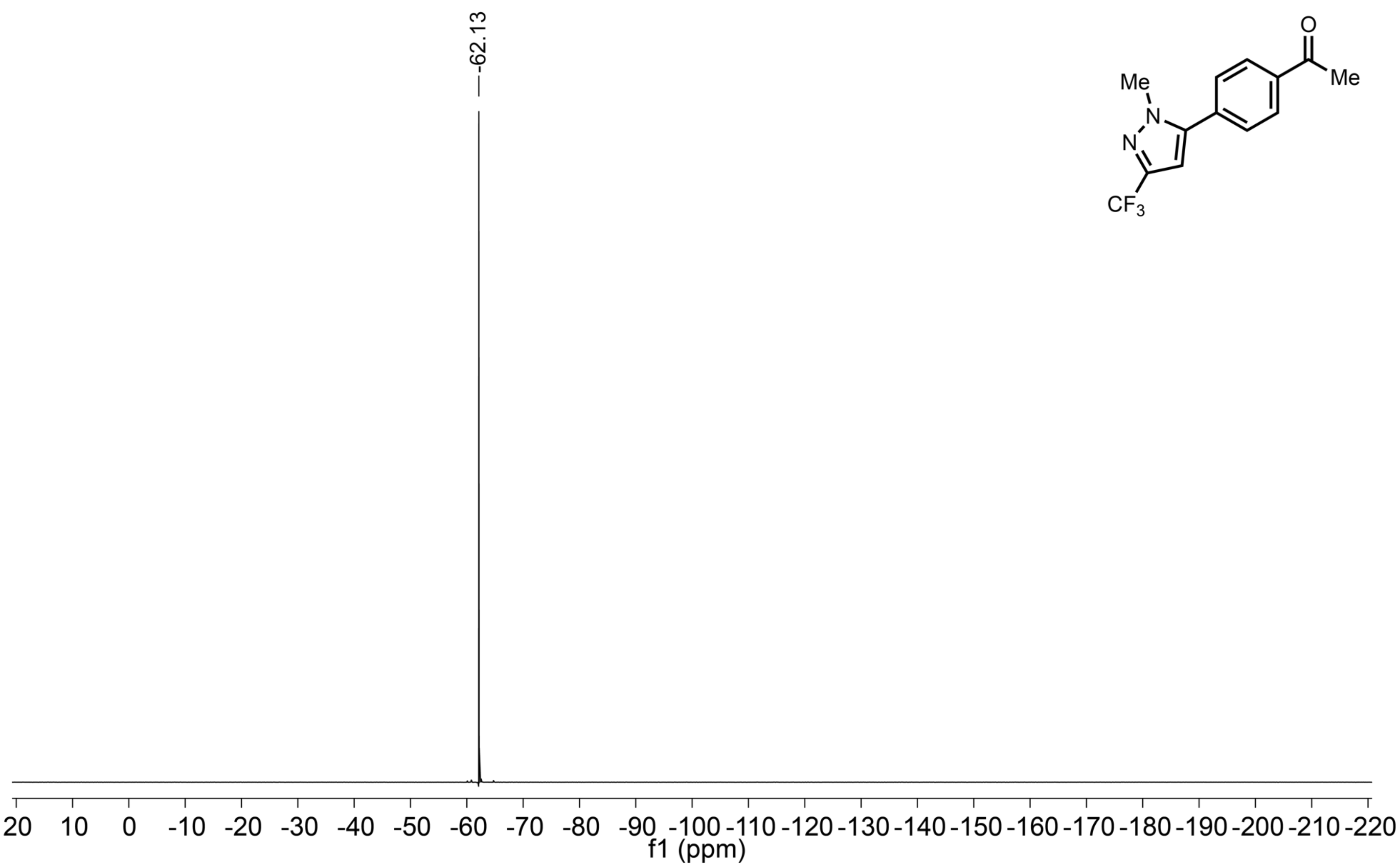

Figure S47. $^{19}F\{^{1}H\}$ NMR (470.61 MHz, $CDCl_3$) spectrum of the crude of compound **32** with internal standard, 1,3,5-trimethoxylbenezene (I.S.).

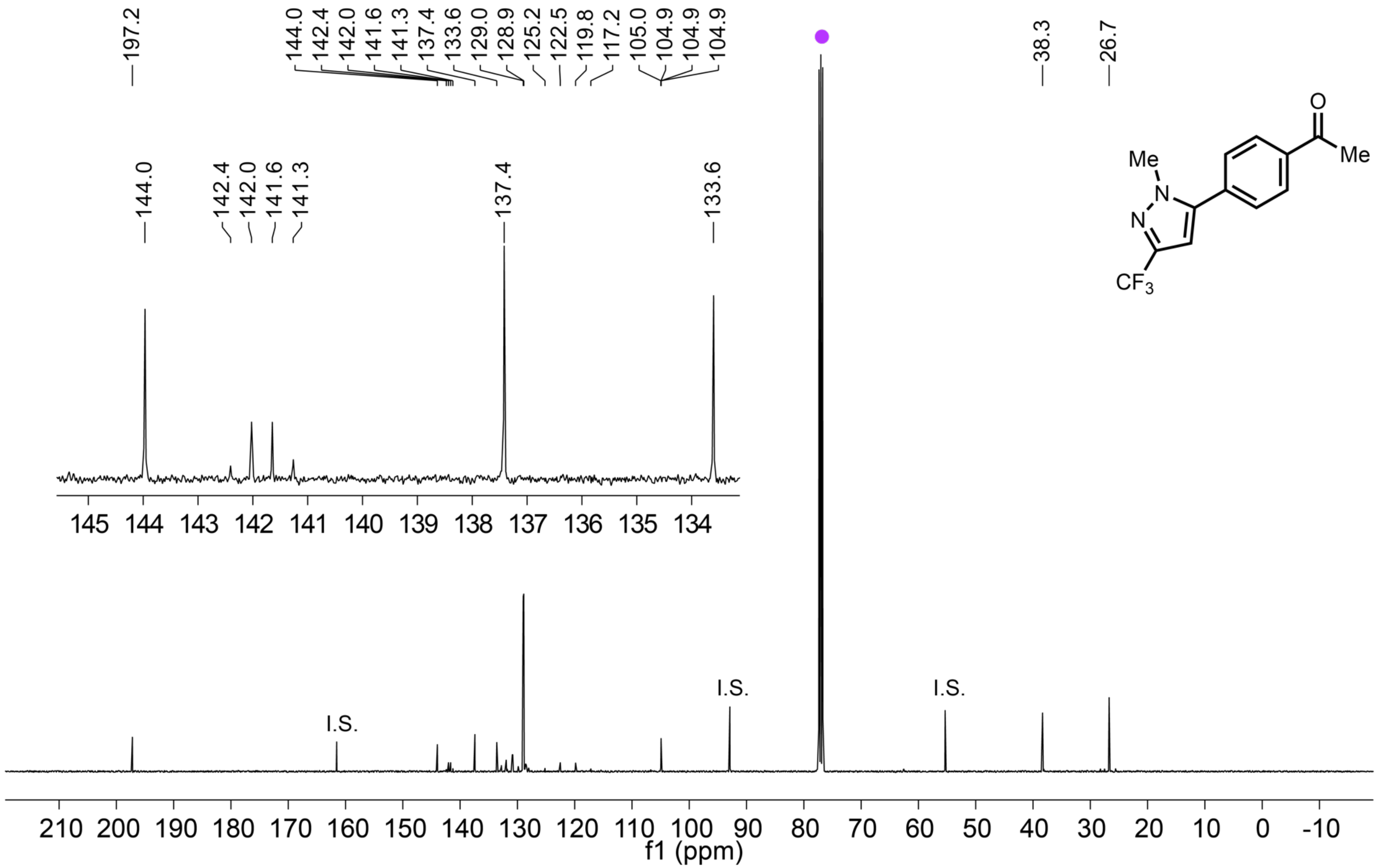

Figure S48. $^{13}C\{^{1}H\}$ NMR (100.67 MHz, $CDCl_3$) spectrum of the crude of compound **32** with internal standard, 1,3,5-trimethoxylbenezene (I.S.). Deuterated solvent (•).

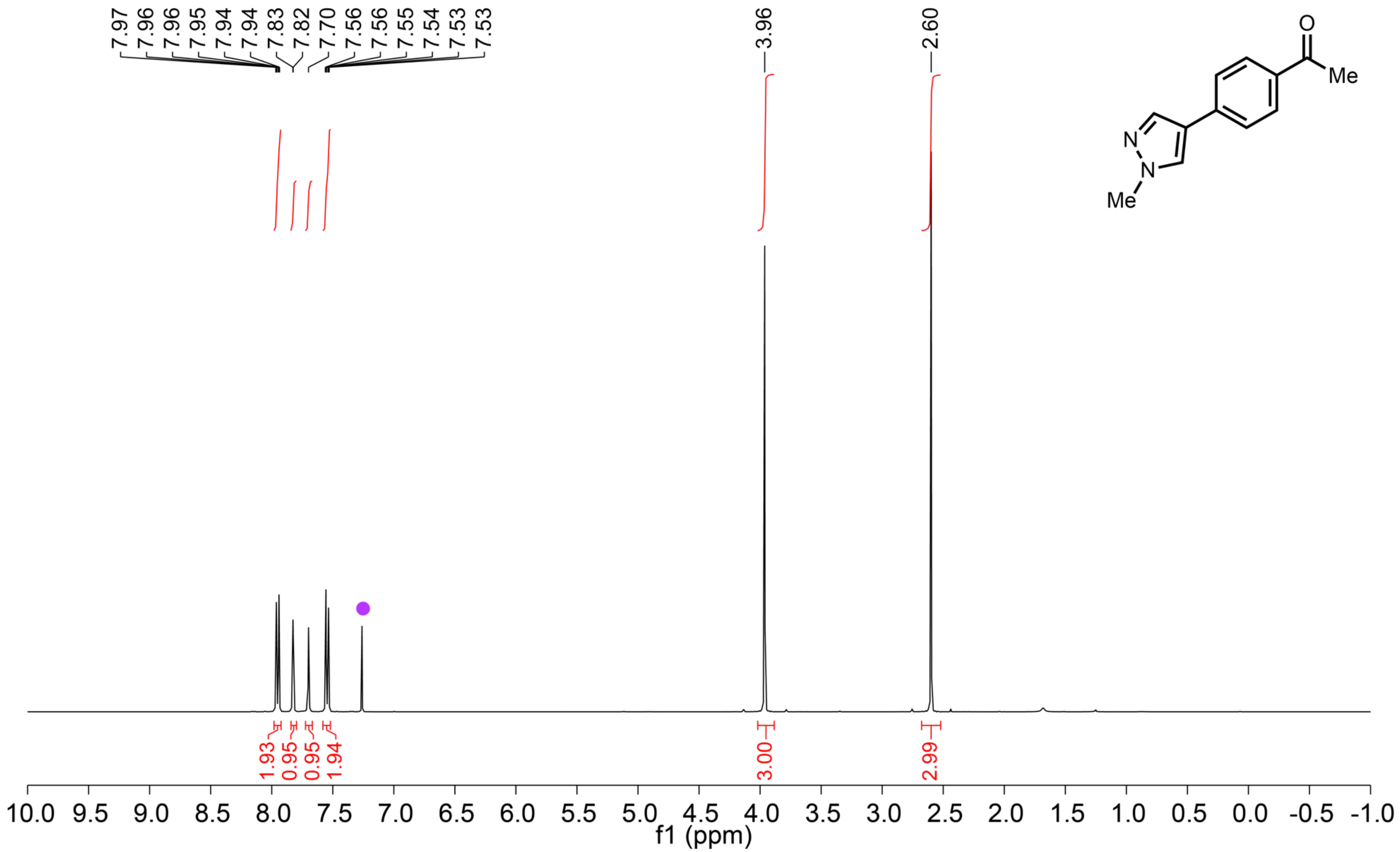

Figure S49. ^{1}H NMR (400.30 MHz, $CDCl_3$) spectrum of compound **6**. Residual proteo-solvent (•).

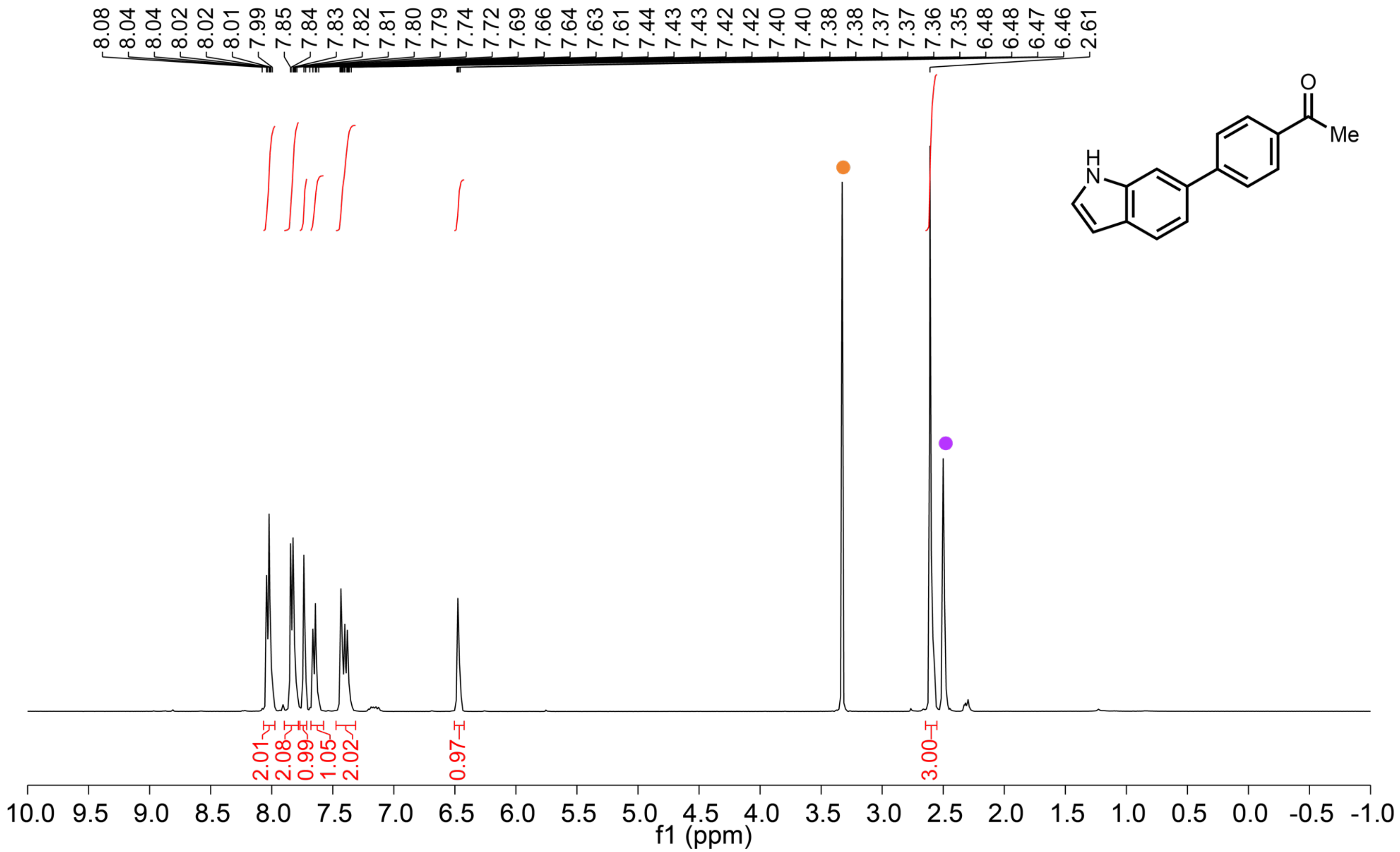

Figure S50. ^{1}H NMR (400.30 MHz, DMSO-d_6) spectrum of compound **33**. Residual proteo-solvent (•).

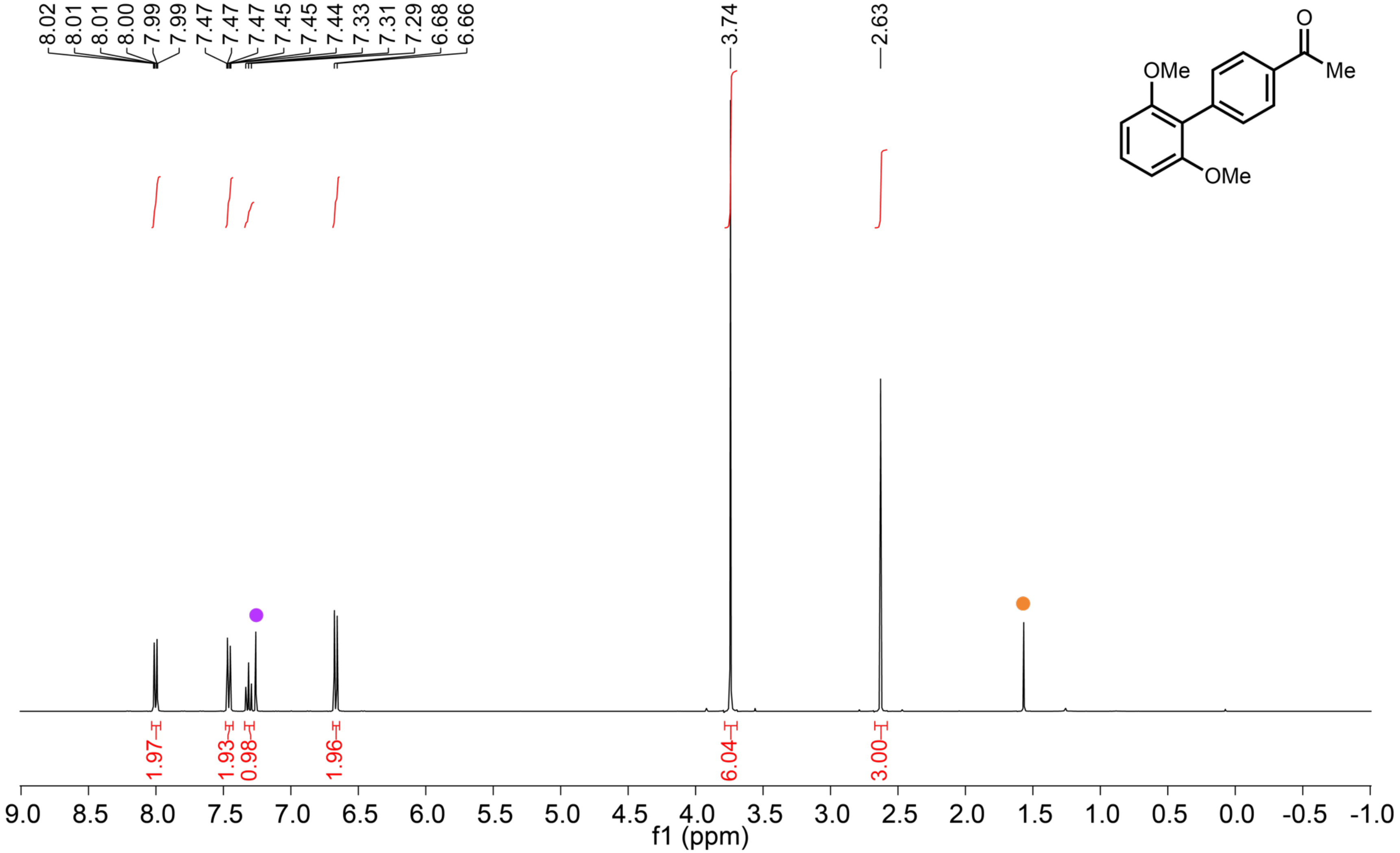

Figure S51. ^{1}H NMR (400.30 MHz, $CDCl_3$) spectrum of compound **34**. Residual proteo-solvent (•) and H_2O (•).

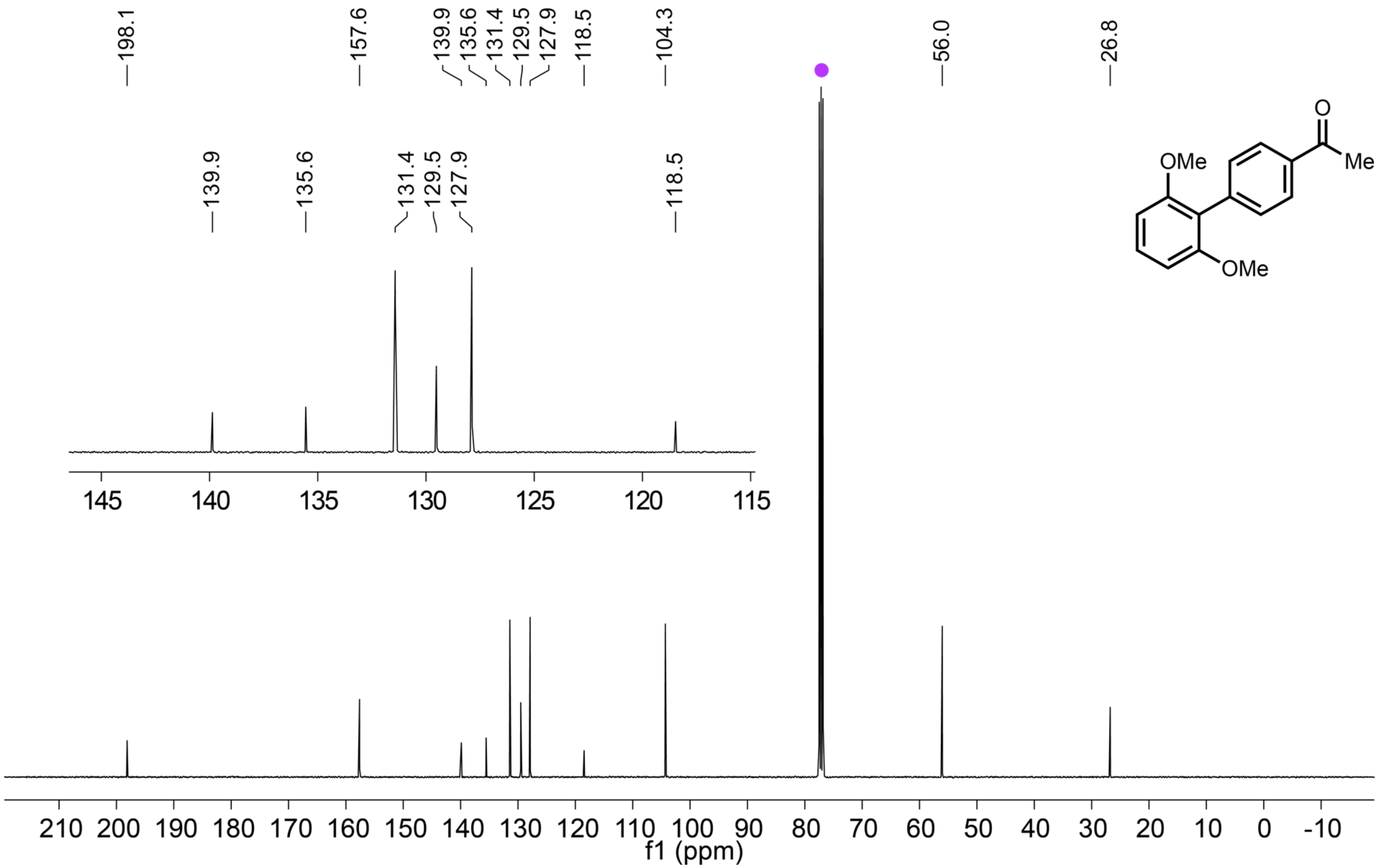

Figure S52. $^{13}C\{^1H\}$ NMR (100.67 MHz, $CDCl_3$) spectrum of compound **34**. Deuterated solvent (•).

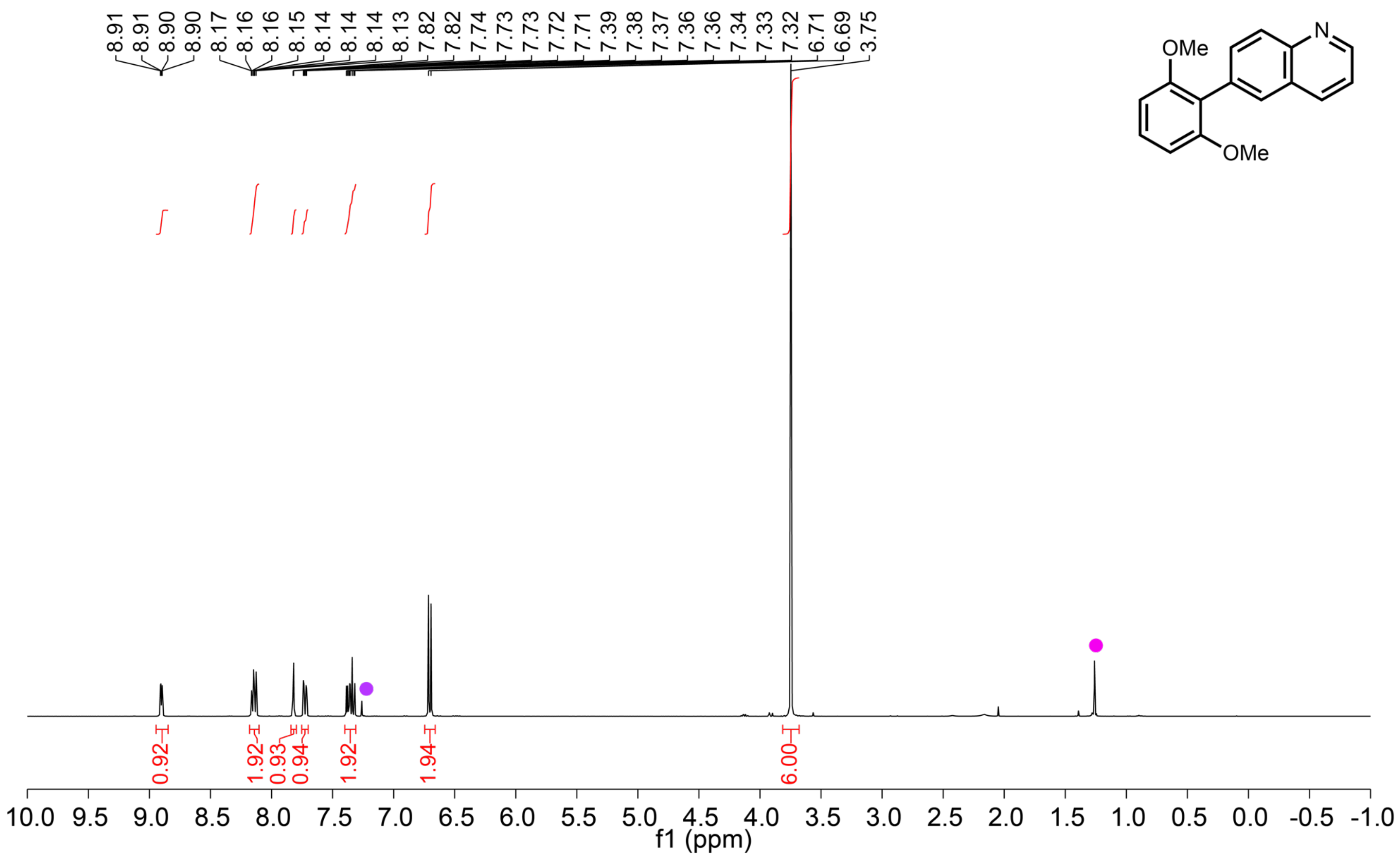

Figure S53. ^{1}H NMR (400.30 MHz, $CDCl_3$) spectrum of compound **35**. Residual proteo-solvent (•) and grease (•).

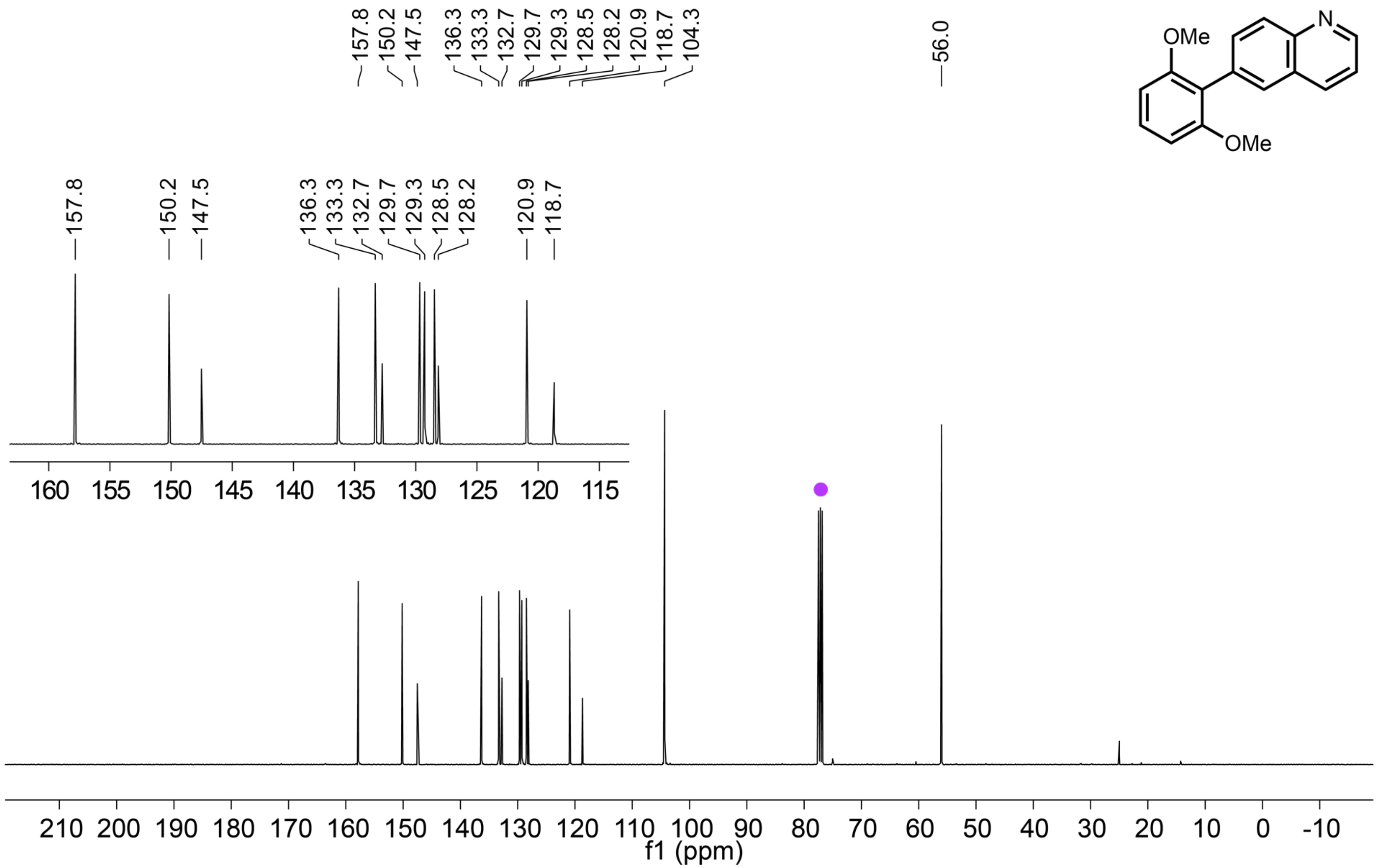

Figure S54. $^{13}C\{^1H\}$ NMR (100.67 MHz, $CDCl_3$) spectrum of compound **35**. Deuterated solvent (•).

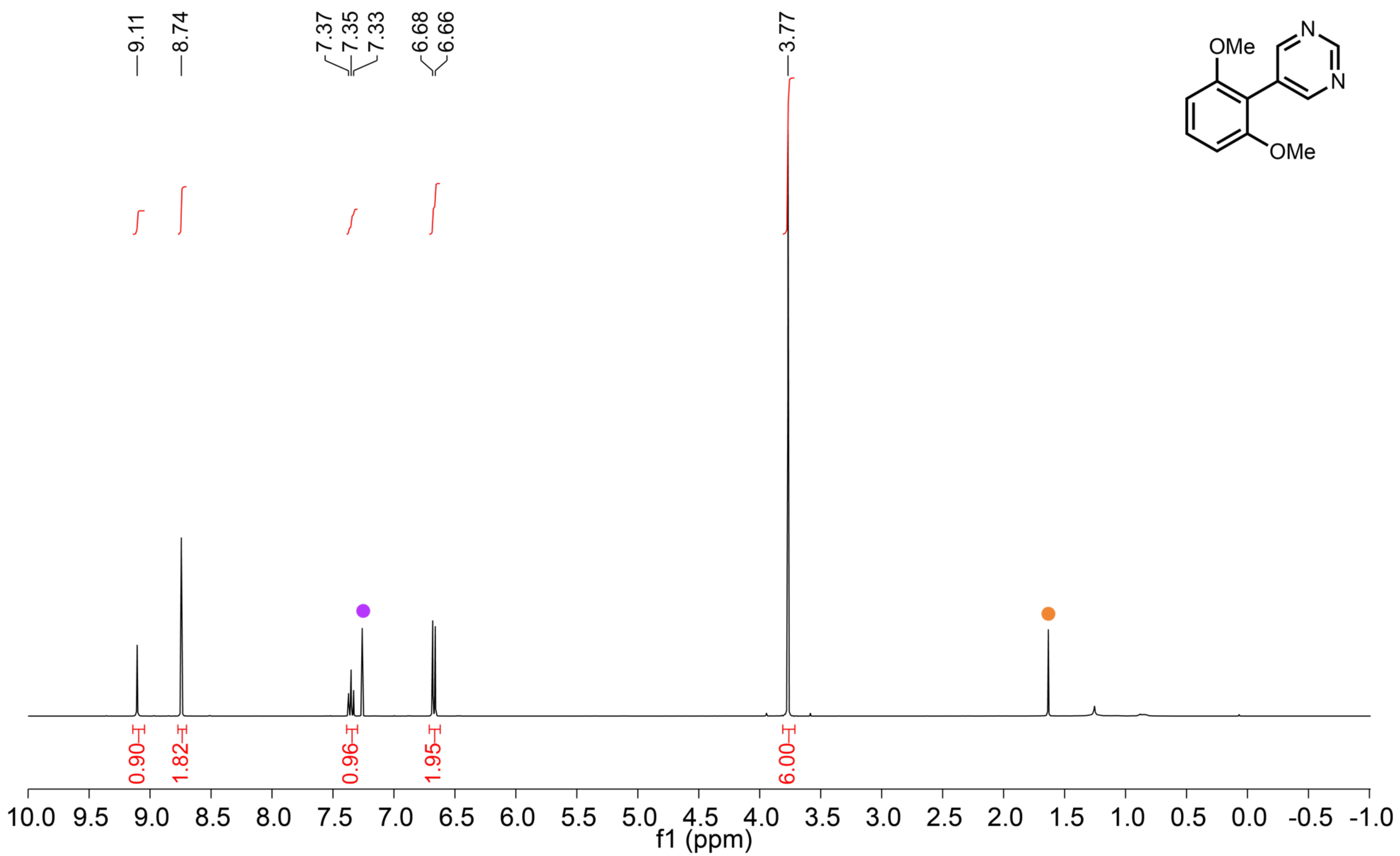

Figure S55. ^{1}H NMR (400.30 MHz, $CDCl_3$) spectrum of compound **36**. Residual proteo-solvent (•) and H_2O (•).

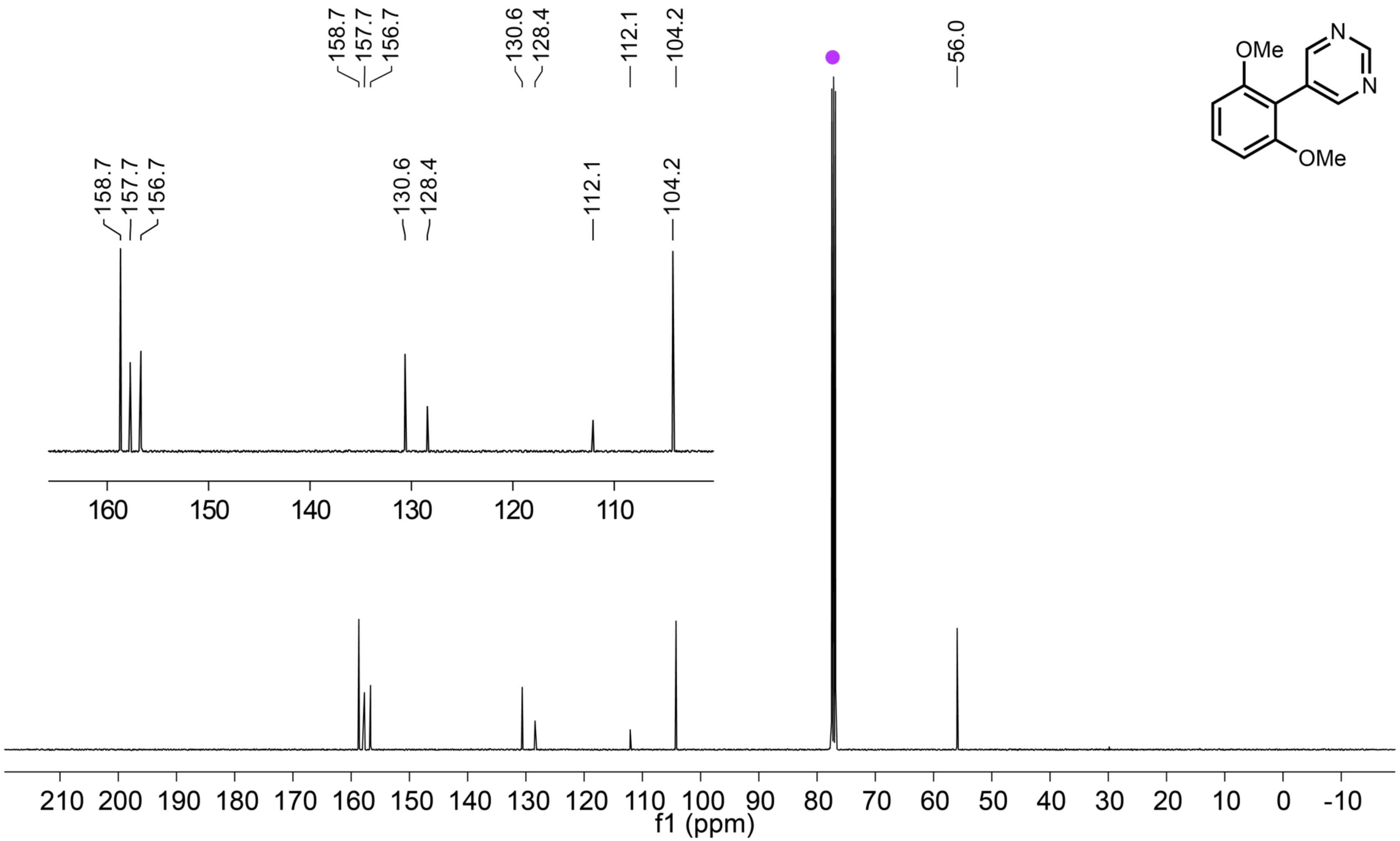

Figure S56. $^{13}C\{^{1}H\}$ NMR (100.67 MHz, $CDCl_3$) spectrum of compound **36**. Deuterated solvent (•).

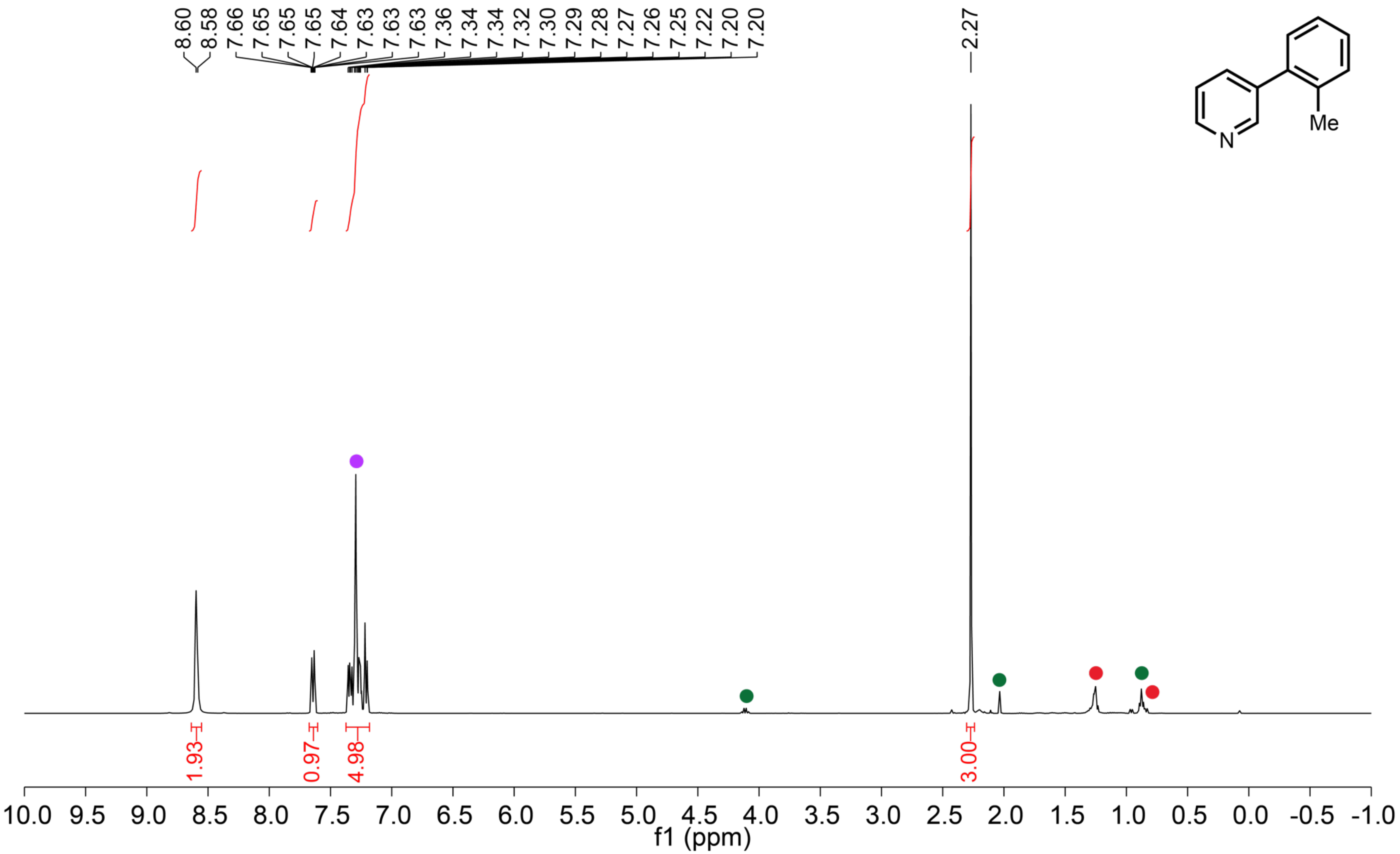

Figure S57. ^{1}H NMR (400.30 MHz, $CDCl_3$) spectrum of compound **37**. Residual proteo-solvent (•), EtOAc (•) and hexane (•).

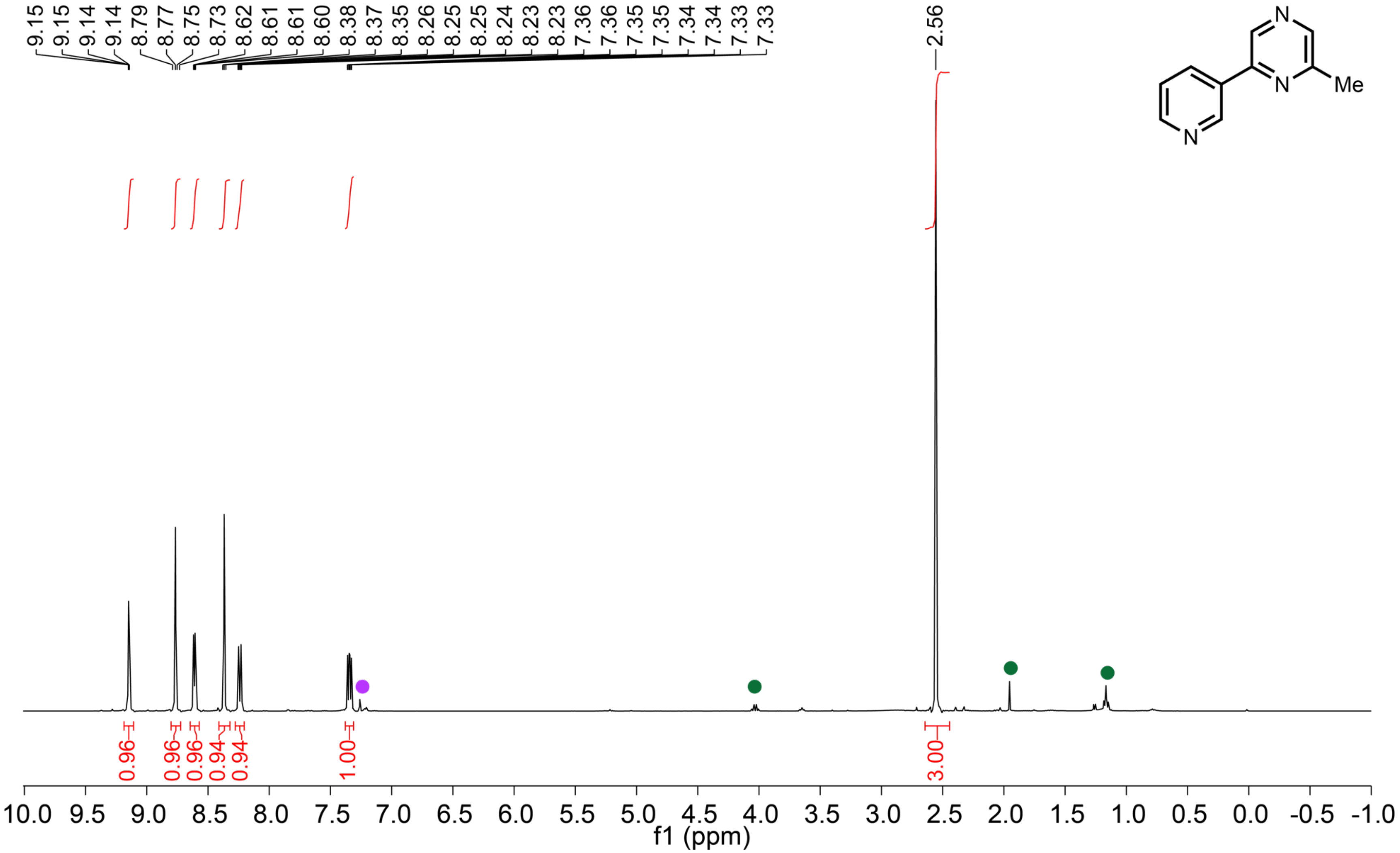

Figure S58. ^{1}H NMR (400.30 MHz, $CDCl_3$) spectrum of compound **38**. Residual proteo-solvent (•) and EtOAc (•).

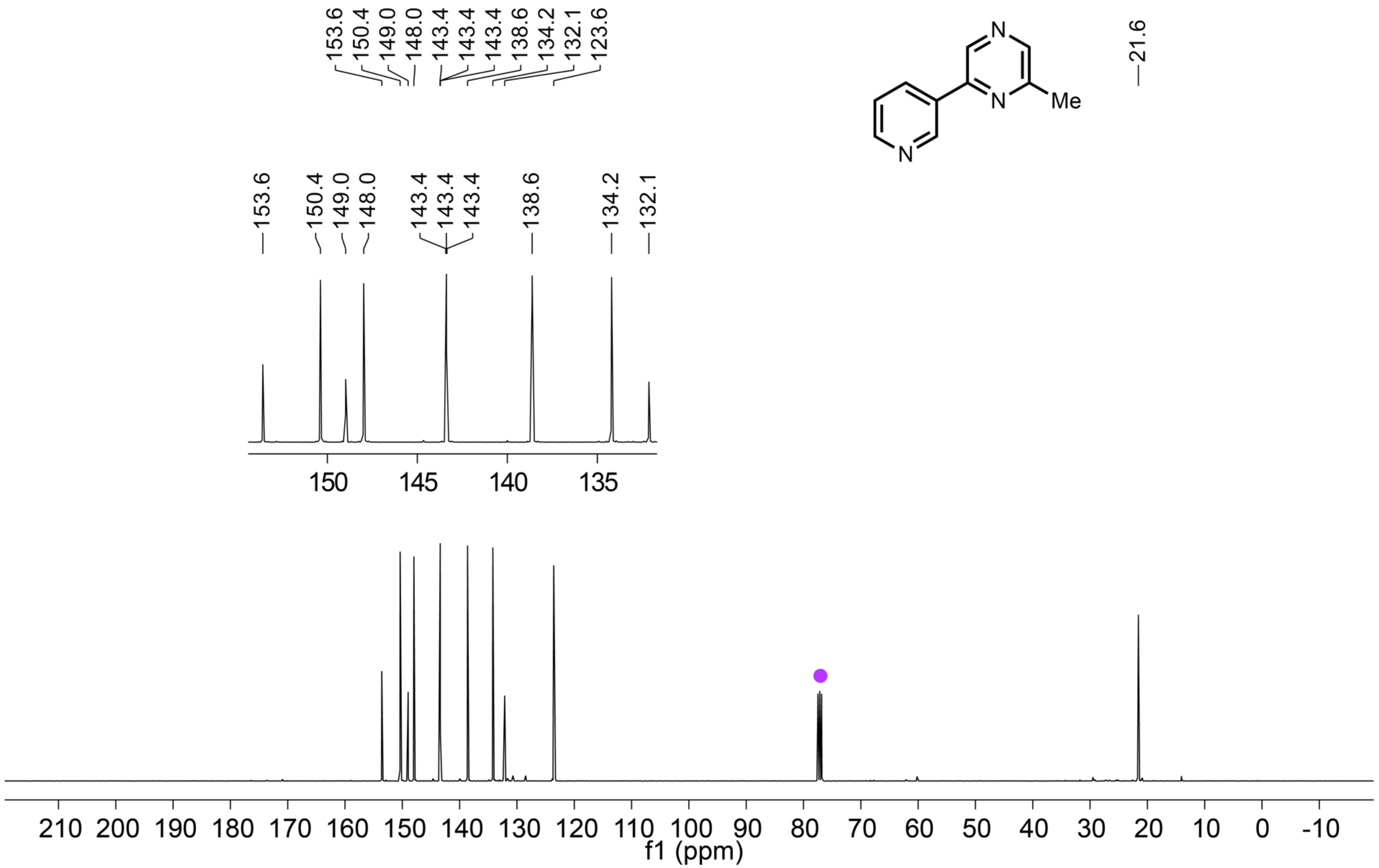

Figure S59. $^{13}C\{^{1}H\}$ NMR (100.67 MHz, $CDCl_3$) spectrum of compound **38**. Deuterated solvent (•).

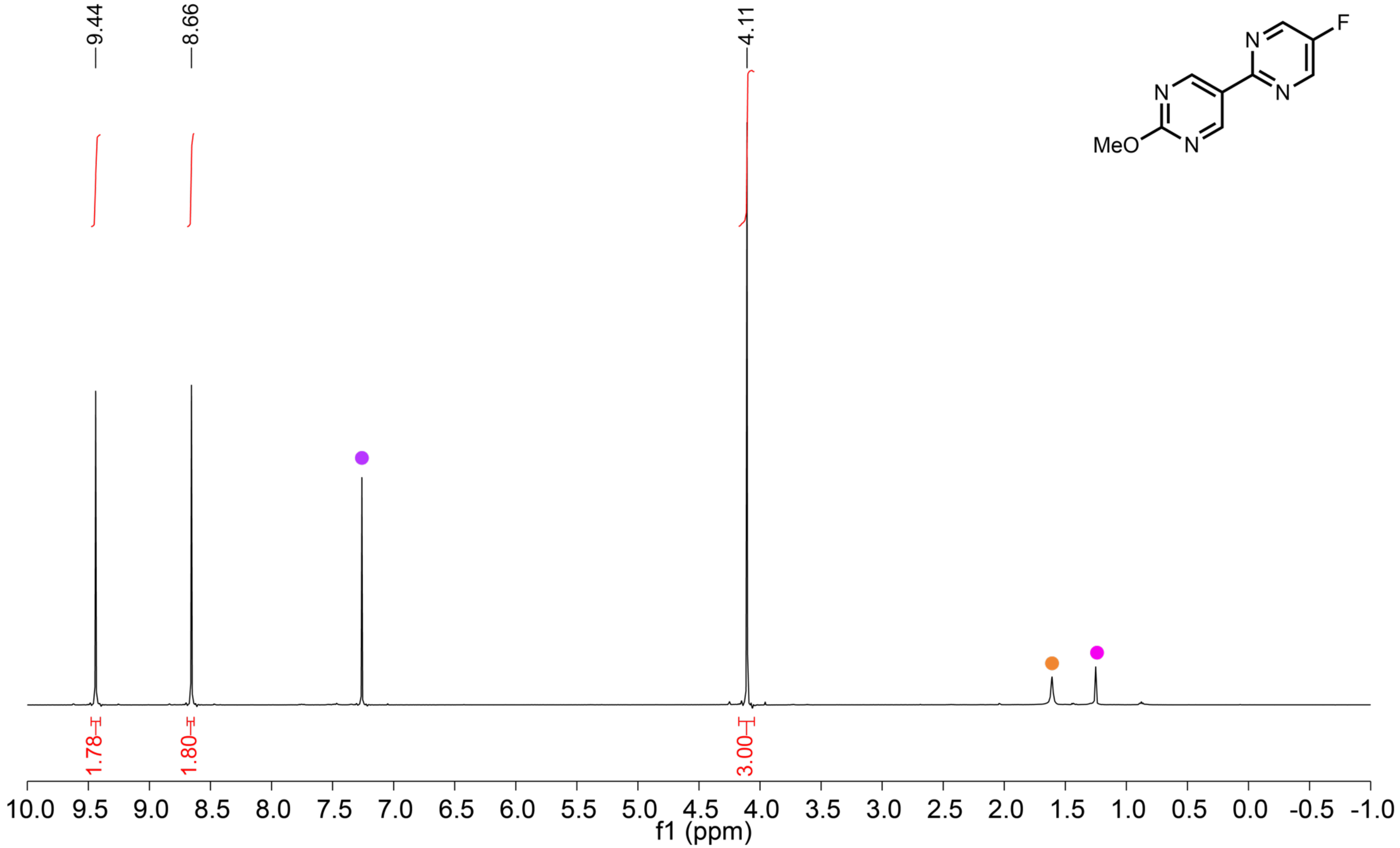

Figure S60. ^{1}H NMR (500.20 MHz, $CDCl_3$) spectrum of compound **39**. Residual proteo-solvent (•), H_2O (•) and grease (•).

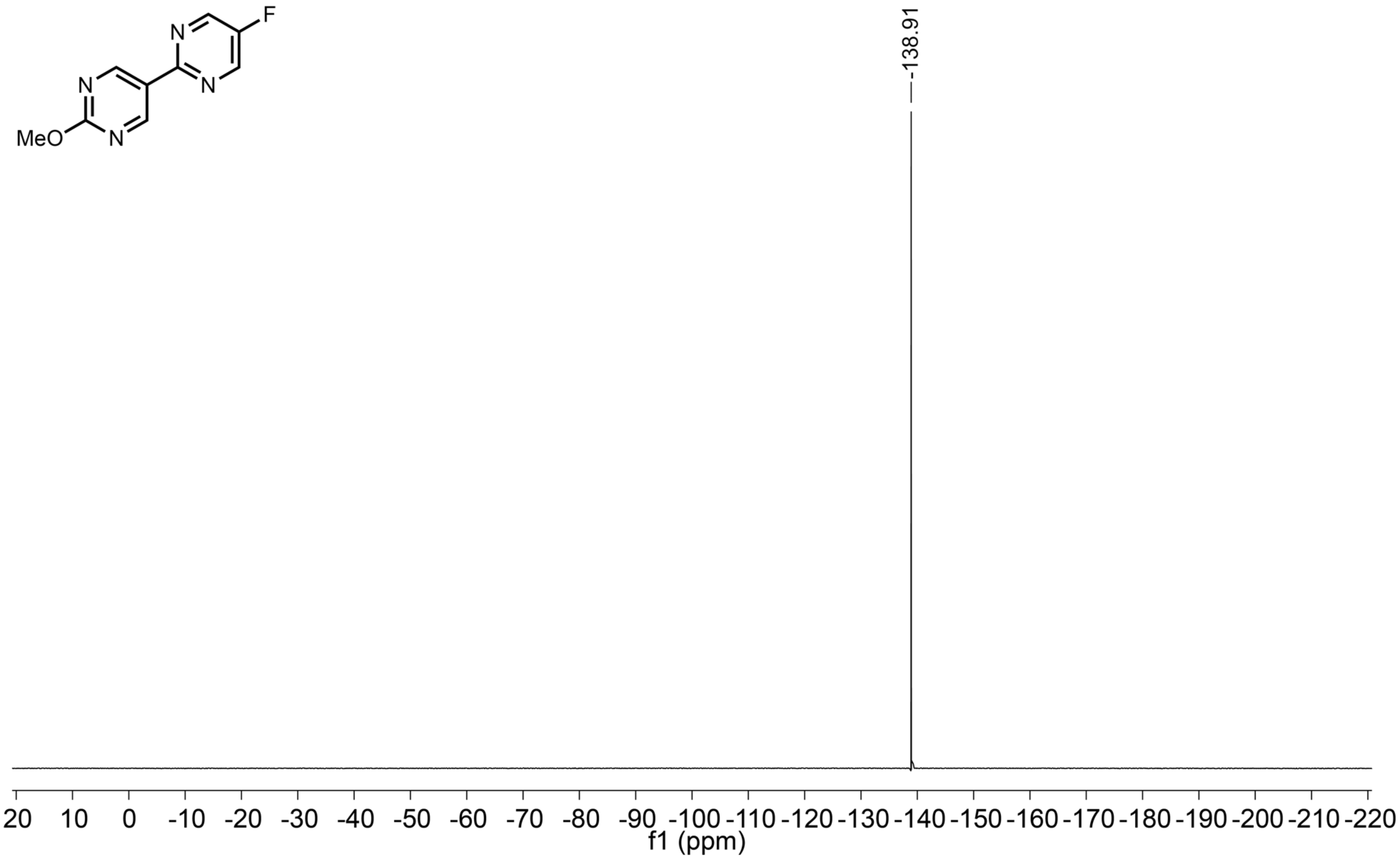

Figure S61. $^{19}F\{^1H\}$ NMR (470.61 MHz, $CDCl_3$) spectrum of compound **39**.

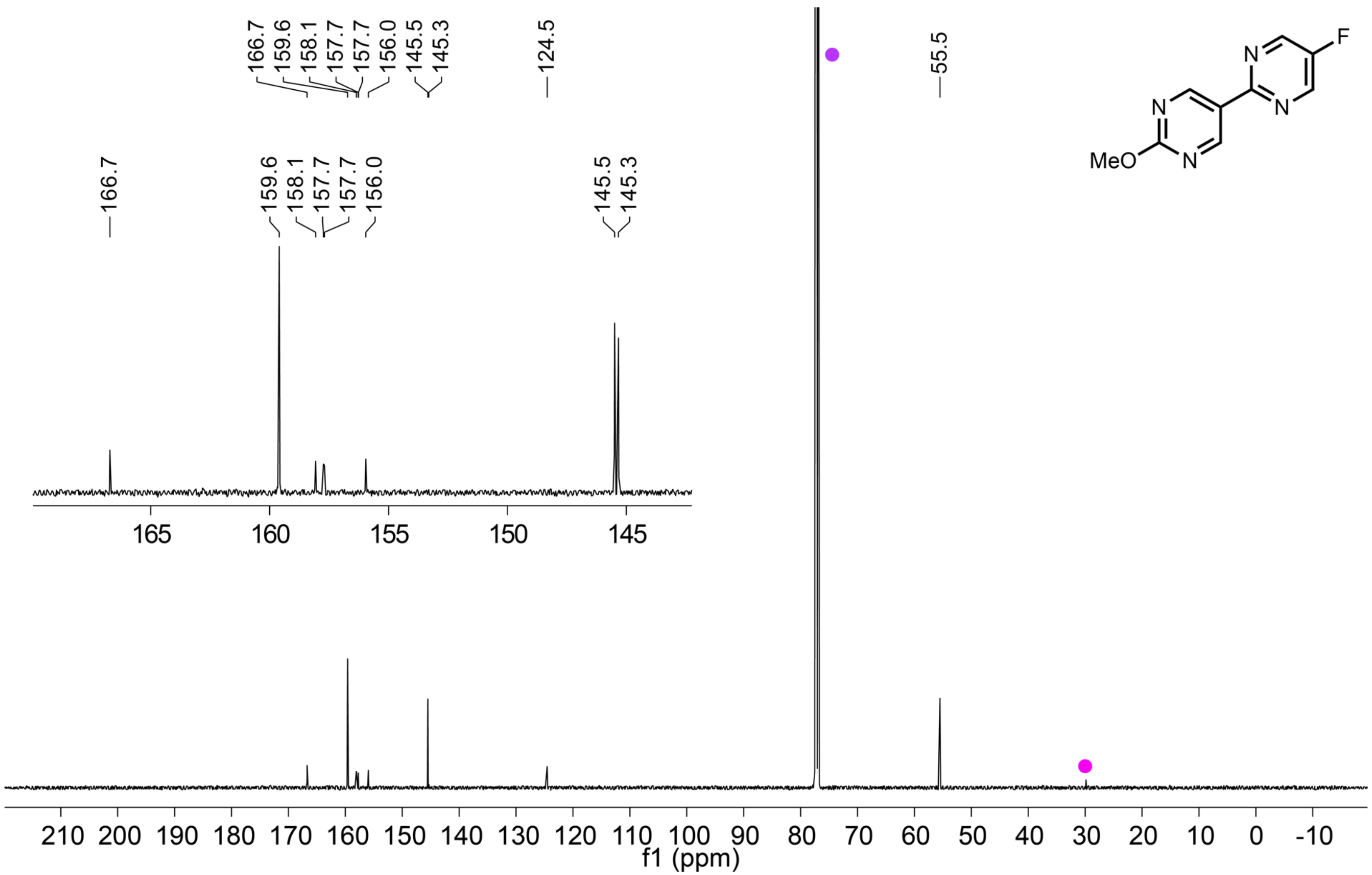

Figure S62 $^{13}C\{^{1}H\}$ NMR (125.79 MHz, $CDCl_3$) spectrum of compound **39**. Deuterated solvent (•) and grease (•).

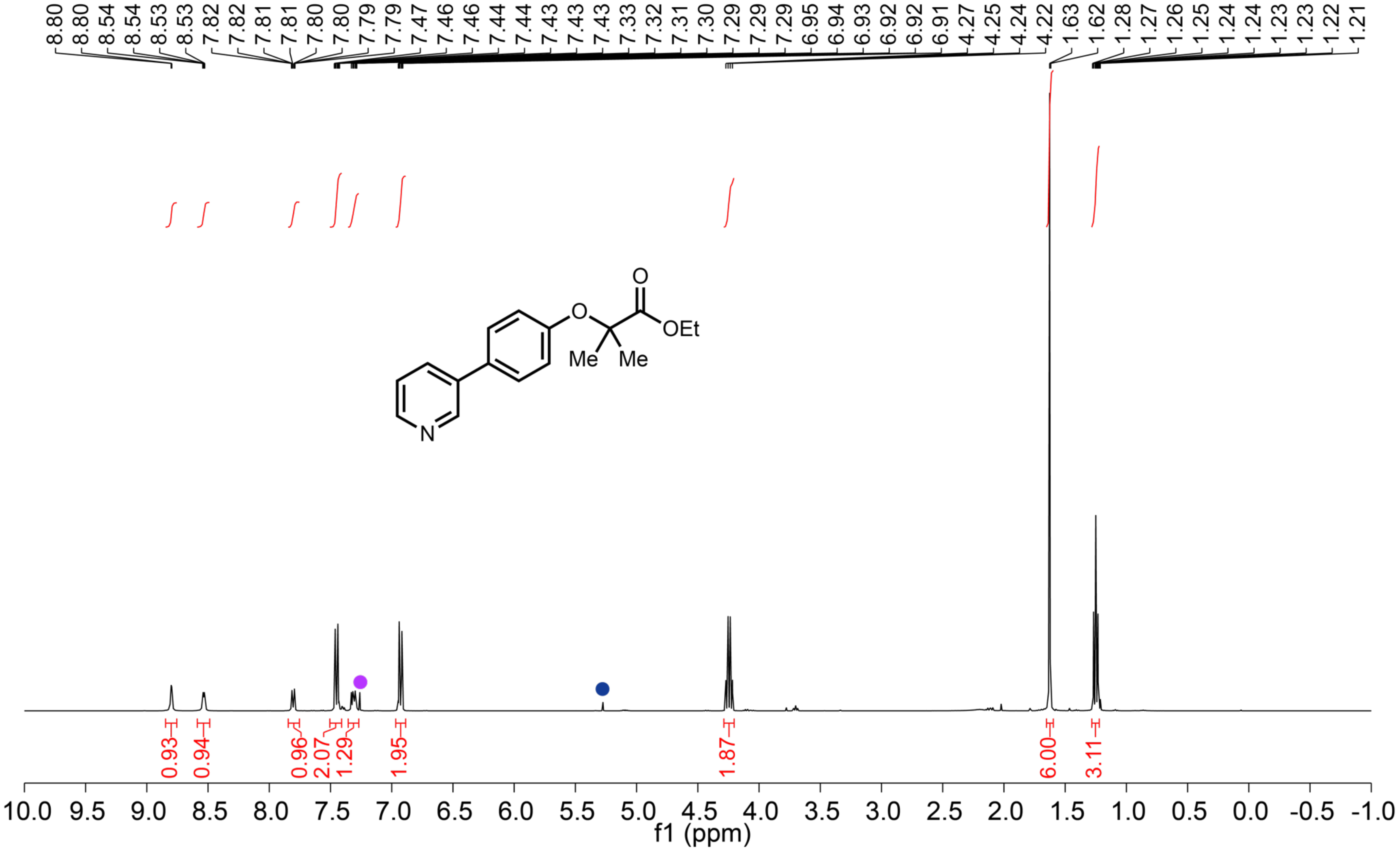

Figure S63. ^{1}H NMR (400.30 MHz, $CDCl_3$) spectrum of compound **40**. Residual proteo-solvent (•) and DCM (•)

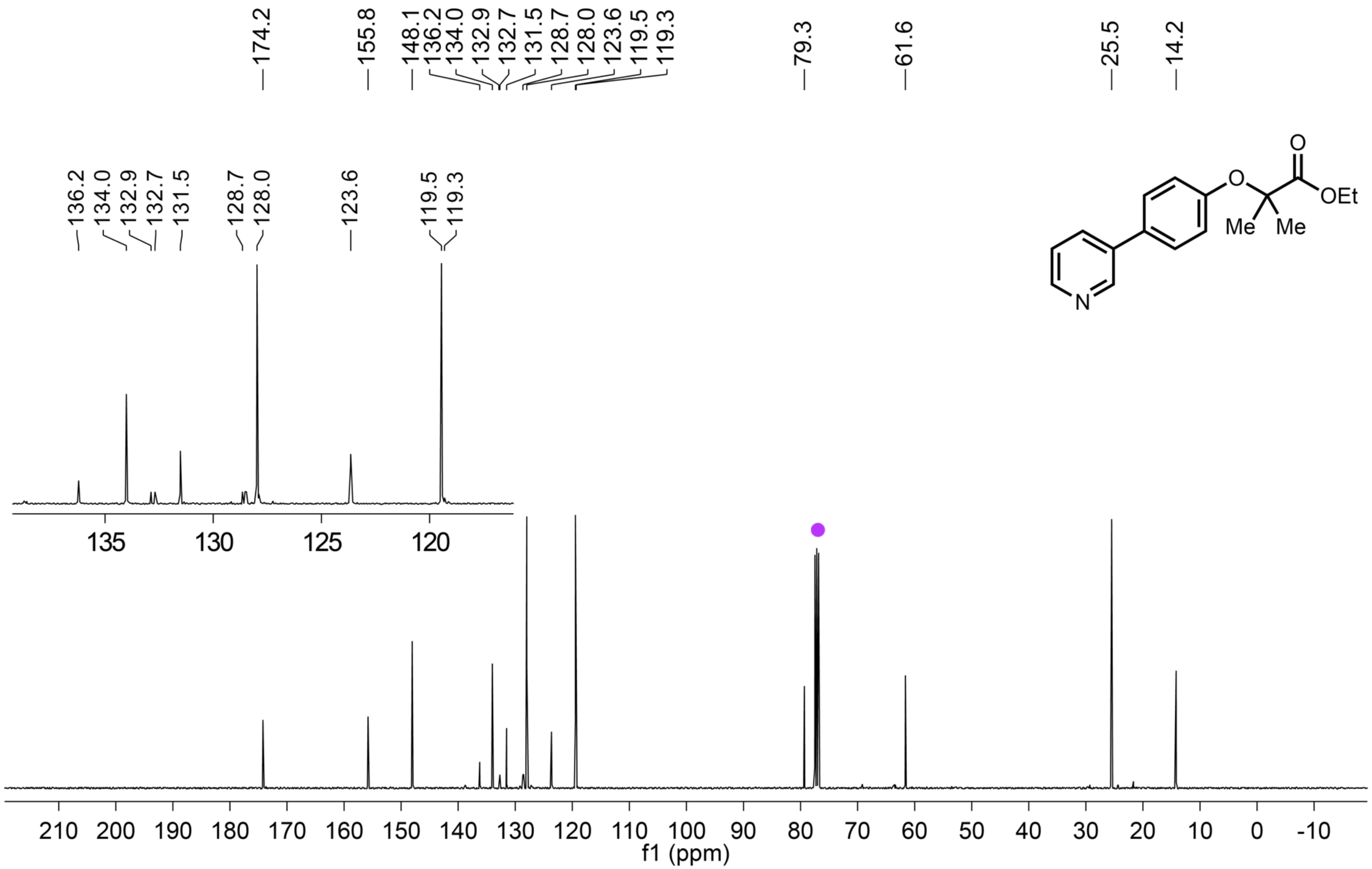

Figure S64 $^{13}C\{^{1}H\}$ NMR (100.67 MHz, $CDCl_3$) spectrum of compound **40**. Deuterated solvent (•).

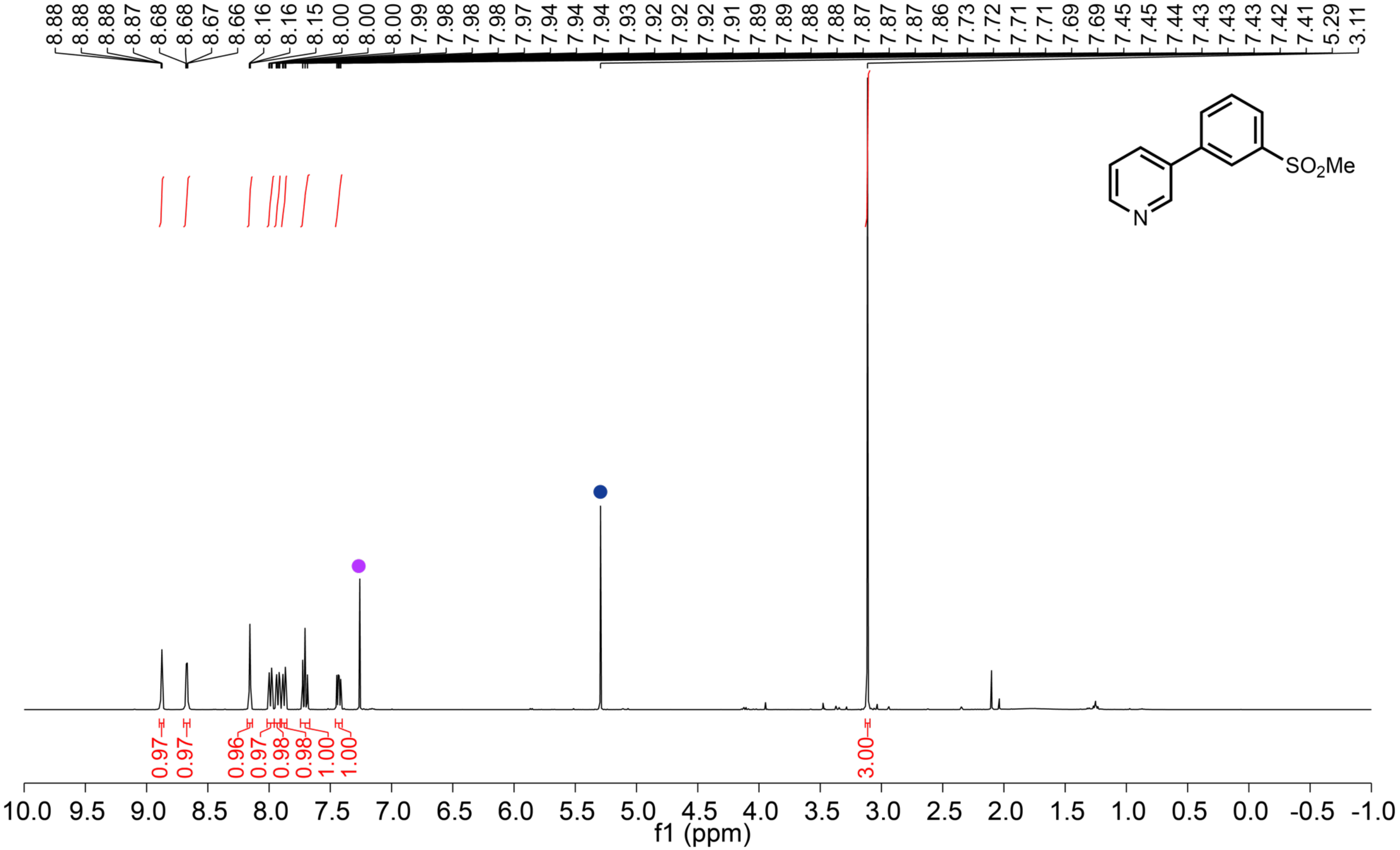

Figure S65. ^{1}H NMR (400.30 MHz, $CDCl_3$) spectrum of compound **41**. Residual proteo-solvent (•) and DCM (•).

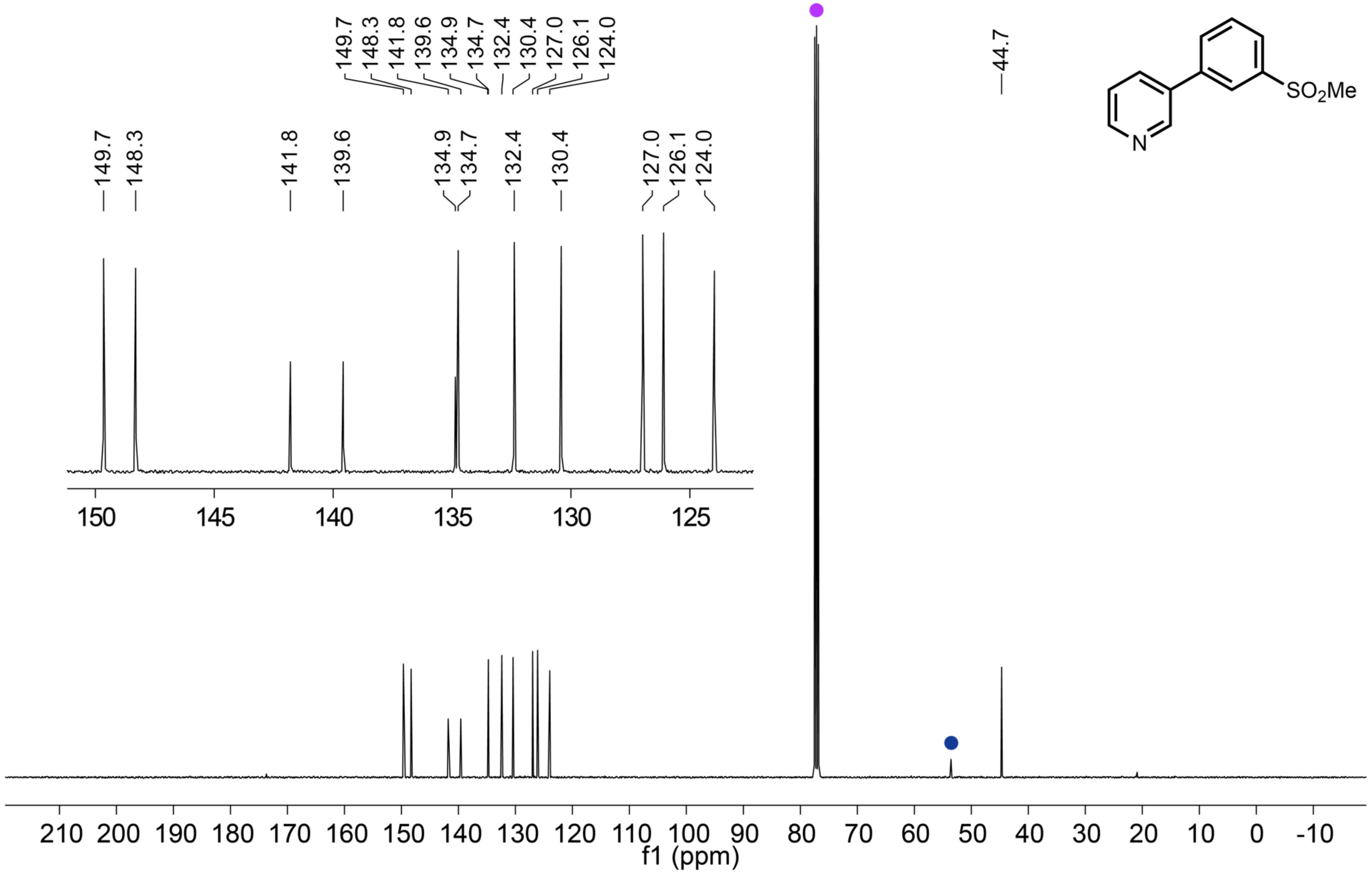

Figure S66. $^{13}C\{^{1}H\}$ NMR (100.67 MHz, $CDCl_3$) spectrum of compound **41**. Deuterated solvent (•) and DCM (•).

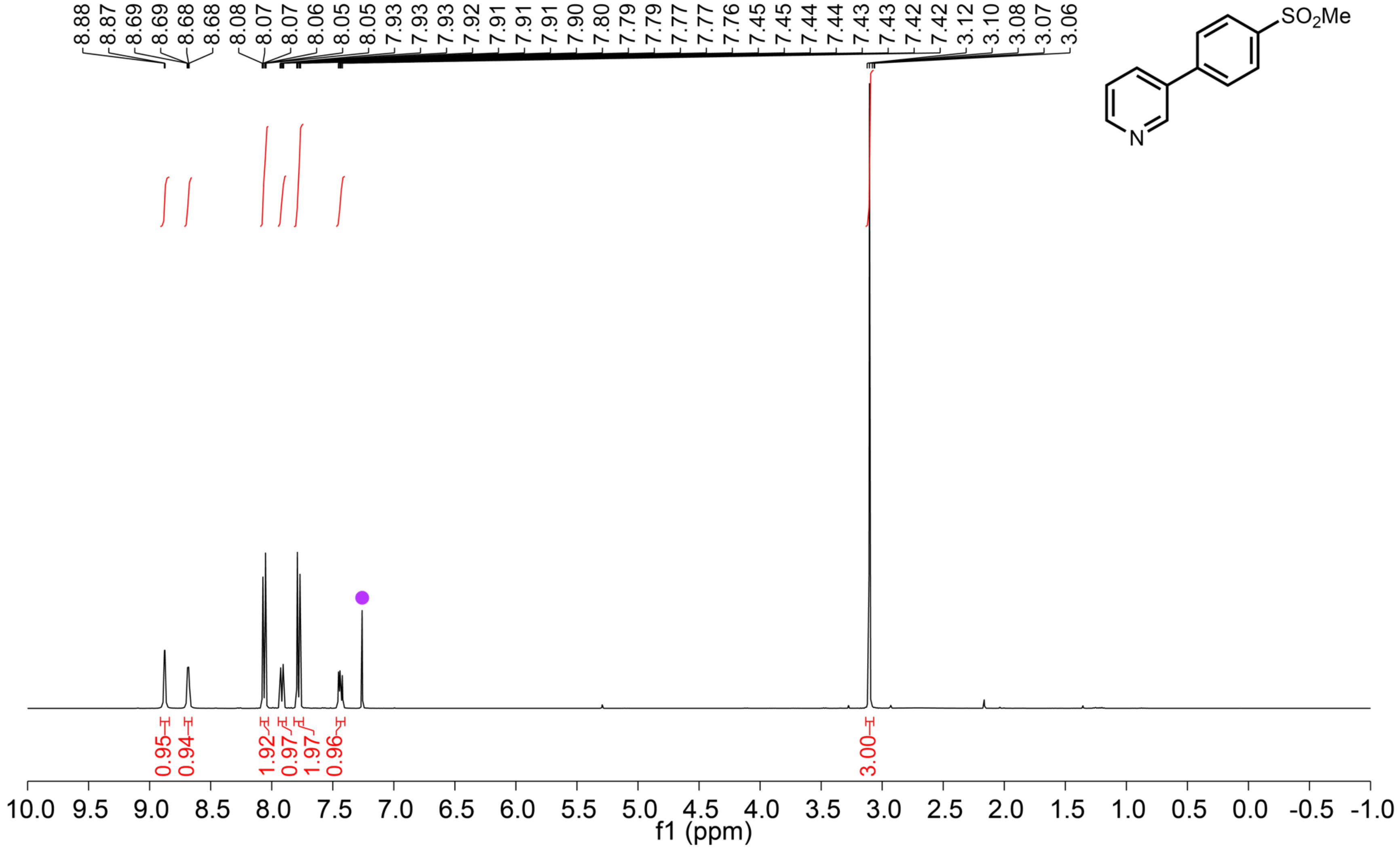

Figure S67. ^{1}H NMR (400.30 MHz, $CDCl_3$) spectrum of compound **42**. Residual proteo-solvent (•).

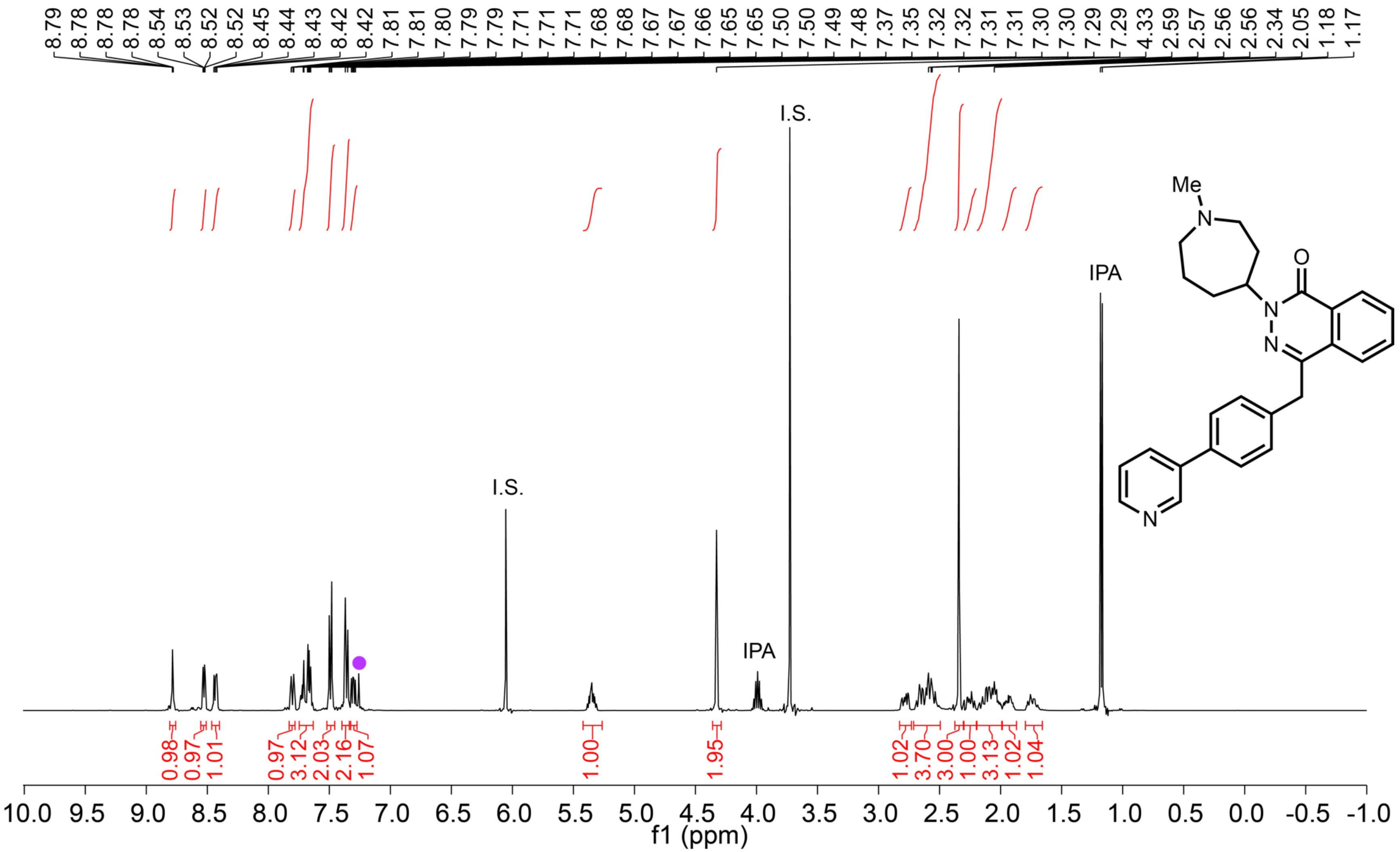

Figure S68. ^1H NMR (400.30 MHz, $CDCl_3$) spectrum of the crude of compound **43** with internal standard, 1,3,5-trimethoxylbenezene (I.S.). Residual proteo-solvent (●). IPA = *i*PrOH.

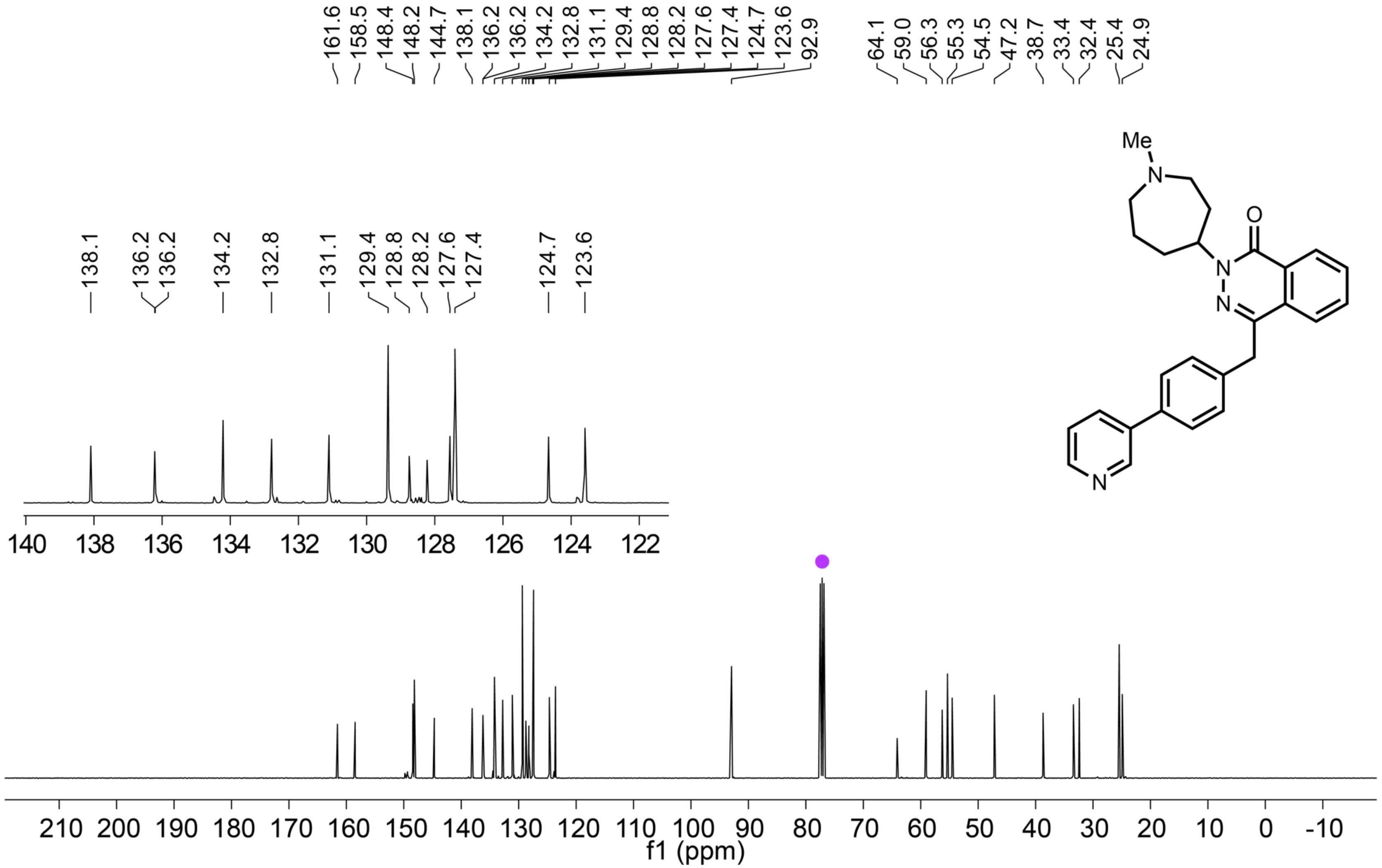

Figure S69. ^{13}C{^{1}H} NMR (100.67 MHz, $CDCl_3$) spectrum of the crude of compound **43** with internal standard, 1,3,5-trimethoxylbenezene (I.S.). Deuterated solvent (•). The ^{13}C signals include 3 signals due to I.S. and 2 signals due to *i*PrOH, which are not labeled.

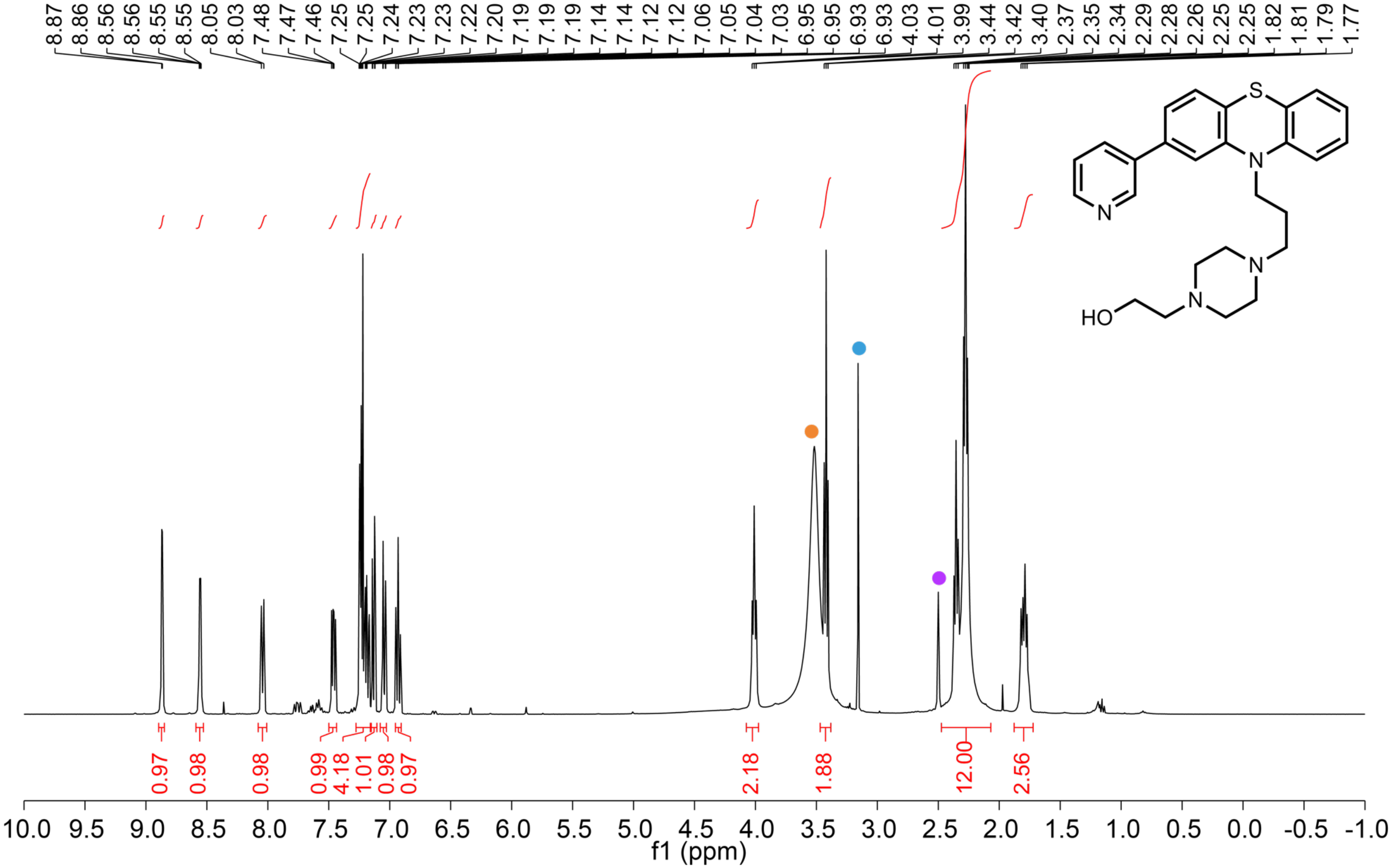

Figure S70. ^{1}H NMR (400.30 MHz, DMSO-d_6) spectrum of compound **44**. H_2O (●), MeOH (●) and residual proteo-solvent (●). The product seems not to be stable in the solvent, some decomposition product is observed overtime.

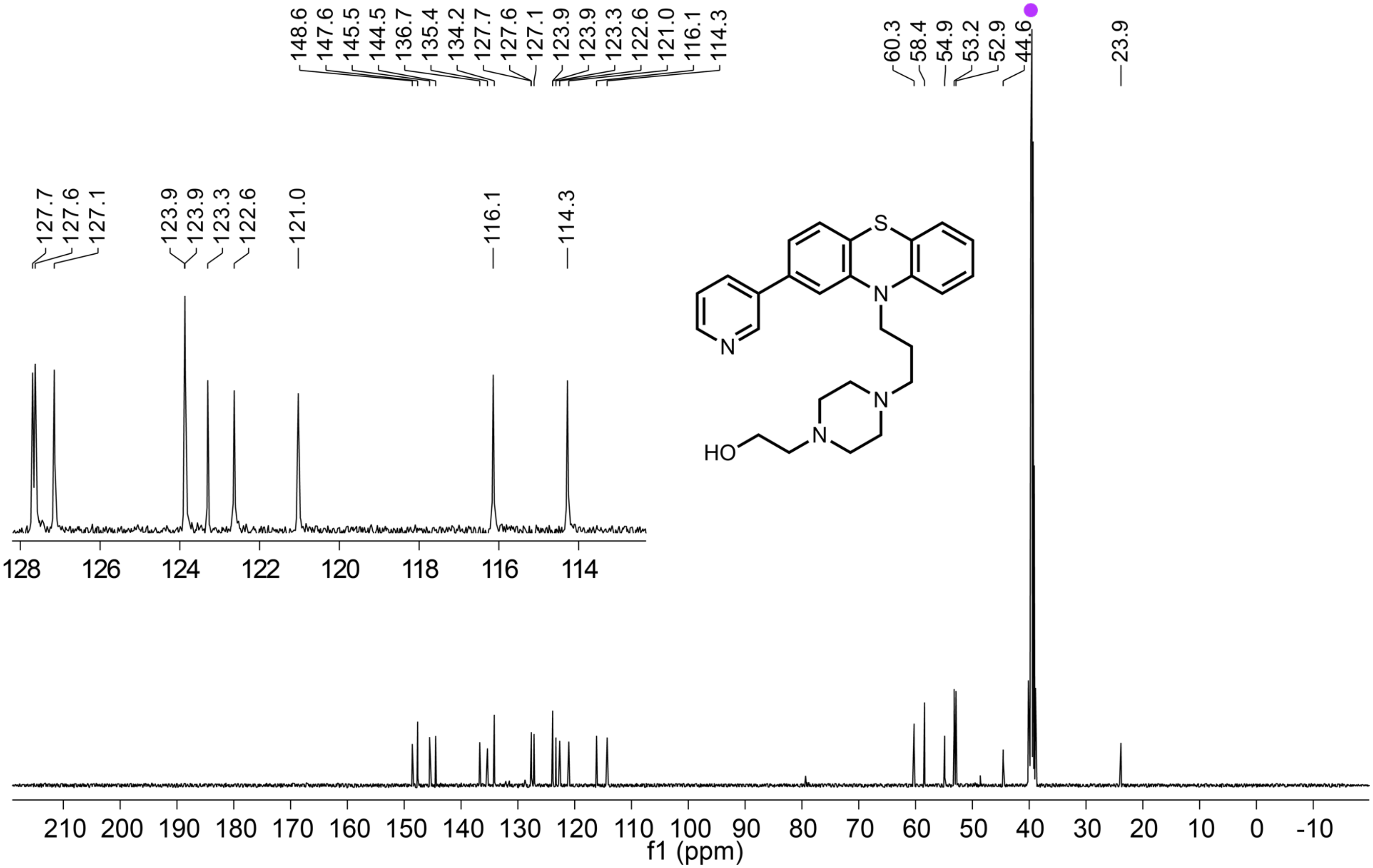

Figure S71. $^{13}C\{^1H\}$ NMR (100.67 MHz, DMSO-d_6) spectrum of compound **44**. Deuterated solvent (•). The product seems not to be stable in the solvent, some decomposition product is observed overtime.

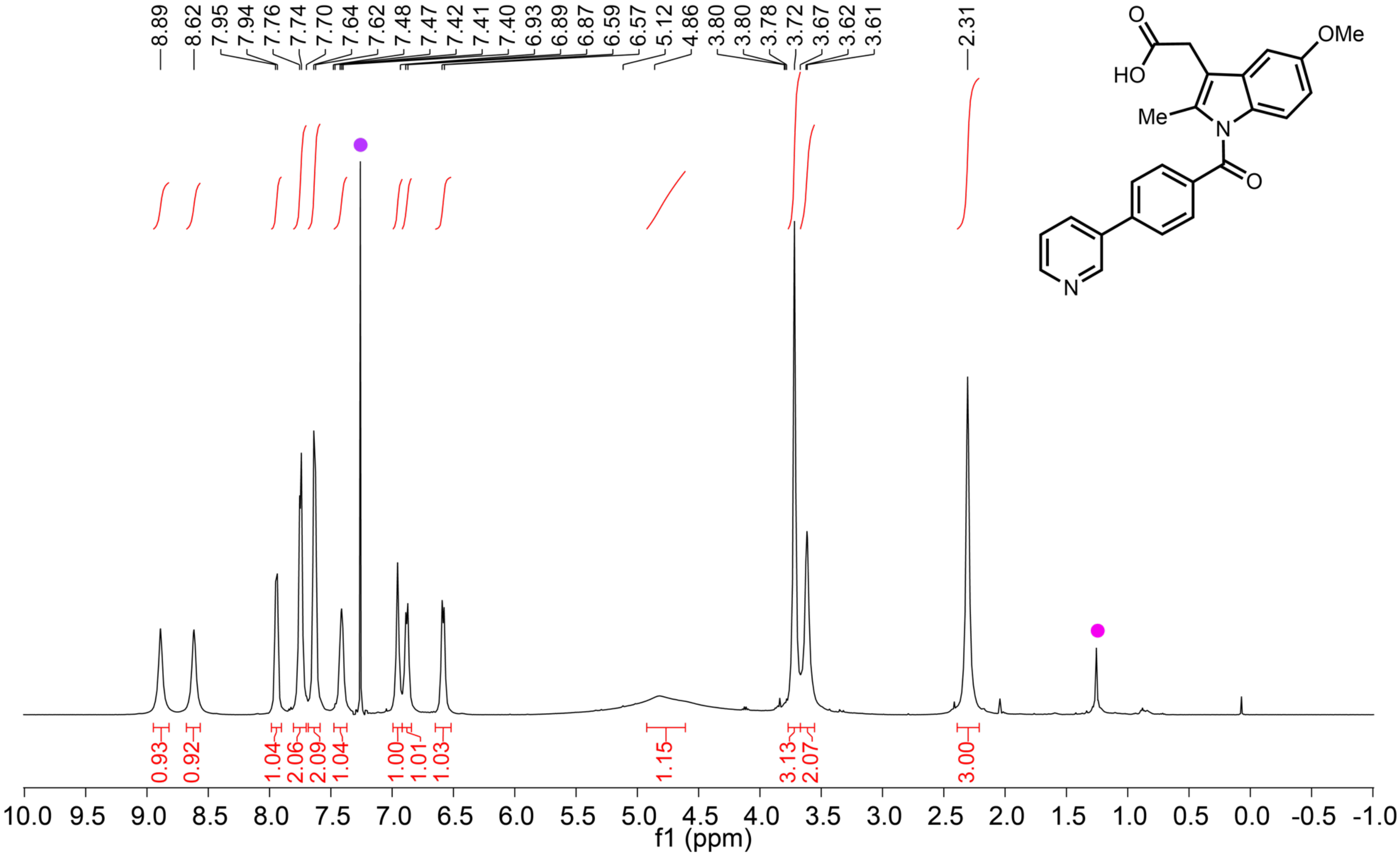

Figure S72. ^{1}H NMR (400.30 MHz, $CDCl_3$) spectrum of compound **45**. Residual proteo-solvent (•) and grease (•).

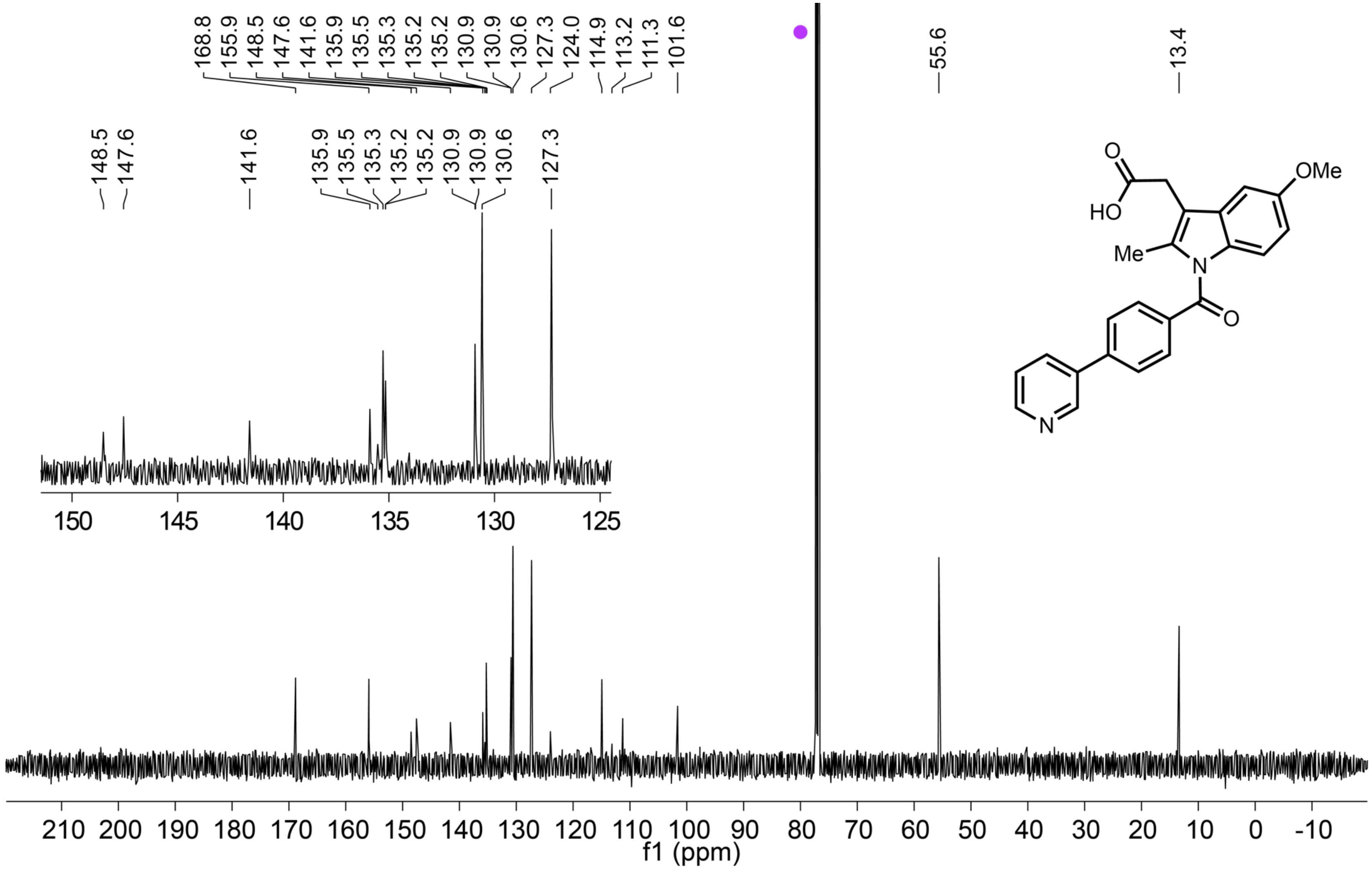

Figure S73. $^{13}C\{^{1}H\}$ NMR (100.67 MHz, $CDCl_3$) spectrum of compound **45**. Deuterated solvent (•).

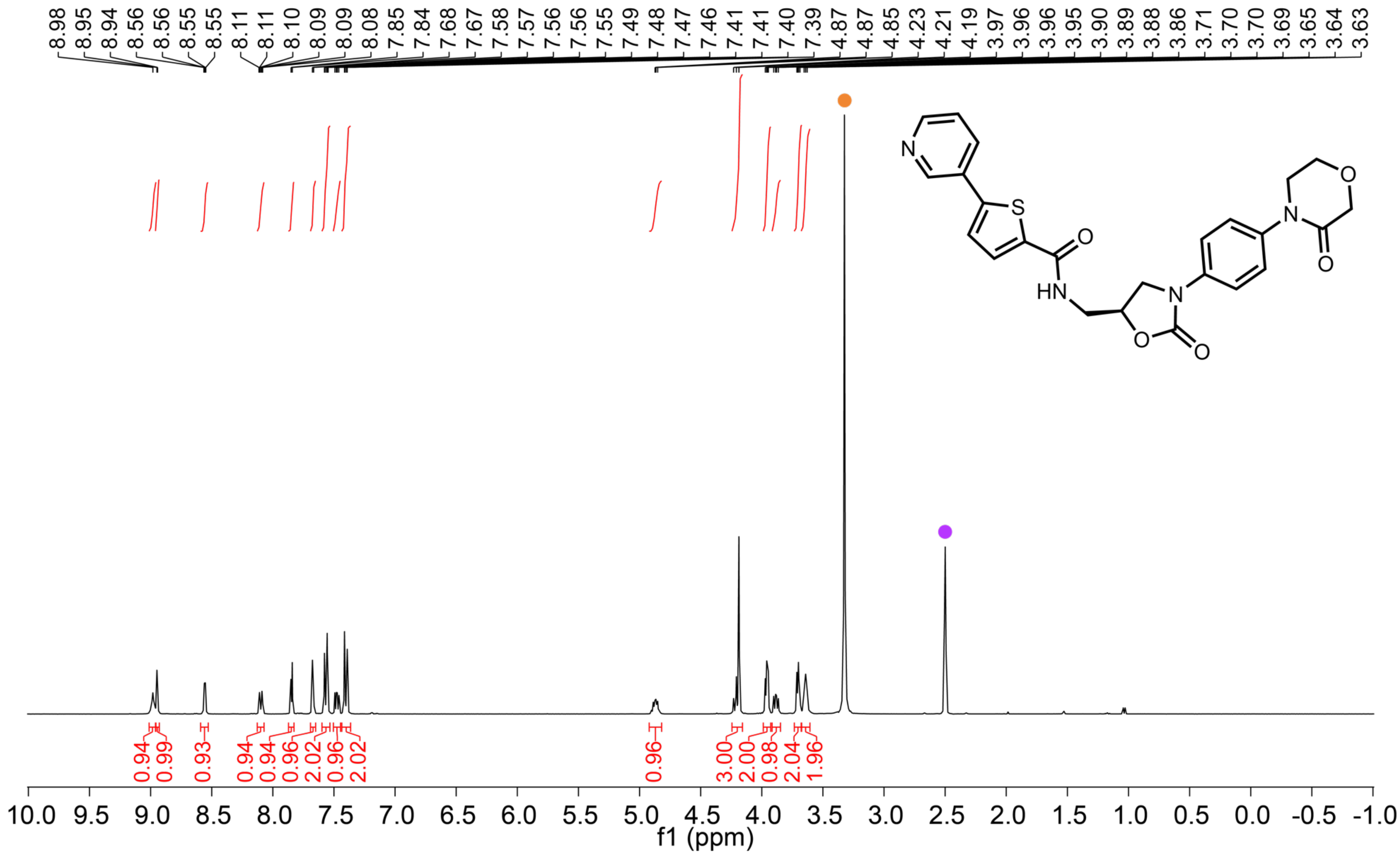

Figure S74. ^{1}H NMR (400.30 MHz, DMSO-d_6) spectrum of compound **46**. Residual proteo-solvent (●) and H_2O (●).

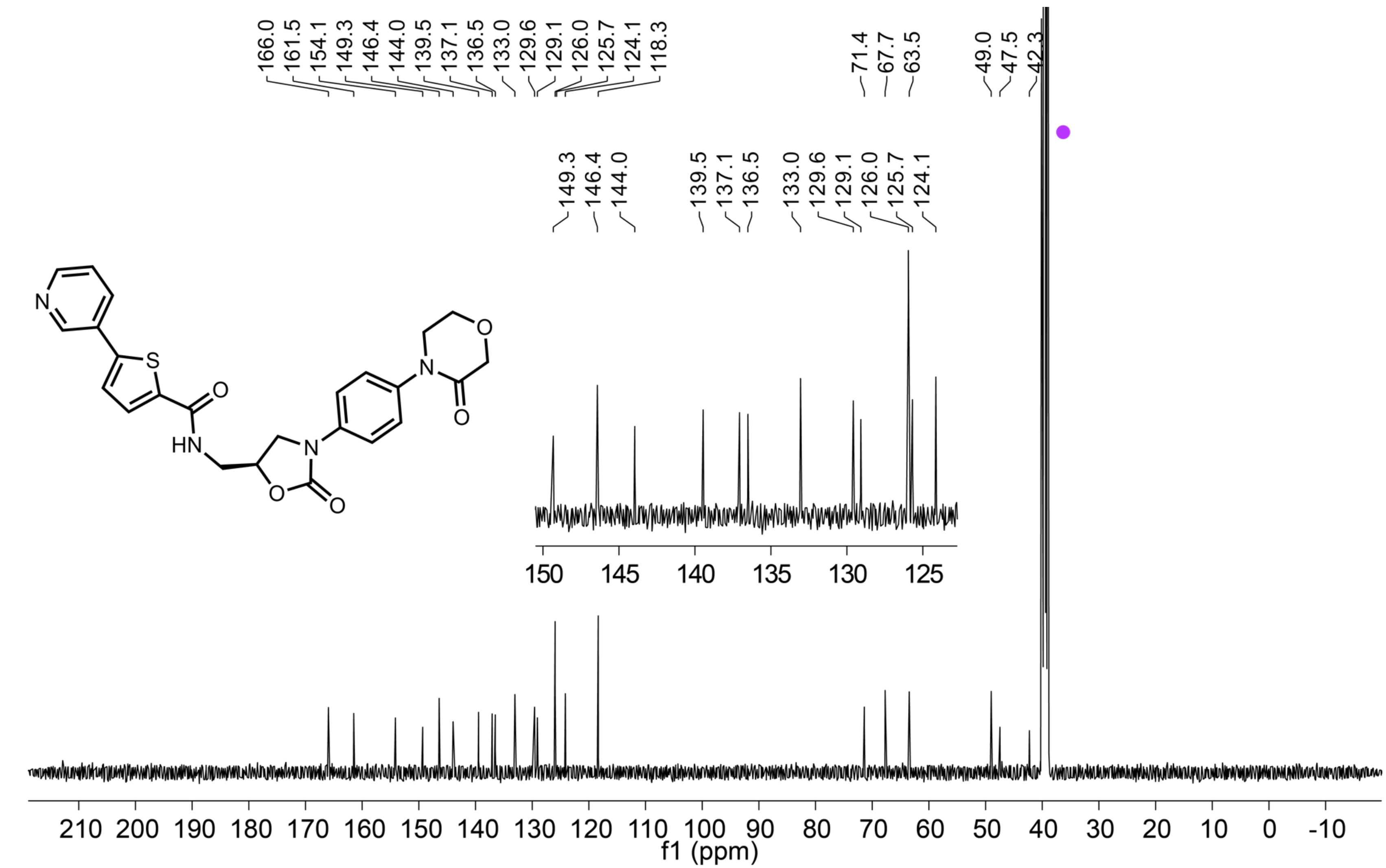

Figure S75. $^{13}C\{^{1}H\}$ NMR (100.67 MHz, $CDCl_3$) spectrum of compound **46**. Deuterated solvent (•).

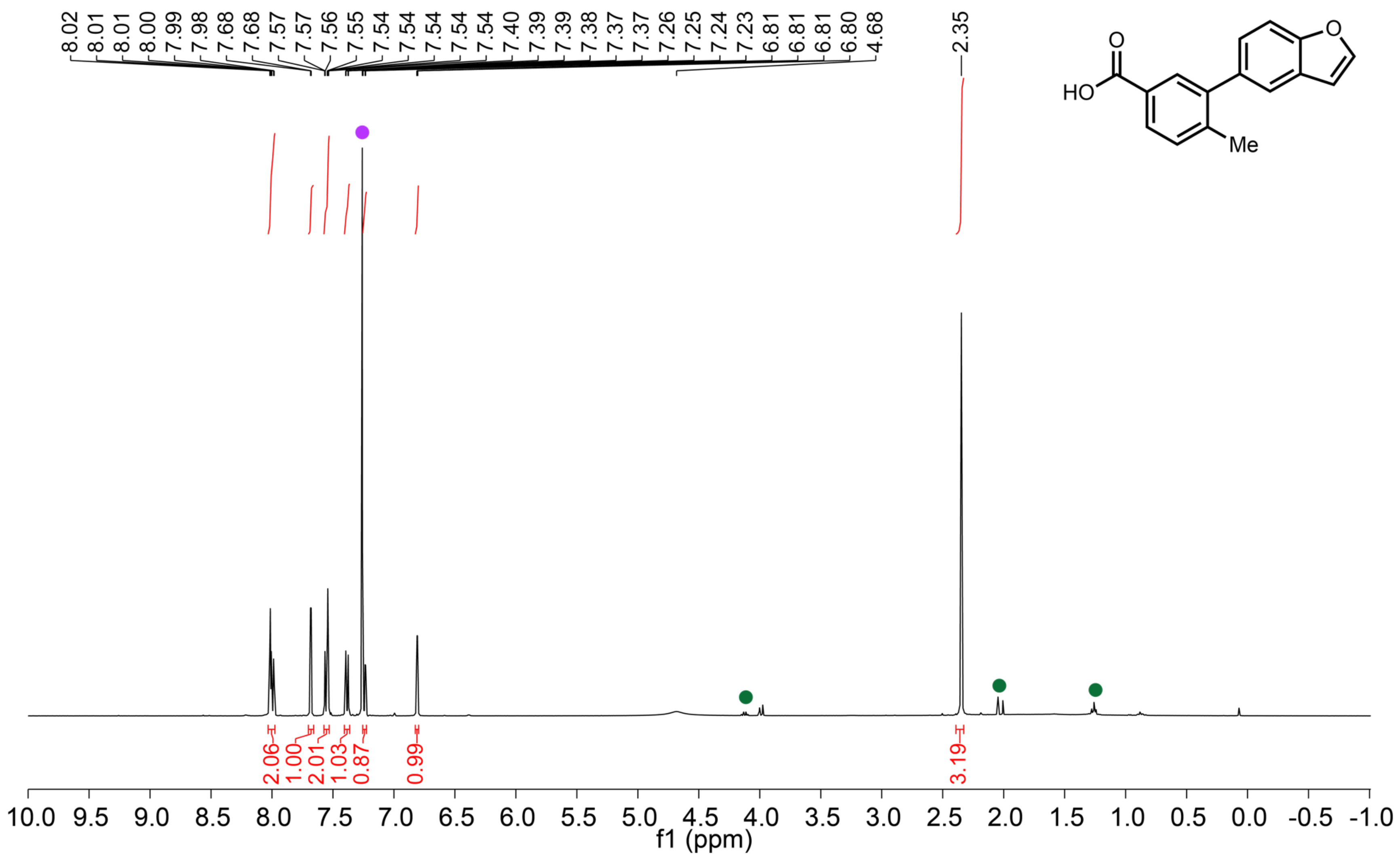

Figure S76. ^{1}H NMR (400.30 MHz, $CDCl_3$) spectrum of compound **47**. Residual proteo-solvent (•) and EtOAc (•). The broad signal at around 4.7 ppm is presumably due to the CO_2H associated with H_2O.

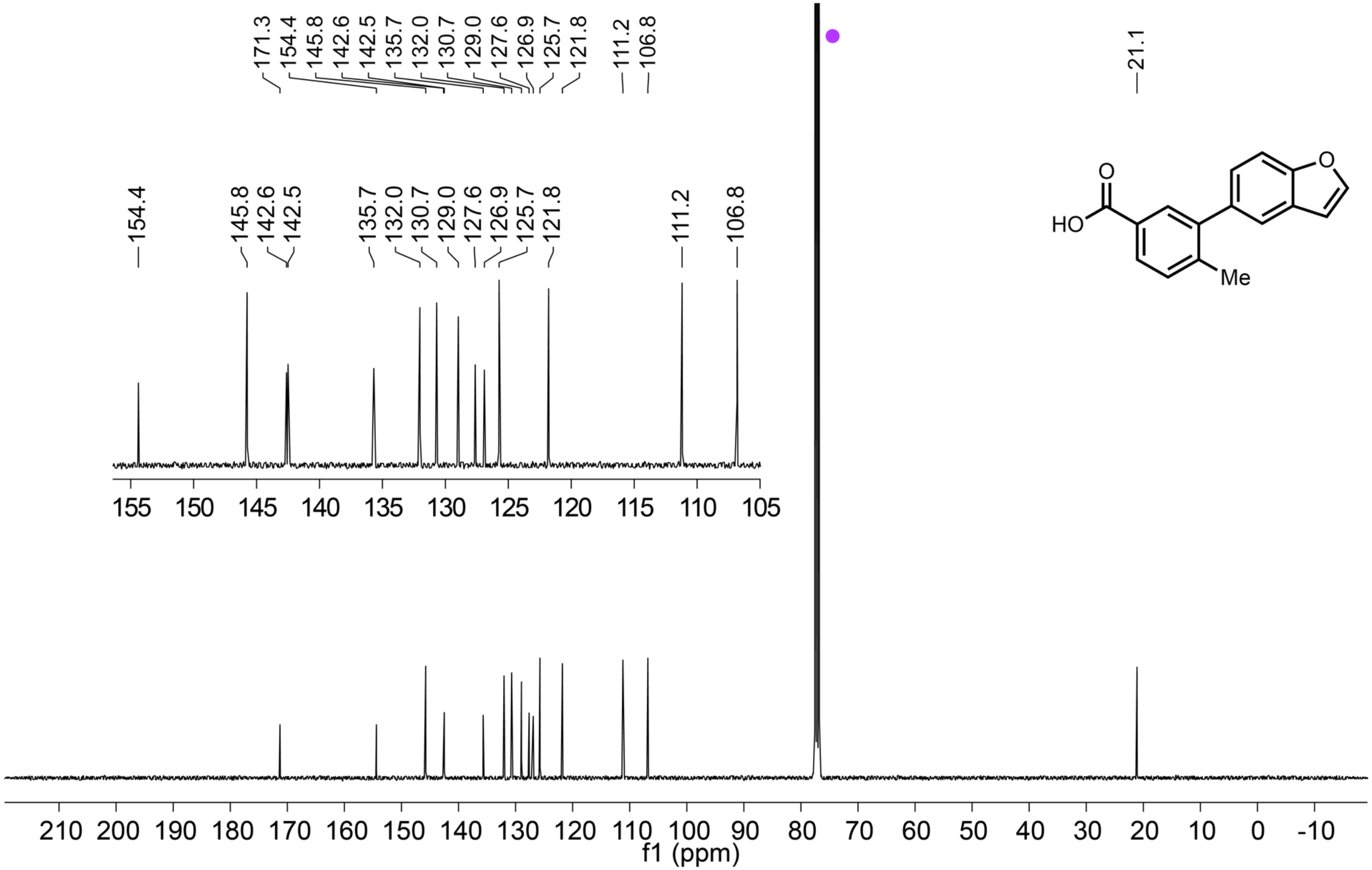

Figure S77. $^{13}C\{^1H\}$ NMR (100.67 MHz, $CDCl_3$) spectrum of compound **47**. Deuterated solvent (•).

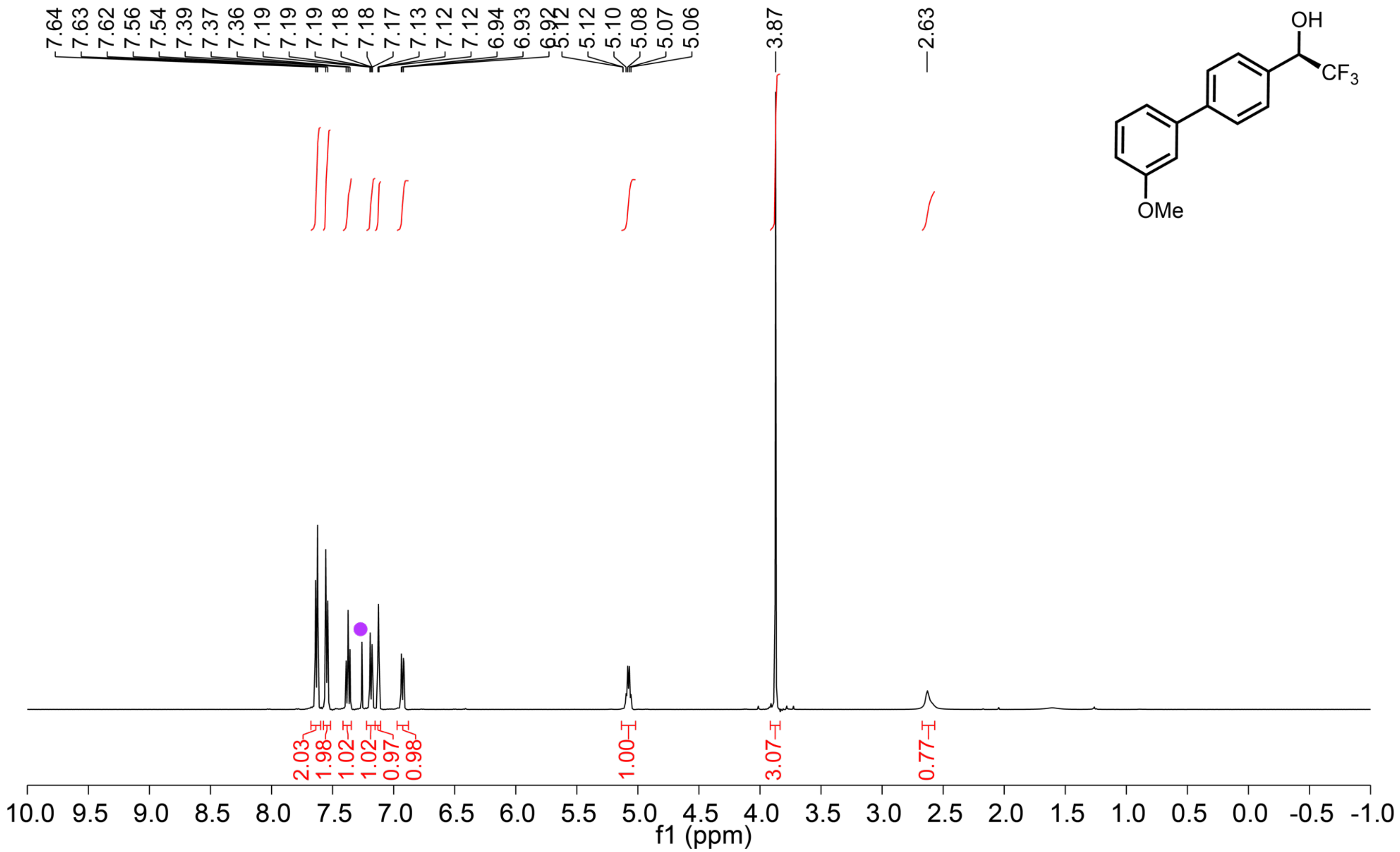

Figure S78. ^{1}H NMR (500.20 MHz, $CDCl_3$) spectrum of compound **48**. Residual proteo-solvent (•).

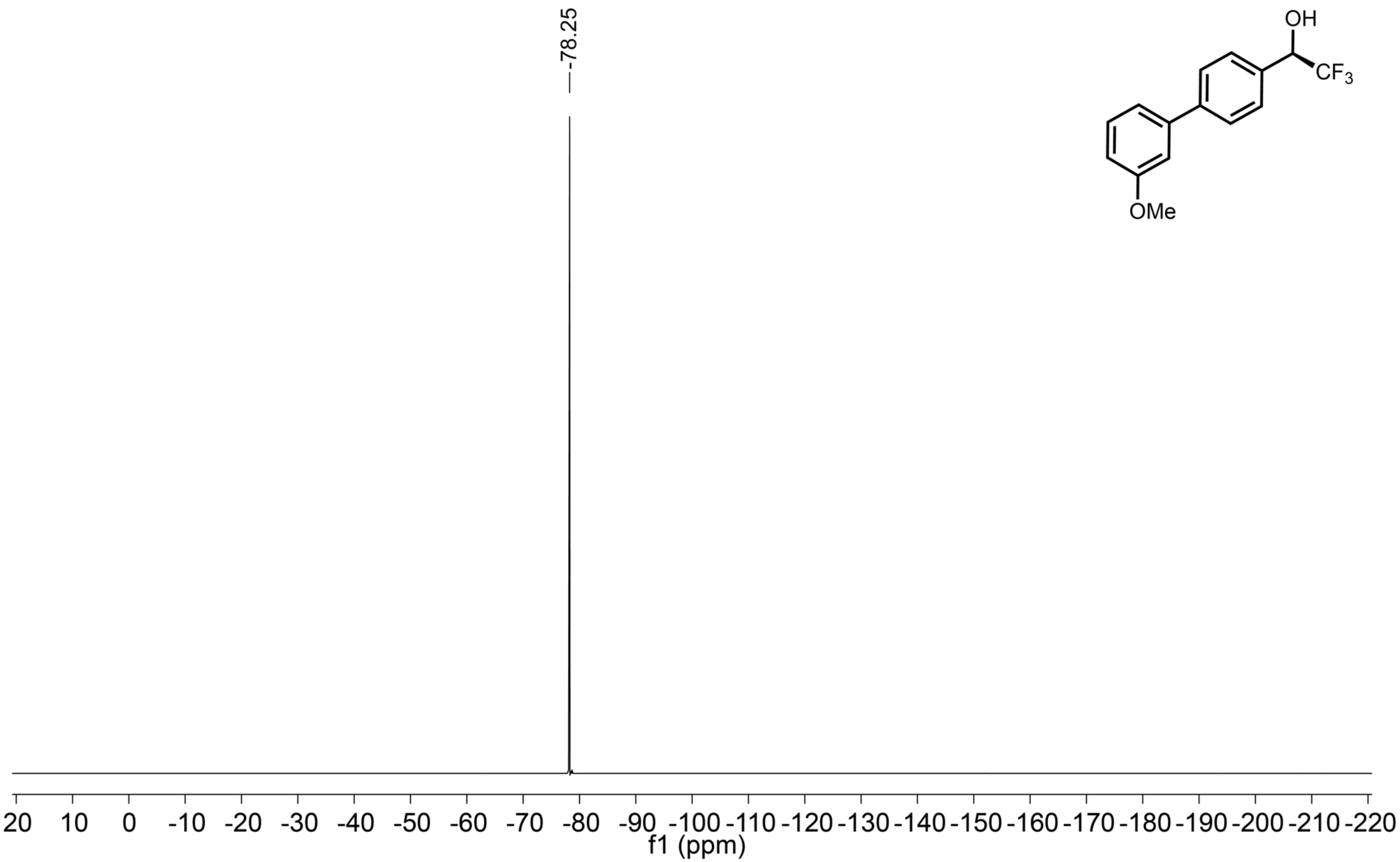

Figure S79. $^{19}F\{^1H\}$ NMR (470.61 MHz, $CDCl_3$) spectrum of compound **48**.

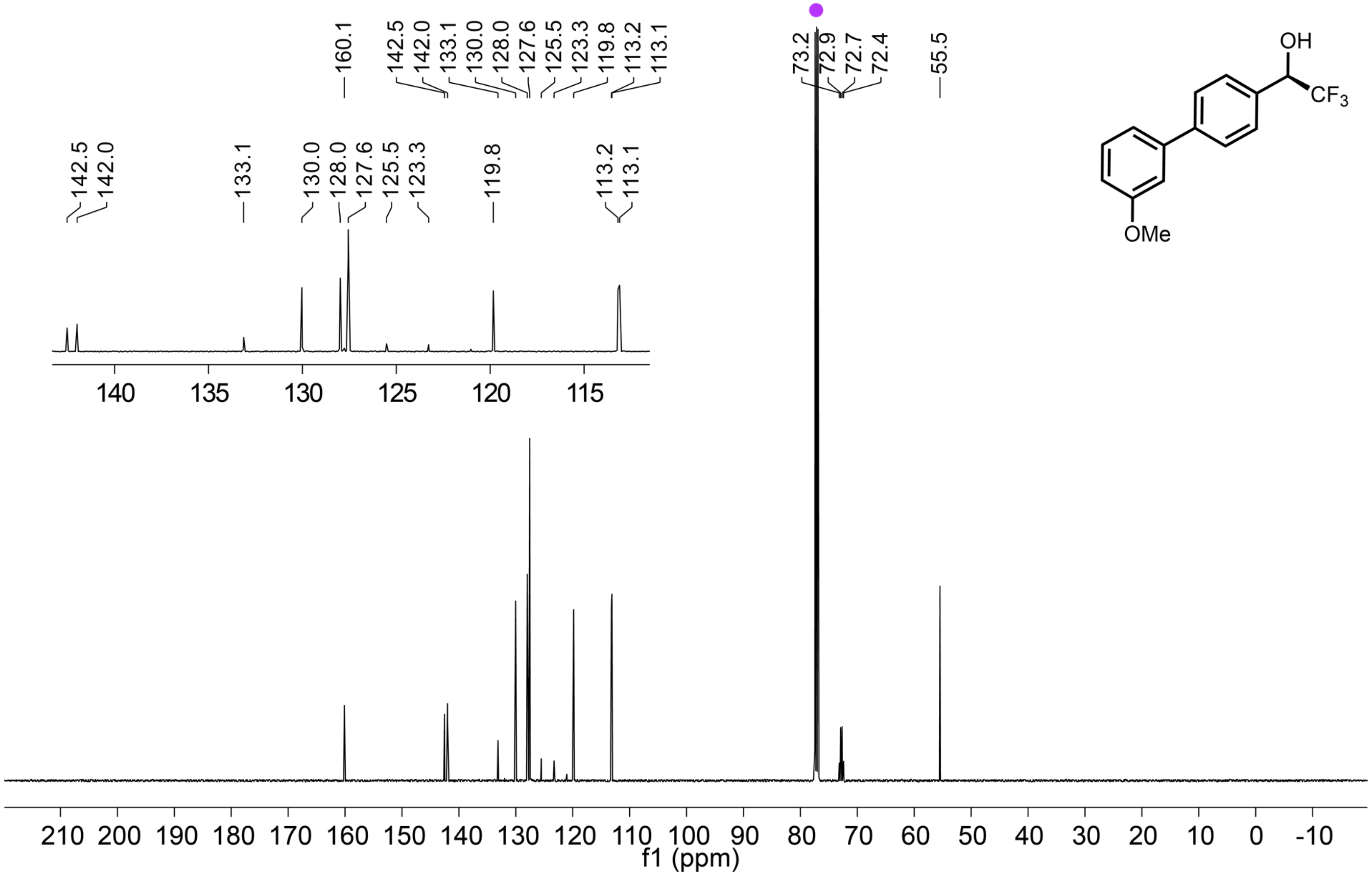

Figure S80. $^{13}C\{^{1}H\}$ NMR (125.79 MHz, $CDCl_3$) spectrum of compound **48**. Deuterated solvent (•).

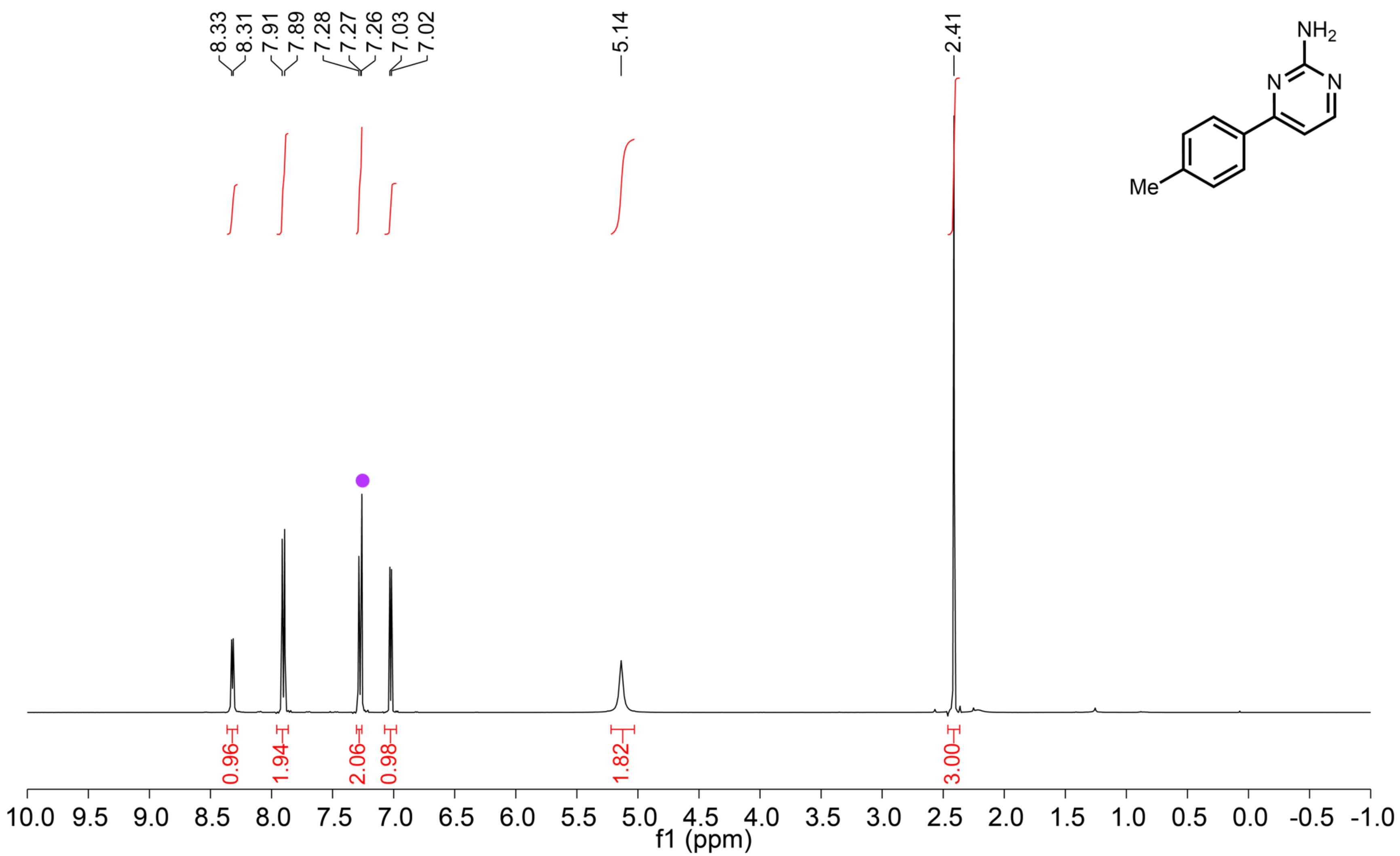

Figure S81. ^{1}H NMR (400.30 MHz, $CDCl_3$) spectrum of compound **49**. Residual proteo-solvent (•).

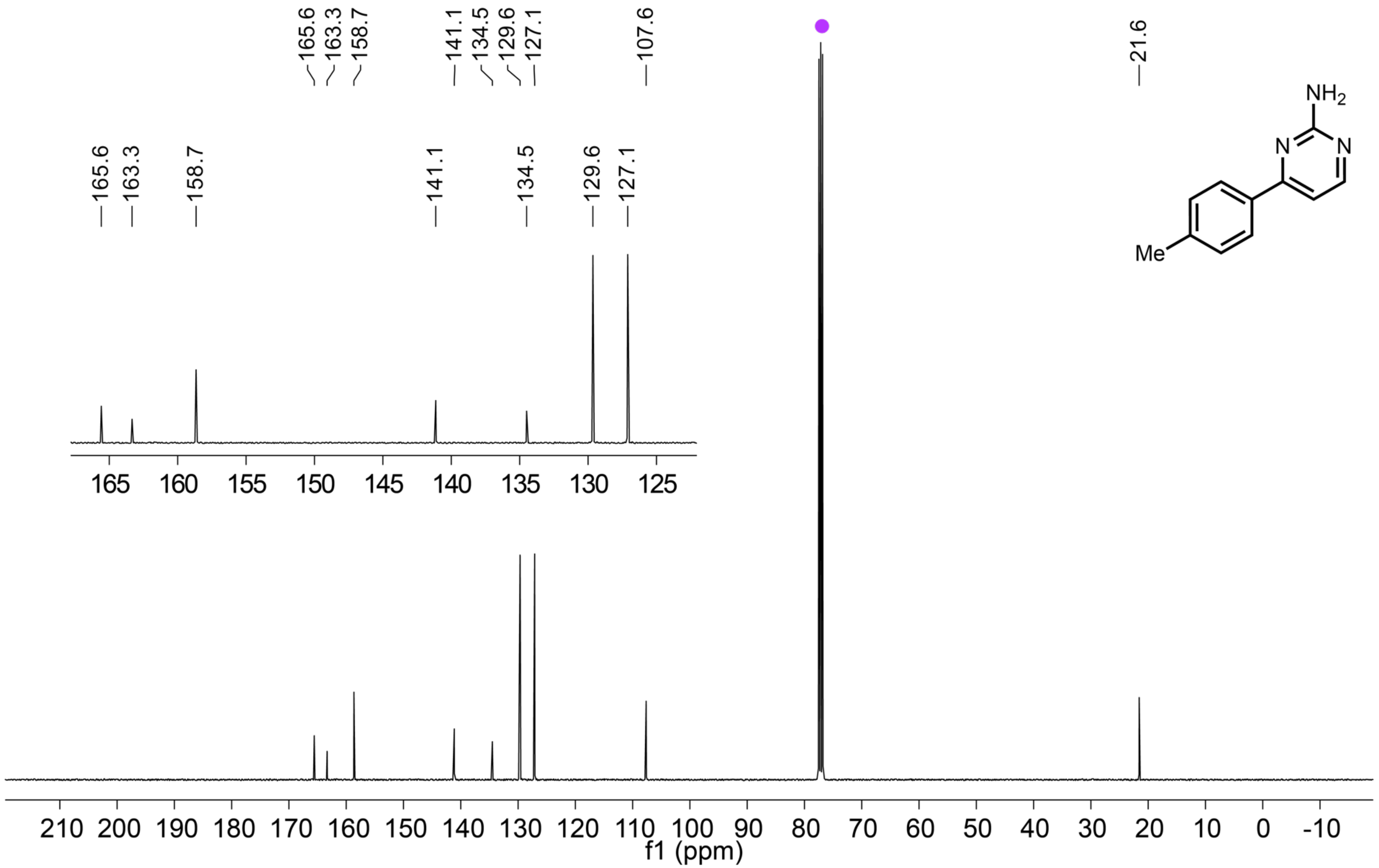

Figure S82. $^{13}C\{^1H\}$ NMR (100.67 MHz, $CDCl_3$) spectrum of compound **49**. Deuterated solvent (•).

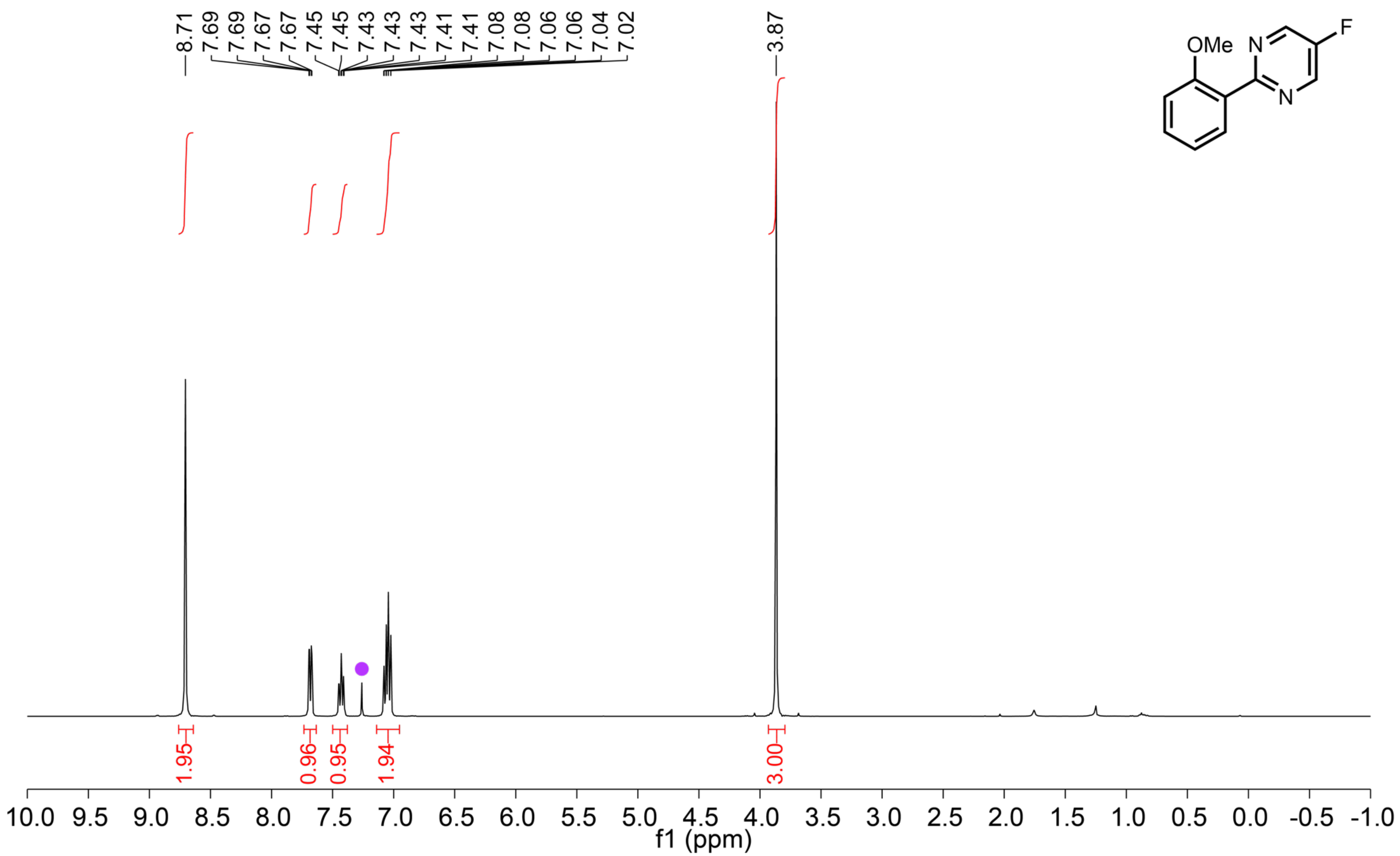

Figure S83. ^{1}H NMR (400.30 MHz, $CDCl_3$) spectrum of compound **50**. Residual proteo-solvent (•).

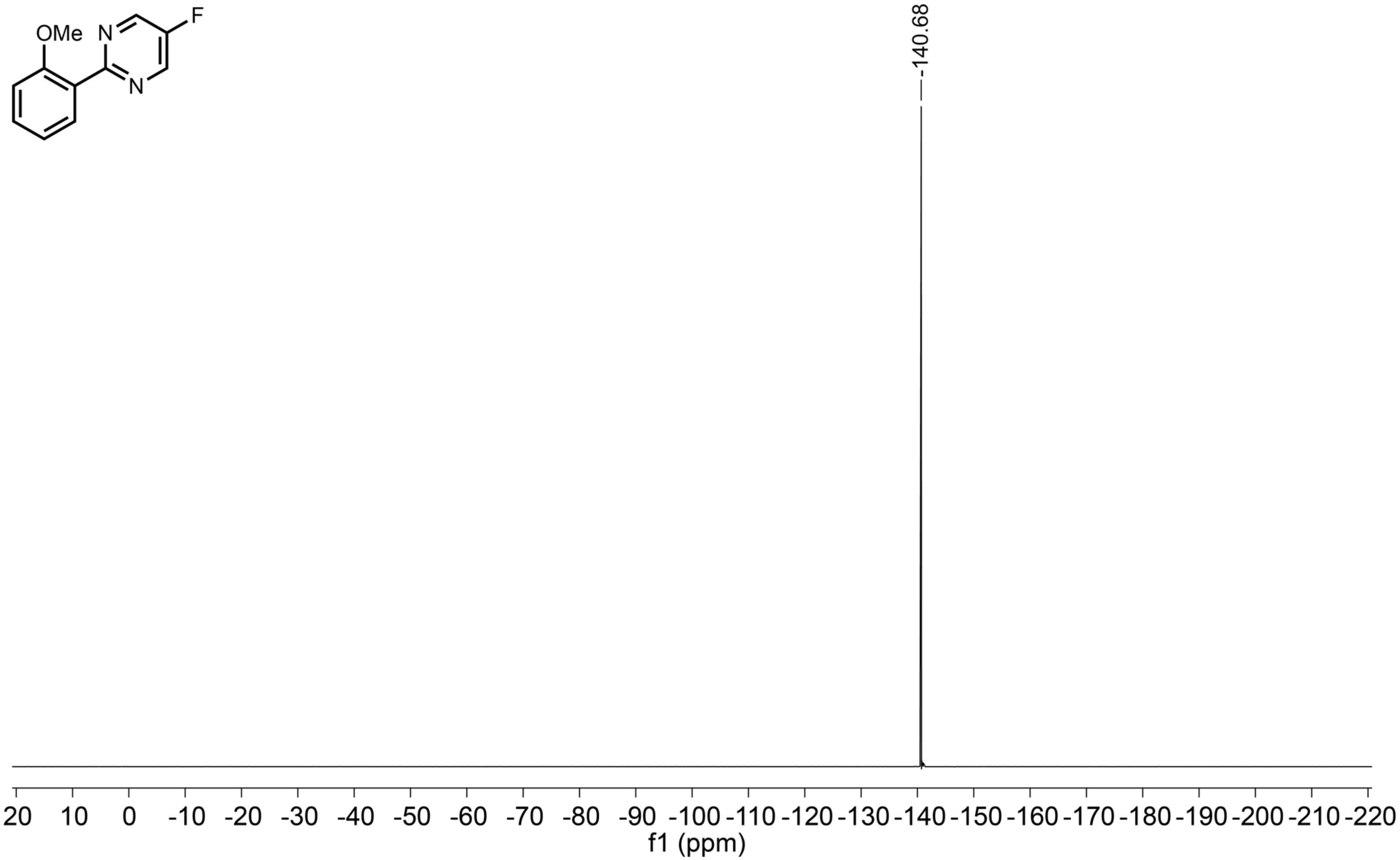

Figure S84. $^{19}F\{^{1}H\}$ NMR (470.61 MHz, $CDCl_3$) spectrum of compound **50**.

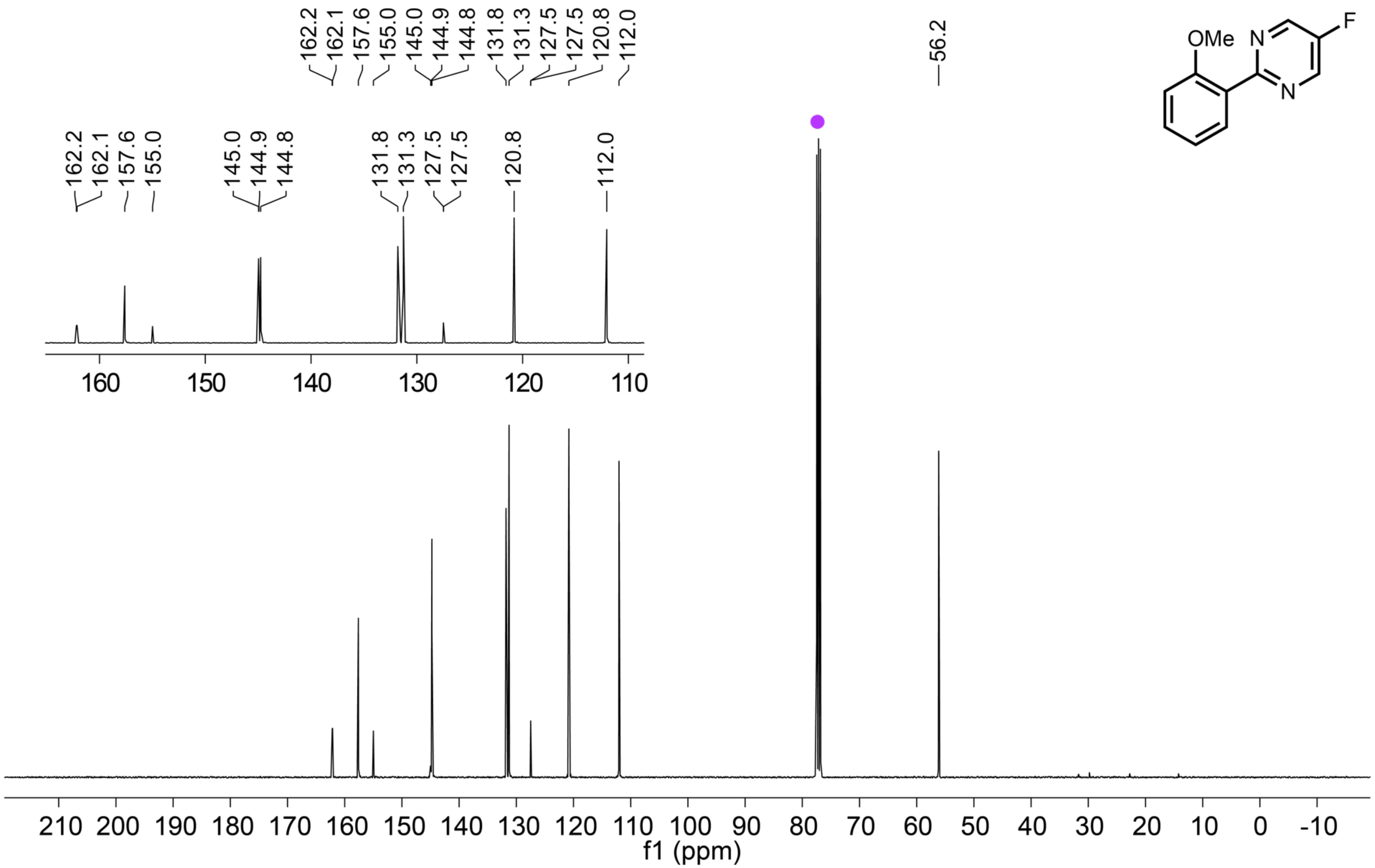

Figure S85. $^{13}C\{^{1}H\}$ NMR (100.67 MHz, $CDCl_3$) spectrum of compound **50**. Deuterated solvent (•).

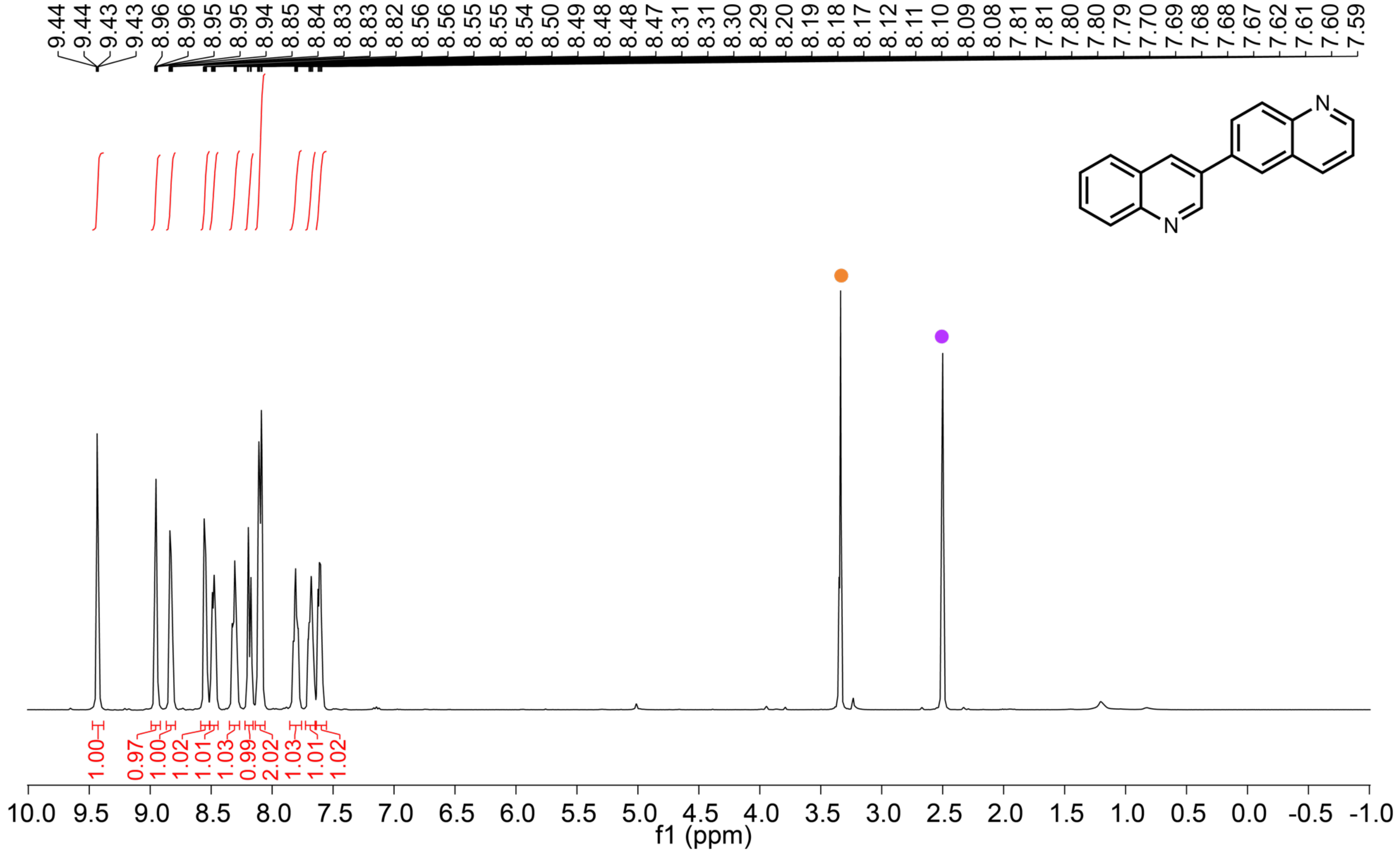

Figure S86. ^{1}H NMR (400.30 MHz, DMSO-d_6) spectrum of compound **51**. Residual proteo-solvent (•) and H_2O (•).

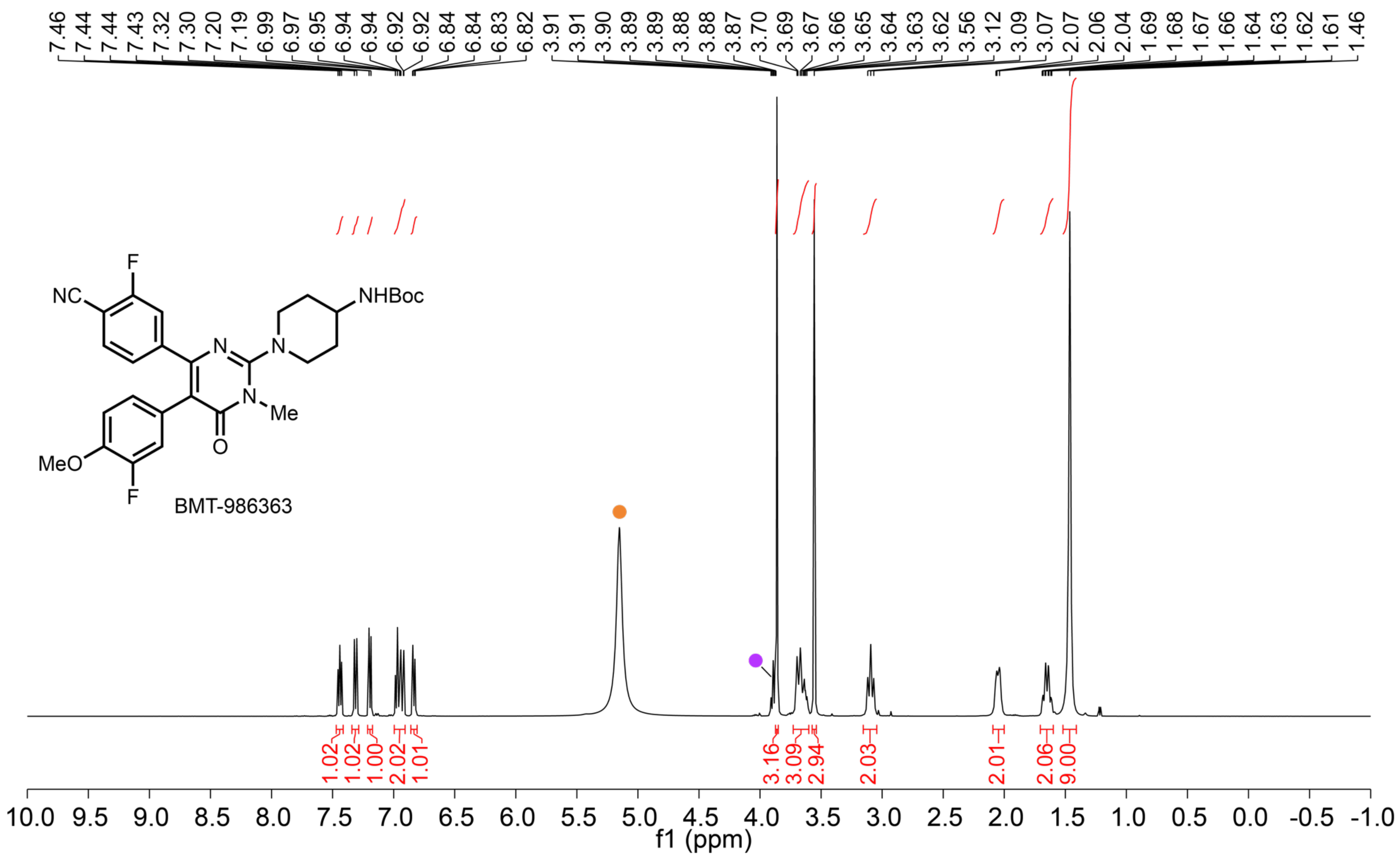

Figure S87. ^{1}H NMR (500.20 MHz, TFE-d_3) spectrum of BMT-986363 (**5**). H_2O (●) and residual proteo-solvent (●).

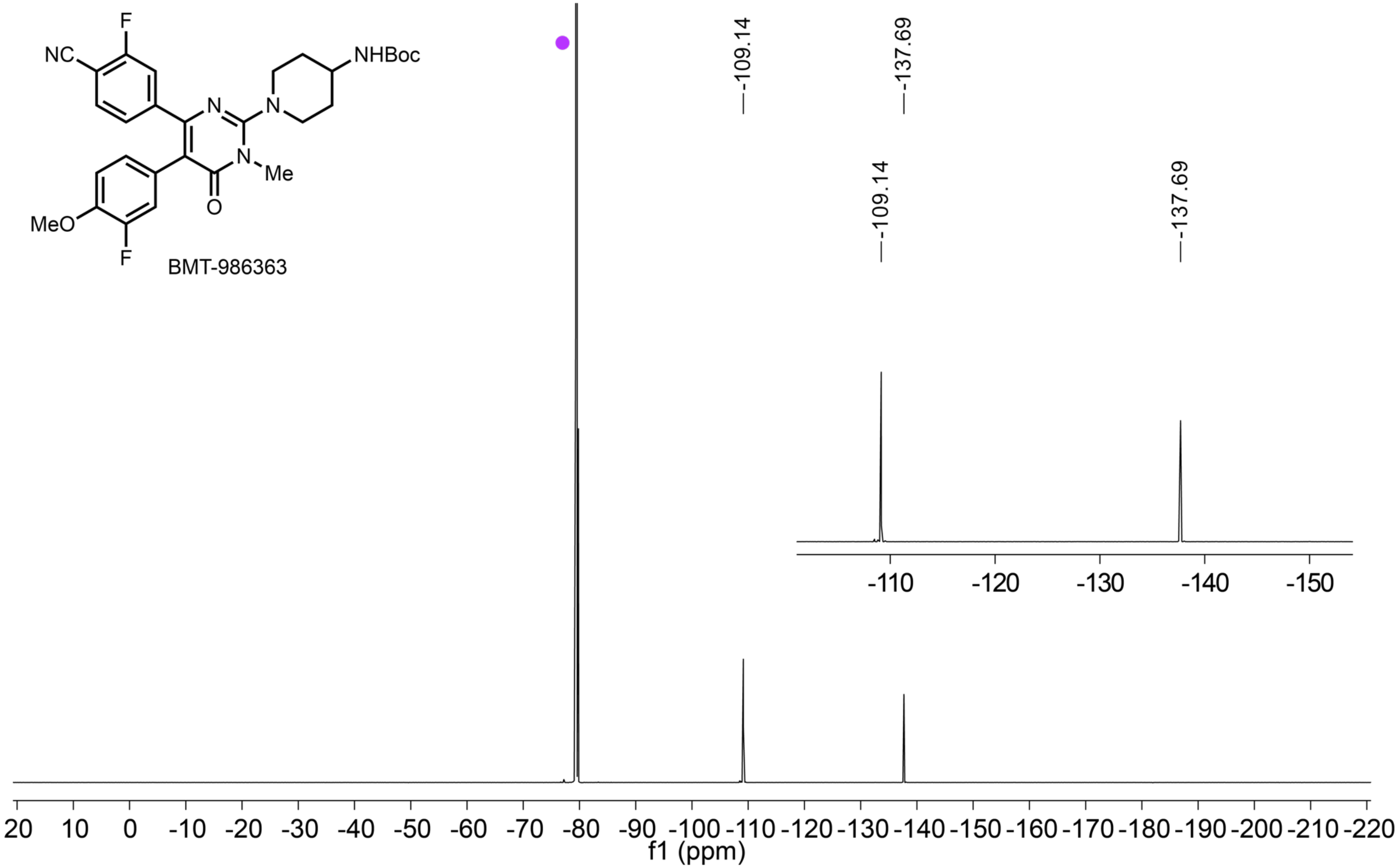

Figure S88. $^{19}F\{^{1}H\}$ NMR (470.61 MHz, TFE-d_3) spectrum of BMT-986363 (**5**). Residual proteo-solvent (•).

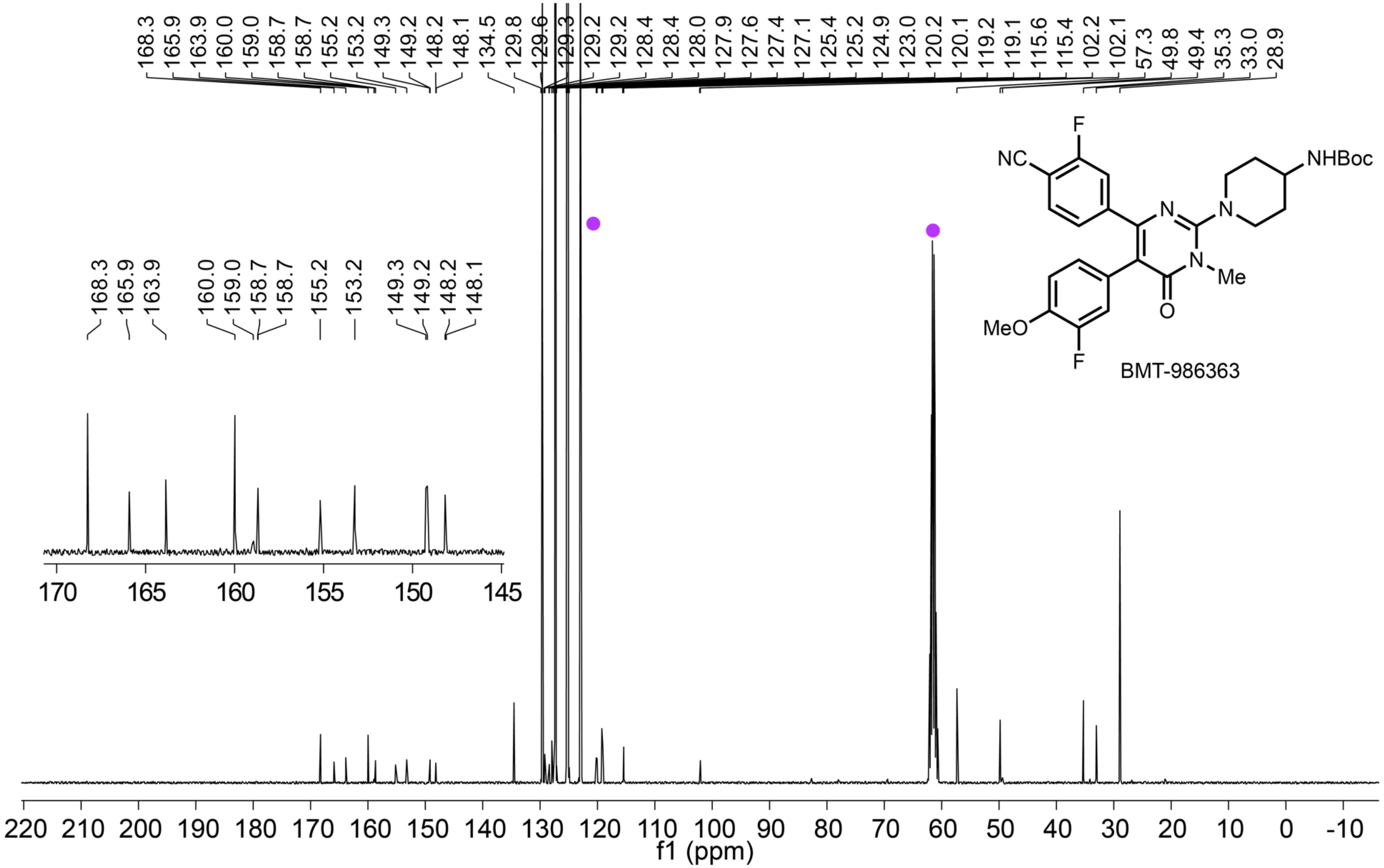

Figure S89. $^{13}C\{^1H\}$ NMR (125.79 MHz, TFE-d_3) spectrum of BMT-986363 (**5**). Deuterated solvent (•).

7. References

1. Yang, J.; Neary, M. C.; Diao, T., ProPhos: A Ligand for Promoting Nickel-Catalyzed Suzuki-Miyaura Coupling Inspired by Mechanistic Insights into Transmetalation. *J. Am. Chem. Soc.* **2024,** *146*, 6360-6368.
2. Magano, J.; Monfette, S., Development of an Air-Stable, Broadly Applicable Nickel Source for Nickel-Catalyzed Cross-Coupling. *ACS Catal.* **2015,** *5*, 3120-3123.
3. Jacobi, A.; Huttner, G.; Winterhalter, U., Tripod Ligands Containing a Mixed P/N/S Doner Set: Synthesis and Coordination Chemistry. *Chem. Ber.* **1997,** *130*, 1279-1294.
4. Kühl, O.; Blaurock, S.; Sieler, J.; Hey-Hawkins, E., Metallatriphos Complexes: Synthesis and Molecular Structure of [TpZr(OCH_2PPh_2)$_3$] (Tp=Tris(pyrazolyl)hydroborate) and Formation of the Heterodinuclear Complex [TpZr(μ-OCH_2PPh_2)$_3$Mo(CO)$_3$] with Bridging Phosphinoalkoxide Ligands. *Polyhedron* **2001,** *20*, 2171-2177.
5. Hope, H.; Olmstead, M. M.; Power, P. P.; Viggiano, M., A Nickel Complex Containing an Extremely Long Nickel-Phosphorus Bond. Syntheses and X-Ray Structures of $[Ni(CN)_2[P(CH_2OH)Ph_2]_3](C_6H_6)_{0.5}$ and *Trans*-$[Ni(CN)_2[P(CH_2\text{-}OH)Ph_2]_2]$. *Inorg. Chem.* **1984,** *23*, 326-330.
6. Wu, L.; Annibale, V. T.; Jiao, H.; Brookfield, A.; Collison, D.; Manners, I., Homo- and Heterodehydrocoupling of Phosphines Mediated by Alkali Metal Catalysts. *Nat. Commun.* **2019,** *10*, 2786.
7. Yang, J.; Zhao, H.; Schultz, J. E.; Wisniewski, S. R.; Simmons, E. M.; Diao, T., Process-Ready Nickel-Catalyzed Suzuki−Miyaura Coupling Enabled by *tri*-ProPhos. *ACS Catal.* **2025**, *15*, 19302-19311.
8. Newman-Stonebraker, S. H.; Smith, S. R.; Borowski, J. E.; Peters, E.; Gensch, T.; Johnson, H. C.; Sigman, M. S.; Doyle, A. G., Univariate Classification of Phosphine Ligation State and Reactivity in Cross-Coupling Catalysis. *Science* **2021,** *374*, 301-308.
9. Newman-Stonebraker, S. H.; Wang, J. Y.; Jeffrey, P. D.; Doyle, A. G., Structure-Reactivity Relationships of Buchwald-Type Phosphines in Nickel-Catalyzed Cross-Couplings. *J. Am. Chem. Soc.* **2022,** *144*, 19635-19648.
10. Borowski, J. E.; Newman-Stonebraker, S. H.; Doyle, A. G., Comparison of Monophosphine and Bisphosphine Precatalysts for Ni-Catalyzed Suzuki–Miyaura Cross-Coupling: Understanding the Role of the Ligation State in Catalysis. *ACS Catal.* **2023,** *13*, 7966-7977.

11. Gensch, T.; dos Passos Gomes, G.; Friederich, P.; Peters, E.; Gaudin, T.; Pollice, R.; Jorner, K.; Nigam, A.; Lindner-D'Addario, M.; Sigman, M. S.; Aspuru-Guzik, A., A Comprehensive Discovery Platform for Organophosphorus Ligands for Catalysis. *J. Am. Chem. Soc.* **2022,** *144*, 1205-1217.

12. Chen, G.-J.; Huang, J.; Gao, L.-X.; Han, F.-S., Nickel-Catalyzed Cross-Coupling of Phenols and Arylboronic Acids Through an In Situ Phenol Activation Mediated by PyBroP. *Chem. Eur. J.* **2011,** *17*, 4038-4042.

13. Janaagal, A.; Sanyam; Mondal, A.; Gupta, I., Robust Zinc(II)porphyrin Catalyst for Visible Light Induced C–H Arylation of Heteroarenes. *J. Org. Chem.* **2023,** *88*, 9424-9431.

14. Bratt, E.; Verho, O.; Johansson, M. J.; Bäckvall, J.-E., A General Suzuki Cross-Coupling Reaction of Heteroaromatics Catalyzed by Nanopalladium on Amino-Functionalized Siliceous Mesocellular Foam. *J. Org. Chem.* **2014,** *79*, 3946-3954.

15. Saoudi, B.; Le Floch, N.; Doucet, H., Evaluation of C3-Substituents on Pyrazoles as Remote Directing Groups in Palladium-Catalyzed Direct Arylation. *Tetrahedron* **2025,** *184*, 134783.

16. Cheng, H.; Wu, Q. Y.; Han, F.; Yang, G. F., Efficient Synthesis of 4-Substituted Pyrazole *via* Microwave-Promoted Suzuki Cross-Coupling Reaction. *Chin. Chem. Lett.* **2014,** *25*, 705-709.

17. Xu, X.; Xu, B.; Li, Y.; Hong, S. H., Abnormal N-Heterocyclic Carbene Promoted Suzuki−Miyaura Coupling Reaction: A Comparative Study. *Organometallics* **2010,** *29*, 6343-6349.

18. Liu, C.; Ji, C.-L.; Qin, Z.-X.; Hong, X.; Szostak, M., Synthesis of Biaryls via Decarbonylative Palladium-Catalyzed Suzuki-Miyaura Cross-Coupling of Carboxylic Acids. *iScience* **2019,** *19*, 749-759.

19. Haibach, M. C.; Ickes, A. R.; Tcyrulnikov, S.; Shekhar, S.; Monfette, S.; Swiatowiec, R.; Kotecki, B. J.; Wang, J.; Wall, A. L.; Henry, R. F.; Hansen, E. C., Enabling Suzuki–Miyaura Coupling of Lewis-Basic Arylboronic Esters with a Nonprecious Metal Catalyst. *Chem. Sci.* **2022,** *13*, 12906-12912.

20. Takale, B. S.; Thakore, R. R.; Casotti, G.; Li, X.; Gallou, F.; Lipshutz, B. H., Mild and Robust Stille Reactions in Water using Parts Per Million Levels of a Triphenylphosphine-Based Palladacycle. *Angew. Chem. Int. Ed.* **2021,** *60*, 4158-4163.

21. Cervantes-Reyes, A.; Smith, A. C.; Chinigo, G. M.; Blakemore, D. C.; Szostak, M., Decarbonylative Pd-Catalyzed Suzuki Cross-Coupling for the Synthesis of Structurally Diverse Heterobiaryls. *Org. Lett.* **2022,** *24*, 1678-1683.

22. Zhao, M. M.; Zhang, H. M.; Iimura, S.; Bednarz, M. S.; Kanamarlapudi, R. C.; Yan, J.; Lim, N. K.; Wu, W. X., Process Development of Tryptophan Hydroxylase Inhibitor LX1031, a Drug

Candidate for the Treatment of Irritable Bowel Syndrome. *Org. Process Res. Dev.* **2020,** *24*, 261-273.

23. Chen, W.; Li, Y.; Zhou, Y.; Ma, Y.; Li, Z., Design, Synthesis and SAR Study of Novel Sulfonylurea Derivatives Containing Arylpyrimidine Moieties as Potential Anti-Phytopathogenic Fungal Agents. *Chin. Chem. Lett.* **2019,** *30*, 2160-2162.

24. Guo, X.; Dang, H.; Wisniewski, S. R.; Simmons, E. M., Nickel-Catalyzed Suzuki–Miyaura Cross-Coupling Facilitated by a Weak Amine Base with Water as a Cosolvent. *Organometallics* **2022,** *41*, 1269-1274.
